# The compact genome of the sponge *Oopsacas minuta* (Hexactinellida) is lacking key metazoan core genes

**DOI:** 10.1186/s12915-023-01619-w

**Published:** 2023-06-19

**Authors:** Sébastien Santini, Quentin Schenkelaars, Cyril Jourda, Marc Duchesne, Hassiba Belahbib, Caroline Rocher, Marjorie Selva, Ana Riesgo, Michel Vervoort, Sally P. Leys, Laurent Kodjabachian, André Le Bivic, Carole Borchiellini, Jean-Michel Claverie, Emmanuelle Renard

**Affiliations:** 1 Aix Marseille Univ, CNRS, IGS, UMR 7256, IMM, IM2B, IOM, Marseille, France; 2 Aix Marseille Univ, Avignon Univ, CNRS, IRD, IMBE, Marseille, France; 3 Institut Jacques Monod, CNRS, UMR 7592, Univ Paris Diderot, Sorbonne Paris Cité, Paris, France; 4 CIRAD, UMR PVBMT, La Réunion, France; 5 Department of Biological Sciences, University of Alberta, Edmonton, AB T6G 2E9 Canada; 6 Department of Biodiversity and Evolutionary Biology, Madrid, Spain; 7 Department of Life Sciences, Natural History Museum of London, London, SW7 5BD UK; 8 Aix Marseille Univ, CNRS, IBDM, UMR 7288, Turing Center for Living Systems, Marseille, France; 9 Aix Marseille Univ, CNRS, IBDM, UMR 7288, Marseille, France

**Keywords:** Microbiome, Symbiosis, Epithelium, Ciliogenesis, Signaling pathways, Transcription factors, Photoreception, Signal transduction, Skeleton, Silicification

## Abstract

**Background:**

Explaining the emergence of the hallmarks of bilaterians is a central focus of evolutionary developmental biology—evodevo—and evolutionary genomics. For this purpose, we must both expand and also refine our knowledge of non-bilaterian genomes, especially by studying early branching animals, in particular those in the metazoan phylum Porifera.

**Results:**

We present a comprehensive analysis of the first whole genome of a glass sponge, *Oopsacas minuta*, a member of the Hexactinellida. Studying this class of sponge is evolutionary relevant because it differs from the three other Porifera classes in terms of development, tissue organization, ecology, and physiology. Although *O. minuta* does not exhibit drastic body simplifications, its genome is among the smallest of animal genomes sequenced so far, and surprisingly lacks several metazoan core genes (including Wnt and several key transcription factors). Our study also provides the complete genome of a symbiotic Archaea dominating the associated microbial community: a new *Thaumarchaeota* species.

**Conclusions:**

The genome of the glass sponge *O. minuta* differs from all other available sponge genomes by its compactness and smaller number of encoded proteins. The unexpected loss of numerous genes previously considered ancestral and pivotal for metazoan morphogenetic processes most likely reflects the peculiar syncytial tissue organization in this group. Our work further documents the importance of convergence during animal evolution, with multiple convergent evolution of septate-like junctions, electrical-signaling and multiciliated cells in metazoans.

**Supplementary Information:**

The online version contains supplementary material available at 10.1186/s12915-023-01619-w.

## Background

Understanding the early steps of animal evolution is one of the major challenges of evolutionary biology. One way to achieve this goal is to compare genomic data across non-bilaterian animals (Ctenophora, Placozoa, Porifera, Cnidaria) [1–3].

**Fig. 1:**
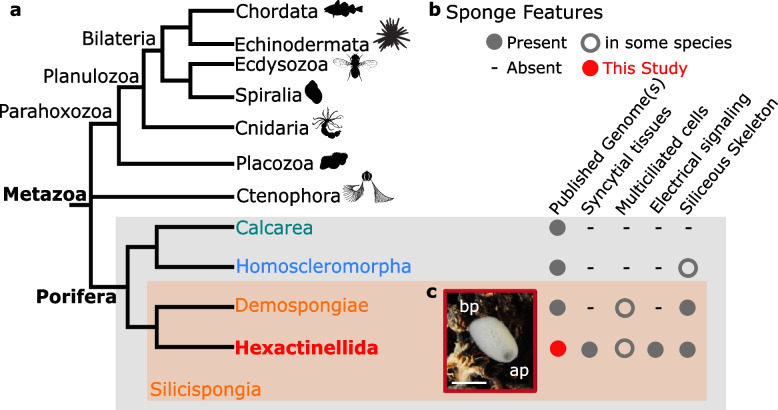
General features of the hexactinellid sponge *Oopsacas minuta*. **a** Phylogenetic position of *O. minuta*: this species belongs to the phylum Porifera (sponges), one of the best candidates as sister group to all other metazoans. Free animal silhouettes were downloaded from PhyloPic (http://phylopic.org/). Among the four lineages of Porifera (> 9500 species), *O. minuta* belongs to Hexactinellida (glass sponges). **b** Hexactinellida (in red) have particular features (e.g., syncytial tissues) compared to the three other sponge classes, Demospongiae (in orange), Homoscleromorpha (in blue), and Calcarea (in green). **c*** O. minuta* adults inhabit Mediterranean canyons and shallow caves (photo credit Dorian Guillemain), where individuals are 1–7 cm long and have a clear basal–apical polarity (ap apical pole, bp basal pole) (scale bar = 1 cm)

As one of the best candidates for sister group to all other animals [4–10] (Fig. 1a), sponges (Porifera) are of particular interest. Although the biology of Porifera is still poorly known [11], their ancient origin (> 600 million years (Myrs)) [12] has given rise to a phylum with over 9500 described species [13] distributed among four classes, with diverse ecological, embryological, cellular, and morphological features [14–17] (Fig. 1a, b).

The first sponge genome sequenced, from the demosponge *Amphimedon queenslandica*, revealed a larger size and gene content than expected [18–20]. However, transcriptomic data from other species indicated that *A. queenslandica* was not representative of the diversity of sponges [17, 21–26]. Several sponge genomes now sequenced [27–31] illustrate the disparity of sponge genomes in terms of size, features of non-coding regions, and gene repertoire [3, 28]. However, the lack of a complete genome from the Hexactinellida (termed “glass sponges” for their often heavily siliceous skeleton) (Fig. 1b) has remained a major gap in our knowledge of Porifera, despite the insights provided by snapshots of their gene contents [3, 23, 26, 32, 33]. A comprehensive description of the genomic features of a glass sponge will show exactly how much they differ from sponges in other classes and will identify key genomic changes associated with the diversification of sponges into four markedly different classes.

Here, we provide the first high-quality genome of a glass sponge. The species *Oopsacas minuta* (Topsent 1927) (Fig. 1c) was chosen for its unambiguous taxonomic identification, its accessibility at shallow depths in Mediterranean caves, and because of important aspects of its embryology and biology that have been studied previously [34–36]. Given the range of unusual characteristics of Hexactinellida including their syncytial organization of tissues, their ability to propagate electrical signals, and their adaptation to the deep-sea habitat, we focused our bioinformatic analyses on genes involved in development, epithelia and multiciliogenesis, silicate biogenesis, and signal transduction. We also gathered data on the dominant microbiome associated with *O. minuta*.

## Results

### Initial metagenomic data processing

The data obtained from the DNA (deoxyribonucleic acid) mixture extracted from non-clonal specimens collected from their natural environment were initially processed as sequences from a low-complexity metagenome consisting of the sponge nuclear genome, its mitochondrial genome, and of an undescribed population of microorganisms including non-resident (food) and resident (symbionts *sensu lato* [37]) species.

The combination of sequence data (from two complementary platforms Pacific Biosciences (PacBio) and Illumina) yielded an initial dataset of 1759 scaffolds larger than 1 kb (kilo base) that were first classified as Eukaryota, Bacteria, Archaea, or virus sequences. Further steps in refining the assembly resulted in three distinctive sets of super-scaffolds associated with large coverage values: the genome of a Thaumarchaeota (coverage = 1206), the *O. minuta* nuclear genome (coverage = 186), and the mitochondrial genome (coverage = 455). A number of other scaffolds with smaller coverage were attributed to the residual microbiome of the sponge (Additional file 1: Table S1). The fully assembled mitochondrial genome was published previously [38].

### The dominance of a Thaumarchaeota symbiont

Data on hexactinellid microbiomes are scarce, and histological observations suggest that bacterial symbionts are rare [36, 39, 40].

**Fig. 2:**
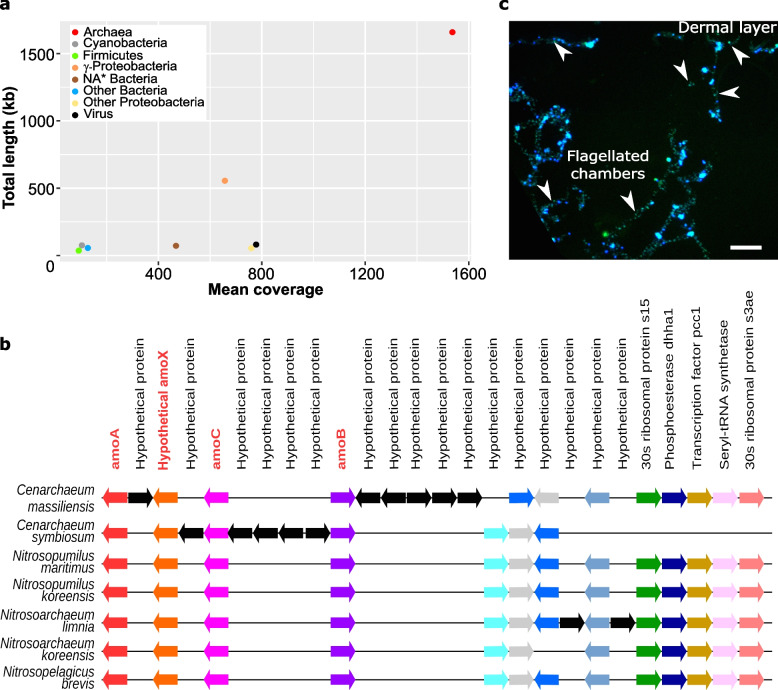
Characterization of the microbiome associated with *O. minuta*. **a** Relative coverage and sequence size of non-eukaryotic contigs according to their taxa (Table S1, Additional file 1). The microbiome of *O. minuta* is dominated by a new *Thaumarchaeota* species that we propose to name *Candidatus Cenarchaeum massiliensis* and various proteobacteria. **b** Microsynteny analysis of genomic segments bearing an *amo* gene cluster in marine Thaumarchaeota showing that the genome *Candidatus Cenarchaeum massiliensis* has features highly conserved with other marine Thaumarchaeota (arrows of the same color indicate orthologous genes and black arrows represent genes without orthology relationship in these regions from OrthoMCL analysis). Other data concerning *Ca. C. massiliensis* are available in Tables S3 and S4 and Figs. S1, S2, and S3 of Additional file 1. **c** Localization of *Ca. C. massiliensis* in the tissues of *O. minuta* by Card-FISH using a specific probe (blue = DAPI staining; green = labeled probe) shows the presence of this symbiont in the whole trabecular syncytium including the dermal layer and the flagellated chambers (see also Fig. S4, Additional file 1)

Of the 1759 scaffolds, 107 scaffolds were assigned to Bacteria (more than 11 phyla) dominated by γ-proteobacteria (Fig. 2a; Additional file 1: Table S2). In addition, one of the two viral contigs was affiliated with the *Circoviridae*, a family of small single-stranded DNA viruses. Surprisingly for an animal that filters sea water where viruses are abundant [41–43], viruses have rarely been observed in sponges [44, 45] and few sponge viromes are available [46–49]. Nevertheless, *Circoviridae*-related sequences have been reported previously in two demosponges [49]. Known circoviruses tend to be associated with vertebrate hosts and are considered rare in marine invertebrates [50], highlighting the novelty of our finding [51].

Besides a more diverse microbiome than previously reported [34–36], the most visible feature emerging from the sequence assembly was the presence of 11 super-scaffolds of a Thaumarchaeota-like genome associated with the highest coverage value (Fig. 2a; Additional file 1: Table S1). This coverage corresponds to a ratio of about 13 archaean cells per sponge cell (presumed diploid according to the few sponge karyotypes available [52]). This Thaumarchaeota species represents the main microorganism associated with *O. minuta*. Its global protein sequence similarity with the NR (non-redundant) databases and phylogenetic analyses strongly suggest that it is a new representative of the *Cenarchaeum* genus that we propose to name *Candidatus Cenarchaeum massiliensis (Ca. C. massiliensis*) (Fig. 2b; Additional file 1: Fig. S1).

The partially assembled genome sequence of *Ca. C. massiliensis* has a size of about 1.63 Mb (Megabases), a G + C content (guanine + cytosine content) of 37.3 ± 2.5%, an estimated completeness of 99.03% [53], and a relatively low coding density (83.9%) compared to other ammonium-oxidizing archaea (AOA) species (Additional file 1: Table S3). The orthology analysis of 23,013 predicted proteins from ten Thaumarcheota species found 3229 ortholog groups (gene families) using OrthoMCL algorithm and database (Additional file 1: Fig. S2a). Among them, 1517 (47%) were found only in one genus and a large part (1158 gene families) was found in only *Nitrososphaera* genus (representing 36% of total Thaumarcheota gene families). A large part of the Thaumarcheota pan genome is found in the *Nitrososphaera* genus that could be explained by its metabolic versatility and environmental adaptations [54–57].

We identified 908 gene families conserved across the five genera of Thaumarchaeota representing the core genome of Thaumarcheota (28% of total Thaumarcheota gene families). Among the conserved gene families, a large part corresponds to unknown function (184 (20%)) and another large part encodes ribosomal proteins, translation factors, or aminoacyl-tRNA synthetases (125 (14%)) (Supplementary Fig. 2b, Additional file 1). We also identified core gene families potentially involved in amino acid and nucleotide metabolism (74 (8%) and 39 (4%), respectively). In addition, we found 41 core gene families (4.5%) involved in transcription including transcription factors and RNA polymerases. The analysis identified 41 core families (4.5%) involved in transport including peptide transporters, aquaporins, cation/ion transporters, and ATPases. Unexpectedly, the metabolism of cofactors and vitamins represented a large part of core Thaumarchaeota gene families (66 (7%)) including essential genes for cobalamin biosynthesis (Additional file 1: Figs. S2 and S3). Among the 908 core gene families, the orthoMCL analysis identified 132 (14.5%) Thaumarchaeota-specific gene families (not found in another species in the OrthoMCL database) corresponding to the Thaumarcheota trademark protein set, including specific ribosomal proteins (presence of r-proteins s26e, s25e, and s30e but absence of the r-proteins L14e and L34e), DNA topoisomerase IB and subunits of ammonia monooxygenase (Amo) (Fig. 2b; Additional file 1: Table S4).

Interestingly, as much as 147 transposase encoding genes are present in this species. This number is among the largest reported to date in a thaumarcheotal genome [58, 59].

Using Card-FISH (catalyzed reporter deposition fluorescent in situ hybridization), we localized *Ca. C. massiliensis* in the tissues of *O. minuta* with a specific probe. This microorganism is present in the trabecular syncytium (Fig. 2c; Additional file 1: Fig. S4). Similar localizations were observed in different individuals sampled at different periods. Taken together, the abundance and the durability of the association suggest that *Ca. C. massiliensis* is a symbiont of *O. minuta*.

Our finding further supports the hypothesis that a stable Thaumarchaeota-Porifera relationships might be based on the ability of all Thaumarchaeota to oxidize ammonia in nitrite and to produce cobalamin anaerobically [58]. Such capabilities are absent in animals while cobalamin is essential for their life cycle [60, 61]. We propose that the symbiotic *Ca. C. massiliensis* may provide cobalamin to *O. minuta* while recycling the ammonia produced by its metabolism [62].

### *O. minuta* genomic features

**Fig. 3:**
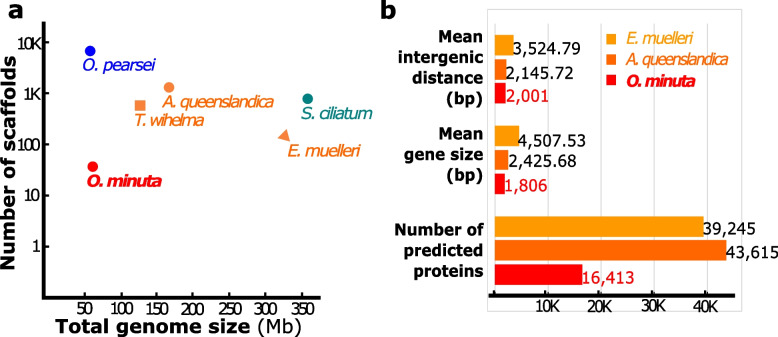
General features of the nuclear genome of *Oopsacas minuta*, compared to that of other sponges. **a** The assembled genome of *O. minuta* is one of the smallest so far described in sponges. **b** The *O. minuta* genome has fewer predicted proteins and is slightly more compact than the genomes of *Amphimedon queenslandica* and *Ephydatia muelleri*, both demosponges, which is the sister group to hexactinellids. Other genomic features are available in Additional file 2: Table S5, Table S6, and Fig. S5

The *O. minuta* genome was assembled into 365 scaffolds. Its G + C content is 36 ± 2.1%. The quality and low fragmentation of the assembly is attested by a N50 value of 0.67 Mb comprising 31 sequences. The estimated total genome size is about 61 Mb, thus two- to six fold smaller compared to Demospongiae and Calcarea (Fig. 3a; Additional file 2: Table S5a). It is one of the smallest sponge genomes reported so far (the other being *Oscarella pearsei*: 57.7 Mb) [28]. Such differences in genome sizes are consistent with previous estimations of DNA content [63].

The variation in genome sizes of closely related eukaryotic organisms can arise from the difference in their gene densities and/or numbers. The average number of introns per gene, the average intron size, and the average intergenic distance all govern gene density. In *O. minuta*, all of these are similar or only slightly lower than those in demosponges: 1.59 intron/gene, average intron size = 341 bp (base pairs), average intergenic distance = 2 kb (Fig. 3b; Additional file 2: Table S5b). Thus, the main reason *O. minuta* has the most compact sponge genome assembled so far is because it encodes far fewer proteins (16,413) than other sponge genomes: 39,245 for *Ephydatia muelleri* [30], 40,122 for *Amphimedon queenslandica* [19], and 37,416 for *Tethya wilhelma* [29]. This observation is validated by the high number of conserved core eukaryotic genes identified in the *O. minuta* draft sequence (91 to 93% using BUSCO (Benchmarking Universal Single-Copy Orthologs) [64]) attesting to its completeness. Through a first automated pass of functional annotation, 7737 protein-coding genes were attributed a Gene Ontology (GO) annotation (Additional file 2: Table S6 and Fig. S5).

The global orthology comparison between *Oopsacas* and other sponge species confirms this difference in gene content. Of the total number of genes, 86.6% were assigned to 24,721 orthogroups. For each species, the percentage of unassigned genes varies from 5.7% (*A. queenslandica*) to 20.3 (*Sycon ciliatum*). *Oopsacas minuta* shares only 36 to 40% of its orthologs with other sponges. It is further noticeable that 761 orthogroups shared by all other sponges are not present in *Oopsacas* (Additional file 2: Fig. S6a). These losses in glass sponges mainly concern enzymatic activities, biomolecule binding properties, and metabolic processes (Additional file 2: Fig. S6b). These findings suggest that *Oopsacas* differs from other sponges in its physiology and call for further experimental studies on this lineage. Other gene losses concern sensing processes, and regulation of cellular and developmental processes, some of which are detailed in the next sections. On the other hand, 637 orthogroups appear to be specific to the hexactinellid lineage as not being identified in the three other sponge lineages: again, GO terms point to probable specificities regarding enzymatic activities, metabolism, binding, cell communication, and signal transduction. We also noticed orthogroups related to transposition [65].

Interspersed repeats represent 34.10% of the genome, and these are mostly represented by unclassified repeats (18.01%) and DNA transposons (15.44%) (Additional file 2: Tables S7 and S8).

### The epithelial gene set of a syncytial sponge

The unique syncytial organization of Hexactinellida [36, 66–69] raises exciting questions regarding the genes involved in the epithelial characteristics: cell polarity, cell junctions (CJs), and basement membrane (BM) [18, 70–76]. A previous survey [32] showed that *Oopsacas* possesses the whole set of genes encoding proteins involved in the polarity complexes (PAR, CRUMBS, SCRIBBLE), and in cadherin-catenin complexes (CCC) despite the apparent absence of conventional adherens junctions (AJs), suggesting that these proteins may have different functions in sponges [75]. Here we searched for proteins involved in other types of CJs and in the BM.

**Fig. 4:**
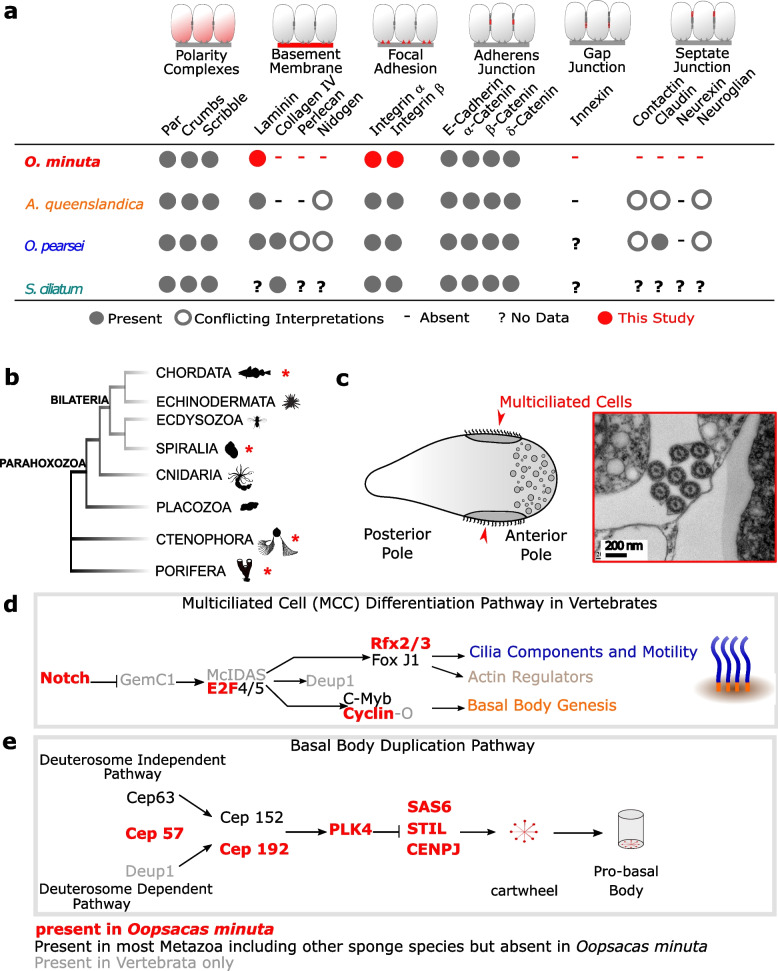
Survey of genes associated with tissue features of *O. minuta*. **a** Presence/absence in *O. minuta,* which has syncytial tissues, of genes involved in epithelial functions (in Bilateria) compared to sponges in the three other poriferan classes, which have cellular tissues. **b** Phylogenetic relationships between metazoan taxa where multiciliated cells (MCC) have been reported (red stars). **c*** O. minuta* larvae possess MCC, seen clearly in only one other group of deep-water demosponges (cladorhizids). The diagram of a larva (on the left) shows the position of MCC (red arrows) several cilia are visible in cross section in the TEM picture on the right (photo credits Sally Leys). **d** Survey of genes involved in MCC differentiation in vertebrates. **e** Survey of genes involved in ciliogenesis and basal body duplication in bilaterians

Three types of cell–cell junctions are defined, namely adherens, gap, and septate junctions (AJs, GJs, and SJs) [75, 77–81]. As for other sponge lineages, no innexin-related genes, involved in GJs, were found in this genome. This is in agreement with (i) the observation that glass sponge plugged junctions are specialized cytoplasmic structures that lie within a cytoplasmic bridge, in contrast to gap junctions which are channels within and between membranes of two apposed cells [67] and (ii) the fact that no gap junctions have been reported so far in any sponge lineage [67]. The content of the glass sponge proteinaceous plugged junction remains to be determined [36, 66]. Of SJ proteins, neither Claudin, Neuroglian, Neurexin IV, nor Contactin were found. Regarding Contactin (Cont), which belongs to the immunoglobulin superfamily (IgSF), neither our best blast hit (Additional file 3: Table S9) nor the sequence from *Aphrocallites vastus* annotated as Contactin [23] exhibit the characteristic glycosylphosphatidylinositol (GPI) anchor domain, supporting the absence of Cont in glass sponges (Fig. 4a). Finally, glass sponges have septae reminiscent of SJs [69] but do not encode the proteins involved in bilaterian SJ, suggesting these are convergent structures.

In cell-to-matrix junctions, the bilaterian focal adhesions (FAs) and hemidesmosomes (HDs) are based on interactions between integrins, proteins of the extracellular matrix (ECM), and the actin network [82–85]. We found a diversified set of integrins as in other sponges [20, 23, 30, 86, 87]. Five were assigned to the alpha chain family and three to the beta chain family. Their predicted domain structures are similar to those reported in other animals (Additional file 3: Fig. S7a).

Among the main components of the BM (laminins (Lam), type IV collagen, nidogen, and perlecan [71, 88–91]), only four laminins were found (Fig. 4a) with a shared characteristic domain architecture consisting in one α-like laminin-related protein, two chimeric Lamβ/γ-like chains as in *A. queenslandica* [92, 93] and a laminin γ-like chain with a characteristic single LamIVA domain (Additional file 3: Fig. S7b). Both our and Fahey’s surveys [92] suggest that α, β/γ, and γ-like laminin chain types were present in the last common ancestor of metazoans (LCAM). In contrast, a β-chain is identified neither in the demosponge *Amphimedon* nor in Hexactinellida, suggesting this type of laminin emerged later. When reassessing (by domain prediction) the sequence annotated as nidogen in *Aphrocallistes vastus* [23], we only identified the NIDO domain, which is not specific to nidogen. The absence of all components of the BM, except laminins, confirms the absence of BM in glass sponges, in agreement with morphological observations and previous transcriptomic analyses [18, 23, 71–73, 75, 94]. Type IV collagen was probably present in the LCAM and secondarily lost several times [20, 23, 30, 95]. In contrast, perlecan and nidogen probably emerged more recently [75].

### Multiciliogenesis

Multiciliated cells (MCCs) have been described in Vertebrata [96], Spiralia, Ctenophora, and Porifera [97–101] (Fig. 4b). Vertebrates and protostomes share the same core set of proteins required for centriole formation and duplication (PLK4 (*polo-like kinase 4*), SAS6 (*spindle assembly abnormal 6)*, and STIL (*SCL/TAL1 interrupting locus*)) but not the upstream regulatory network [102–105]. Three evolutionary scenarios are possible: (1) the upstream regulators of vertebrate MCCs are ancestral and were lost/replaced in protostomes, (2) the (unknown) upstream regulators of protostomes are ancestral and were lost/replaced in vertebrates, and (3) none of the upstream regulators are ancestral and MCCs emerged several times in Metazoa. Data from non-bilaterians are needed to assess these scenarios. *O. minuta* is one of the few sponge species having MCCs in the larva (Fig. 4c) [34, 66, 68, 94, 106, 107]. We therefore searched for genes involved in MCC differentiation and centriole duplication in bilaterians.

Except for the Notch signaling pathway (next section), neither the vertebrate upstream regulators (Mcidas *(multiciliate differentiation and DNA synthesis associated cell)*, GMNC/GEMC1 *(geminin coiled-coil domain containing)* and the transcription factor E2F4) nor their targets (FoxJ1 *(forkhead box J1)*, c-Myb *(myeloblastosis proto-oncogen)*, Deup 1 *(deuterosome assembly protein 1)*, Cyclin O (CCNO)) involved in MCC differentiation were found (Fig. 4d; Additional file 3: Table S10). The absence of FoxJ and Myb-related genes is unexpected, because they have an ancient origin [108, 109], and the latter has already been reported in sponges [110]. This suggests secondary losses of these two transcription factors in hexactinellids.

Among the key upstream proteins involved in bilaterian centriole duplication (Fig. 4e), we found genes encoding centrosome-associated proteins (CEP) CEP192 and CEP57, but not CEP63 and CEP152 (Additional file 3: Table S10), suggesting that centriole duplication may be initiated by a different proteomic network in *Oopsacas* than in bilaterians. In contrast, we found PLK4, SAS6, STIL, and CENPJ *(Centromere Protein J)*, in agreement with their conserved function in centriole duplication across Eukaryota [111].

The above findings suggest that the formation of multiciliated cells in *Oopsacas* may involve common downstream terminal effectors, but that upstream regulators are different from those described in vertebrates and protostomes. In other words, sponge and bilaterian MCCs probably result from convergent evolution, as previously proposed on the basis of ultrastructural [105] and embryological [66] observations.

### Signaling pathways

In metazoans, conserved signaling pathways are critical transduction cascades [112–118]. Surprisingly, previous transcriptomic analyses have suggested that key components of the canonical Wnt pathway are absent in glass sponges [23, 26, 119].

**Fig. 5:**
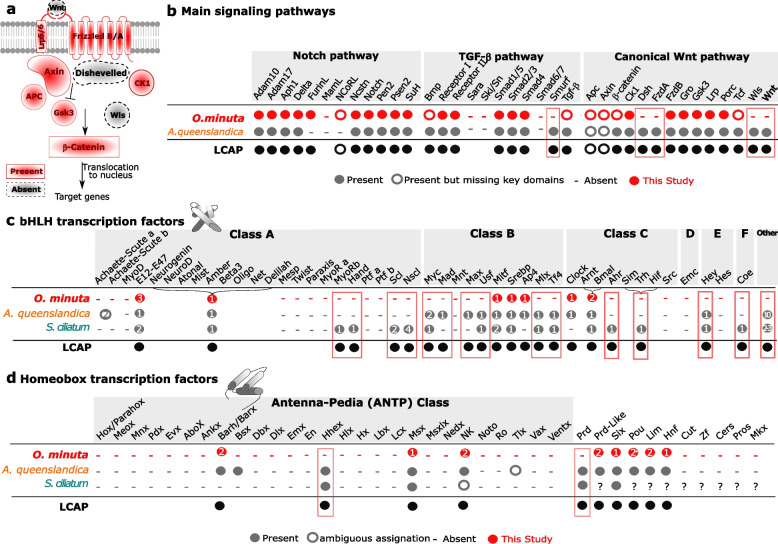
Survey of key genes involved in the metazoan developmental toolkit. **a** Presence/absence of genes involved in the canonical Wnt pathway: the absence of Wnt and dishevelled genes is an unusual feature suggesting the use of alternative pathways. **b** Survey of genes involved in three critical signaling pathways in *O. minuta* compared to the demosponge *Amphimedon queenslandica:* core members of Notch and TGF-β are present. **c** Inventory of basic Helix loop Helix (bHLH) transcription factors present in *O. minuta* compared to two other sponges. Numerous genes are absent in *O. minuta*. **d** Inventory of Homeobox transcription factors present in *O. minuta* compared to two other sponges. Several ancestral classes are absent in this species. Numbers in circles indicate the number of genes found, question mark indicates the absence of data in the literature

Our present analysis of *Oopsacas* whole genome confirms that neither *wntless*, *wnt*, nor *dishevelled* genes are present (Fig. 5a). In addition, *frizzled (Fzd) A* gene is absent, whereas *frizzled B* is present. In contrast, core members of two other key developmental pathways, namely Notch and TGF-β (*transforming growth factor*-*beta*), are present (Fig. 5b; Additional file 3: Table S11). So far, the absence of the Wnt pathway was only reported in myxozoans [120], a group of microscopic parasitic cnidarians. But, in contrast to myxozoans, glass sponges do not show a highly reduced body plan compared to other sponges (Fig. 1c), and so the absence of Wnt challenges the pivotal role often attributed to this pathway in the acquisition of multicellularity and axial patterning [121].

In search of a pathway that may “compensate” for such an absence, we surveyed genes encoding heteromeric G proteins and G protein-coupled receptors (GPCRs), which are known to play important roles in transducing a broad range of extracellular signals. Heterodimeric G proteins include Gα (Gs, Gi, Gq, G12, and Gv classes [122]), Gβ, and Gγ subunits. Here, we found a diverse set of 8 Gα, one belonging to Gs, G12, and Gv classes, two to the Gi class, and three to the Gq class (Additional file 3: Table S12) [123]. We suppose that the early development in *Oopsacas* could use a truncated Wnt pathway (no Wnt activation) and that the expanded Gq set found in Porifera (and Ctenophora), compared to most bilaterians, placozoans and cnidarians [123–126], suggests a broader involvement of Gq proteins in the development of these animals. Because recent studies have suggested the ability of Fzd5 to activate Gq [127], the FzdB copy found in glass sponges (the Fzd5-ortholog) might not be a remnant of a reduced Wnt pathway but might instead reflect its involvement in G protein signaling.

### Transcription factors

Transcription factors (TFs) are also pivotal in animal body patterning. Here, we focused on basic helix loop helix (bHLH) and homeobox classes because (1) these TFs are significantly enriched in the animal TF repertoire [108] and (2) they have already been exhaustively surveyed in two other sponges thereby enabling comparisons [31].

There are six major groups (A, B, C, D, E, F) of bHLH TFs [128]. We identified 10 genes encoding proteins with a bHLH domain, two of which have an additional Per-Arnt-Sim (PAS) domain (Fig. 5c). Our phylogenetic analyses showed the presence of proteins in the AP4, MITF, SREBP, and E12/E47 families (class B bHLH) (Additional file 3: Table S13 and Fig. S8). In addition, one *Oopsacas* protein was found to cluster with several bHLH families constituting the Atonal-related superfamily [129–131]. The three remaining proteins are class C bHLH-PAS proteins: one of the Clock family, the two others clustering with ARNT (aryl hydrocarbon receptor nuclear translocator) and BMAL (brain and muscle Arnt-like) families [129] (Additional file 3: Fig. S9). Neither D, E, nor F class members were identified. There are clearly fewer bHLH homologs in *Oopsacas* than in *S. ciliatum* (30) and *A. queenslandica* (21). More than 10 families that are likely ancestral to sponges have been lost in this species (Fig. 5c).

Among the animal homeobox TFs classes, the Antennapedia (ANTP) and pair-ruled (PRD) are the largest [132–134]. In *Oopsacas*, we found 20 predicted proteins with a homeodomain (HD). According to both the identity of the best blast hits and domain analyses (Additional file 3: Table S14 and Fig. S10), only five sequences pertain to the ANTP class. Phylogenetic analyses show that there are members of the Msx (1), BarX (2), and NkX (2) families (Fig. 5d; Additional file 3: Fig. S11). Also present are six members of the super-class TALE, two members in the prd-like, LIM and Pou classes. We also identified one sequence assigned to the SIX (*sine oculis* homeobox) and the HNF (hepatocyte nuclear factor) classes (Fig. 5d; Additional file 3: Table S14). We found proteins containing a PAX domain, but none associating HD and PRD domains, suggesting that Prd class members are absent. We did not find any sequences from the ZF (zinc finger), Cut, Pros (Prospero), CerS (ceramid synthase), and MKX *(Mohawk* homeobox) classes.

Altogether, the number of ANTP TFs in *Oopsacas* (5) is lower than in other sponges (9–11) [31]. According to previous studies, some ANTP members were lost independently in sponge classes. The absence in *Oopsacas* of Hhex (haematopoietically expressed homeobox) and Prd genes, that are usually considered ancestral, is meaningful [31, 133, 135–138].

In summary, the TF complement of *Oopsacas* differs from that of other sponges studied so far and exhibits a markedly reduced number of the core metazoan TFs. Some authors have suggested that TF loss plays a major role in adaptation to environmental changes and in macroevolution [139]. However, the functional consequence of such TF losses on regulatory networks in glass sponges remains to be explored.

### Photokinesis and signal transduction

Like unicellular eukaryotes, sponges lack neurons but respond to stimuli [140–148]. Photokinesis is the best studied sensory mechanism involved in sponge larval behavior; however, all sponge genomes examined so far lack opsins, as is also the case here. Nevertheless, *O. minuta* possesses two photolyase genes, also found in the demosponge *A. queenslandica* (Additional file 3: Table S15). It has been shown previously that, in a glass sponge, photolyases are not only involved in DNA repair but have also been found to be expressed regionally, which was suggested to be a response to light even at a depth of 30 m [149]. No experiments have been carried out to determine whether glass sponge larvae carry out photokinesis or whether the function of the photolyase genes in *Oopsacas* are similar to those described in *Amphimedon* [150, 151].

Hexactinellids are unusual among Porifera in terms of signal transduction because they coordinate arrests of their feeding current using action potentials that travel through syncytial tissues [143, 152, 153]. Although glass sponge syncytia are quite different than neurons, as they do propagate electrical signals, we searched for genes typically involved in chemical signaling in bilaterians. We found no evidence for conventional monoamine signaling receptors or biosynthesis pathway components (serotonin or dopamine). In contrast, components for glutamate and GABA (gamma-aminobutyric acid) synthesis and signaling were identified. Nitric oxide signaling is likely present as well as acetylcholinesterase (Additional file 3: Table S16).

In terms of voltage-gated ion channels (Additional file 3: Table S17), we found genes for voltage-gated calcium channels (two pore channels, sperm-associated calcium channels, and voltage-gated hydrogen channels) (Additional file 3: Fig. S12) but no genes for voltage-gated sodium channels (Nav) nor for obvious voltage-gated potassium channels (Kv) (Additional file 3: Fig. S13). We did not find ENaC (epithelial sodium channels) nor LEAK channels. Ionotropic glutamate receptors (iGluR) were also absent. In contrast, four genes encoding for anoctamins (voltage sensitive calcium activated chloride channels) and six chloride channels (H + /Cl-transporters) were identified. *Oopsacas* also has cyclic-gated nucleotide (CGN/HCN) channels as well as purinergic and ryanodine receptors. In addition, there were many hits for transient receptor potential (TRP) family proteins (including one TRP-ML and one TRP-A family protein).

The study of glass sponge conduction system performed on *Rhabdocalyptus dawsoni* [153] suggested that the action potential could be driven by calcium because it was blocked by calcium channel blockers. This may be consistent with the diversity of calcium channels identified here. While the return to resting potential is sensitive to a potassium channel blocker, *Oopsacas* does not appear to possess the voltage sensor region of K channels, so it is unknown which channels are responsible for resetting the membrane potential.

Finally, we also searched for genes involved in synapses in animals with neurons. Postsynaptic proteins include a wide range of scaffolding and vesicle fusion/transport proteins, and many are present in *Oopsacas*, among them synaptobrevin, syntaxin, Homer, and members of the SNARE (SNAP receptor) family SNAP 25 (soluble N-ethylmaleimide-sensitive factor-attachment protein), with the exception of neurexin and neuroligin. In contrast, most of conventional presynaptic proteins such as profilin, synaphin, synaptoporin, and synaptogamin were missing (Additional file 3: Tables S18 and S19). In summary, a complete synaptic machinery is absent in *O. minuta*. Although proteins involved in vesicle transport and ion channels were identified, they may play a variety of other roles.

#### The biosilicification toolkit

**Fig. 6:**
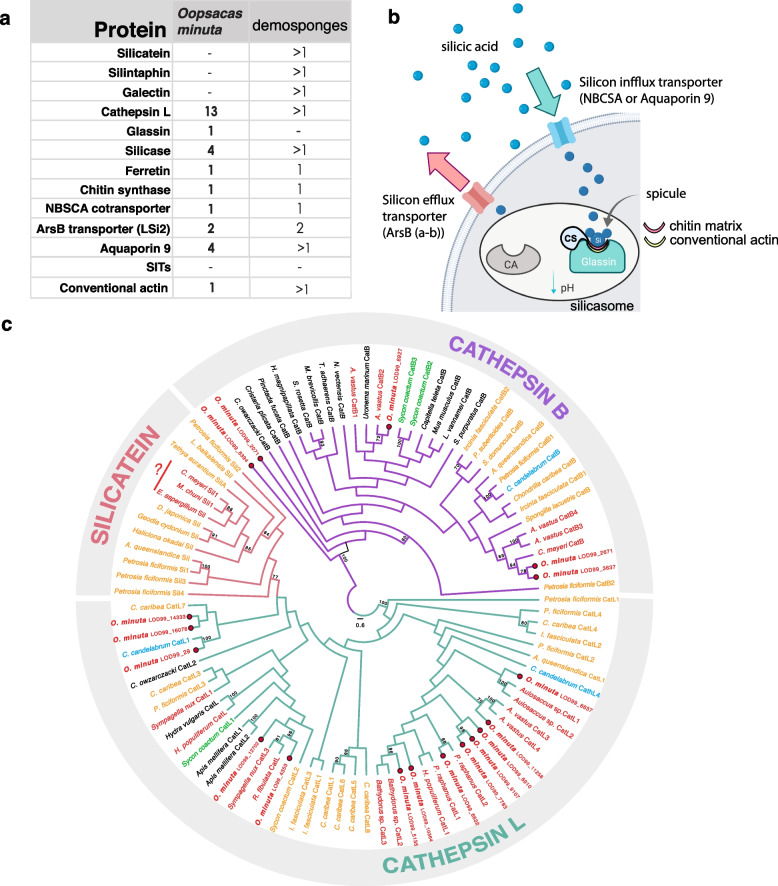
Biosilicification toolkit in *O. minuta*. **a** Summary of major biosilicification proteins involved in demosponge siliceous skeleton production. **b** Schematic of the biomineralization process in the sclerosyncytia of Hexactinellida. CA carbonic anhydrase, CS chitin synthase, NBSCA NBC (Na^+^/HCO_3_^−^ [Si(OH)_4_]) transporter, Si biosilica. **c** Phylogenetic hypothesis obtained by maximum likelihood of cathepsin evolution of the major sponge lineages. The sequences obtained from the genome of *O. minuta* are shown in blue. Bootstrap values less than 70 are not shown. The list of sequences used and their accession numbers are available in Additional file 3: Table S21, the alignment is provided at https://zenodo.org/communities/oops_13 [166]

The typical 6-rayed spicules of glass sponges*,* hexactines, are made of silica. Although spiculogenesis has similarities across siliceous sponges, there are fundamental differences between hexactinellids and demosponges [154–158]. We therefore searched the genome of *O. minuta* for homologs of genes known to be involved in spicule formation in demosponges (Fig. 6a, b; Additional file 3: Table S20). We found no evidence of *silicatein*, *silintaphin*, or *galectin* genes, which are common in demosponges, but we identified a single *glassin* gene, 13 *cathepsin L* genes (Fig. 6c; Additional file 3: Tables S20 and S21), 1 *ferretin*, 4 *silicases*, and 1 *chitin synthase*. In addition, we found a homolog of conventional *actin*, which has been recently reported to be important for spicule scaffolding [159]. Shimizu and collaborators [156] tried unsuccessfully to isolate silicatein proteins from the spicules of the glass sponge *Euplectella aspergillum* and failed to recover any sequence belonging to the silicatein family in this species. Similarly, we did not find any *silicatein* ortholog (except cathepsin L). Transcripts for silicatein were also not found in the hexactinellid *Aphrocallistes vastus*, nor in any transcriptome of hexactinellids published to date, suggesting silicatein is only present in demosponges [23, 160]. While some authors claimed to have purified silicateins directly from the skeleton of other hexactinellid species [161–165], these reports most likely reflects either contamination or quite divergent sequences in other glass sponges. In light of our results, the most plausible explanation is that hexactinellids and demosponges, as well as homoscleromorphs, use different enzymes to build their skeleton, which is also supported by the fact that Homoscleromorpha (the third class with siliceous spicules) lack both silicatein and glassin [160]. This hypothesis has profound implications since it suggests a convergent evolution of the ability to produce siliceous spicules. However, it is also possible that the numerous cathepsin L genes (the family to which silicatein belongs) present in glass sponges (and also homoscleromorphs) are involved in spiculogenesis; functional studies would have to be performed to test this hypothesis.

For silicon transport into the glass sponge sclerosyncytia [167, 168], the *O. minuta* genome encodes a NBCSA (Na^+^/HCO_3_^−^[Si(OH)_4_]) cotransporter, four *aquaporin 9* genes, and two ArsB (arsenite–antimonite) transporters. The genes encoding silicon transporters (SITs) used by other silicifying organisms [169] were not found in the *Oopsacas* genome. The exact mechanisms of interaction between the different enzymes are poorly known, but the identification of the largest complement of genes used for biosilicification in *Oopsacas* is definitely a fundamental step towards the full characterization of the process in Hexactinellida.

## Discussion

The *Oopsacas minuta* whole genome is the first reported for a glass sponge. It differs significantly from all other available sponge genomes in having a far smaller complement of predicted protein-coding genes. At only 16,413 genes, it is among the smallest of non-parasitic metazoan genomes reported so far [120, 170–172].

The most striking and unexpected feature of the *Oopsacas* genome is the absence of many genes that are typically considered ancestral and essential for metazoan morphogenetic processes (e.g., a functional Wnt pathway and numerous transcription factors). Because these genes are present in the three other sponge lineages, their absence in *Oopsacas* most likely indicates secondary losses given that current phylogenetic relationships place Hexactinellida as sister to Demospongiae (Fig. 1a).

These losses are not associated with a highly reduced body size and/or complexity or a parasitic lifestyle but instead may reflect the peculiar syncytial organization typical of this group. However, given the large differences documented between demosponge species [30], it would be premature to infer that these characteristics are shared by the nearly 700 other described hexactinellid species [13]. Both the ongoing sequencing of two other glass sponge species, *Aphrocallistes vastus* and the “tulip sponge” (Sally Leys and Darrin Schultz, personal communications) and the use of powerful phylogenetic-based annotation pipelines, such as the OMA (Orthologous matrix) database [173], should allow further valuable comparisons.

Interestingly, our work further documents the important role of convergence during animal evolution, by suggesting the independent emergence of the different mineral skeletons found in sponges, of septate-like junctions, of electrical signaling, and of multiciliated cells at an early stage of animal evolution.

We also report, for the first time, the association of a new *Thaumarchaeota* species (named *Candidatus Cenarchaeum massiliensis*) with a hexactinellid sponge. *Thaumarchaeota* species were only previously known to associate with Demospongiae [58]. An unusual enrichment of transposases in the *C. massiliensis* genome points to a high potential for lateral gene transfer [58].

## Conclusion

Sponges (Porifera) are ecologically important in aquatic ecosystems because of their high number of species (> 9500) and also for the habitat they provide for many other animals. They emerged and diversified over 600 Myrs ago into four distinct lineages, each having distinct features. Here we report on the first whole-genome analysis of a glass sponge (Hexactinellida) and provide new insights on sponge biology and evolution. Despite a filter feeding body plan similar to that of other sponges, *Oopsacas* has unusual genomic features and a very reduced gene content. More generally because sponges are part of one of the most ancient still extant animal lineages, the study of their genomes provides valuable insights into the evolution of animal body plans. Our findings reinforce the idea that gene losses, convergent evolution of similar body features, and tight relationships with microorganisms were important processes in the early diversification of animals.

## Methods

### Sample collections

Adult individuals (which brood embryos and pre-larvae all year long) were collected in the 3PP cave, La Ciotat (France, 43° 9.797′ N, 5° 36.000′ E). Because this population lives in a deep cave, it is considered to be isolated from other populations (from other caves and canyons), thereby the heterozygosity is expected to be low. Samples were brought back to the laboratory in a cooler, then immediately cut and cleaned of superficial sediment and organisms under a stereomicroscope with brush and forceps, and rinsed in 0.2 µm filtered sea water to reduce the chance of contamination. Samples were either used freshly or stored at − 80 °C for future use.

### Genomic DNA and RNA extraction and sequencing

Because of the low quantity of tissues present in an adult, in order to obtain sufficient material, one different adult was used for each RNA or DNA extraction mentioned below.

For Illumina sequencing, gDNA extractions were performed on 22 mg of sample using the QIAamp® DNA Mini kit from QIAGEN according to manufacturer instructions. DNA quality and quantity were checked by electrophoresis, NanoDrop, and Qubit fluorometer.

RNA extractions were performed (Qiagen kits) by the ProfileXpert IBISA platform. Sequencing was performed using Illumina technology with RNA-seq paired-ends, DNA-seq paired-ends, and Nextera Mate Pair protocols on a HiSeq2500 sequencer with a read length of 150 bp (ProfileXpert-LCMT, Lyon, France, http://profilexpert.fr).

For PacBio sequencing, tissues (1.34 g) were cut in pieces and stirred in calcium and magnesium-free sea water together with 20 mM EDTA (following the protocol described for *Oscarella lobularis* [174]). The suspension was passed through a 40-µm cell strainer to remove spicules and then concentrated by centrifugation (400 g -3 min). DNA extraction was performed on the cell suspension with the MasterPure™ Complete DNA kit (Tebu Bio) according to the manufacturer instructions. DNA quantity and quality were checked by gel electrophoresis, NanoDrop, and Qubit. Long-read sequencing was performed on a Pacific Biosciences platform (University of Lausanne, Swiss, https://wp.unil.ch/gtf/).

Unless explicitly otherwise noted, all bioinformatic analyses, programs, and software used the default options.

### Genome assembly

#### Raw assembly

Genome sequencing was first done using the Illumina platform as previously described [38]. In addition, complementary sequencing was carried out on a PacBio platform to give a total of 751,460 long reads. These reads were filtered based on their length and quality with Pacific Bioscience tools (SMRT Portal) then self-corrected with Canu [175]. All Illumina reads were mapped to the corrected PacBio reads with Bowtie 2.3.4.1 [176]. Selected Illumina reads and corrected PacBio reads were then assembled together with spades v3.9 using options careful and Kmers 21, 41, 61, 81, and 99 [177].

#### Scaffold selection

Scaffolds longer than 1 kb were retained and submitted to MetaGeneMark v3.26 [178] followed by a blastp v2.2.26 [179] strategy against NR (best hits with evalue < 10^−5^). The best hit taxonomy of each gene was used in a LCA-like method where the taxonomy of each scaffold was assigned if at least half of the annotated genes had the same annotation. Scaffolds were then attributed to 4 groups: Eukaryota, Bacteria, Archaea, and Not Annotated (also containing viruses).

#### Scaffold polishing

All Illumina reads selected by mapping on the corrected PacBio reads during the raw assembly process were also used for polishing. The Eukaryota group (936 scaffolds) and the Archaea group (50 scaffolds) were individually submitted to SSPACE v3.9 [171] with parameters -× 0 -z 0 -k 5 -g 0 -a 0.7 -n 15 -p 0. The resulting super-scaffolds were then submitted to pilon v1.21 [172] with default parameters and Gapfiller v1.10 [173] with parameters -m 30 -o 2 -r 0.7 -n 10 -d 50 -t 10 -g 0 -i 1 to obtain 11 Archaea and 365 Eukaryota polished super-scaffolds which were regarded as the two draft genomes.

### Assembly metrics and comparisons

The 11 Archaea scaffolds were submitted to Checkm v1.07 [53] with a completeness score of 99.03% without contamination.

To compare the genome quality of *O. minuta* to that of other sponges, the 16,413 genes of *O. minuta* CDS (coding sequences) were submitted to BUSCO v2/3 [64] through the gVolante server (https://gvolante.riken.jp/analysis.html) using the 303 Eukaryota core genes set. The absence of potential contamination by human DNA was checked by blast against NR database, yielding no hit with similarities higher than 64%.

### Gene prediction and annotation of the dominant Thaumarchaeota

We predicted 1990 Archaea genes using GeneMarkS v4.6b and the --prok option. Each sequence was submitted to multiple annotation strategies. A blastp (Basic Local Alignment Search Tool) against NR with an e-value < 10^−5^ and using the 10 best hits was used as the main information for functional annotation. A domain search was performed against Pfam 28.0, TIGRFAM 15.0, SMART 6.2, ProDom 2006.1, PANTHER 9.0, Prosite 20.113, Hamap 201502.04 using interproscan v5.14-53 [180]. A CD-search [181] was done against the conserved domain database at NCBI (National Center for Biotechnology Information). Potential (trans)membrane proteins were predicted using Phobius [182]. Specific repeat domains were assessed using HMM (Hidden Markov models) search from the hmmer suite v3.1b1 [183] on different hmm profiles (ankyrin repeat, BTB/POZ domain, CASC3/Barentsz eIF4AIII binding, collagen triple helix repeat, DUF3420, DUF3447, F-box, MORN repeat, pentapeptide repeat). All predicted proteins shorter than 100 amino acids without match to at least one of the above methods were discarded. These results were manually integrated to improve the functional annotation of the 1675 remaining Thaumarchaeota proteins. All 20 standard transfer RNAs except for tryptophan were predicted using tRNA-scan-SE v1.3.1 [184, 185]. Each 23S, 16S, and 5S ribosomal RNAs were predicted using barrnap v0.7 (https://github.com/tseemann/barrnap) for the archaeal kingdom.

### Thaumarchaeota orthology analysis

We created a specific Thaumarchaeota protein database by merging predicted proteins from the new Thaumarchaeota genome with those from nine Thaumarchaeota genomes previously published [54, 55, 57, 62, 186–190]. Proteins were assigned to OrthoMCL-DB groups or to clusters into new ortholog groups (specific to Thaumarchaeota) using the OrthoMCL algorithm and database [191].

### Repeat analysis and gene predictions in *Oopsacas minuta*

The 365 contigs were scanned and masked for repeats using RepeatModeler 2.0.4 and RepeatMasker 4.1.4 (http://www.repeatmasker.org) from the Dfam TE tool container (https://github.com/Dfam-consortium/TETools).

Four masked versions of the genome were obtained combining soft masking (-xsmall), hardmasking with or without low complexity regions masking (-nolow) [192].

Transcriptomic reads were mapped to all Eukaryota scaffolds using TopHat v2.0.12 [180] with the following options: -i 20 -I 5000 --b2-very-sensitive --library-type fr-unstranded --no-discordant. The genes were predicted using Braker v1.9 [181] and Augustus v3.2.3 [182] on the different versions of the genome, masked or not. An aberrant protein of around 48,000 amino acids was removed from the predictions before the submission to BUSCO v2/v3 [58] through the gVolante server (https://gvolante.riken.jp/analysis.html) using the 303 Eukaryota core genes set (OrthoDB v9). The results, summarized in Additional file 2: Table S7, show that for a comparable complete BUSCO score, the lowest duplication level was obtained with the predictions from the unmasked version of the genome.

The 17,059 predicted genes were then annotated and manually curated using different criteria. Genes shorter than 100 amino acids with a null transcriptomic coverage and alternative transcripts not supported by Augustus were removed. The 16,444 remaining genes were submitted to NCBI Tbl2asn and all discrepancies corrected (33 genes removed accordingly). Two genes manually detected during specific annotation were added, leading to a final dataset of 16,413 genes.

All proteins were annotated on the basis of the best hit using blastp against NR (evalue < 10^−5^). To infer Gene Ontology for each protein, a domain search was performed using InterProScan on the same databases as for Thaumarchaeota and the GO identifiers were used on WEGO (http://wego.genomics.org.cn/). Results on the first two levels are presented in the Additional file 1 for the 7737 genes that received a GO annotation (Additional file 1: Table S6 and Fig. S5) [193].

All 20 standard tRNAs as well as initiator methionyl-tRNA, selenocystein tRNA, and suppressor tRNA were predicted with at least one copy using tRNA-scan-SE for the eukaryotic model. 28S, 18S, 5.8S, and 5S ribosomal RNAs were identified using barrnap for the eukaryotes.

More detailed annotations were performed for a number of candidate genes (see “Result” subsections). For this purpose, blastP was used to search for specific proteins of interest in the predicted proteome of *O. minuta*. The returned hits with e-value < 10^−2^ were then checked by a reciprocal best-hit approach against NR database (NCBI). When needed, additional analyses were carried out: phylogenetic analyses and protein domain analyses were used for some sequences, and the specific methods used are indicated in the comments of the corresponding table or figure (Additional file 3). The list of query sequences and of best-hits obtained at the reciprocal best-hit step are provided in Additional file 3: Tables S7 through Table S18. Supporting phylogenetic analyses or domain prediction are provided in Additional file 3: Figs. S8 to S11 (for corresponding datasets see [166, 194–197]).

### Global genome comparisons

A systematic orthology clustering was performed using orthofinder v2.3.12 [183] on 6 representative sponge proteomes: *Oopsacas minuta* (16,413 protein sequences from the present study), *Oscarella pearsei* (29,220 protein sequences), *Sycon ciliatum* (50,731 protein sequences), *Tethya wilhelma* (37,633 protein sequences), *Amphimedon queenslandica* (23,542 protein sequences), and *Ephydatia muelleri* (39,329 protein sequences). Corresponding source datasets used for comparison are available at:

*Oscarella pearsei* (previously named *O. carmela)*: https://web.archive.org/web/20190531133238/http://www.compagen.org/datasets/OCAR_T-PEP_130911.zip

*Sycon ciliatum:* https://web.archive.org/web/20170108095659/http://compagen.org/datasets/SCIL_T-PEP_130802.zip

*Tethya wilhelma*: https://bitbucket.org/molpalmuc/tethya_wilhelma-genome/src/master/gene_sets/twilhelma_v01_augustus_prots.fasta.gz

*Amphimedon queenslandica*: https://ftp-ncbi-nlm-nih-gov.insb.bib.cnrs.fr/genomes/all/GCF/000/090/795/GCF_000090795.2_v1.1/GCF_000090795.2_v1.1_protein.faa.gz

*Ephydatia muelleri*: https://bitbucket.org/EphydatiaGenome/ephydatiagenome/downloads/Emu_v1_prots.fasta.gz

### Localization of the main Thaumarchaeota species associated to *O. minuta*

*O. minuta* adults were collected from the 3PP cave in December 2014 (*N* = 3) and in August 2015 (*N* = 2) and preserved in 4% paraformaldehyde (PFA). After dehydration using ethanol, samples were embeded in paraffin wax. To obtain 8-µm sections, samples were desilicified in 5% hydrofluoric acid *en-bloc* for 8 min at room temperature; the block was rinsed with distilled water before sectioning.

After dewaxing with Neoclear®, endogenenous peroxydase was inhibited by adding 0.3% hydrogen peroxide in the first of 2 ethanol washes for 15 min, then sections were rehydrated and permeabilized with proteinase K (2 µg ml^−1^) for 45 min. Sections were post-fixed with 4% PFA for 15 min and Card-FISH (catalyzed reporter deposition fluorescent in situ hybridization) was carried out following protocols from [198, 199] (detailed protocols provided upon request).

The most abundant thaumarchaeotal species was localized using a specifically designed probe: THAUMOOPS840: CATTAGTACCGCTTCAGACC- HRP (horseradish peroxidase).

Negative controls consisted of the absence of probe (with or without TSA, tyramide signal amplification) or the use of a random sequence not 100% matching any sequence of the metagenome: NONOOPS02: GGTTCCTTAGTCACGCAGAA-HRP. We observed that the sponge tissue had an endogenous peroxidase activity (green background) that was incompletely abolished by 0.3% H_2_O_2_. Unfortunately, higher concentrations of hydrogen peroxide damaged the tissues to a point preventing localization.

## Supplementary Information

**Additional file 1:** Characterization of the microbiome of *Oopsacas minuta*. **Table S1.** Main features and taxonomic assignment of *Oopsacas minuta’s* metagenome assembly. **Table S2.** Taxonomic assignment of non-Eukaryotic and non-Archaeal Taxa. **Figure S1.** Phylogenetic positions of the main microbial species associated with *O. minuta*. **Table S3.** Genome features of Candidatus Cenarchaeum massiliensis compared to other Thaumarchaea. **Table S4.** Annotation of the predicted protein-coding genes of Ca. C. massilliensis. **Figure S2.** Characterization of the genome of *Ca. C. massiliensis*. **Figure S3.** The cobalamin synthesis genes in Thaumarchaeota. **Figure S4.** Localization of *Ca. C. massiliensis* in the tissues of *O. minuta* by Card-FISH.

**Additional file 2:** Genomic characteristics of *Oopascas minuta*. **Table S5.** Genome features of the glass sponge *O. minuta*. **Table S6.** Respective number of predicted and annotated sequences in the genome of *O. minuta*. **Figure S5.** Gene ontology at the first two levels for the 7,737 genes that received GO annotations. **Figure S6.** a) Orthology clustering on predicted proteomes of six sponges. b) Gene Ontology at level3 for orthogroups absent in *O. minuta* and present in all other sponge lineages. **Table S7.** ORFING report, Comparison of protein sequence lengths and BUSCO scores between the different versions of the genome masked or unmasked. **Table S8.** Types and relative abundances of repeats in the genome of *O. minuta*.

**Additional file 3:** Survey of candidate genes for epithelia, multicilliogenesis, signaling pathways, transcription factors, neuro-sensory functions, biosilicification. **Table S9.** Blastp search of proteins involved in bilaterian epithelial functions. **Figure S7.** Domain prediction of integrins and of laminins. **Table S10.** Blastp search concerning proteins involved in bilaterian multiciliogenesis. **Table S11.** Blast Search of core genes involved in three main ancestral signalling pathways. **Table S12.** Blastp search results concerning G-proteins. **Table S13.** Blastp search and protein domain analyses concerning basic Helix Loop Helixtranscription factors. **Figure S8.** Phylogenetic relationships among bHLH Transcription factors. **Figure S9.** Phylogenetic position of *Oopsacas* bHLH-PAS Transcription factors. **Table S14.** Blastp search, domain and phylogenetic analyses performed on various transcription factor types. **Figure S10.** Domain Prediction of HD transcription factors. **Figure S11.** Phylogenetic positions of *Oopsacas* Homeobox Transcription factors of the ANTP class. **Table S15.** Survey of candidate genes for photoreception. **Table S16.** Blastp search for proteins involved in chemical signaling. **Table S17.** HHMER searches in *O. minuta* for voltage gated ion channels. **Figure S12.** Diversity and phylogenetic positions of *Oopsacas* calcium channels. **Figure S13.** Phylogenetic positions of *Oopsacas* potassium channels. **Table S18.** Blastp search of proteins involved in bilaterian synapses. **Table S19.** Results of HMMER searches for SNARES proteins. **Table S20.** Blast P search of proteins involved in silica biogenesis. **Table S21.** List of sequences used for phylogenetic analyses of silicatein and cathepsin.

## Data Availability

Most datasets supporting the conclusions of this article are included within the article and its additional file(s). Sequences used to build HHMER profiles or alignments (phylogenetic analyses) are publicly available at https://zenodo.org/communities/oops_13/ [166, 194–197]. Transcriptomes and genomes are available at GenBank under the BioProject accession PRJNA761294 [200]. The Whole Genome Shotgun project of *Oopsacas minuta* is available under the accession JAKMXF000000000 [201]. The Whole Genome Shotgun project of the Thaumarchaeota species Candidatus Cenarchaeum massiliensis is available under the accession JAJIZT000000000 [202]. The reads dataset are available at the Sequence Read Archive (https://identifiers.org/insdc.sra:SRR23032961, https://identifiers.org/insdc.sra:SRR23032960, https://identifiers.org/insdc.sra:SRR23032959, https://identifiers.org/insdc.sra:SRR23032958, https://identifiers.org/insdc.sra:SRR23032957, https://identifiers.org/insdc.sra:SRR23032956, https://identifiers.org/insdc.sra:SRR23032955, https://identifiers.org/insdc.sra:SRR23032954, https://identifiers.org/insdc.sra:SRR23032953, https://identifiers.org/insdc.sra:SRR23032952).

## Abbreviations

AJ: Adherens junctions
AMO: Ammonia monooxygenase
ANTP: Antennapedia
AOA: Ammonium-oxidizing archaea
ARNT: Aryl hydrocarbon receptor nuclear translocator
ArsB: Arsenite-antimonite (transporter)
bHLH: Basic helix loop helix
BLAST: Basic local alignment search tool
BM: Basement membrane
BMAL: Brain and muscle Arnt-like
bp: Base pair
BUSCO: Benchmarking Universal Single-Copy Orthologs
*Ca. C. massiliensis*: *Candidatus Cenarchaeum massiliensis*
Card-FISH: Catalyzed reporter deposition fluorescent in situ hybridization
CCC: Cadherin–catenin complexes
CDS: Coding sequences
CEP: Centrosome-associated protein
CerS: Ceramid synthase
CJ: Cell junction
Cont: Contactin
CGN: Cyclic-gated nucleotide
CCNO: Cyclin O
DNA: Deoxyribonucleic acid
ECM: Extracellular matrix
ENaC: Epithelial sodium channel
FA: Focal adhesion
FoxJ1: Forkhead box J1
Fzd: Frizzled
GABA: Gamma-aminobutyric acid
G + C content: Guanine + cytosine content
GJ: Gap junction
GMNC: Geminin coiled-coil domain containing
GO: Gene Ontology
GPCR: G protein-coupled receptor
HD: Homeodomain
HDs: Hemidesmosomes
Hhex: Hematopoietically expressed homeobox
HMM: Hidden Markov models
HNF: Hepatocyte nuclear factor
HRP: Horseradish peroxidase
iGluR: Ionotropic glutamate receptors
IgSF: Immunoglobulin superfamily
Kb: Kilo bases
Kv: Voltage-gated potassium channels
Lam: Laminins
LCAM: Last common ancestor of metazoans
Mb: Megabases
MCC: Multiciliated cell
Mcidas: Multiciliate differentiation and DNA synthesis associated cell
MKX: Mohawk homeobox
Myb: Myeloblastosis proto-oncogen
Myrs: Million years
NAV: Voltage gated sodium channels
NBCSA: (Na^+^/HCO_3_^−^[Si(OH)_4_]) co-transporter
NCBI: National Center for Biotechnology Information
NR: Non-redundant databases
NO: Nitric oxide
PacBio: Pacific Biosciences
PA: Per-Arnt-Sim domain
PRD: Pair-Ruled
PLK4: Polo-like kinase 4
PROS: Prospero
SAS6: Spindle assembly abnormal 6
SIT: Silicon transporters
SIX: *Sine oculis* homeobox
SJ: Septate junctions
SNAP: Soluble N-ethylmaleimide-sensitive factor-attachment protein
SNARE: SNAP receptor
STIL: SCL/TAL1 interrupting locus
TF: Transcription factors
TGF-β: Transforming growth factor-beta
TRP: Transient receptor potential
TSA: Tyramide signal amplification
ZF: Zinc finger
