# Supplementary material for: The compact genome of the sponge Oopsacas minuta (Hexactinellida) is lacking key metazoan core genes
Source: BMC Biol. 2023 Jun 19;21:139. doi: 10.1186/s12915-023-01619-w (PMC10280926; doi:10.1186/s12915-023-01619-w)
Supplement: Supplementary file 1 — Additional file 1: Characterization of the microbiome of Oopsacas minuta. Table S1. Main features and taxonomic assignment of Oopsacas minuta’s metagenome assembly. Table S2. Taxonomic assignment of non-Eukaryotic and non-Archaeal Taxa. Figure S1. Phylogenetic positions of the main microbial species associated with O. minuta. Table S3. Genome features of Candidatus Cenarchaeum massiliensis compared to other Thaumarchaea. Table S4. Annotation of the predicted protein-coding genes of Ca. C. massilliensis. Figure S2. Characterization of the genome of Ca. C. massiliensis. Figure S3. The cobalamin synthesis genes in Thaumarchaeota. Figure S4. Localization of Ca. C. massiliensis in the tissues of O. minuta by Card-FISH. [file 12915_2023_1619_MOESM1_ESM.pdf]

<u>Additional File 1 Index</u>

Table S1 : *Main features and taxonomic assignment of* Oopsacas minuta's *metagenome assembly*

Table S2 *: Taxonomic assignment of non-Eukaryotic and non-Archaeal Taxa*

Figure S1 : *Phylogenetic position of the main microbial species associated with* O. minuta

Table S3 *: Genome features of* Candidatus Cenarchaeum massiliensis *compared to other Thaumarchaea.*

Table S4 *: Annotation of the predicted protein-coding genes of* Ca. C. massilliensis

Figure S2 : *Characterization of the genome of* Ca. C. massiliensis

Figure S3 : *The cobalamin synthesis genes in Thaumarchaeota.*

Figure S4 : *Localization of* Ca. C. massiliensis *in the tissues of* O. minuta *by Card-FISH*

<u>Table S1:</u> Main features and taxonomic assignment of Oopsacas minuta's metagenome assembly.

Scaffolds (>1kb) were retained and submitted to MetaGeneMark (Zhu et al, 2010)
followed by a blastp search (Altschul et al, 1990) strategy against NR (best hits with evalue < 10-5). A LCA-like method computed over all annotated genes was applied on each scaffold:
the taxonomy assigned to each scaffold corresponds to that found for at least half of the genes composing the scaffold.
Scaffolds were then classified into 5 groups: Eukaryota, Bacteria, Archaea, virus and Not Assigned taxa.

	Taxa	Mapped Reads	% of total reads	Mean Coverage	Total length (bp)	% of total assembly	Contigs > 1kb	polished scaffolds	Polished scaffold mean coverage	Reference ID
**Eukaryota**	Porifera nuclear genome	111941409	77.95	177	61170773	95.91	936	365	186	JAKMXF010000000
	Mitochondria	89460	0.06	455	19518	0.03	1	1	455	NC_027419.1
**Archae**	mainlyThaumarcheota	26189162	18.24	1537	1658688	2.60	50	11	1206	JAJIZT010000000
	Gammaproteobacteria	3762639	2.62	657	555205	0.87	44			
	other-Proteobacteria	454494	0.32	759	54400	0.09	13			
	Cyanobacteria	79313	0.06	104	75563	0.12	17			
**Bacteria**	Firmicutes	33314	0.02	92	36020	0.06	10			
	other-bacteria	71121	0.05	127	55808	0.09	14			
	NA*-bacteria	344951	0.24	468	71964	0.11	9			
**Virus**		633133	0.44	778	81222	0.13	2			
**Ambigous-taxa**		2037432	1.42	1124	164832	0.26	33			
**NA*-Taxa**		3888087	2.71	241	1525204	2.39	630			
**TOTAL**		143598996		220**	63779161		1096			

All statistics were collected before final scaffolding and polishing and only for contigs > 1 kbp
the total number of raw reads is: 395 851 746 (197 925 873 pairs)
* NA= Not Assigned

Table S2: Taxonomic assignment of non-Eukaryotic and non-Archaeal Taxa

Scaffolds >1kb have been submitted to MetaGeneMark followed by a blastp search against NR (best hits with evalue < 10-5) A LCA-like method computed over all annotated genes was applied on each scaffold: the taxonomy assigned to each scaffold corresponds to that found for at least half of the genes composing the scaffold. Scaffolds were then classified into 5 groups: Eukaryota, Bacteria, Archaea, virus and Not Assigned taxa.

number of scaffolds					Taxonomic assignation			
1	Bacteria	Aquificae	Aquificae	Aquificales	Hydrogenothermaceae	Persephonella	Persephonella sp.	
1	Bacteria	Bacteroidetes	Flavobacteriia	Flavobacteriales	Flavobacteriaceae	Flavobacterium	Flavobacterium sp.	
1	Bacteria	Bacteroidetes	Cytophagia	Cytophagales	Amoebophilaceae	Candidatus Amoebophilus	Candidatus Amoebophilus asiaticus	
1	Bacteria	Bacteroidetes						
2	Bacteria	Candidatus Dadabacteria	Candidatus Dadabacteria bacterium	CSP1-2				
1	Bacteria	Candidatus Tectomicrobia	Candidatus Entotheonella	Candidatus Entotheonella sp.	TSY2			
1	Bacteria	Chloroflexi	Chloroflexia	Chloroflexales	Chloroflexineae	Oscillochloridaceae	Oscillochloris	Oscillochloris trichoides
13	Bacteria	Cyanobacteria	Oscillatoriophycideae	Chroococcales	Microcystaceae	Microcystis	Microcystis aeruginosa	
1	Bacteria	Cyanobacteria	Oscillatoriophycideae	Oscillatoriales	Oscillatoriaceae	Limnoraphis	Limnoraphis robusta	
1	Bacteria	Cyanobacteria	Oscillatoriophycideae	Chroococcales	Microcystaceae	Microcystis	Microcystis sp.	
1	Bacteria	Cyanobacteria	Nostocales	Nostocaceae	Anabaena	Anabaena sp.		
1	Bacteria	Cyanobacteria	cyanobacterium					
3	Bacteria	Firmicutes	Bacilli	Bacillales	Paenibacillaceae	Aneurinibacillus	Aneurinibacillus soli	
1	Bacteria	Firmicutes	Negativicutes	Selenomonadales	Selenomonadaceae	Selenomonas	Selenomonas ruminantium	
1	Bacteria	Firmicutes	Clostridia	Thermoanaerobacterales	Thermoanaerobacterales	Family III.Incertae Sedis	Caldicellulosiruptor	Caldicellulosiruptor kristjanssonii
1	Bacteria	Firmicutes	Clostridia	Halanaerobiales	Halobacteroidaceae	Halobacteroidaceae	bacterium	
1	Bacteria	Firmicutes	Bacilli	Lactobacillales	Enterococcaceae	Enterococcus	Enterococcus hirae	
1	Bacteria	Firmicutes	Bacilli	Bacillales	Bacillaceae	Bacillus	Bacillus humi	
1	Bacteria	Firmicutes	Bacilli	Bacillales	Bacillaceae	Bacillus	Bacillus fastidiosus	
1	Bacteria	Firmicutes	Bacilli	Bacillales				
2	Bacteria	Nitrospirae	Nitrospirae	bacterium				
1	Bacteria	Nitrospirae	Nitrospira	Nitrospirales	Nitrospiraceae	Candidatus Magnetoovum	Candidatus Magnetoovum chiemensis	
1	Bacteria	Parcubacteria	group bacterium GW2011_GWC1_45_13					
1	Bacteria	Parcubacteria	group bacterium GW2011_GWC1_38_22					
34	Bacteria	Proteobacteria	Gammaproteobacteria					
5	Bacteria	Proteobacteria						
2	Bacteria	Proteobacteria	Gammaproteobacteria	endosymbiont of Ridgeia piscesae				
1	Bacteria	Proteobacteria	Gammaproteobacteria	Vibrionales	Vibrionaceae	Vibrio	Vibrio crassostreae	
1	Bacteria	Proteobacteria	Gammaproteobacteria	Oceanospirillales	Oleiphilaceae	Oleiphilus	Oleiphilus sp.	HI0086
1	Bacteria	Proteobacteria	Gammaproteobacteria	Oceanospirillales	Oceanospirillaceae	Oleispira	Oleispira antarctica	
1	Bacteria	Proteobacteria	Gammaproteobacteria	Oceanospirillales	Oceanospirillaceae	Marinobacterium	Marinobacterium jannaschii	
1	Bacteria	Proteobacteria	Gammaproteobacteria	Cellvibrionales	Halieaceae	Haliea	Haliea salexigens	
1	Bacteria	Proteobacteria	Gammaproteobacteria	Cellvibrionales				
1	Bacteria	Proteobacteria	Gammaproteobacteria	Alteromonadales	Moritellaceae	Moritella	Moritella sp.	
1	Bacteria	Proteobacteria	Gammaproteobacteria	Alteromonadales	Alteromonadaceae	Marinobacter	Marinobacter lipolyticus	
1	Bacteria	Proteobacteria	delta/epsilon subdivisions	Epsilonproteobacteria	Sulfurovum	Sulfurovum sp.		
1	Bacteria	Proteobacteria	delta/epsilon subdivisions	Epsilonproteobacteria	Campylobacterales	Campylobacteraceae	Sulfurospirillum	Sulfurospirillum sp.
1	Bacteria	Proteobacteria	delta/epsilon subdivisions	Deltaproteobacteria	Desulfuromonadales	Desulfuromonadaceae	Pelobacter	Pelobacter carbinolicus
1	Bacteria	Proteobacteria	delta/epsilon subdivisions	Deltaproteobacteria	Desulfovibrionales	Desulfovibrionaceae	Desulfovibrio	Desulfovibrio longus
1	Bacteria	Proteobacteria	delta/epsilon subdivisions					
1	Bacteria	Proteobacteria	Betaproteobacteria	Candidatus Accumulibacter	Candidatus Accumulibacter sp.			
1	Bacteria	Proteobacteria	Alphaproteobacteria	Sphingomonadales	Sphingomonadaceae	Sphingobium	Sphingobium sp.	
1	Bacteria	Proteobacteria	Alphaproteobacteria					
1	Bacteria	Spirochaetes	Spirochaetia	Leptospiraceae	Leptospira	Leptospira kmetyi		
9	Bacteria							
1	Viruses	Circoviridae	Circovirus	Bat	circovirus			
1	Viruses							

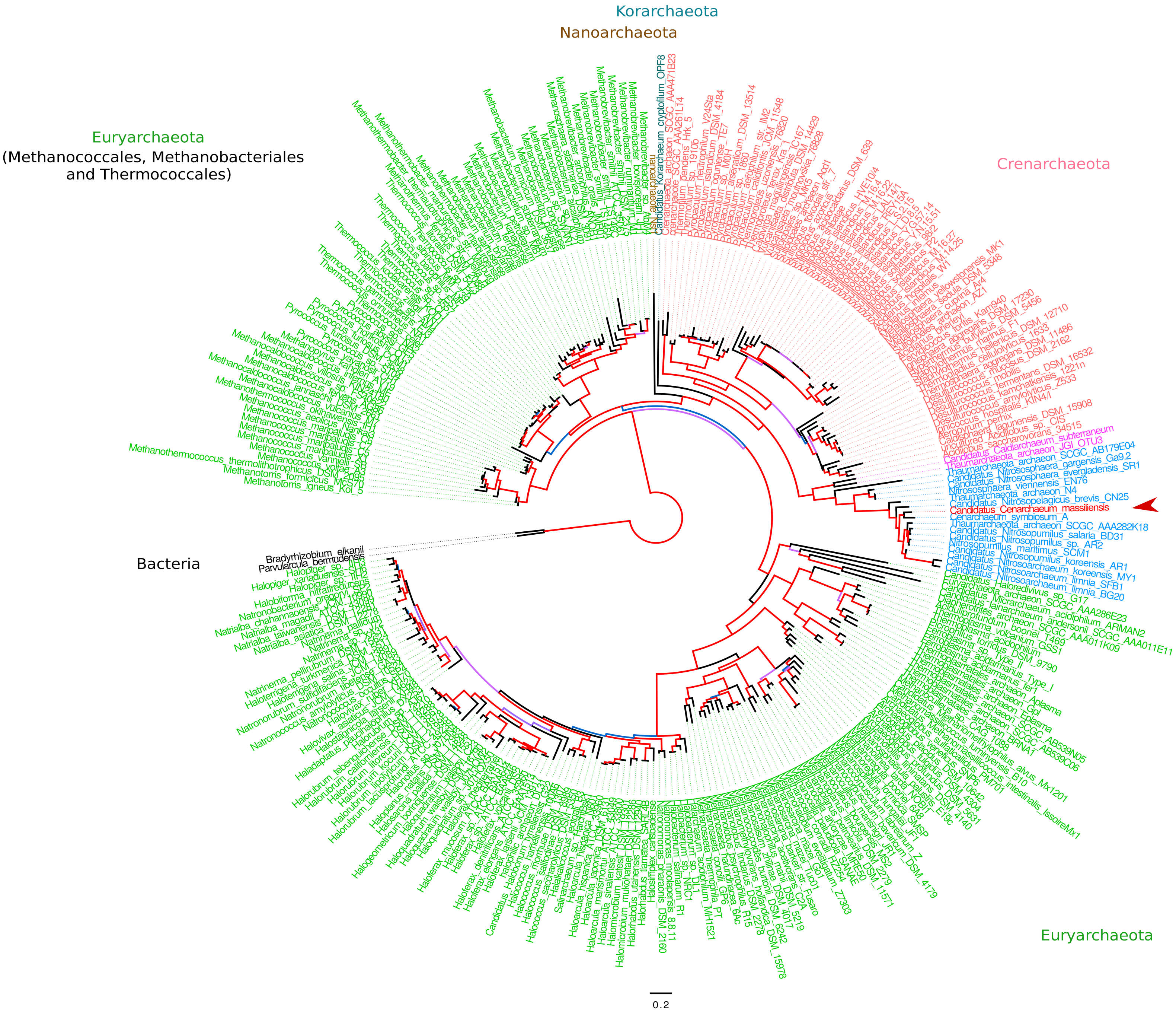

<u>Figure S1</u>: Phylogenetic position of the main microbial species (in red) associated to *O. minuta*
(infered by maximum-likelihood on ribosomal RNA genes, see material and method section for details). Main archaeal phyla are indicated and branch supports (approximate likelihood ratio test statistics with a Shimodaira–Hasegawa-like procedure) are indicated with a color code (>0.9, red; >0.7, purple; >0.5, blue and <0.5, black).
Scale bar represents the number of substitutions per site.

Table S3: **Genome features of *Candidatus Cenarchaeum massiliensis* compared to other Thaumarchaea.**

All statistics were obtained from the current version of the genome and the tRNA were predicted using tRNA-scan-SE (Chan & Lowe 2019).

Species	Size (Mb)	GC content	CDS content	Coding genes	tRNA number	Source
Cenarchaeum massiliensis	1.63	37.30%	**83.90%**	1675	48	
Cenarchaeum symbiosum	2.05	57.70%	91.40%	2017	45	http://bacteria.ensembl.org/cenarchaeum_symbiosum_a/Info/Index
Nitrosopumilus maritimus	1.65	34.20%	90.30%	1795	44	http://bacteria.ensembl.org/nitrosopumilus_maritimus_scm1/Info/Index
Nitrosopumilus koreensis	1.64	34.20%	87.20%	1890	41	http://bacteria.ensembl.org/candidatus_nitrosopumilus_koreensis_ar1/Info/Index
Nitrosoarchaeum koreensis	1.61	32.70%	89%	1945	42	http://bacteria.ensembl.org/candidatus_nitrosoarchaeum_koreensis_my1/Info/Index
Nitrosoarchaeum limnia	1.77	32.50%	83.40%	2038	45	http://bacteria.ensembl.org/candidatus_nitrosoarchaeum_limnia_sfb1/Info/Index
Nitrosopelagicus brevis	1.23	33.20%	94.60%	1445	42	https://www.ncbi.nlm.nih.gov/nuccore/CP007026.1
Nitrososphaera viennensis	2.53	52.70%	87%	3123	39	http://www.ncbi.nlm.nih.gov/genome/?term=Nitrososphaera+viennensis
Nitrososphaera gargensis	2.83	48.30%	81.60%	3565	34	https://www.ncbi.nlm.nih.gov/genome/?term=Candidatus+Nitrososphaera%20gargensis
Nitrososphaera evergladensis	2.95	50.10%	83.40%	3499	39	http://www.ncbi.nlm.nih.gov/nuccore/CP007174.1

Mb: megabase

CDS: coding sequence.

<u>Table S4:</u> Annotation of the predicted protein-coding genes of *Ca. C. massilliensis*

A specific Thaumarchaeota protein database was created merging these predicted proteins with predicted proteins from nine Thaumarchaeota genomes (Santoro et al., 2015; Hallam et al., 2006; Walker et al., 2010; Park et al., 2012; Blainey et al., 2011; Kim et al., 2011; Stieglmeier et al., 2014; Spang et al., 2012; Zhalnina et al., 2014). KEGG orthology was assessed using KAAS server (Moriya et al., 2007). Proteins were clustered using OrthoMCL algorithm and database (Fischer et al., 2011) . The assignment of gene ontology and enzyme codes was infered from homology sequence search on the nr database using Blast2GO tool (Conesa et al., 2005).

	protein code	protein size	annotation	KEGG O	protein domains	orthoMCL group	functionnal class	conserved in
Candidatus Cenarchaeum massiliensis	K8823_7	248	hypothetical protein		IPR004919	OG5_165184	Unknown function	Cenarchaeum
Candidatus Cenarchaeum massiliensis	K8823_887	72	Transposase		IPR004291	OG5_135857	Unknown function	Cenarchaeum
Candidatus Cenarchaeum massiliensis	K8823_900	194	Transposase			OG5_145215	Unknown function	Cenarchaeum
Candidatus Cenarchaeum massiliensis	K8823_935	332	Transposase		IPR027806	OG5_134022	Misc	Cenarchaeum
Candidatus Cenarchaeum massiliensis	K8823_936	119	Transposase		IPR004291	OG5_135857	Unknown function	Cenarchaeum
Candidatus Cenarchaeum massiliensis	K8823_938	277	Transposase		IPR025959	OG5_145215	Unknown function	Cenarchaeum
Candidatus Cenarchaeum massiliensis	K8823_941	630	hypothetical protein		IPR004919;IPR011089	OG5_165184	Unknown function	Cenarchaeum
Candidatus Cenarchaeum massiliensis	K8823_946	471	DNA sulfur modification protein DndC		IPR017598;IPR002500	OG5_205107	Sulfur metabolism	Cenarchaeum
Candidatus Cenarchaeum massiliensis	K8823_947	319	DNA sulfur modification protein DndB		IPR017642;IPR017642;IPR017601	sent|NP_455494	DNA modification	Cenarchaeum
Candidatus Cenarchaeum massiliensis	K8823_948	305	Transposase		IPR004291	OG5_135857	Unknown function	Cenarchaeum
Candidatus Cenarchaeum massiliensis	K8823_951	529	HNH endonuclease	K07454	IPR003615	bant|YP_020431	Unknown function	Cenarchaeum
Candidatus Cenarchaeum massiliensis	K8823_953	649	AAA domain-containing protein			OG5_178808	DNA modification	Cenarchaeum
Candidatus Cenarchaeum massiliensis	K8823_957	171	Transposase		IPR002525	OG5_191381	Unknown function	Cenarchaeum
Candidatus Cenarchaeum massiliensis	K8823_967	449	Transposase		IPR004291	OG5_135857	Unknown function	Cenarchaeum
Candidatus Cenarchaeum massiliensis	K8823_969	167	hypothetical protein			aful|NP_069985	Unknown function	Cenarchaeum
Candidatus Cenarchaeum massiliensis	K8823_973	222	Transposase		IPR002525	OG5_191381	Unknown function	Cenarchaeum
Candidatus Cenarchaeum massiliensis	K8823_980	338	HNH endonuclease		IPR003615;IPR025978	OG5_193878	Unknown function	Cenarchaeum
Candidatus Cenarchaeum massiliensis	K8823_985	439	Transposase		IPR004291	OG5_135857	Unknown function	Cenarchaeum
Candidatus Cenarchaeum massiliensis	K8823_986	471	HNH endonuclease		IPR003615;IPR025978	OG5_193878	Unknown function	Cenarchaeum
Candidatus Cenarchaeum massiliensis	K8823_989	151	Transposase		IPR004291	OG5_135857	Unknown function	Cenarchaeum
Candidatus Cenarchaeum massiliensis	K8823_990	178	Transposase		IPR004291	OG5_135857	Unknown function	Cenarchaeum
Candidatus Cenarchaeum massiliensis	K8823_994	293	ATPase AAA	K03798	IPR003593;IPR003959	OG5_147505	Unknown function	Cenarchaeum
Candidatus Cenarchaeum massiliensis	K8823_996	61	Transposase		IPR003346	OG5_191381	Unknown function	Cenarchaeum
Candidatus Cenarchaeum massiliensis	K8823_998	80	Transposase		IPR004291	OG5_135857	Unknown function	Cenarchaeum
Candidatus Cenarchaeum massiliensis	K8823_1001	2303	LVIVD repeat-containing protein		IPR006626;IPR013211	OG5_142178	Unknown function	Cenarchaeum
Candidatus Cenarchaeum massiliensis	K8823_1003	353	helicase			STG_0811	DNA modification	Cenarchaeum
Candidatus Cenarchaeum massiliensis	K8823_1016	148	Transposase		IPR004291	OG5_135857	Unknown function	Cenarchaeum
Candidatus Cenarchaeum massiliensis	K8823_1019	284	Transposase		IPR025959	OG5_145215	Unknown function	Cenarchaeum
Candidatus Cenarchaeum massiliensis	K8823_1023	3364	FG-GAP repeat domain protein	K06482	IPR013519;IPR013517;IPR013519;IPR013519;IPR013519;IPR013519	OG5_129708	Unknown function	Cenarchaeum
Candidatus Cenarchaeum massiliensis	K8823_1035	59	Transposase		IPR004291	OG5_135857	Unknown function	Cenarchaeum
Candidatus Cenarchaeum massiliensis	K8823_1037	112	Transposase		IPR004291	OG5_135857	Unknown function	Cenarchaeum
Candidatus Cenarchaeum massiliensis	K8823_1038	148	Transposase		IPR004291	OG5_135857	Unknown function	Cenarchaeum
Candidatus Cenarchaeum massiliensis	K8823_1078	294	Transposase		IPR002559	OG5_155537	Unknown function	Cenarchaeum
Candidatus Cenarchaeum massiliensis	K8823_100	69	DNA-binding protein	K07069	IPR018652	OG5_216874	Unknown function	Cenarchaeum
Candidatus Cenarchaeum massiliensis	K8823_1081	161	Transposase		IPR002525	OG5_191381	Unknown function	Cenarchaeum
Candidatus Cenarchaeum massiliensis	K8823_1091	229	Transposase		IPR004291	OG5_135857	Unknown function	Cenarchaeum
Candidatus Cenarchaeum massiliensis	K8823_10	58	hypothetical protein			STG_0142	Unknown function	Cenarchaeum
Candidatus Cenarchaeum massiliensis	K8823_1159	375	Transposase		IPR027806	OG5_134022	Misc	Cenarchaeum
Candidatus Cenarchaeum massiliensis	K8823_1170	151	Transposase		IPR004291	OG5_135857	Unknown function	Cenarchaeum
Candidatus Cenarchaeum massiliensis	K8823_1171	178	Transposase		IPR004291	OG5_135857	Unknown function	Cenarchaeum
Candidatus Cenarchaeum massiliensis	K8823_1176	178	Transposase		IPR004291	OG5_135857	Unknown function	Cenarchaeum
Candidatus Cenarchaeum massiliensis	K8823_1177	151	Transposase		IPR004291	OG5_135857	Unknown function	Cenarchaeum
Candidatus Cenarchaeum massiliensis	K8823_1181	128	Transposase		IPR004291	OG5_135857	Unknown function	Cenarchaeum
Candidatus Cenarchaeum massiliensis	K8823_1182	175	Transposase		IPR004291	OG5_135857	Unknown function	Cenarchaeum
Candidatus Cenarchaeum massiliensis	K8823_1191	151	Transposase		IPR004291	OG5_135857	Unknown function	Cenarchaeum
Candidatus Cenarchaeum massiliensis	K8823_1192	115	Transposase		IPR004291	OG5_135857	Unknown function	Cenarchaeum
Candidatus Cenarchaeum massiliensis	K8823_11	328	hypothetical protein			STG_0812	Unknown function	Cenarchaeum
Candidatus Cenarchaeum massiliensis	K8823_1236	160	Transposase		IPR002559	OG5_155537	Unknown function	Cenarchaeum
Candidatus Cenarchaeum massiliensis	K8823_1245	701	hypothetical protein		IPR004919;IPR011089	OG5_165184	Unknown function	Cenarchaeum
Candidatus Cenarchaeum massiliensis	K8823_1338	501	hypothetical membrane protein			cmaq|YP_001541022	Unknown function	Cenarchaeum
Candidatus Cenarchaeum massiliensis	K8823_1379	226	hypothetical protein		IPR019288	OG5_246936	Unknown function	Cenarchaeum
Candidatus Cenarchaeum massiliensis	K8823_1384	352	modification methylase	K07318	IPR012327;IPR002052	cjej|YP_002343666	Misc	Cenarchaeum
Candidatus Cenarchaeum massiliensis	K8823_13	318	hypothetical protein		IPR025248	STG_0815	Unknown function	Cenarchaeum
Candidatus Cenarchaeum massiliensis	K8823_1402	251	ABC transporter ATP-binding protein	K01995	IPR003593;IPR003439;IPR003439	OG5_130962	Energy	Cenarchaeum
Candidatus Cenarchaeum massiliensis	K8823_1403	240	ABC transporter ATP-binding protein	K01996	IPR003593;IPR003439;IPR017871;IPR003439	OG5_130857	Energy	Cenarchaeum
Candidatus Cenarchaeum massiliensis	K8823_1408	135	Transposase			OG5_135857	Unknown function	Cenarchaeum
Candidatus Cenarchaeum massiliensis	K8823_1409	82	Transposase		IPR004291	OG5_135857	Unknown function	Cenarchaeum
Candidatus Cenarchaeum massiliensis	K8823_1410	329	Transposase		IPR027806	OG5_134022	Misc	Cenarchaeum
Candidatus Cenarchaeum massiliensis	K8823_1412	3438	hypothetical protein		IPR006626;IPR013211	OG5_127159	Unknown function	Cenarchaeum
Candidatus Cenarchaeum massiliensis	K8823_1413	1450	restriction enzyme		IPR014001;IPR001650;IPR001650;IPR006935;IPR002052;IPR014001;IPR001650	OG5_163753	Stress & defense	Cenarchaeum
Candidatus Cenarchaeum massiliensis	K8823_1500	229	Transposase		IPR004291	OG5_135857	Unknown function	Cenarchaeum
Candidatus Cenarchaeum massiliensis	K8823_1527	199	hypothetical protein			OG5_156194	Unknown function	Cenarchaeum
Candidatus Cenarchaeum massiliensis	K8823_1562	274	hypothetical protein			STG_0816	Unknown function	Cenarchaeum
Candidatus Cenarchaeum massiliensis	K8823_1568	222	Transposase		IPR002525	OG5_191381	Unknown function	Cenarchaeum
Candidatus Cenarchaeum massiliensis	K8823_1572	325	Transposase		IPR027806	OG5_134022	Misc	Cenarchaeum
Candidatus Cenarchaeum massiliensis	K8823_1593	717	DNA sulfur modification protein DndD			OG5_178808	DNA modification	Cenarchaeum
Candidatus Cenarchaeum massiliensis	K8823_1594	325	DNA sulfur modification protein DndB		IPR017642;IPR017642;IPR017601	sent|NP_455494	DNA modification	Cenarchaeum
Candidatus Cenarchaeum massiliensis	K8823_1595	470	Phosphoadenosine phosphosulfate reductase		IPR002500;IPR017598	OG5_205107	Sulfur metabolism	Cenarchaeum
Candidatus Cenarchaeum massiliensis	K8823_1596	660	DNA sulfur modification protein DndD		IPR017599	OG5_178808	DNA modification	Cenarchaeum
Candidatus Cenarchaeum massiliensis	K8823_1599	55	hypothetical protein			STG_0142	Unknown function	Cenarchaeum
Candidatus Cenarchaeum massiliensis	K8823_159	930	hypothetical protein			STG_0817	Unknown function	Cenarchaeum
Candidatus Cenarchaeum massiliensis	K8823_1612	196	Transposase		IPR002525	OG5_191381	Unknown function	Cenarchaeum
Candidatus Cenarchaeum massiliensis	K8823_1621	196	Transposase		IPR004291	OG5_135857	Unknown function	Cenarchaeum
Candidatus Cenarchaeum massiliensis	K8823_1622	127	Transposase		IPR004291	OG5_135857	Unknown function	Cenarchaeum
Candidatus Cenarchaeum massiliensis	K8823_1625	219	Transposase		IPR002525	OG5_191381	Unknown function	Cenarchaeum
Candidatus Cenarchaeum massiliensis	K8823_1630	174	hypothetical protein			STG_0818	Unknown function	Cenarchaeum
Candidatus Cenarchaeum massiliensis	K8823_1653	198	Transposase			OG5_145215	Unknown function	Cenarchaeum
Candidatus Cenarchaeum massiliensis	K8823_1660	334	periplasmic serine protease		IPR002825	OG5_144428	Protein modification	Cenarchaeum
Candidatus Cenarchaeum massiliensis	K8823_1665	132	Transposase		IPR004291	OG5_135857	Unknown function	Cenarchaeum
Candidatus Cenarchaeum massiliensis	K8823_16	561	ATP-binding protein			STG_0820	Unknown function	Cenarchaeum
Candidatus Cenarchaeum massiliensis	K8823_172	711	hypothetical protein			STG_0821	Unknown function	Cenarchaeum
Candidatus Cenarchaeum massiliensis	K8823_17	566	hypothetical protein	K19789	IPR014001;IPR001650;IPR006935;IPR001650;IPR001650;IPR014001	OG5_131644	DNA modification	Cenarchaeum
Candidatus Cenarchaeum massiliensis	K8823_196	360	Transposase	K07486	IPR002525;IPR003346	OG5_191381	Unknown function	Cenarchaeum
Candidatus Cenarchaeum massiliensis	K8823_202	308	branched-chain amino acid transport permease	K01997	IPR001851	OG5_132026	Misc	Cenarchaeum
Candidatus Cenarchaeum massiliensis	K8823_203	345	branched-chain amino acid transport permease	K01998	IPR001851	OG5_132448	Transport	Cenarchaeum
Candidatus Cenarchaeum massiliensis	K8823_19	189	Transposase		IPR027806	OG5_134022	Misc	Cenarchaeum
Candidatus Cenarchaeum massiliensis	K8823_20	345	Transposase		IPR027806	OG5_134022	Misc	Cenarchaeum
Candidatus Cenarchaeum massiliensis	K8823_312	364	Transposase		IPR027806	OG5_134022	Misc	Cenarchaeum
Candidatus Cenarchaeum massiliensis	K8823_28	281	Radical SAM protein	K15045	IPR006638;IPR007197	OG5_136937	Unknown function	Cenarchaeum

Candidatus Cenarchaeum massiliensis	K8823_367	236	restriction endonuclease		IPR029127	OG5_250848	Unknown function	Cenarchaeum
Candidatus Cenarchaeum massiliensis	K8823_378	155	hypothetical protein		IPR020232	OG5_130679	Unknown function	Cenarchaeum
Candidatus Cenarchaeum massiliensis	K8823_379	497	hypothetical protein		IPR000594	OG5_129035	Unknown function	Cenarchaeum
Candidatus Cenarchaeum massiliensis	K8823_395	823	CRISPR-associated protein, Csd1 family		IPR010155;IPR021124;IPR013422;IPR010144;IPR010144	ctep|NP_662024	Stress & defense	Cenarchaeum
Candidatus Cenarchaeum massiliensis	K8823_417	239	hypothetical protein		IPR029127	OG5_250848	Unknown function	Cenarchaeum
Candidatus Cenarchaeum massiliensis	K8823_441	58	Transposase		IPR003346	OG5_191381	Unknown function	Cenarchaeum
Candidatus Cenarchaeum massiliensis	K8823_490	375	hypothetical protein		IPR002793	OG5_148029	Unknown function	Cenarchaeum
Candidatus Cenarchaeum massiliensis	K8823_537	248	hypothetical protein			STG_0823	Unknown function	Cenarchaeum
Candidatus Cenarchaeum massiliensis	K8823_547	293	NAD dependent epimerase/dehydratase family protein		IPR001509	OG5_139145	Oxidation-reduction	Cenarchaeum
Candidatus Cenarchaeum massiliensis	K8823_601	323	oxydoreductase	K00384	IPR023753	OG5_149638	Unknown function	Cenarchaeum
Candidatus Cenarchaeum massiliensis	K8823_633	128	Transposase IS66		IPR004291	OG5_135857	Unknown function	Cenarchaeum
Candidatus Cenarchaeum massiliensis	K8823_658	148	transposase		IPR004291	OG5_135857	Unknown function	Cenarchaeum
Candidatus Cenarchaeum massiliensis	K8823_696	144	Transposase		IPR004291	OG5_135857	Unknown function	Cenarchaeum
Candidatus Cenarchaeum massiliensis	K8823_699	153	Transposase		IPR004291	OG5_135857	Unknown function	Cenarchaeum
Candidatus Cenarchaeum massiliensis	K8823_5	299	hypothetical protein		IPR011089	OG5_165184	Unknown function	Cenarchaeum
Candidatus Cenarchaeum massiliensis	K8823_711	61	Sec61beta family protein		IPR016482;IPR023531	msmi|YP_001273936	Unknown function	Cenarchaeum
Candidatus Cenarchaeum massiliensis	K8823_715	267	hypothetical protein			cbot|YP_001787251.1	Unknown function	Cenarchaeum
Candidatus Cenarchaeum massiliensis	K8823_63	243	hypothetical protein		IPR024524	STG_0825	Unknown function	Cenarchaeum
Candidatus Cenarchaeum massiliensis	K8823_718	200	Transposase		IPR002525	OG5_191381	Unknown function	Cenarchaeum
Candidatus Cenarchaeum massiliensis	K8823_751	287	Transposase		IPR025959	OG5_145215	Unknown function	Cenarchaeum
Candidatus Cenarchaeum massiliensis	K8823_753	151	Transposase		IPR004291	OG5_135857	Unknown function	Cenarchaeum
Candidatus Cenarchaeum massiliensis	K8823_754	390	Tetratricopeptide repeat-containing protein		IPR019734;IPR001440;IPR019734;IPR019734;IPR019734;IPR013026;IPR019734;IPR019734	OG5_131333	Unknown function	Cenarchaeum
Candidatus Cenarchaeum massiliensis	K8823_757	186	Transposase		IPR002525	OG5_191381	Unknown function	Cenarchaeum
Candidatus Cenarchaeum massiliensis	K8823_760	55	Transposase		IPR004291	OG5_135857	Unknown function	Cenarchaeum
Candidatus Cenarchaeum massiliensis	K8823_761	128	Transposase		IPR004291	OG5_135857	Unknown function	Cenarchaeum
Candidatus Cenarchaeum massiliensis	K8823_768	55	hypothetical protein			STG_0142	Unknown function	Cenarchaeum
Candidatus Cenarchaeum massiliensis	K8823_769	359	DNA sulfur modification protein DndB		IPR017642;IPR017642;IPR017601	sent|NP_455494	DNA modification	Cenarchaeum
Candidatus Cenarchaeum massiliensis	K8823_770	468	sulfotransferase		IPR017598;IPR002500	OG5_205107	Sulfur metabolism	Cenarchaeum
Candidatus Cenarchaeum massiliensis	K8823_771	673	DNA sulfur modification protein DndD		IPR017599	OG5_178808	DNA modification	Cenarchaeum
Candidatus Cenarchaeum massiliensis	K8823_776	739	AAA domain containing protein			OG5_178808	DNA modification	Cenarchaeum
Candidatus Cenarchaeum massiliensis	K8823_777	137	hypothetical protein			hwal|YP_657837	Unknown function	Cenarchaeum
Candidatus Cenarchaeum massiliensis	K8823_779	461	HNH endonuclease		IPR003615;IPR025978	OG5_193878	Unknown function	Cenarchaeum
Candidatus Cenarchaeum massiliensis	K8823_784	159	transposase		IPR004291	OG5_135857	Unknown function	Cenarchaeum
Candidatus Cenarchaeum massiliensis	K8823_786	678	hypothetical protein		IPR004919;IPR011089	OG5_165184	Unknown function	Cenarchaeum
Candidatus Cenarchaeum massiliensis	K8823_788	349	Transposase		IPR004291	OG5_135857	Unknown function	Cenarchaeum
Candidatus Cenarchaeum massiliensis	K8823_789	200	Transposase		IPR002559	OG5_155537	Unknown function	Cenarchaeum
Candidatus Cenarchaeum massiliensis	K8823_792	200	Transposase		IPR002559	OG5_155537	Unknown function	Cenarchaeum
Candidatus Cenarchaeum massiliensis	K8823_793	70	Transposase		IPR004291	OG5_135857	Unknown function	Cenarchaeum
Candidatus Cenarchaeum massiliensis	K8823_6	375	Transposase		IPR027806	OG5_134022	Misc	Cenarchaeum
Candidatus Cenarchaeum massiliensis	K8823_795	337	HNH endonuclease		IPR003615;IPR025978	OG5_193878	Unknown function	Cenarchaeum
Candidatus Cenarchaeum massiliensis	K8823_802	114	hypothetical protein			STG_0826	Misc	Cenarchaeum
Candidatus Cenarchaeum massiliensis	K8823_811	809	AAA domain containing protein	K03546		OG5_126577	Unknown function	Cenarchaeum
Candidatus Cenarchaeum massiliensis	K8823_871	375	Transposase		IPR027806	OG5_134022	Misc	Cenarchaeum
Candidatus Cenarchaeum massiliensis	K8823_1244	52	metallochaperone-like domain-containing protein		IPR011017;IPR007029	STG_0813	Transport	Cenarchaeum Nitrosoarchaeum
Candidatus Cenarchaeum massiliensis	K8823_1263	2338	peptidase S8/S53	K06585	IPR013519;IPR013517;IPR000209;IPR023828;IPR013519;IPR013519;IPR013519;IPR013519;IPR013519	OG5_182868	Protein modification	Cenarchaeum Nitrosoarchaeum
Candidatus Cenarchaeum massiliensis	K8823_538	244	hypothetical protein			mjan|NP_248520	Unknown function	Cenarchaeum Nitrosoarchaeum
Candidatus Cenarchaeum massiliensis	K8823_618	44	hypothetical protein			STG_0824	Unknown function	Cenarchaeum Nitrosoarchaeum
Candidatus Cenarchaeum massiliensis	K8823_1119	52	hypothetical protein			STG_0144	Unknown function	Cenarchaeum Nitrosoarchaeum Nitrosopelagicus
Candidatus Cenarchaeum massiliensis	K8823_1134	534	hypothetical protein			STG_0133	Unknown function	Cenarchaeum Nitrosoarchaeum Nitrosopelagicus
Candidatus Cenarchaeum massiliensis	K8823_500	37	hypothetical protein		IPR007087	STG_0271	Unknown function	Cenarchaeum Nitrosoarchaeum Nitrosopelagicus
Candidatus Cenarchaeum massiliensis	K8823_78	172	cytidyltransferase	K00952	IPR004821;IPR004821;IPR006418	OG5_225053	Metabolism of cofactors and vitamins	Cenarchaeum Nitrosoarchaeum Nitrosopelagicus Nitrosopumilus
Candidatus Cenarchaeum massiliensis	K8823_913	142	hypothetical protein			nmar|YP_001582106	Stress & defense	Cenarchaeum Nitrosoarchaeum Nitrosopelagicus Nitrosopumilus
Candidatus Cenarchaeum massiliensis	K8823_929	249	hypothetical protein		IPR010297	OG5_191698	Unknown function	Cenarchaeum Nitrosoarchaeum Nitrosopelagicus Nitrosopumilus
Candidatus Cenarchaeum massiliensis	K8823_82	171	NADPH-dependent FMN reductase		IPR005025	OG5_152629	Oxidation-reduction	Cenarchaeum Nitrosoarchaeum Nitrosopelagicus Nitrosopumilus
Candidatus Cenarchaeum massiliensis	K8823_83	146	hypothetical membrane protein			nmar|YP_001583072	Unknown function	Cenarchaeum Nitrosoarchaeum Nitrosopelagicus Nitrosopumilus
Candidatus Cenarchaeum massiliensis	K8823_84	73	hypothetical protein			nmar|YP_001583073	Unknown function	Cenarchaeum Nitrosoarchaeum Nitrosopelagicus Nitrosopumilus
Candidatus Cenarchaeum massiliensis	K8823_952	710	type III restriction endonuclease res subunit		IPR014001;IPR001650;IPR006935;IPR001650;IPR014001	OG5_187305	DNA modification	Cenarchaeum Nitrosoarchaeum Nitrosopelagicus Nitrosopumilus
Candidatus Cenarchaeum massiliensis	K8823_961	131	hypothetical exported protein			nmar|YP_001582290	Unknown function	Cenarchaeum Nitrosoarchaeum Nitrosopelagicus Nitrosopumilus
Candidatus Cenarchaeum massiliensis	K8823_982	91	Mut7-C RNAse domain-containig protein		IPR002782	nmar|YP_001582093	Unknown function	Cenarchaeum Nitrosoarchaeum Nitrosopelagicus Nitrosopumilus
Candidatus Cenarchaeum massiliensis	K8823_1031	246	hypothetical membrane protein			OG5_180485	Unknown function	Cenarchaeum Nitrosoarchaeum Nitrosopelagicus Nitrosopumilus
Candidatus Cenarchaeum massiliensis	K8823_1033	455	Cupredoxin nitrite reductase	K00368	IPR011707	OG5_163551	Nitrogen metabolism	Cenarchaeum Nitrosoarchaeum Nitrosopelagicus Nitrosopumilus
Candidatus Cenarchaeum massiliensis	K8823_1075	79	hypothetical protein			nmar|YP_001582189	Unknown function	Cenarchaeum Nitrosoarchaeum Nitrosopelagicus Nitrosopumilus
Candidatus Cenarchaeum massiliensis	K8823_1095	115	hypothetical protein			nmar|YP_001582195	Unknown function	Cenarchaeum Nitrosoarchaeum Nitrosopelagicus Nitrosopumilus
Candidatus Cenarchaeum massiliensis	K8823_1098	129	hypothetical membrane protein			nmar|YP_001582179	Unknown function	Cenarchaeum Nitrosoarchaeum Nitrosopelagicus Nitrosopumilus
Candidatus Cenarchaeum massiliensis	K8823_1108	126	hypothetical protein			nmar|YP_001582168	Unknown function	Cenarchaeum Nitrosoarchaeum Nitrosopelagicus Nitrosopumilus
Candidatus Cenarchaeum massiliensis	K8823_1115	133	hypothetical protein			nmar|YP_001582086	Unknown function	Cenarchaeum Nitrosoarchaeum Nitrosopelagicus Nitrosopumilus
Candidatus Cenarchaeum massiliensis	K8823_1116	213	methyltransferase type 11			OG5_168301	Misc	Cenarchaeum Nitrosoarchaeum Nitrosopelagicus Nitrosopumilus
Candidatus Cenarchaeum massiliensis	K8823_1118	122	hypothetical protein			nmar|YP_001582185	Unknown function	Cenarchaeum Nitrosoarchaeum Nitrosopelagicus Nitrosopumilus
Candidatus Cenarchaeum massiliensis	K8823_1124	88	aspartyl/glutamyl-tRNA amidotransferase subunit C			nmar|YP_001582204	Translation	Cenarchaeum Nitrosoarchaeum Nitrosopelagicus Nitrosopumilus
Candidatus Cenarchaeum massiliensis	K8823_1126	82	hypothetical protein			STG_0086	Unknown function	Cenarchaeum Nitrosoarchaeum Nitrosopelagicus Nitrosopumilus
Candidatus Cenarchaeum massiliensis	K8823_1135	198	hypothetical membrane protein			OG5_241575	Unknown function	Cenarchaeum Nitrosoarchaeum Nitrosopelagicus Nitrosopumilus
Candidatus Cenarchaeum massiliensis	K8823_1144	70	hypothetical membrane protein			nmar|YP_001582739	Unknown function	Cenarchaeum Nitrosoarchaeum Nitrosopelagicus Nitrosopumilus
Candidatus Cenarchaeum massiliensis	K8823_1197	62	hypothetical protein			nmar|YP_001581934	Unknown function	Cenarchaeum Nitrosoarchaeum Nitrosopelagicus Nitrosopumilus
Candidatus Cenarchaeum massiliensis	K8823_1201	70	hypothetical protein			STG_0085	Unknown function	Cenarchaeum Nitrosoarchaeum Nitrosopelagicus Nitrosopumilus
Candidatus Cenarchaeum massiliensis	K8823_1232	132	hypothetical protein			nmar|YP_001582435	Unknown function	Cenarchaeum Nitrosoarchaeum Nitrosopelagicus Nitrosopumilus
Candidatus Cenarchaeum massiliensis	K8823_1241	95	DNA/RNA-binding protein AlbA		IPR002775	nmar|YP_001582267	DNA modification	Cenarchaeum Nitrosoarchaeum Nitrosopelagicus Nitrosopumilus
Candidatus Cenarchaeum massiliensis	K8823_1266	214	short-chain alcohol dehydrogenase	K00059	IPR002198	nmar|YP_001581507	Lipid metabolism	Cenarchaeum Nitrosoarchaeum Nitrosopelagicus Nitrosopumilus
Candidatus Cenarchaeum massiliensis	K8823_1267	79	hypothetical protein			nmar|YP_001581508	Unknown function	Cenarchaeum Nitrosoarchaeum Nitrosopelagicus Nitrosopumilus
Candidatus Cenarchaeum massiliensis	K8823_119	67	hypothetical protein			nmar|YP_001583118	Unknown function	Cenarchaeum Nitrosoarchaeum Nitrosopelagicus Nitrosopumilus
Candidatus Cenarchaeum massiliensis	K8823_123	137	hypothetical protein			nmar|YP_001582884	Unknown function	Cenarchaeum Nitrosoarchaeum Nitrosopelagicus Nitrosopumilus
Candidatus Cenarchaeum massiliensis	K8823_1320	98	hypothetical protein			nmar|YP_001581660	Unknown function	Cenarchaeum Nitrosoarchaeum Nitrosopelagicus Nitrosopumilus
Candidatus Cenarchaeum massiliensis	K8823_1321	190	hypothetical exported protein			nmar|YP_001581661	Unknown function	Cenarchaeum Nitrosoarchaeum Nitrosopelagicus Nitrosopumilus
Candidatus Cenarchaeum massiliensis	K8823_1334	337	glucose-6-phosphate isomerase	K15916	IPR019490;IPR001347	OG5_149643	Carbohydrate metabolism	Cenarchaeum Nitrosoarchaeum Nitrosopelagicus Nitrosopumilus
Candidatus Cenarchaeum massiliensis	K8823_1345	292	NAD-dependent epimerase/dehydratase	K00329	IPR001509	OG5_128491	Oxidation-reduction	Cenarchaeum Nitrosoarchaeum Nitrosopelagicus Nitrosopumilus
Candidatus Cenarchaeum massiliensis	K8823_1356	85	hypothetical protein			OG5_241557	Unknown function	Cenarchaeum Nitrosoarchaeum Nitrosopelagicus Nitrosopumilus
Candidatus Cenarchaeum massiliensis	K8823_1374	53	hypothetical protein			nmar|YP_001581450	Unknown function	Cenarchaeum Nitrosoarchaeum Nitrosopelagicus Nitrosopumilus
Candidatus Cenarchaeum massiliensis	K8823_1414	406	hypothetical protein			OG5_213901	Misc	Cenarchaeum Nitrosoarchaeum Nitrosopelagicus Nitrosopumilus
Candidatus Cenarchaeum massiliensis	K8823_1424	78	Transcription factor Pcc1 domain-containing protein		IPR015419	nmar|YP_001581769	Transcription	Cenarchaeum Nitrosoarchaeum Nitrosopelagicus Nitrosopumilus
Candidatus Cenarchaeum massiliensis	K8823_135	114	hypothetical membrane protein			nmar|YP_001581377	Unknown function	Cenarchaeum Nitrosoarchaeum Nitrosopelagicus Nitrosopumilus
Candidatus Cenarchaeum massiliensis	K8823_1434	131	hypothetical protein			OG5_241558	Unknown function	Cenarchaeum Nitrosoarchaeum Nitrosopelagicus Nitrosopumilus
Candidatus Cenarchaeum massiliensis	K8823_1438	96	hypothetical protein			nmar|YP_001582033	Unknown function	Cenarchaeum Nitrosoarchaeum Nitrosopelagicus Nitrosopumilus
Candidatus Cenarchaeum massiliensis	K8823_1444	141	DoxX family protein		IPR011637	OG5_139587	Unknown function	Cenarchaeum Nitrosoarchaeum Nitrosopelagicus Nitrosopumilus
Candidatus Cenarchaeum massiliensis	K8823_1453	486	Collagen triple helix repeat-containing protein	K19721	IPR008160	OG5_126592	Cell & development	Cenarchaeum Nitrosoarchaeum Nitrosopelagicus Nitrosopumilus
Candidatus Cenarchaeum massiliensis	K8823_138	64	hypothetical membrane protein			nmar|YP_001581374	Unknown function	Cenarchaeum Nitrosoarchaeum Nitrosopelagicus Nitrosopumilus
Candidatus Cenarchaeum massiliensis	K8823_139	177	transcriptional regulator			nmar|YP_001581369	Translation	Cenarchaeum Nitrosoarchaeum Nitrosopelagicus Nitrosopumilus
Candidatus Cenarchaeum massiliensis	K8823_1466	436	ergothioneine biosynthesis protein EgtB	K18912	IPR017806;IPR005532;IPR024775	OG5_130034	Protein modification	Cenarchaeum Nitrosoarchaeum Nitrosopelagicus Nitrosopumilus
Candidatus Cenarchaeum massiliensis	K8823_1467	339	Histidine-specific methyltransferase EgtD		IPR019257;IPR017804	OG5_130034	Protein modification	Cenarchaeum Nitrosoarchaeum Nitrosopelagicus Nitrosopumilus
Candidatus Cenarchaeum massiliensis	K8823_1469	164	hypothetical membrane protein			nmar|YP_001582519	Unknown function	Cenarchaeum Nitrosoarchaeum Nitrosopelagicus Nitrosopumilus

Candidatus Cenarchaeum massiliensisK8823_1474	91	hypothetical protein			OG5_241553	Unknown function	Cenarchaeum Nitrosoarchaeum Nitrosopelagicus Nitrosopumilus
Candidatus Cenarchaeum massiliensisK8823_1477	84	hypothetical protein			STG_0063	Unknown function	Cenarchaeum Nitrosoarchaeum Nitrosopelagicus Nitrosopumilus
Candidatus Cenarchaeum massiliensisK8823_1479	212	hypothetical membrane protein			nmar|YP_001582530	Unknown function	Cenarchaeum Nitrosoarchaeum Nitrosopelagicus Nitrosopumilus
Candidatus Cenarchaeum massiliensisK8823_1496	61	hypothetical protein			STG_0062	Unknown function	Cenarchaeum Nitrosoarchaeum Nitrosopelagicus Nitrosopumilus
Candidatus Cenarchaeum massiliensisK8823_146	145	hypothetical protein			nmar|YP_001581365	Unknown function	Cenarchaeum Nitrosoarchaeum Nitrosopelagicus Nitrosopumilus
Candidatus Cenarchaeum massiliensisK8823_147	66	hypothetical membrane protein			OG5_241554	Unknown function	Cenarchaeum Nitrosoarchaeum Nitrosopelagicus Nitrosopumilus
Candidatus Cenarchaeum massiliensisK8823_1591	734	type III restriction enzyme res subunit		IPR001650;IPR014001;IPR025202;IPR006935;IPR001650;IPR001650;IPR014001	OG5_187305	DNA modification	Cenarchaeum Nitrosoarchaeum Nitrosopelagicus Nitrosopumilus
Candidatus Cenarchaeum massiliensisK8823_1607	79	hypothetical protein			nmar|YP_001582189	Unknown function	Cenarchaeum Nitrosoarchaeum Nitrosopelagicus Nitrosopumilus
Candidatus Cenarchaeum massiliensisK8823_1634	633	Pyridine nucleotide-disulfide oxidoreductase	K03885	IPR023753;IPR007087;IPR007087	OG5_126960	Oxidation-reduction	Cenarchaeum Nitrosoarchaeum Nitrosopelagicus Nitrosopumilus
Candidatus Cenarchaeum massiliensisK8823_1635	74	hypothetical membrane protein			STG_0045	Unknown function	Cenarchaeum Nitrosoarchaeum Nitrosopelagicus Nitrosopumilus
Candidatus Cenarchaeum massiliensisK8823_1636	148	hypothetical protein		IPR018747	nmar|YP_001582125	Unknown function	Cenarchaeum Nitrosoarchaeum Nitrosopelagicus Nitrosopumilus
Candidatus Cenarchaeum massiliensisK8823_1637	163	hypothetical protein			nmar|YP_001582123	Unknown function	Cenarchaeum Nitrosoarchaeum Nitrosopelagicus Nitrosopumilus
Candidatus Cenarchaeum massiliensisK8823_1655	278	hypothetical protein			OG5_144028	Energy	Cenarchaeum Nitrosoarchaeum Nitrosopelagicus Nitrosopumilus
Candidatus Cenarchaeum massiliensisK8823_192	61	hypothetical protein			STG_0065	Unknown function	Cenarchaeum Nitrosoarchaeum Nitrosopelagicus Nitrosopumilus
Candidatus Cenarchaeum massiliensisK8823_211	398	ABC transporter permease	K09808	IPR003838;IPR025857	OG5_133112	Transport	Cenarchaeum Nitrosoarchaeum Nitrosopelagicus Nitrosopumilus
Candidatus Cenarchaeum massiliensisK8823_227	72	AN1-type Zinc finger protein		IPR000058;IPR000058;IPR000058	OG5_187359	Unknown function	Cenarchaeum Nitrosoarchaeum Nitrosopelagicus Nitrosopumilus
Candidatus Cenarchaeum massiliensisK8823_239	385	molecular chaperone DnaJ		IPR001623;IPR001623;IPR001623	OG5_237379	Stress & defense	Cenarchaeum Nitrosoarchaeum Nitrosopelagicus Nitrosopumilus
Candidatus Cenarchaeum massiliensisK8823_246	576	mechanosensitive ion channel protein MscS		IPR006685	OG5_194603	Transport	Cenarchaeum Nitrosoarchaeum Nitrosopelagicus Nitrosopumilus
Candidatus Cenarchaeum massiliensisK8823_247	453	lysine 2,3-aminomutase			OG5_134233	Misc	Cenarchaeum Nitrosoarchaeum Nitrosopelagicus Nitrosopumilus
Candidatus Cenarchaeum massiliensisK8823_347	153	hypothetical protein			OG5_166106	Misc	Cenarchaeum Nitrosoarchaeum Nitrosopelagicus Nitrosopumilus
Candidatus Cenarchaeum massiliensisK8823_428	85	hypothetical membrane protein			OG5_165586	Transcription	Cenarchaeum Nitrosoarchaeum Nitrosopelagicus Nitrosopumilus
Candidatus Cenarchaeum massiliensisK8823_463	210	hypothetical protein			nmar|YP_001582817	Unknown function	Cenarchaeum Nitrosoarchaeum Nitrosopelagicus Nitrosopumilus
Candidatus Cenarchaeum massiliensisK8823_39	266	Alcohol dehydrogenase	K00059	IPR002198;IPR020904	OG5_127561	Metabolism of cofactors and vitamins	Cenarchaeum Nitrosoarchaeum Nitrosopelagicus Nitrosopumilus
Candidatus Cenarchaeum massiliensisK8823_480	57	preprotein translocase subunit SecE			STG_0061	Transport	Cenarchaeum Nitrosoarchaeum Nitrosopelagicus Nitrosopumilus
Candidatus Cenarchaeum massiliensisK8823_486	654	peptidase S8/S53 subtilisin kexin sedolisin (vpr)	K14647	IPR003137;IPR000209;IPR015500;IPR023828;IPR023827	OG5_137388	Protein modification	Cenarchaeum Nitrosoarchaeum Nitrosopelagicus Nitrosopumilus
Candidatus Cenarchaeum massiliensisK8823_517	215	DNA repair helicase			OG5_187305	DNA modification	Cenarchaeum Nitrosoarchaeum Nitrosopelagicus Nitrosopumilus
Candidatus Cenarchaeum massiliensisK8823_524	117	TPR repeat-containing protein		IPR019734;IPR019734;IPR013026	OG5_229055	Unknown function	Cenarchaeum Nitrosoarchaeum Nitrosopelagicus Nitrosopumilus
Candidatus Cenarchaeum massiliensisK8823_527	105	hypothetical protein		IPR004378	nmar|YP_001581807	Oxidation-reduction	Cenarchaeum Nitrosoarchaeum Nitrosopelagicus Nitrosopumilus
Candidatus Cenarchaeum massiliensisK8823_531	1611	NHL repeat-containing protein	K12035	IPR001258;IPR003410;IPR018511;IPR018511;IPR013017;IPR013017;IPR013017;IPR013017;IPR013017;IPR013017;IPR003	OG5_131005	Stress & defense	Cenarchaeum Nitrosoarchaeum Nitrosopelagicus Nitrosopumilus
Candidatus Cenarchaeum massiliensisK8823_532	72	hypothetical protein			STG_0064	Unknown function	Cenarchaeum Nitrosoarchaeum Nitrosopelagicus Nitrosopumilus
Candidatus Cenarchaeum massiliensisK8823_533	57	hypothetical protein			nmar|YP_001582703	Unknown function	Cenarchaeum Nitrosoarchaeum Nitrosopelagicus Nitrosopumilus
Candidatus Cenarchaeum massiliensisK8823_535	395	hypothetical protein			nmar|YP_001582701	Unknown function	Cenarchaeum Nitrosoarchaeum Nitrosopelagicus Nitrosopumilus
Candidatus Cenarchaeum massiliensisK8823_541	61	hypothetical protein			nmar|YP_001582280	Unknown function	Cenarchaeum Nitrosoarchaeum Nitrosopelagicus Nitrosopumilus
Candidatus Cenarchaeum massiliensisK8823_553	129	Zinc finger C2H2-type domain-containing protein		IPR007087	nmar|YP_001581971	Misc	Cenarchaeum Nitrosoarchaeum Nitrosopelagicus Nitrosopumilus
Candidatus Cenarchaeum massiliensisK8823_556	578	superfamily II DNA/RNA helicase, SNF2 family	K14440	IPR014001;IPR001650;IPR000330;IPR001650;IPR014001;IPR001650	OG5_128295	DNA modification	Cenarchaeum Nitrosoarchaeum Nitrosopelagicus Nitrosopumilus
Candidatus Cenarchaeum massiliensisK8823_557	397	hypothetical protein			nmar|YP_001582057	Unknown function	Cenarchaeum Nitrosoarchaeum Nitrosopelagicus Nitrosopumilus
Candidatus Cenarchaeum massiliensisK8823_566	431	hypothetical protein			nmar|YP_001582069	Unknown function	Cenarchaeum Nitrosoarchaeum Nitrosopelagicus Nitrosopumilus
Candidatus Cenarchaeum massiliensisK8823_569	178	heat shock protein DnaJ domain-containing protein	K03686	IPR001623;IPR001623;IPR001623	OG5_144570	Stress & defense	Cenarchaeum Nitrosoarchaeum Nitrosopelagicus Nitrosopumilus
Candidatus Cenarchaeum massiliensisK8823_570	64	TPR repeat-containing protein		IPR019734;IPR013026;IPR019734;IPR019734	OG5_146094	Unknown function	Cenarchaeum Nitrosoarchaeum Nitrosopelagicus Nitrosopumilus
Candidatus Cenarchaeum massiliensisK8823_571	167	TPR repeat-containing protein		IPR019734;IPR019734;IPR019734;IPR019734;IPR013026;IPR019734	OG5_168615	Unknown function	Cenarchaeum Nitrosoarchaeum Nitrosopelagicus Nitrosopumilus
Candidatus Cenarchaeum massiliensisK8823_579	91	hypothetical protein			nmar|YP_001582093	Unknown function	Cenarchaeum Nitrosoarchaeum Nitrosopelagicus Nitrosopumilus
Candidatus Cenarchaeum massiliensisK8823_587	63	Zinc finger protein		IPR007087	nmar|YP_001582361	Unknown function	Cenarchaeum Nitrosoarchaeum Nitrosopelagicus Nitrosopumilus
Candidatus Cenarchaeum massiliensisK8823_592	217	hypothetical protein			nmar|YP_001582354	Unknown function	Cenarchaeum Nitrosoarchaeum Nitrosopelagicus Nitrosopumilus
Candidatus Cenarchaeum massiliensisK8823_621	111	hypothetical protein			nmar|YP_001582386	Unknown function	Cenarchaeum Nitrosoarchaeum Nitrosopelagicus Nitrosopumilus
Candidatus Cenarchaeum massiliensisK8823_629	357	hypothetical protein			nmar|YP_001582513	Unknown function	Cenarchaeum Nitrosoarchaeum Nitrosopelagicus Nitrosopumilus
Candidatus Cenarchaeum massiliensisK8823_634	120	hypothetical protein			nmar|YP_001581966	Unknown function	Cenarchaeum Nitrosoarchaeum Nitrosopelagicus Nitrosopumilus
Candidatus Cenarchaeum massiliensisK8823_648	125	hypothetical protein			nmar|YP_001582566	Unknown function	Cenarchaeum Nitrosoarchaeum Nitrosopelagicus Nitrosopumilus
Candidatus Cenarchaeum massiliensisK8823_680	690	ATP-dependent endonuclease			OG5_181724	DNA modification	Cenarchaeum Nitrosoarchaeum Nitrosopelagicus Nitrosopumilus
Candidatus Cenarchaeum massiliensisK8823_681	145	hypothetical exported protein			nmar|YP_001582731	Unknown function	Cenarchaeum Nitrosoarchaeum Nitrosopelagicus Nitrosopumilus
Candidatus Cenarchaeum massiliensisK8823_689	275	dihydroorotate dehydrogenase	K17828	IPR005720;IPR005720;IPR001295;IPR024920	OG5_129390	Nucleotide metabolism	Cenarchaeum Nitrosoarchaeum Nitrosopelagicus Nitrosopumilus
Candidatus Cenarchaeum massiliensisK8823_690	269	dihydroorotate dehydrogenase electron transfer subunit	K02823	IPR019480;IPR001433;IPR017927	OG5_133098	Nucleotide metabolism	Cenarchaeum Nitrosoarchaeum Nitrosopelagicus Nitrosopumilus
Candidatus Cenarchaeum massiliensisK8823_62	313	nitrogen fixation protein NifR	K05540	IPR004652;IPR001269;IPR001269	OG5_127749	Translation	Cenarchaeum Nitrosoarchaeum Nitrosopelagicus Nitrosopumilus
Candidatus Cenarchaeum massiliensisK8823_722	324	abortive infection protein		IPR003675	nmar|YP_001581957	Stress & defense	Cenarchaeum Nitrosoarchaeum Nitrosopelagicus Nitrosopumilus
Candidatus Cenarchaeum massiliensisK8823_747	269	hypothetical protein			OG5_159684	Misc	Cenarchaeum Nitrosoarchaeum Nitrosopelagicus Nitrosopumilus
Candidatus Cenarchaeum massiliensisK8823_774	620	abortive infection protein		IPR018891	OG5_179408	Stress & defense	Cenarchaeum Nitrosoarchaeum Nitrosopelagicus Nitrosopumilus
Candidatus Cenarchaeum massiliensisK8823_778	743	type III restriction enzyme res subunit		IPR001650;IPR014001;IPR001650;IPR025202;IPR006935;IPR001650;IPR014001	OG5_187305	DNA modification	Cenarchaeum Nitrosoarchaeum Nitrosopelagicus Nitrosopumilus
Candidatus Cenarchaeum massiliensisK8823_781	91	Mut7-C RNAse domain-containing protein		IPR002782	nmar|YP_001582093	Unknown function	Cenarchaeum Nitrosoarchaeum Nitrosopelagicus Nitrosopumilus
Candidatus Cenarchaeum massiliensisK8823_798	65	hypothetical membrane protein			STG_0059	Unknown function	Cenarchaeum Nitrosoarchaeum Nitrosopelagicus Nitrosopumilus
Candidatus Cenarchaeum massiliensisK8823_807	116	hypothetical protein			nmar|YP_001581919	Unknown function	Cenarchaeum Nitrosoarchaeum Nitrosopelagicus Nitrosopumilus
Candidatus Cenarchaeum massiliensisK8823_843	154	PEBP family protein	K06910	IPR005247;IPR008914	OG5_130855	Misc	Cenarchaeum Nitrosoarchaeum Nitrosopelagicus Nitrosopumilus
Candidatus Cenarchaeum massiliensisK8823_863	212	isopentenyl-diphosphate delta-isomerase	K01823	IPR000086;IPR011876;IPR000086	OG5_128735	Lipid metabolism	Cenarchaeum Nitrosoarchaeum Nitrosopelagicus Nitrosopumilus
Candidatus Cenarchaeum massiliensisK8823_867	294	ROK family protein	K00847	IPR000600;IPR000600	OG5_129253	Carbohydrate metabolism	Cenarchaeum Nitrosoarchaeum Nitrosopelagicus Nitrosopumilus
Candidatus Cenarchaeum massiliensisK8823_869	594	adenine deaminase	K01486	IPR006680;IPR026912;IPR006679	OG5_138532	Nucleotide metabolism	Cenarchaeum Nitrosoarchaeum Nitrosopelagicus Nitrosopumilus
Candidatus Cenarchaeum massiliensisK8823_879	182	hypothetical membrane protein			nmar|YP_001582512	Unknown function	Cenarchaeum Nitrosoarchaeum Nitrosopelagicus Nitrosopumilus
Candidatus Cenarchaeum massiliensisK8823_890	367	luciferase family protein	K14728	IPR011251	OG5_140202	Oxidation-reduction	Cenarchaeum Nitrosoarchaeum Nitrosopelagicus Nitrosopumilus Nitrososphaera
Candidatus Cenarchaeum massiliensisK8823_891	931	Lamin tail domain-containing protein		IPR001322	OG5_204911	Misc	Cenarchaeum Nitrosoarchaeum Nitrosopelagicus Nitrosopumilus Nitrososphaera
Candidatus Cenarchaeum massiliensisK8823_892	81	hypothetical protein			nmar|YP_001582376	Unknown function	Cenarchaeum Nitrosoarchaeum Nitrosopelagicus Nitrosopumilus Nitrososphaera
Candidatus Cenarchaeum massiliensisK8823_893	124	hypothetical protein			nmar|YP_001582375	Unknown function	Cenarchaeum Nitrosoarchaeum Nitrosopelagicus Nitrosopumilus Nitrososphaera
Candidatus Cenarchaeum massiliensisK8823_894	181	tRNA intron endonuclease	K01170	IPR006677;IPR006678;IPR006676;IPR006676	OG5_128736	Translation	Cenarchaeum Nitrosoarchaeum Nitrosopelagicus Nitrosopumilus Nitrososphaera
Candidatus Cenarchaeum massiliensisK8823_895	217	phosphate transport regulator		IPR018445	OG5_166099	Misc	Cenarchaeum Nitrosoarchaeum Nitrosopelagicus Nitrosopumilus Nitrososphaera
Candidatus Cenarchaeum massiliensisK8823_896	583	ATP-dependent DNA ligase	K10747	IPR012309;IPR012308;IPR012310;IPR000977;IPR016059;IPR012310;IPR022865	OG5_127375	DNA modification	Cenarchaeum Nitrosoarchaeum Nitrosopelagicus Nitrosopumilus Nitrososphaera
Candidatus Cenarchaeum massiliensisK8823_897	135	hypothetical membrane protein			OG5_241570	Unknown function	Cenarchaeum Nitrosoarchaeum Nitrosopelagicus Nitrosopumilus Nitrososphaera
Candidatus Cenarchaeum massiliensisK8823_898	378	Fructose-1,6-bisphosphatase	K01622	IPR002803;IPR002803	OG5_139273	Oxidation-reduction	Cenarchaeum Nitrosoarchaeum Nitrosopelagicus Nitrosopumilus Nitrososphaera
Candidatus Cenarchaeum massiliensisK8823_79	164	copper export protein		IPR008457	OG5_187186	Transport	Cenarchaeum Nitrosoarchaeum Nitrosopelagicus Nitrosopumilus Nitrososphaera
Candidatus Cenarchaeum massiliensisK8823_905	440	Elongation factor EF-1, subunit alpha	K03231	IPR004160;IPR004161;IPR004539;IPR031157;IPR000795;IPR004539	OG5_126631	Translation	Cenarchaeum Nitrosoarchaeum Nitrosopelagicus Nitrosopumilus Nitrososphaera
Candidatus Cenarchaeum massiliensisK8823_906	102	ribosomal protein S10	K02946	IPR027486;IPR005729;IPR001848;IPR001848	OG5_126760	Translation	Cenarchaeum Nitrosoarchaeum Nitrosopelagicus Nitrosopumilus Nitrososphaera
Candidatus Cenarchaeum massiliensisK8823_907	74	DNA-directed RNA polymerase subunit P (rpoP)		IPR006591	OG5_161356	Transcription	Cenarchaeum Nitrosoarchaeum Nitrosopelagicus Nitrosopumilus Nitrososphaera
Candidatus Cenarchaeum massiliensisK8823_908	136	hypothetical protein			nmar|YP_001582365	Unknown function	Cenarchaeum Nitrosoarchaeum Nitrosopelagicus Nitrosopumilus Nitrososphaera
Candidatus Cenarchaeum massiliensisK8823_909	254	DNA methylase	K00571	IPR002941	OG5_135744	DNA modification	Cenarchaeum Nitrosoarchaeum Nitrosopelagicus Nitrosopumilus Nitrososphaera
Candidatus Cenarchaeum massiliensisK8823_911	268	methylase N-4/N-6 domain-containing protein	K00571	IPR002941;IPR002052	OG5_135744	DNA modification	Cenarchaeum Nitrosoarchaeum Nitrosopelagicus Nitrosopumilus Nitrososphaera
Candidatus Cenarchaeum massiliensisK8823_80	159	hypothetical membrane protein		IPR004864	OG5_241590	Unknown function	Cenarchaeum Nitrosoarchaeum Nitrosopelagicus Nitrosopumilus Nitrososphaera
Candidatus Cenarchaeum massiliensisK8823_912	185	decarboxylase	K03186	IPR003382;IPR004507	OG5_131043	Misc	Cenarchaeum Nitrosoarchaeum Nitrosopelagicus Nitrosopumilus Nitrososphaera
Candidatus Cenarchaeum massiliensisK8823_914	66	hypothetical membrane protein		IPR003369	OG5_129693	Transport	Cenarchaeum Nitrosoarchaeum Nitrosopelagicus Nitrosopumilus Nitrososphaera
Candidatus Cenarchaeum massiliensisK8823_915	234	preprotein translocase subunit TatC	K03118	IPR002033;IPR002033;IPR002033;IPR002033	OG5_130464	Transport	Cenarchaeum Nitrosoarchaeum Nitrosopelagicus Nitrosopumilus Nitrososphaera
Candidatus Cenarchaeum massiliensisK8823_916	167	hypothetical protein			nmar|YP_001582109	Unknown function	Cenarchaeum Nitrosoarchaeum Nitrosopelagicus Nitrosopumilus Nitrososphaera
Candidatus Cenarchaeum massiliensisK8823_917	86	Phosphoribosylformylglycinamidine synthase		IPR003850;IPR003850;IPR003850	OG5_135086	Misc	Cenarchaeum Nitrosoarchaeum Nitrosopelagicus Nitrosopumilus Nitrososphaera
Candidatus Cenarchaeum massiliensisK8823_918	224	Phosphoribosylformylglycinamidine synthase 1	K01952	IPR010075;IPR017926;IPR010075	OG5_133669	Nucleotide metabolism	Cenarchaeum Nitrosoarchaeum Nitrosopelagicus Nitrosopumilus Nitrososphaera
Candidatus Cenarchaeum massiliensisK8823_919	701	phosphoribosylformylglycinamidine synthase II	K01952	IPR016188;IPR010074;IPR010918;IPR010074	OG5_127725	Nucleotide metabolism	Cenarchaeum Nitrosoarchaeum Nitrosopelagicus Nitrosopumilus Nitrososphaera
Candidatus Cenarchaeum massiliensisK8823_920	483	glutamine amidotransferase class II	K00764	IPR000836;IPR005854;IPR017932	OG5_127706	Nucleotide metabolism	Cenarchaeum Nitrosoarchaeum Nitrosopelagicus Nitrosopumilus Nitrososphaera
Candidatus Cenarchaeum massiliensisK8823_81	217	cob(II)yrinic acid a,c-diamide reductase (bluB)	K04719	IPR029479;IPR012825	OG5_146497	Metabolism of cofactors and vitamins	Cenarchaeum Nitrosoarchaeum Nitrosopelagicus Nitrosopumilus Nitrososphaera
Candidatus Cenarchaeum massiliensisK8823_922	322	bifunctional biotin-- synthetase/biotin operon repressor	K03524	IPR004408;IPR013196;IPR004143;IPR004408;IPR030855;IPR004143	OG5_126858	Metabolism of cofactors and vitamins	Cenarchaeum Nitrosoarchaeum Nitrosopelagicus Nitrosopumilus Nitrososphaera
Candidatus Cenarchaeum massiliensisK8823_923	158	hypothetical membrane protein			OG5_171685	Unknown function	Cenarchaeum Nitrosoarchaeum Nitrosopelagicus Nitrosopumilus Nitrososphaera
Candidatus Cenarchaeum massiliensisK8823_924	455	cysteine--tRNA ligase	K01883	IPR015273;IPR015803;IPR024909;IPR015803	OG5_126755	Translation	Cenarchaeum Nitrosoarchaeum Nitrosopelagicus Nitrosopumilus Nitrososphaera
Candidatus Cenarchaeum massiliensisK8823_925	100	Rieske (2Fe-2S) domain protein		IPR017941;IPR017941	OG5_133960	Oxidation-reduction	Cenarchaeum Nitrosoarchaeum Nitrosopelagicus Nitrosopumilus Nitrososphaera
Candidatus Cenarchaeum massiliensisK8823_926	413	adenosylhomocysteinase	K01251	IPR015878;IPR000043;IPR015878;IPR000043;IPR000043;IPR000043	OG5_127233	Amino acid metabolism	Cenarchaeum Nitrosoarchaeum Nitrosopelagicus Nitrosopumilus Nitrososphaera
Candidatus Cenarchaeum massiliensisK8823_927	392	adenosylhomocysteinase	K01251	IPR000043;IPR015878;IPR000043;IPR015878;IPR000043	OG5_127233	Amino acid metabolism	Cenarchaeum Nitrosoarchaeum Nitrosopelagicus Nitrosopumilus Nitrososphaera
Candidatus Cenarchaeum massiliensisK8823_928	548	methionyl-tRNA synthetase	K01874	IPR015413;IPR014758;IPR023458	OG5_127626	Translation	Cenarchaeum Nitrosoarchaeum Nitrosopelagicus Nitrosopumilus Nitrososphaera
Candidatus Cenarchaeum massiliensisK8823_930	310	translation elongation factor Tu		IPR004161	OG5_161361	Translation	Cenarchaeum Nitrosoarchaeum Nitrosopelagicus Nitrosopumilus Nitrososphaera

Candidatus Cenarchaeum massiliensisK8823_931	335	ketol-acid reductoisomerase	K00053	IPR013023;IPR013116;IPR000506;IPR013023;IPR013023	OG5_129100	Amino acid metabolism	Cenarchaeum Nitrosoarchaeum Nitrosopelagicus Nitrosopumilus Nitrososphaera
Candidatus Cenarchaeum massiliensisK8823_932	142	methylmalonyl CoA mutase B12 binding domain-containing protein	K01849	IPR006159;IPR006158;IPR006158	OG5_149727	Misc	Cenarchaeum Nitrosoarchaeum Nitrosopelagicus Nitrosopumilus Nitrososphaera
Candidatus Cenarchaeum massiliensisK8823_85	158	peptidyl-prolyl cis-trans isomerase	K03768	IPR002130;IPR024936;IPR020892;IPR002130	OG5_127991	Protein modification	Cenarchaeum Nitrosoarchaeum Nitrosopelagicus Nitrosopumilus Nitrososphaera
Candidatus Cenarchaeum massiliensisK8823_960	836	Aminopeptidase N	K08776	IPR024571;IPR014782;IPR001930	OG5_127177	Protein modification	Cenarchaeum Nitrosoarchaeum Nitrosopelagicus Nitrosopumilus Nitrososphaera
Candidatus Cenarchaeum massiliensisK8823_962	305	protein kinase	K07588	IPR005129	OG5_131892	Misc	Cenarchaeum Nitrosoarchaeum Nitrosopelagicus Nitrosopumilus Nitrososphaera
Candidatus Cenarchaeum massiliensisK8823_963	526	methylmalonyl-CoA mutase	K01848	IPR006099;IPR006098	OG5_130968	Misc	Cenarchaeum Nitrosoarchaeum Nitrosopelagicus Nitrosopumilus Nitrososphaera
Candidatus Cenarchaeum massiliensisK8823_964	130	glyoxalase/bleomycin resistance protein/dioxygenase	K05606	IPR017515	OG5_130889	Pyruvate metabolism	Cenarchaeum Nitrosoarchaeum Nitrosopelagicus Nitrosopumilus Nitrososphaera
Candidatus Cenarchaeum massiliensisK8823_965	184	CBS domain protein		IPR001387;IPR001387;IPR000644;IPR001387	OG5_152632	Transcription	Cenarchaeum Nitrosoarchaeum Nitrosopelagicus Nitrosopumilus Nitrososphaera
Candidatus Cenarchaeum massiliensisK8823_86	298	hypothetical membrane protein			nmar|YP_001583075	Unknown function	Cenarchaeum Nitrosoarchaeum Nitrosopelagicus Nitrosopumilus Nitrososphaera
Candidatus Cenarchaeum massiliensisK8823_975	894	Pyruvate phosphate dikinase	K01006	IPR008279;IPR002192;IPR010121;IPR000121;IPR010121;IPR018274	OG5_127082	Pyruvate metabolism	Cenarchaeum Nitrosoarchaeum Nitrosopelagicus Nitrosopumilus Nitrososphaera
Candidatus Cenarchaeum massiliensisK8823_976	220	triosephosphate isomerase	K01803	IPR000652;IPR000652;IPR000652;IPR000652	OG5_139077	Energy	Cenarchaeum Nitrosoarchaeum Nitrosopelagicus Nitrosopumilus Nitrososphaera
Candidatus Cenarchaeum massiliensisK8823_87	49	hypothetical protein			OG5_204746	Unknown function	Cenarchaeum Nitrosoarchaeum Nitrosopelagicus Nitrosopumilus Nitrososphaera
Candidatus Cenarchaeum massiliensisK8823_977	105	Sugar efflux transporter for intercellular exchange			OG5_161428	Unknown function	Cenarchaeum Nitrosoarchaeum Nitrosopelagicus Nitrosopumilus Nitrososphaera
Candidatus Cenarchaeum massiliensisK8823_978	870	DNA polymerase type-B	K02319	IPR006172;IPR006133;IPR006134;IPR017964	OG5_155438	DNA modification	Cenarchaeum Nitrosoarchaeum Nitrosopelagicus Nitrosopumilus Nitrososphaera
Candidatus Cenarchaeum massiliensisK8823_979	176	CMP/dCMP deaminase zinc-binding	K01493	IPR002125;IPR015517;IPR002125	OG5_128503	Nucleotide metabolism	Cenarchaeum Nitrosoarchaeum Nitrosopelagicus Nitrosopumilus Nitrososphaera
Candidatus Cenarchaeum massiliensisK8823_981	220	DEAD-like helicase domain-containing protein	K10896	IPR014001;IPR006935;IPR014001	OG5_128649	DNA modification	Cenarchaeum Nitrosoarchaeum Nitrosopelagicus Nitrosopumilus Nitrososphaera
Candidatus Cenarchaeum massiliensisK8823_983	319	deoxyhypusine synthase	K00809	IPR002773;IPR002773	OG5_127351	Misc	Cenarchaeum Nitrosoarchaeum Nitrosopelagicus Nitrosopumilus Nitrososphaera
Candidatus Cenarchaeum massiliensisK8823_90	297	Ribokinase-like protein	K00852	IPR011611;IPR002173	OG5_168126	Energy	Cenarchaeum Nitrosoarchaeum Nitrosopelagicus Nitrosopumilus Nitrososphaera
Candidatus Cenarchaeum massiliensisK8823_1002	260	hypothetical protein			OG5_161025	Unknown function	Cenarchaeum Nitrosoarchaeum Nitrosopelagicus Nitrosopumilus Nitrososphaera
Candidatus Cenarchaeum massiliensisK8823_91	112	dUTPase		IPR014871	OG5_143192	Misc	Cenarchaeum Nitrosoarchaeum Nitrosopelagicus Nitrosopumilus Nitrososphaera
Candidatus Cenarchaeum massiliensisK8823_1007	251	asparagine synthase	K01953	IPR001962	OG5_127204	Amino acid metabolism	Cenarchaeum Nitrosoarchaeum Nitrosopelagicus Nitrosopumilus Nitrososphaera
Candidatus Cenarchaeum massiliensisK8823_1008	453	adenylosuccinate lyase	K01756	IPR019468;IPR019468;IPR004769;IPR022761;IPR000362	OG5_128176	Nucleotide metabolism	Cenarchaeum Nitrosoarchaeum Nitrosopelagicus Nitrosopumilus Nitrososphaera
Candidatus Cenarchaeum massiliensisK8823_1009	484	tRNA-splicing ligase RtcB	K14415	IPR001233;IPR001233	OG5_127467	Translation	Cenarchaeum Nitrosoarchaeum Nitrosopelagicus Nitrosopumilus Nitrososphaera
Candidatus Cenarchaeum massiliensisK8823_1010	127	hypothetical protein			OG5_196562	Unknown function	Cenarchaeum Nitrosoarchaeum Nitrosopelagicus Nitrosopumilus Nitrososphaera
Candidatus Cenarchaeum massiliensisK8823_1012	179	ribosomal protein S11	K02948	IPR019961;IPR001971;IPR001971;IPR018102;IPR001971	OG5_126681	Translation	Cenarchaeum Nitrosoarchaeum Nitrosopelagicus Nitrosopumilus Nitrososphaera
Candidatus Cenarchaeum massiliensisK8823_1013	227	glucose sorbosone dehydrogenase		IPR012938	OG5_130382	Carbohydrate metabolism	Cenarchaeum Nitrosoarchaeum Nitrosopelagicus Nitrosopumilus Nitrososphaera
Candidatus Cenarchaeum massiliensisK8823_1014	485	dehydrogenase	K17830		OG5_130321	Oxidation-reduction	Cenarchaeum Nitrosoarchaeum Nitrosopelagicus Nitrosopumilus Nitrososphaera
Candidatus Cenarchaeum massiliensisK8823_1015	363	glucose sorbosone dehydrogenase		IPR012938	OG5_130382	Carbohydrate metabolism	Cenarchaeum Nitrosoarchaeum Nitrosopelagicus Nitrosopumilus Nitrososphaera
Candidatus Cenarchaeum massiliensisK8823_1017	151	ribosomal protein L15	K02877	IPR000439;IPR000439	OG5_127033	Translation	Cenarchaeum Nitrosoarchaeum Nitrosopelagicus Nitrosopumilus Nitrososphaera
Candidatus Cenarchaeum massiliensisK8823_1021	219	thiol-disulfide isomerase		IPR000866;IPR012336	nmar|YP_001582586	Oxidation-reduction	Cenarchaeum Nitrosoarchaeum Nitrosopelagicus Nitrosopumilus Nitrososphaera
Candidatus Cenarchaeum massiliensisK8823_1025	162	HEAT repeat-containing PBS lyase	K06072	IPR004155	OG5_128124	Misc	Cenarchaeum Nitrosoarchaeum Nitrosopelagicus Nitrosopumilus Nitrososphaera
Candidatus Cenarchaeum massiliensisK8823_1026	697	SMC domain-containing protein	K03546	IPR003395	OG5_149650	Transcription	Cenarchaeum Nitrosoarchaeum Nitrosopelagicus Nitrosopumilus Nitrososphaera
Candidatus Cenarchaeum massiliensisK8823_1027	377	metallophosphoesterase		IPR024654	OG5_131553	Misc	Cenarchaeum Nitrosoarchaeum Nitrosopelagicus Nitrosopumilus Nitrososphaera
Candidatus Cenarchaeum massiliensisK8823_1028	509	hypothetical protein	K06915	IPR002789	OG5_134229	Unknown function	Cenarchaeum Nitrosoarchaeum Nitrosopelagicus Nitrosopumilus Nitrososphaera
Candidatus Cenarchaeum massiliensisK8823_1029	280	nuclease		IPR018977;IPR018977	OG5_161025	Unknown function	Cenarchaeum Nitrosoarchaeum Nitrosopelagicus Nitrosopumilus Nitrososphaera
Candidatus Cenarchaeum massiliensisK8823_1030	202	hypothetical protein			OG5_151395	Carbohydrate metabolism	Cenarchaeum Nitrosoarchaeum Nitrosopelagicus Nitrosopumilus Nitrososphaera
Candidatus Cenarchaeum massiliensisK8823_1032	311	3-phosphoglycerate dehydrogenase	K00058	IPR006140;IPR006139;IPR029752	OG5_127006	Amino acid metabolism	Cenarchaeum Nitrosoarchaeum Nitrosopelagicus Nitrosopumilus Nitrososphaera
Candidatus Cenarchaeum massiliensisK8823_1039	455	aspartate ammonia-lyase	K01744	IPR018951;IPR022761;IPR000362;IPR020557	OG5_127258	Energy	Cenarchaeum Nitrosoarchaeum Nitrosopelagicus Nitrosopumilus Nitrososphaera
Candidatus Cenarchaeum massiliensisK8823_1040	421	MiaB-like tRNA modifying enzyme	K06168	IPR006638;IPR005839;IPR006466;IPR007197;IPR013848;IPR023970;IPR020612;IPR013848	OG5_126807	Translation	Cenarchaeum Nitrosoarchaeum Nitrosopelagicus Nitrosopumilus Nitrososphaera
Candidatus Cenarchaeum massiliensisK8823_1041	308	cell division protein FtsZ		IPR003008;IPR003008	OG5_128807	Cell & development	Cenarchaeum Nitrosoarchaeum Nitrosopelagicus Nitrosopumilus Nitrososphaera
Candidatus Cenarchaeum massiliensisK8823_1042	194	Ribosomal protein L31e	K02910	IPR000054;IPR000054;IPR020052;IPR000054;IPR000054	OG5_126907	Translation	Cenarchaeum Nitrosoarchaeum Nitrosopelagicus Nitrosopumilus Nitrososphaera
Candidatus Cenarchaeum massiliensisK8823_1043	52	ribosomal protein L39		IPR000077;IPR000077	OG5_127376	Translation	Cenarchaeum Nitrosoarchaeum Nitrosopelagicus Nitrosopumilus Nitrososphaera
Candidatus Cenarchaeum massiliensisK8823_1045	182	NUDIX hydrolase		IPR000086;IPR000086	OG5_127325	Misc	Cenarchaeum Nitrosoarchaeum Nitrosopelagicus Nitrosopumilus Nitrososphaera
Candidatus Cenarchaeum massiliensisK8823_1046	241	5,10-methylenetetrahydrofolate reductase-like protein			nmar|YP_001582600	Amino acid metabolism	Cenarchaeum Nitrosoarchaeum Nitrosopelagicus Nitrosopumilus Nitrososphaera
Candidatus Cenarchaeum massiliensisK8823_1049	97	hypothetical protein			nmar|YP_001582127	Unknown function	Cenarchaeum Nitrosoarchaeum Nitrosopelagicus Nitrosopumilus Nitrososphaera
Candidatus Cenarchaeum massiliensisK8823_97	347	tyrosyl-tRNA synthetase	K01866	IPR002305	OG5_127567	Translation	Cenarchaeum Nitrosoarchaeum Nitrosopelagicus Nitrosopumilus Nitrososphaera
Candidatus Cenarchaeum massiliensisK8823_1050	186	ribosomal protein L6	K02940	IPR020040;IPR000702;IPR002359	OG5_126993	Translation	Cenarchaeum Nitrosoarchaeum Nitrosopelagicus Nitrosopumilus Nitrososphaera
Candidatus Cenarchaeum massiliensisK8823_1051	130	ribosomal protein S8	K02957	IPR000630;IPR000630;IPR000630	OG5_126878	Translation	Cenarchaeum Nitrosoarchaeum Nitrosopelagicus Nitrosopumilus Nitrososphaera
Candidatus Cenarchaeum massiliensisK8823_1052	59	ribosomal protein S14	K02980	IPR001209;IPR018271;IPR023676	OG5_127205	Translation	Cenarchaeum Nitrosoarchaeum Nitrosopelagicus Nitrosopumilus Nitrososphaera
Candidatus Cenarchaeum massiliensisK8823_1053	174	ribosomal protein L5	K02931	IPR031310;IPR031309;IPR022804	OG5_126688	Translation	Cenarchaeum Nitrosoarchaeum Nitrosopelagicus Nitrosopumilus Nitrososphaera
Candidatus Cenarchaeum massiliensisK8823_1054	239	ribosomal protein S4e	K02987	IPR013843;IPR013845;IPR000876;IPR013845;IPR000876;IPR002942	OG5_126890	Translation	Cenarchaeum Nitrosoarchaeum Nitrosopelagicus Nitrosopumilus Nitrososphaera
Candidatus Cenarchaeum massiliensisK8823_1055	204	ribosomal protein L24P	K02895	IPR005756;IPR005756;IPR005756	OG5_126931	Translation	Cenarchaeum Nitrosoarchaeum Nitrosopelagicus Nitrosopumilus Nitrososphaera
Candidatus Cenarchaeum massiliensisK8823_1056	144	ribosomal protein L14	K02874	IPR000218;IPR000218;IPR019972;IPR000218	OG5_126678	Translation	Cenarchaeum Nitrosoarchaeum Nitrosopelagicus Nitrosopumilus Nitrososphaera
Candidatus Cenarchaeum massiliensisK8823_1057	109	ribosomal protein S17	K02949	IPR000266;IPR000266;IPR000266;IPR000266	OG5_126986	Translation	Cenarchaeum Nitrosoarchaeum Nitrosopelagicus Nitrosopumilus Nitrososphaera
Candidatus Cenarchaeum massiliensisK8823_1058	86	Ribonuclease P protein component 1		IPR002730;IPR002730;IPR023538	OG5_142319	RNA modification	Cenarchaeum Nitrosoarchaeum Nitrosopelagicus Nitrosopumilus Nitrososphaera
Candidatus Cenarchaeum massiliensisK8823_1059	67	ribosomal protein L29		IPR001854;IPR001854;IPR001854	OG5_127176	Translation	Cenarchaeum Nitrosoarchaeum Nitrosopelagicus Nitrosopumilus Nitrososphaera
Candidatus Cenarchaeum massiliensisK8823_98	255	precorrin-3B C17-methyltransferase (cobJ, cbiH)	K05934	IPR006363;IPR000878	OG5_135738	Metabolism of cofactors and vitamins	Cenarchaeum Nitrosoarchaeum Nitrosopelagicus Nitrosopumilus Nitrososphaera
Candidatus Cenarchaeum massiliensisK8823_1060	246	ribosomal protein S3	K02982	IPR004087;IPR004044;IPR005703;IPR004044;IPR027488	OG5_126820	Translation	Cenarchaeum Nitrosoarchaeum Nitrosopelagicus Nitrosopumilus Nitrososphaera
Candidatus Cenarchaeum massiliensisK8823_1061	152	ribosomal protein L22	K02890	IPR001063;IPR005721;IPR005721;IPR005721	OG5_126900	Translation	Cenarchaeum Nitrosoarchaeum Nitrosopelagicus Nitrosopumilus Nitrososphaera
Candidatus Cenarchaeum massiliensisK8823_1062	131	ribosomal protein S19	K02958	IPR002222;IPR005713;IPR002222;IPR020934;IPR002222	OG5_126687	Translation	Cenarchaeum Nitrosoarchaeum Nitrosopelagicus Nitrosopumilus Nitrososphaera
Candidatus Cenarchaeum massiliensisK8823_1063	88	ribosomal protein L23		IPR013025;IPR013025	OG5_126659	Translation	Cenarchaeum Nitrosoarchaeum Nitrosopelagicus Nitrosopumilus Nitrososphaera
Candidatus Cenarchaeum massiliensisK8823_1064	270	ribosomal protein L4	K02930	IPR002136	OG5_127050	Translation	Cenarchaeum Nitrosoarchaeum Nitrosopelagicus Nitrosopumilus Nitrososphaera
Candidatus Cenarchaeum massiliensisK8823_1065	227	ribosomal protein L3	K02906	IPR000597	OG5_126874	Translation	Cenarchaeum Nitrosoarchaeum Nitrosopelagicus Nitrosopumilus Nitrososphaera
Candidatus Cenarchaeum massiliensisK8823_1066	163	ribosomal protein L3	K02906	IPR000597	OG5_126874	Translation	Cenarchaeum Nitrosoarchaeum Nitrosopelagicus Nitrosopumilus Nitrososphaera
Candidatus Cenarchaeum massiliensisK8823_1067	264	hypothetical protein	K09142	IPR003750;IPR003750	OG5_128701	Unknown function	Cenarchaeum Nitrosoarchaeum Nitrosopelagicus Nitrosopumilus Nitrososphaera
Candidatus Cenarchaeum massiliensisK8823_1068	245	proteasome subunit alpha	K03432	IPR000426;IPR001353;IPR000426;IPR023332	OG5_127424	Protein modification	Cenarchaeum Nitrosoarchaeum Nitrosopelagicus Nitrosopumilus Nitrososphaera
Candidatus Cenarchaeum massiliensisK8823_1069	138	hypothetical protein	K09738	IPR007171	OG5_141330	Unknown function	Cenarchaeum Nitrosoarchaeum Nitrosopelagicus Nitrosopumilus Nitrososphaera
Candidatus Cenarchaeum massiliensisK8823_1070	115	hypothetical protein			nmar|YP_001582147	Unknown function	Cenarchaeum Nitrosoarchaeum Nitrosopelagicus Nitrosopumilus Nitrososphaera
Candidatus Cenarchaeum massiliensisK8823_1071	156	pyridoxamine 5'-phosphate oxidase		IPR011576	OG5_216789	Oxidation-reduction	Cenarchaeum Nitrosoarchaeum Nitrosopelagicus Nitrosopumilus Nitrososphaera
Candidatus Cenarchaeum massiliensisK8823_1072	214	Snf7 domain-containing protein		IPR005024	OG5_150062	Transport	Cenarchaeum Nitrosoarchaeum Nitrosopelagicus Nitrosopumilus Nitrososphaera
Candidatus Cenarchaeum massiliensisK8823_1074	241	cytochrome c biogenesis protein	K06196	IPR003834	OG5_130369	Oxidation-reduction	Cenarchaeum Nitrosoarchaeum Nitrosopelagicus Nitrosopumilus Nitrososphaera
Candidatus Cenarchaeum massiliensisK8823_1076	74	transcriptional regulator AsnC family		IPR019887	OG5_135100	Transcription	Cenarchaeum Nitrosoarchaeum Nitrosopelagicus Nitrosopumilus Nitrososphaera
Candidatus Cenarchaeum massiliensisK8823_1077	138	Glyoxalase/bleomycin resistance protein/dioxygenase		IPR025870	OG5_143131	Pyruvate metabolism	Cenarchaeum Nitrosoarchaeum Nitrosopelagicus Nitrosopumilus Nitrososphaera
Candidatus Cenarchaeum massiliensisK8823_101	464	aspartate kinase	K00928	IPR001341;IPR002912;IPR001048;IPR002912	OG5_128018	Amino acid metabolism	Cenarchaeum Nitrosoarchaeum Nitrosopelagicus Nitrosopumilus Nitrososphaera
Candidatus Cenarchaeum massiliensisK8823_1092	404	threonine ammonia-lyase	K01754	IPR001926;IPR005789;IPR000634;IPR002912	OG5_126759	Amino acid metabolism	Cenarchaeum Nitrosoarchaeum Nitrosopelagicus Nitrosopumilus Nitrososphaera
Candidatus Cenarchaeum massiliensisK8823_1093	376	N5-carboxyaminoimidazole ribonucleotide synthase	K01589	IPR003135;IPR005875;IPR005875;IPR011761	OG5_127668	Nucleotide metabolism	Cenarchaeum Nitrosoarchaeum Nitrosopelagicus Nitrosopumilus Nitrososphaera
Candidatus Cenarchaeum massiliensisK8823_1094	176	metal dependent phosphohydrolase	K07023	IPR003607;IPR006674	OG5_127807	Misc	Cenarchaeum Nitrosoarchaeum Nitrosopelagicus Nitrosopumilus Nitrososphaera
Candidatus Cenarchaeum massiliensisK8823_102	248	proliferating cell nuclear antigen PcnA	K04802	IPR022648;IPR022649;IPR000730;IPR000730;IPR000730	OG5_127352	DNA modification	Cenarchaeum Nitrosoarchaeum Nitrosopelagicus Nitrosopumilus Nitrososphaera
Candidatus Cenarchaeum massiliensisK8823_1096	126	hypothetical protein			OG5_241574	Unknown function	Cenarchaeum Nitrosoarchaeum Nitrosopelagicus Nitrosopumilus Nitrososphaera
Candidatus Cenarchaeum massiliensisK8823_1097	416	LOR/SDH bifunctional domain-containing protein		IPR007545;IPR002580;IPR005239	OG5_149706	Unknown function	Cenarchaeum Nitrosoarchaeum Nitrosopelagicus Nitrosopumilus Nitrososphaera
Candidatus Cenarchaeum massiliensisK8823_1099	176	hypothetical membrane protein			OG5_130966	Misc	Cenarchaeum Nitrosoarchaeum Nitrosopelagicus Nitrosopumilus Nitrososphaera
Candidatus Cenarchaeum massiliensisK8823_1100	224	nucleotidyl transferase	K00966	IPR005835	OG5_127109	Carbohydrate metabolism	Cenarchaeum Nitrosoarchaeum Nitrosopelagicus Nitrosopumilus Nitrososphaera
Candidatus Cenarchaeum massiliensisK8823_1102	316	luciferase family protein	K00320	IPR011251	OG5_140202	Oxidation-reduction	Cenarchaeum Nitrosoarchaeum Nitrosopelagicus Nitrosopumilus Nitrososphaera
Candidatus Cenarchaeum massiliensisK8823_1103	348	Thiolase	K00626	IPR020616;IPR020617	OG5_126757	Lipid metabolism	Cenarchaeum Nitrosoarchaeum Nitrosopelagicus Nitrosopumilus Nitrososphaera
Candidatus Cenarchaeum massiliensisK8823_1104	68	hypothetical protein			nmar|YP_001582174	Unknown function	Cenarchaeum Nitrosoarchaeum Nitrosopelagicus Nitrosopumilus Nitrososphaera
Candidatus Cenarchaeum massiliensisK8823_1105	181	Holliday junction resolvase			nmar|YP_001582171	Misc	Cenarchaeum Nitrosoarchaeum Nitrosopelagicus Nitrosopumilus Nitrososphaera
Candidatus Cenarchaeum massiliensisK8823_103	103	transcription factor S	K03057	IPR001222;IPR001222;IPR006288;IPR001222;IPR001222	OG5_127419	Transcription	Cenarchaeum Nitrosoarchaeum Nitrosopelagicus Nitrosopumilus Nitrososphaera
Candidatus Cenarchaeum massiliensisK8823_1106	155	hypothetical protein			OG5_141261	Unknown function	Cenarchaeum Nitrosoarchaeum Nitrosopelagicus Nitrosopumilus Nitrososphaera
Candidatus Cenarchaeum massiliensisK8823_1107	105	hypothetical protein			nmar|YP_001582169	Unknown function	Cenarchaeum Nitrosoarchaeum Nitrosopelagicus Nitrosopumilus Nitrososphaera
Candidatus Cenarchaeum massiliensisK8823_1109	455	Threonine synthase	K01733	IPR001926;IPR004450	OG5_130923	Amino acid metabolism	Cenarchaeum Nitrosoarchaeum Nitrosopelagicus Nitrosopumilus Nitrososphaera
Candidatus Cenarchaeum massiliensisK8823_1110	419	hemolysin	K03699	IPR000644;IPR005170;IPR002550;IPR005170;IPR000644;IPR000644;IPR000644	OG5_126632	Misc	Cenarchaeum Nitrosoarchaeum Nitrosopelagicus Nitrosopumilus Nitrososphaera
Candidatus Cenarchaeum massiliensisK8823_1111	348	glyceraldehyde-3-phosphate dehydrogenase	K00150		OG5_140172	Energy	Cenarchaeum Nitrosoarchaeum Nitrosopelagicus Nitrosopumilus Nitrososphaera
Candidatus Cenarchaeum massiliensisK8823_104	88	hypothetical protein			nmar|YP_001583091	Transcription	Cenarchaeum Nitrosoarchaeum Nitrosopelagicus Nitrosopumilus Nitrososphaera
Candidatus Cenarchaeum massiliensisK8823_1112	225	protein disulfide-isomerase		IPR012336;IPR012336	OG5_142695	Oxidation-reduction	Cenarchaeum Nitrosoarchaeum Nitrosopelagicus Nitrosopumilus Nitrososphaera
Candidatus Cenarchaeum massiliensisK8823_1113	464	3-hydroxy-3-methylglutaryl-CoA synthase	K01641	IPR013528;IPR013746	OG5_127526	Amino acid metabolism	Cenarchaeum Nitrosoarchaeum Nitrosopelagicus Nitrosopumilus Nitrososphaera
Candidatus Cenarchaeum massiliensisK8823_1114	231	dethiobiotin synthase	K01935	IPR004472;IPR004472	OG5_131754	Metabolism of cofactors and vitamins	Cenarchaeum Nitrosoarchaeum Nitrosopelagicus Nitrosopumilus Nitrososphaera
Candidatus Cenarchaeum massiliensisK8823_1117	442	Aminotransferase class-III	K00833	IPR005814;IPR005815;IPR005814;IPR005814	OG5_128068	Metabolism of cofactors and vitamins	Cenarchaeum Nitrosoarchaeum Nitrosopelagicus Nitrosopumilus Nitrososphaera
Candidatus Cenarchaeum massiliensisK8823_1120	245	adenylate/guanylate cyclase	K01768	IPR001054;IPR001054;IPR001054	OG5_156714	Nucleotide metabolism	Cenarchaeum Nitrosoarchaeum Nitrosopelagicus Nitrosopumilus Nitrososphaera

Candidatus Cenarchaeum massiliensis	K8823_105	178	hypothetical protein			nmar|YP_001583092	Unknown function	Cenarchaeum Nitrosoarchaeum Nitrosopelagicus Nitrosopumilus Nitrososphaera
Candidatus Cenarchaeum massiliensis	K8823_1121	283	hypothetical protein			OG5_169435	Unknown function	Cenarchaeum Nitrosoarchaeum Nitrosopelagicus Nitrosopumilus Nitrososphaera
Candidatus Cenarchaeum massiliensis	K8823_1122	464	aspartyl/glutamyl-tRNA amidotransferase subunit B	K02434	IPR018027;IPR018027;IPR004413;IPR006075;IPR017959;IPR017958;IPR004413	OG5_127330	Translation	Cenarchaeum Nitrosoarchaeum Nitrosopelagicus Nitrosopumilus Nitrososphaera
Candidatus Cenarchaeum massiliensis	K8823_1123	481	aspartyl/glutamyl-tRNA amidotransferase subunit A	K02433	IPR004412;IPR000120;IPR000120;IPR020556;IPR004412	OG5_127466	Translation	Cenarchaeum Nitrosoarchaeum Nitrosopelagicus Nitrosopumilus Nitrososphaera
Candidatus Cenarchaeum massiliensis	K8823_1125	434	aspartate--tRNA(Asn) ligase	K01876	IPR004365;IPR004364;IPR004523;IPR018150;IPR006195	OG5_126756	Translation	Cenarchaeum Nitrosoarchaeum Nitrosopelagicus Nitrosopumilus Nitrososphaera
Candidatus Cenarchaeum massiliensis	K8823_1127	340	5-formaminoimidazole-4-carboxamide-1-(beta)-D-ribofuranosyl 5'-monophosphate synthetase	K06863	IPR010672;IPR009720;IPR011761;IPR023656	OG5_137370	Nucleotide metabolism	Cenarchaeum Nitrosoarchaeum Nitrosopelagicus Nitrosopumilus Nitrososphaera
Candidatus Cenarchaeum massiliensis	K8823_1128	114	Transcriptional regulator		IPR002831	OG5_182872	Transcription	Cenarchaeum Nitrosoarchaeum Nitrosopelagicus Nitrosopumilus Nitrososphaera
Candidatus Cenarchaeum massiliensis	K8823_1129	242	Cobalt transporter subunit (CbtA)		IPR012666	nmar|YP_001582212	Transport	Cenarchaeum Nitrosoarchaeum Nitrosopelagicus Nitrosopumilus Nitrososphaera
Candidatus Cenarchaeum massiliensis	K8823_1130	71	Cobalt transporter subunit (CbtB)		IPR012667	nmar|YP_001582213	Transport	Cenarchaeum Nitrosoarchaeum Nitrosopelagicus Nitrosopumilus Nitrososphaera
Candidatus Cenarchaeum massiliensis	K8823_1131	235	DSBA oxidoreductase		IPR012336	OG5_142695	Oxidation-reduction	Cenarchaeum Nitrosoarchaeum Nitrosopelagicus Nitrosopumilus Nitrososphaera
Candidatus Cenarchaeum massiliensis	K8823_1132	432	peptide chain release factor 1	K03265	IPR005141;IPR005140;IPR004403;IPR005142;IPR004403	OG5_127250	Translation	Cenarchaeum Nitrosoarchaeum Nitrosopelagicus Nitrosopumilus Nitrososphaera
Candidatus Cenarchaeum massiliensis	K8823_1133	134	HIT family protein	K02503	IPR001310;IPR001310;IPR019808;IPR011146	OG5_126794	RNA modification	Cenarchaeum Nitrosoarchaeum Nitrosopelagicus Nitrosopumilus Nitrososphaera
Candidatus Cenarchaeum massiliensis	K8823_1136	559	dihydroxy-acid dehydratase	K01687	IPR000581;IPR004404;IPR000581;IPR020558;IPR020558;IPR004404	OG5_128618	Amino acid metabolism	Cenarchaeum Nitrosoarchaeum Nitrosopelagicus Nitrosopumilus Nitrososphaera
Candidatus Cenarchaeum massiliensis	K8823_1137	449	Anthranilate synthase component 1	K01657	IPR015890;IPR006805	OG5_128544	Amino acid metabolism	Cenarchaeum Nitrosoarchaeum Nitrosopelagicus Nitrosopumilus Nitrososphaera
Candidatus Cenarchaeum massiliensis	K8823_1138	198	Anthranilate synthase component 2	K01658	IPR017926;IPR006221;IPR017926	OG5_128663	Amino acid metabolism	Cenarchaeum Nitrosoarchaeum Nitrosopelagicus Nitrosopumilus Nitrososphaera
Candidatus Cenarchaeum massiliensis	K8823_1139	344	Anthranilate phosphoribosyltransferase	K00766	IPR005940;IPR000312;IPR017459;IPR005940	OG5_129303	Amino acid metabolism	Cenarchaeum Nitrosoarchaeum Nitrosopelagicus Nitrosopumilus Nitrososphaera
Candidatus Cenarchaeum massiliensis	K8823_1141	399	tryptophan synthase subunit beta	K01696	IPR001926;IPR006654;IPR006654;IPR006653;IPR023026	OG5_128016	Amino acid metabolism	Cenarchaeum Nitrosoarchaeum Nitrosopelagicus Nitrosopumilus Nitrososphaera
Candidatus Cenarchaeum massiliensis	K8823_1142	271	Tryptophan synthase subunit alpha	K01695	IPR002028;IPR002028;IPR018204;IPR002028	OG5_130207	Amino acid metabolism	Cenarchaeum Nitrosoarchaeum Nitrosopelagicus Nitrosopumilus Nitrososphaera
Candidatus Cenarchaeum massiliensis	K8823_1143	531	CTP synthase	K01937	IPR017926;IPR017456;IPR004468;IPR004468;IPR017926	OG5_126851	Nucleotide metabolism	Cenarchaeum Nitrosoarchaeum Nitrosopelagicus Nitrosopumilus Nitrososphaera
Candidatus Cenarchaeum massiliensis	K8823_1145	129	hypothetical protein			nmar|YP_001582738	Unknown function	Cenarchaeum Nitrosoarchaeum Nitrosopelagicus Nitrosopumilus Nitrososphaera
Candidatus Cenarchaeum massiliensis	K8823_1146	134	hypothetical protein			nmar|YP_001582737	Unknown function	Cenarchaeum Nitrosoarchaeum Nitrosopelagicus Nitrosopumilus Nitrososphaera
Candidatus Cenarchaeum massiliensis	K8823_1147	94	hypothetical protein			nmar|YP_001582736	Unknown function	Cenarchaeum Nitrosoarchaeum Nitrosopelagicus Nitrosopumilus Nitrososphaera
Candidatus Cenarchaeum massiliensis	K8823_1148	264	methylthioadenosine phosphorylase	K00772	IPR000845;IPR010044;IPR001369;IPR018099;IPR010044	OG5_128424	Amino acid metabolism	Cenarchaeum Nitrosoarchaeum Nitrosopelagicus Nitrosopumilus Nitrososphaera
Candidatus Cenarchaeum massiliensis	K8823_1149	172	adenine phosphoribosyltransferase	K00759	IPR000836;IPR005764	OG5_127379	Nucleotide metabolism	Cenarchaeum Nitrosoarchaeum Nitrosopelagicus Nitrosopumilus Nitrososphaera
Candidatus Cenarchaeum massiliensis	K8823_107	50	hypothetical protein			STG_0030	Unknown function	Cenarchaeum Nitrosoarchaeum Nitrosopelagicus Nitrosopumilus Nitrososphaera
Candidatus Cenarchaeum massiliensis	K8823_1150	290	PfkB domain-containing protein			OG5_196258	Misc	Cenarchaeum Nitrosoarchaeum Nitrosopelagicus Nitrosopumilus Nitrososphaera
Candidatus Cenarchaeum massiliensis	K8823_1151	98	ribosomal protein S26e	K02976	IPR000892;IPR000892;IPR000892	OG5_127138	Translation	Cenarchaeum Nitrosoarchaeum Nitrosopelagicus Nitrosopumilus Nitrososphaera
Candidatus Cenarchaeum massiliensis	K8823_1152	195	CDP-alcohol phosphatidyltransferase	K17884	IPR000462;IPR000462	OG5_135089	Lipid metabolism	Cenarchaeum Nitrosoarchaeum Nitrosopelagicus Nitrosopumilus Nitrososphaera
Candidatus Cenarchaeum massiliensis	K8823_1153	290	Agmatinase	K01480	IPR005925;IPR006035;IPR006035;IPR020855;IPR006035	OG5_127636	Amino acid metabolism	Cenarchaeum Nitrosoarchaeum Nitrosopelagicus Nitrosopumilus Nitrososphaera
Candidatus Cenarchaeum massiliensis	K8823_1154	619	threonine--tRNA ligase	K01868	IPR015011;IPR002314;IPR004154;IPR002320;IPR015011;IPR002320;IPR006195	OG5_126764	Translation	Cenarchaeum Nitrosoarchaeum Nitrosopelagicus Nitrosopumilus Nitrososphaera
Candidatus Cenarchaeum massiliensis	K8823_1155	370	Deoxyhypusine synthase	K00809	IPR002773;IPR002773	OG5_127351	Misc	Cenarchaeum Nitrosoarchaeum Nitrosopelagicus Nitrosopumilus Nitrososphaera
Candidatus Cenarchaeum massiliensis	K8823_1156	70	hypothetical protein			nmar|YP_001582263	Unknown function	Cenarchaeum Nitrosoarchaeum Nitrosopelagicus Nitrosopumilus Nitrososphaera
Candidatus Cenarchaeum massiliensis	K8823_1157	101	ribosomal protein S25		IPR004977	OG5_126998	Translation	Cenarchaeum Nitrosoarchaeum Nitrosopelagicus Nitrosopumilus Nitrososphaera
Candidatus Cenarchaeum massiliensis	K8823_1158	126	Cyclophilin-like domain containing protein		IPR025658	nmar|YP_001582265	Protein modification	Cenarchaeum Nitrosoarchaeum Nitrosopelagicus Nitrosopumilus Nitrososphaera
Candidatus Cenarchaeum massiliensis	K8823_1160	318	exonuclease	K07577		OG5_141157	RNA modification	Cenarchaeum Nitrosoarchaeum Nitrosopelagicus Nitrosopumilus Nitrososphaera
Candidatus Cenarchaeum massiliensis	K8823_1161	205	phosphoribosylglycinamide formyltransferase	K11175	IPR004607;IPR002376;IPR001555;IPR004607	OG5_129694	Nucleotide metabolism	Cenarchaeum Nitrosoarchaeum Nitrosopelagicus Nitrosopumilus Nitrososphaera
Candidatus Cenarchaeum massiliensis	K8823_1162	142	cupin		IPR013096	nmar|YP_001582449	Misc	Cenarchaeum Nitrosoarchaeum Nitrosopelagicus Nitrosopumilus Nitrososphaera
Candidatus Cenarchaeum massiliensis	K8823_1163	287	bifunctional 5,10-methylene-tetrahydrofolate dehydrogenase/5,10-methylene-tetrahydrofolate cyclohydrolase	K01491	IPR020631;IPR020630;IPR020867;IPR000672	OG5_126671	Energy	Cenarchaeum Nitrosoarchaeum Nitrosopelagicus Nitrosopumilus Nitrososphaera
Candidatus Cenarchaeum massiliensis	K8823_1164	185	5-formyltetrahydrofolate cyclo-ligase	K01934	IPR002698;IPR002698;IPR002698	OG5_127997	Metabolism of cofactors and vitamins	Cenarchaeum Nitrosoarchaeum Nitrosopelagicus Nitrosopumilus Nitrososphaera
Candidatus Cenarchaeum massiliensis	K8823_1165	413	phosphoribosylamine--glycine ligase	K01945	IPR020560;IPR020561;IPR020562;IPR000115;IPR000115;IPR011761	OG5_126894	Nucleotide metabolism	Cenarchaeum Nitrosoarchaeum Nitrosopelagicus Nitrosopumilus Nitrososphaera
Candidatus Cenarchaeum massiliensis	K8823_1166	1067	isoleucine--tRNA ligase	K01870	IPR013155;IPR002301;IPR002300;IPR001412	OG5_126684	Translation	Cenarchaeum Nitrosoarchaeum Nitrosopelagicus Nitrosopumilus Nitrososphaera
Candidatus Cenarchaeum massiliensis	K8823_1167	109	hypothetical protein			nmar|YP_001582456	Unknown function	Cenarchaeum Nitrosoarchaeum Nitrosopelagicus Nitrosopumilus Nitrososphaera
Candidatus Cenarchaeum massiliensis	K8823_108	167	Protein-tyrosine-phosphatase	K14165	IPR020422;IPR003595;IPR000340;IPR020422;IPR000387	OG5_134117	Misc	Cenarchaeum Nitrosoarchaeum Nitrosopelagicus Nitrosopumilus Nitrososphaera
Candidatus Cenarchaeum massiliensis	K8823_1168	368	succinate--CoA ligase	K01903	IPR005811;IPR013650;IPR005809;IPR017866;IPR011761	OG5_126862	Energy	Cenarchaeum Nitrosoarchaeum Nitrosopelagicus Nitrosopumilus Nitrososphaera
Candidatus Cenarchaeum massiliensis	K8823_1169	306	succinate--CoA ligase subunit alpha	K01902	IPR003781;IPR003781;IPR005811;IPR017440	OG5_127106	Energy	Cenarchaeum Nitrosoarchaeum Nitrosopelagicus Nitrosopumilus Nitrososphaera
Candidatus Cenarchaeum massiliensis	K8823_109	236	cyclase	K07130	IPR007325	OG5_130210	Amino acid metabolism	Cenarchaeum Nitrosoarchaeum Nitrosopelagicus Nitrosopumilus Nitrososphaera
Candidatus Cenarchaeum massiliensis	K8823_110	89	AsnC family protein			OG5_135100	Transcription	Cenarchaeum Nitrosoarchaeum Nitrosopelagicus Nitrosopumilus Nitrososphaera
Candidatus Cenarchaeum massiliensis	K8823_111	378	cysteine desulfurase	K11717	IPR000192	OG5_128800	Amino acid metabolism	Cenarchaeum Nitrosoarchaeum Nitrosopelagicus Nitrosopumilus Nitrososphaera
Candidatus Cenarchaeum massiliensis	K8823_1196	296	methionine aminopeptidase	K01265	IPR002468;IPR000994;IPR002468;IPR018349;IPR028595	OG5_127231	Protein modification	Cenarchaeum Nitrosoarchaeum Nitrosopelagicus Nitrosopumilus Nitrososphaera
Candidatus Cenarchaeum massiliensis	K8823_1198	369	hypothetical protein		IPR009995	OG5_161029	Unknown function	Cenarchaeum Nitrosoarchaeum Nitrosopelagicus Nitrosopumilus Nitrososphaera
Candidatus Cenarchaeum massiliensis	K8823_1199	186	TATA-box binding protein (TBP)	K03120	IPR000814;IPR000814;IPR030491;IPR000814	OG5_126912	Transcription	Cenarchaeum Nitrosoarchaeum Nitrosopelagicus Nitrosopumilus Nitrososphaera
Candidatus Cenarchaeum massiliensis	K8823_1200	107	tRNA-binding protein	K01874	IPR002547;IPR002547	OG5_127125	Translation	Cenarchaeum Nitrosoarchaeum Nitrosopelagicus Nitrosopumilus Nitrososphaera
Candidatus Cenarchaeum massiliensis	K8823_112	183	dephospho-CoA kinase			OG5_137351	Misc	Cenarchaeum Nitrosoarchaeum Nitrosopelagicus Nitrosopumilus Nitrososphaera
Candidatus Cenarchaeum massiliensis	K8823_1202	142	Cyclase/dehydrase		IPR019587	OG5_165858	Misc	Cenarchaeum Nitrosoarchaeum Nitrosopelagicus Nitrosopumilus Nitrososphaera
Candidatus Cenarchaeum massiliensis	K8823_1203	399	glycosyl transferase		IPR001173	OG5_147346	Misc	Cenarchaeum Nitrosoarchaeum Nitrosopelagicus Nitrosopumilus Nitrososphaera
Candidatus Cenarchaeum massiliensis	K8823_1204	86	AsnC family transcriptional regulator		IPR019887	OG5_135100	Transcription	Cenarchaeum Nitrosoarchaeum Nitrosopelagicus Nitrosopumilus Nitrososphaera
Candidatus Cenarchaeum massiliensis	K8823_1205	97	hypothetical protein			nmar|YP_001582518	Unknown function	Cenarchaeum Nitrosoarchaeum Nitrosopelagicus Nitrosopumilus Nitrososphaera
Candidatus Cenarchaeum massiliensis	K8823_1206	1178	chromosome segregation ATPase	K03529	IPR010935;IPR010935;IPR003395	OG5_127360	Transcription	Cenarchaeum Nitrosoarchaeum Nitrosopelagicus Nitrosopumilus Nitrososphaera
Candidatus Cenarchaeum massiliensis	K8823_1208	163	Peptide methionine sulfoxide reductase	K07304	IPR002569;IPR002569;IPR028427;IPR002569	OG5_126847	Oxidation-reduction	Cenarchaeum Nitrosoarchaeum Nitrosopelagicus Nitrosopumilus Nitrososphaera
Candidatus Cenarchaeum massiliensis	K8823_1209	283	lysine biosynthesis enzyme LysX	K05827	IPR011870;IPR004666;IPR013651;IPR011761	OG5_130067	Amino acid metabolism	Cenarchaeum Nitrosoarchaeum Nitrosopelagicus Nitrosopumilus Nitrososphaera
Candidatus Cenarchaeum massiliensis	K8823_1210	55	lysine biosynthesis protein LysW			OG5_157236	Amino acid metabolism	Cenarchaeum Nitrosoarchaeum Nitrosopelagicus Nitrosopumilus Nitrososphaera
Candidatus Cenarchaeum massiliensis	K8823_1211	399	argininosuccinate synthase	K01940	IPR001518;IPR001518;IPR018223;IPR023434	OG5_127707	Amino acid metabolism	Cenarchaeum Nitrosoarchaeum Nitrosopelagicus Nitrosopumilus Nitrososphaera
Candidatus Cenarchaeum massiliensis	K8823_1212	331	aliphatic sulfonate ABC transporter periplasmic ligand-binding protein	K02051	IPR010067	OG5_137895	Transport	Cenarchaeum Nitrosoarchaeum Nitrosopelagicus Nitrosopumilus Nitrososphaera
Candidatus Cenarchaeum massiliensis	K8823_1213	253	nitrate ABC transporter ATP-binding protein	K02049	IPR003593;IPR003439;IPR003439	OG5_130079	Nucleotide metabolism	Cenarchaeum Nitrosoarchaeum Nitrosopelagicus Nitrosopumilus Nitrososphaera
Candidatus Cenarchaeum massiliensis	K8823_1214	251	ABC transporter permease	K02050	IPR000515;IPR000515	OG5_129908	Transport	Cenarchaeum Nitrosoarchaeum Nitrosopelagicus Nitrosopumilus Nitrososphaera
Candidatus Cenarchaeum massiliensis	K8823_1215	348	N-acetyl-gamma-glutamyl-phosphate reductase	K00145	IPR000534;IPR000706;IPR000534;IPR012280;IPR000706	OG5_128464	Amino acid metabolism	Cenarchaeum Nitrosoarchaeum Nitrosopelagicus Nitrosopumilus Nitrososphaera
Candidatus Cenarchaeum massiliensis	K8823_1216	267	acetylglutamate kinase	K00930	IPR001048;IPR004662;IPR004662	OG5_130618	Amino acid metabolism	Cenarchaeum Nitrosoarchaeum Nitrosopelagicus Nitrosopumilus Nitrososphaera
Candidatus Cenarchaeum massiliensis	K8823_1217	398	acetylornithine and succinylornithine aminotransferase	K00821	IPR005814;IPR004636;IPR005814;IPR005814;IPR004636	OG5_126982	Amino acid metabolism	Cenarchaeum Nitrosoarchaeum Nitrosopelagicus Nitrosopumilus Nitrososphaera
Candidatus Cenarchaeum massiliensis	K8823_1218	138	AsnC family transcriptional regulator	K06154	IPR019888;IPR019887;IPR000485	OG5_129697	Transcription	Cenarchaeum Nitrosoarchaeum Nitrosopelagicus Nitrosopumilus Nitrososphaera
Candidatus Cenarchaeum massiliensis	K8823_1219	399	homocitrate synthase	K01649	IPR000891;IPR002034;IPR000891	OG5_128072	Misc	Cenarchaeum Nitrosoarchaeum Nitrosopelagicus Nitrosopumilus Nitrososphaera
Candidatus Cenarchaeum massiliensis	K8823_1220	283	lysine biosynthesis enzyme (lysX)	K05827	IPR011870;IPR004666;IPR013651;IPR011761	OG5_130067	Amino acid metabolism	Cenarchaeum Nitrosoarchaeum Nitrosopelagicus Nitrosopumilus Nitrososphaera
Candidatus Cenarchaeum massiliensis	K8823_1221	371	N-acetyl-ornithine/N-acetyl-lysine deacetylase	K05831	IPR010175;IPR002933;IPR011650	OG5_127974	Amino acid metabolism	Cenarchaeum Nitrosoarchaeum Nitrosopelagicus Nitrosopumilus Nitrososphaera
Candidatus Cenarchaeum massiliensis	K8823_1222	348	diphthine synthase	K20215	IPR023140;IPR004551;IPR000878	OG5_127443	Misc	Cenarchaeum Nitrosoarchaeum Nitrosopelagicus Nitrosopumilus Nitrososphaera
Candidatus Cenarchaeum massiliensis	K8823_1224	191	cytidyltransferase-like protein	K14656	IPR004821;IPR004821	OG5_131963	Misc	Cenarchaeum Nitrosoarchaeum Nitrosopelagicus Nitrosopumilus Nitrososphaera
Candidatus Cenarchaeum massiliensis	K8823_1225	229	Riboflavin kinase	K07732	IPR023602	OG5_140211	Metabolism of cofactors and vitamins	Cenarchaeum Nitrosoarchaeum Nitrosopelagicus Nitrosopumilus Nitrososphaera
Candidatus Cenarchaeum massiliensis	K8823_1226	380	DNA primase		IPR006171;IPR006171;IPR006171;IPR020607	OG5_139266	DNA modification	Cenarchaeum Nitrosoarchaeum Nitrosopelagicus Nitrosopumilus Nitrososphaera
Candidatus Cenarchaeum massiliensis	K8823_1227	155	YjgF/chorismate mutase-like domain containing protein		IPR006175	OG5_136477	Misc	Cenarchaeum Nitrosoarchaeum Nitrosopelagicus Nitrosopumilus Nitrososphaera
Candidatus Cenarchaeum massiliensis	K8823_1228	344	phosphoesterase DHHA1		IPR003156	OG5_161654	Misc	Cenarchaeum Nitrosoarchaeum Nitrosopelagicus Nitrosopumilus Nitrososphaera
Candidatus Cenarchaeum massiliensis	K8823_1230	390	citrate synthase II	K01647	IPR011278;IPR002020;IPR002020	OG5_128590	Energy	Cenarchaeum Nitrosoarchaeum Nitrosopelagicus Nitrosopumilus Nitrososphaera
Candidatus Cenarchaeum massiliensis	K8823_1231	94	hypothetical protein			nmar|YP_001582434	Unknown function	Cenarchaeum Nitrosoarchaeum Nitrosopelagicus Nitrosopumilus Nitrososphaera
Candidatus Cenarchaeum massiliensis	K8823_1233	445	MOFRL domain containing protein	K11529	IPR025286;IPR007835	OG5_131197	Amino acid metabolism	Cenarchaeum Nitrosoarchaeum Nitrosopelagicus Nitrosopumilus Nitrososphaera
Candidatus Cenarchaeum massiliensis	K8823_1238	306	Iron-containing alcohol dehydrogenase	K04072	IPR001670	OG5_127140	Oxidation-reduction	Cenarchaeum Nitrosoarchaeum Nitrosopelagicus Nitrosopumilus Nitrososphaera
Candidatus Cenarchaeum massiliensis	K8823_1239	143	SRPBCC domain containing protein		IPR019587	OG5_156550	Stress & defense	Cenarchaeum Nitrosoarchaeum Nitrosopelagicus Nitrosopumilus Nitrososphaera
Candidatus Cenarchaeum massiliensis	K8823_1240	154	DNA-directed RNA polymerase subunit K	K03014	IPR006110;IPR006111;IPR020708;IPR006111	OG5_127345	Transcription	Cenarchaeum Nitrosoarchaeum Nitrosopelagicus Nitrosopumilus Nitrososphaera
Candidatus Cenarchaeum massiliensis	K8823_1242	138	transcriptional regulator	K07108		OG5_142680	Transcription	Cenarchaeum Nitrosoarchaeum Nitrosopelagicus Nitrosopumilus Nitrososphaera
Candidatus Cenarchaeum massiliensis	K8823_1243	327	asparagine synthase	K01953	IPR001962	OG5_127204	Amino acid metabolism	Cenarchaeum Nitrosoarchaeum Nitrosopelagicus Nitrosopumilus Nitrososphaera
Candidatus Cenarchaeum massiliensis	K8823_1246	293	prenyltransferase	K02548	IPR000537	OG5_130024	Metabolism of cofactors and vitamins	Cenarchaeum Nitrosoarchaeum Nitrosopelagicus Nitrosopumilus Nitrososphaera
Candidatus Cenarchaeum massiliensis	K8823_1247	130	hypothetical protein		IPR018699	OG5_175623	Unknown function	Cenarchaeum Nitrosoarchaeum Nitrosopelagicus Nitrosopumilus Nitrososphaera
Candidatus Cenarchaeum massiliensis	K8823_1248	92	peptidyl-prolyl isomerase	K03769	IPR000297;IPR000297	OG5_128509	Protein modification	Cenarchaeum Nitrosoarchaeum Nitrosopelagicus Nitrosopumilus Nitrososphaera
Candidatus Cenarchaeum massiliensis	K8823_115	179	2'-5' RNA ligase	K01975	IPR004175;IPR019510;IPR004175	OG5_132439	RNA modification	Cenarchaeum Nitrosoarchaeum Nitrosopelagicus Nitrosopumilus Nitrososphaera
Candidatus Cenarchaeum massiliensis	K8823_1249	180	helicase	K10896	IPR001650;IPR001650;IPR001650	OG5_128649	DNA modification	Cenarchaeum Nitrosoarchaeum Nitrosopelagicus Nitrosopumilus Nitrososphaera
Candidatus Cenarchaeum massiliensis	K8823_1250	312	DEAD-like helicase	K10896	IPR014001;IPR006935;IPR014001	OG5_128649	DNA modification	Cenarchaeum Nitrosoarchaeum Nitrosopelagicus Nitrosopumilus Nitrososphaera
Candidatus Cenarchaeum massiliensis	K8823_116	446	CCA-adding enzyme	K07558	IPR002934;IPR008229;IPR015329;IPR008229	OG5_139302	RNA modification	Cenarchaeum Nitrosoarchaeum Nitrosopelagicus Nitrosopumilus Nitrososphaera
Candidatus Cenarchaeum massiliensis	K8823_1256	157	hypothetical membrane protein			nmar|YP_001581394	Unknown function	Cenarchaeum Nitrosoarchaeum Nitrosopelagicus Nitrosopumilus Nitrososphaera
Candidatus Cenarchaeum massiliensis	K8823_1257	202	precorrin-6y C5,15-methyltransferase subunit CbiT	K02191	IPR000682;IPR014008;IPR023475	OG5_135539	Metabolism of cofactors and vitamins	Cenarchaeum Nitrosoarchaeum Nitrosopelagicus Nitrosopumilus Nitrososphaera
Candidatus Cenarchaeum massiliensis	K8823_1258	233	Precorrin-2 C20-methyltransferase	K03394	IPR000878;IPR006364	OG5_135387	Metabolism of cofactors and vitamins	Cenarchaeum Nitrosoarchaeum Nitrosopelagicus Nitrosopumilus Nitrososphaera
Candidatus Cenarchaeum massiliensis	K8823_1259	257	Precorrin-4 C11-methyltransferase	K05936	IPR000878;IPR006362	OG5_135370	Metabolism of cofactors and vitamins	Cenarchaeum Nitrosoarchaeum Nitrosopelagicus Nitrosopumilus Nitrososphaera
Candidatus Cenarchaeum massiliensis	K8823_1260	273	Asp/Glu/hydantoin racemase	K01776	IPR015942	OG5_133316	Amino acid metabolism	Cenarchaeum Nitrosoarchaeum Nitrosopelagicus Nitrosopumilus Nitrososphaera

Candidatus Cenarchaeum massiliensisK8823_1261	216	SNF7		IPR005024	OG5_150062	Transport	Cenarchaeum Nitrosoarchaeum Nitrosopelagicus Nitrosopumilus Nitrososphaera
Candidatus Cenarchaeum massiliensisK8823_117	247	serine/threonine protein kinase	K07176	IPR017441	OG5_144423	Signaling	Cenarchaeum Nitrosoarchaeum Nitrosopelagicus Nitrosopumilus Nitrososphaera
Candidatus Cenarchaeum massiliensisK8823_1264	640	hypothetical protein		IPR008532	OG5_127300	Unknown function	Cenarchaeum Nitrosoarchaeum Nitrosopelagicus Nitrosopumilus Nitrososphaera
Candidatus Cenarchaeum massiliensisK8823_1265	213	metal-sulfur cluster biosynthetic enzyme		IPR002744;IPR008792	OG5_132611	Misc	Cenarchaeum Nitrosoarchaeum Nitrosopelagicus Nitrosopumilus Nitrososphaera
Candidatus Cenarchaeum massiliensisK8823_1268	394	aminotransferase		IPR004839;IPR004838	OG5_128985	Amino acid metabolism	Cenarchaeum Nitrosoarchaeum Nitrosopelagicus Nitrosopumilus Nitrososphaera
Candidatus Cenarchaeum massiliensisK8823_1269	131	pyridoxamine 5'-phosphate oxidase		IPR011576	OG5_216789	Oxidation-reduction	Cenarchaeum Nitrosoarchaeum Nitrosopelagicus Nitrosopumilus Nitrososphaera
Candidatus Cenarchaeum massiliensisK8823_1270	77	hypothetical membrane protein			nmar|YP_001581513	Unknown function	Cenarchaeum Nitrosoarchaeum Nitrosopelagicus Nitrosopumilus Nitrososphaera
Candidatus Cenarchaeum massiliensisK8823_1271	223	NADPH-dependent F420 reductase	K06988	IPR028939;IPR010185	OG5_133748	Misc	Cenarchaeum Nitrosoarchaeum Nitrosopelagicus Nitrosopumilus Nitrososphaera
Candidatus Cenarchaeum massiliensisK8823_1272	229	Phosphoglycerate mutase	K15634	IPR013078;IPR013078	OG5_129378	Energy	Cenarchaeum Nitrosoarchaeum Nitrosopelagicus Nitrosopumilus Nitrososphaera
Candidatus Cenarchaeum massiliensisK8823_1273	354	multicopper oxidase	K00368	IPR011706;IPR011707	OG5_141028	Nitrogen metabolism	Cenarchaeum Nitrosoarchaeum Nitrosopelagicus Nitrosopumilus Nitrososphaera
Candidatus Cenarchaeum massiliensisK8823_1274	388	divalent cation transporter			OG5_159190	Transport	Cenarchaeum Nitrosoarchaeum Nitrosopelagicus Nitrosopumilus Nitrososphaera
Candidatus Cenarchaeum massiliensisK8823_1281	126	hypothetical membrane protein			nmar|YP_001583044	Unknown function	Cenarchaeum Nitrosoarchaeum Nitrosopelagicus Nitrosopumilus Nitrososphaera
Candidatus Cenarchaeum massiliensisK8823_1282	511	Protein kinase	K03688	IPR004147;IPR000719	OG5_141142	Protein modification	Cenarchaeum Nitrosoarchaeum Nitrosopelagicus Nitrosopumilus Nitrososphaera
Candidatus Cenarchaeum massiliensisK8823_1283	418	phosphopantothenoylcysteine decarboxylase/phosphopantothenate--cysteine ligase	K13038	IPR007085;IPR003382;IPR005252	OG5_127040	Metabolism of cofactors and vitamins	Cenarchaeum Nitrosoarchaeum Nitrosopelagicus Nitrosopumilus Nitrososphaera
Candidatus Cenarchaeum massiliensisK8823_1284	278	3-methyl-2-oxobutanoate hydroxymethyltransferase	K00606	IPR003700;IPR003700;IPR003700;IPR003700	OG5_129046	Metabolism of cofactors and vitamins	Cenarchaeum Nitrosoarchaeum Nitrosopelagicus Nitrosopumilus Nitrososphaera
Candidatus Cenarchaeum massiliensisK8823_1285	255	hypothetical protein	K09722	IPR002855;IPR002855	OG5_142691	Metabolism of cofactors and vitamins	Cenarchaeum Nitrosoarchaeum Nitrosopelagicus Nitrosopumilus Nitrososphaera
Candidatus Cenarchaeum massiliensisK8823_1286	303	GHMP kinase	K06982	IPR006204	OG5_142698	Metabolism of cofactors and vitamins	Cenarchaeum Nitrosoarchaeum Nitrosopelagicus Nitrosopumilus Nitrososphaera
Candidatus Cenarchaeum massiliensisK8823_1287	325	PhoU family transcriptional regulator		IPR007159;IPR026022	OG5_140165	Misc	Cenarchaeum Nitrosoarchaeum Nitrosopelagicus Nitrosopumilus Nitrososphaera
Candidatus Cenarchaeum massiliensisK8823_120	177	hypothetical membrane protein			OG5_161365	Unknown function	Cenarchaeum Nitrosoarchaeum Nitrosopelagicus Nitrosopumilus Nitrososphaera
Candidatus Cenarchaeum massiliensisK8823_1288	303	malate dehydrogenase	K00024	IPR022383;IPR001236;IPR001557;IPR011275	OG5_126911	Energy	Cenarchaeum Nitrosoarchaeum Nitrosopelagicus Nitrosopumilus Nitrososphaera
Candidatus Cenarchaeum massiliensisK8823_1289	206	hypothetical protein			OG5_182874	Unknown function	Cenarchaeum Nitrosoarchaeum Nitrosopelagicus Nitrosopumilus Nitrososphaera
Candidatus Cenarchaeum massiliensisK8823_1290	78	hypothetical membrane protein			nmar|YP_001581516	Energy	Cenarchaeum Nitrosoarchaeum Nitrosopelagicus Nitrosopumilus Nitrososphaera
Candidatus Cenarchaeum massiliensisK8823_1291	145	Cytochrome c oxidase subunit 2	K02275	IPR002429;IPR001505;IPR002429	OG5_129173	Oxidation-reduction	Cenarchaeum Nitrosoarchaeum Nitrosopelagicus Nitrosopumilus Nitrososphaera
Candidatus Cenarchaeum massiliensisK8823_1292	508	cytochrome c oxidase subunit I	K02274	IPR000883;IPR000883;IPR023616	OG5_128358	Oxidation-reduction	Cenarchaeum Nitrosoarchaeum Nitrosopelagicus Nitrosopumilus Nitrososphaera
Candidatus Cenarchaeum massiliensisK8823_1293	316	Blue (Type1) copper domain-containing protein		IPR000923;IPR028871;IPR002355;IPR028871;IPR002355	OG5_144955	Misc	Cenarchaeum Nitrosoarchaeum Nitrosopelagicus Nitrosopumilus Nitrososphaera
Candidatus Cenarchaeum massiliensisK8823_1294	147	cytochrome oxidase assembly protein	K02259	IPR003780	OG5_144407	Misc	Cenarchaeum Nitrosoarchaeum Nitrosopelagicus Nitrosopumilus Nitrososphaera
Candidatus Cenarchaeum massiliensisK8823_1295	172	hypothetical membrane protein			nmar|YP_001581521	Unknown function	Cenarchaeum Nitrosoarchaeum Nitrosopelagicus Nitrosopumilus Nitrososphaera
Candidatus Cenarchaeum massiliensisK8823_1296	363	Zn-dependent oxidoreductase		IPR020843;IPR013149;IPR013154;IPR002085	OG5_129009	Oxidation-reduction	Cenarchaeum Nitrosoarchaeum Nitrosopelagicus Nitrosopumilus Nitrososphaera
Candidatus Cenarchaeum massiliensisK8823_1297	285	Citrate lyase	K01644	IPR005000	OG5_130136	Energy	Cenarchaeum Nitrosoarchaeum Nitrosopelagicus Nitrosopumilus Nitrososphaera
Candidatus Cenarchaeum massiliensisK8823_121	440	Thif family protein	K21029	IPR000594;IPR003749	OG5_126966	Sulfur metabolism	Cenarchaeum Nitrosoarchaeum Nitrosopelagicus Nitrosopumilus Nitrososphaera
Candidatus Cenarchaeum massiliensisK8823_1298	491	glycine--tRNA ligase	K01880	IPR002315;IPR004154;IPR002314;IPR027031;IPR006195	OG5_127272	Translation	Cenarchaeum Nitrosoarchaeum Nitrosopelagicus Nitrosopumilus Nitrososphaera
Candidatus Cenarchaeum massiliensisK8823_1299	280	endonuclease 4	K01151	IPR001719;IPR013022;IPR001719;IPR001719;IPR018246;IPR018246;IPR001719;IPR001719	OG5_129467	DNA modification	Cenarchaeum Nitrosoarchaeum Nitrosopelagicus Nitrosopumilus Nitrososphaera
Candidatus Cenarchaeum massiliensisK8823_1300	111	hypothetical protein			OG5_172013	Unknown function	Cenarchaeum Nitrosoarchaeum Nitrosopelagicus Nitrosopumilus Nitrososphaera
Candidatus Cenarchaeum massiliensisK8823_1301	319	ATP phosphoribosyltransferase	K00765	IPR013115;IPR013820;IPR013820;IPR013115;IPR001348	OG5_129102	Amino acid metabolism	Cenarchaeum Nitrosoarchaeum Nitrosopelagicus Nitrosopumilus Nitrososphaera
Candidatus Cenarchaeum massiliensisK8823_1302	282	histidinol dehydrogenase	K00013	IPR012131;IPR012131;IPR001692	OG5_129099	Amino acid metabolism	Cenarchaeum Nitrosoarchaeum Nitrosopelagicus Nitrosopumilus Nitrososphaera
Candidatus Cenarchaeum massiliensisK8823_1303	122	histidinol dehydrogenase	K00013	IPR012131	OG5_129099	Amino acid metabolism	Cenarchaeum Nitrosoarchaeum Nitrosopelagicus Nitrosopumilus Nitrososphaera
Candidatus Cenarchaeum massiliensisK8823_1304	355	aminotransferase	K00817	IPR004839;IPR001917	OG5_127656	Amino acid metabolism	Cenarchaeum Nitrosoarchaeum Nitrosopelagicus Nitrosopumilus Nitrososphaera
Candidatus Cenarchaeum massiliensisK8823_1305	326	phosphatase		IPR023214	OG5_182754	Misc	Cenarchaeum Nitrosoarchaeum Nitrosopelagicus Nitrosopumilus Nitrososphaera
Candidatus Cenarchaeum massiliensisK8823_122	411	threonine synthase	K01733	IPR004450;IPR001926	OG5_130923	Amino acid metabolism	Cenarchaeum Nitrosoarchaeum Nitrosopelagicus Nitrosopumilus Nitrososphaera
Candidatus Cenarchaeum massiliensisK8823_1307	392	translation-associated GTPase	K06942	IPR006073;IPR004095;IPR013646;IPR031167	OG5_130225	Misc	Cenarchaeum Nitrosoarchaeum Nitrosopelagicus Nitrosopumilus Nitrososphaera
Candidatus Cenarchaeum massiliensisK8823_1308	307	Kae1	K01409	IPR000905;IPR017861;IPR017860	OG5_126710	Protein modification	Cenarchaeum Nitrosoarchaeum Nitrosopelagicus Nitrosopumilus Nitrososphaera
Candidatus Cenarchaeum massiliensisK8823_1309	105	hypothetical protein			nmar|YP_001582866	Unknown function	Cenarchaeum Nitrosoarchaeum Nitrosopelagicus Nitrosopumilus Nitrososphaera
Candidatus Cenarchaeum massiliensisK8823_1310	171	Transcription initiation factor IIE	K03136	IPR002853;IPR024550;IPR017919;IPR016481	OG5_140198	Transcription	Cenarchaeum Nitrosoarchaeum Nitrosopelagicus Nitrosopumilus Nitrososphaera
Candidatus Cenarchaeum massiliensisK8823_1311	179	tRNA methyltransferase	K07254	IPR002845;IPR002845;IPR002845	OG5_142690	RNA modification	Cenarchaeum Nitrosoarchaeum Nitrosopelagicus Nitrosopumilus Nitrososphaera
Candidatus Cenarchaeum massiliensisK8823_1312	346	GTPase	K03665	IPR006073;IPR016496;IPR025121;IPR016496;IPR030394;IPR016496	OG5_128084	Misc	Cenarchaeum Nitrosoarchaeum Nitrosopelagicus Nitrosopumilus Nitrososphaera
Candidatus Cenarchaeum massiliensisK8823_1313	344	PhoU family transcriptional regulator		IPR007159;IPR007159;IPR026022	OG5_140165	Misc	Cenarchaeum Nitrosoarchaeum Nitrosopelagicus Nitrosopumilus Nitrososphaera
Candidatus Cenarchaeum massiliensisK8823_1314	213	NAD(P)H-hydrate epimerase	K17759	IPR004443;IPR004443;IPR004443;IPR004443	OG5_128872	Misc	Cenarchaeum Nitrosoarchaeum Nitrosopelagicus Nitrosopumilus Nitrososphaera
Candidatus Cenarchaeum massiliensisK8823_1315	203	metallohydrolase	K17725	IPR001279;IPR001279	OG5_127147	Misc	Cenarchaeum Nitrosoarchaeum Nitrosopelagicus Nitrosopumilus Nitrososphaera
Candidatus Cenarchaeum massiliensisK8823_1316	351	hypothetical protein		IPR002805;IPR003200;IPR002805	OG5_146669	Metabolism of cofactors and vitamins	Cenarchaeum Nitrosoarchaeum Nitrosopelagicus Nitrosopumilus Nitrososphaera
Candidatus Cenarchaeum massiliensisK8823_1317	581	glutamine-fructose-6-phosphate transaminase	K00820	IPR005855;IPR001347;IPR005855;IPR001347;IPR001347;IPR017932	OG5_126842	Amino acid metabolism	Cenarchaeum Nitrosoarchaeum Nitrosopelagicus Nitrosopumilus Nitrososphaera
Candidatus Cenarchaeum massiliensisK8823_1318	222	ribosomal protein S4	K02997	IPR002942;IPR002942;IPR005710;IPR022801;IPR002942	OG5_126983	Translation	Cenarchaeum Nitrosoarchaeum Nitrosopelagicus Nitrosopumilus Nitrososphaera
Candidatus Cenarchaeum massiliensisK8823_1319	189	ribosomal protein S13	K02964	IPR001892;IPR018269;IPR001892;IPR001892	OG5_126668	Translation	Cenarchaeum Nitrosoarchaeum Nitrosopelagicus Nitrosopumilus Nitrososphaera
Candidatus Cenarchaeum massiliensisK8823_1323	197	cyclase		IPR019587	nmar|YP_001581522	Stress & defense	Cenarchaeum Nitrosoarchaeum Nitrosopelagicus Nitrosopumilus Nitrososphaera
Candidatus Cenarchaeum massiliensisK8823_1326	365	cobalt-precorrin-6A synthase	K02188	IPR002748;IPR002748;IPR002748	OG5_136503	Metabolism of cofactors and vitamins	Cenarchaeum Nitrosoarchaeum Nitrosopelagicus Nitrosopumilus Nitrososphaera
Candidatus Cenarchaeum massiliensisK8823_124	191	orotate phosphoribosyltransferase	K00762	IPR023031	OG5_126793	Nucleotide metabolism	Cenarchaeum Nitrosoarchaeum Nitrosopelagicus Nitrosopumilus Nitrososphaera
Candidatus Cenarchaeum massiliensisK8823_1327	350	cobalamin biosynthesis protein (CbiG)	K02189	IPR021745;IPR002750;IPR021744	OG5_137381	Metabolism of cofactors and vitamins	Cenarchaeum Nitrosoarchaeum Nitrosopelagicus Nitrosopumilus Nitrososphaera
Candidatus Cenarchaeum massiliensisK8823_1328	251	cobalamin biosynthesis CbiX protein	K03795	IPR002762	OG5_133515	Metabolism of cofactors and vitamins	Cenarchaeum Nitrosoarchaeum Nitrosopelagicus Nitrosopumilus Nitrososphaera
Candidatus Cenarchaeum massiliensisK8823_1329	207	precorrin-8X methylmutase (cobH, cbiC)	K06042	IPR003722	OG5_136504	Metabolism of cofactors and vitamins	Cenarchaeum Nitrosoarchaeum Nitrosopelagicus Nitrosopumilus Nitrososphaera
Candidatus Cenarchaeum massiliensisK8823_1330	448	cobyrinic acid a,c-diamide synthase	K02224	IPR011698;IPR002586;IPR004484;IPR004484;IPR017929	OG5_132745	Metabolism of cofactors and vitamins	Cenarchaeum Nitrosoarchaeum Nitrosopelagicus Nitrosopumilus Nitrososphaera
Candidatus Cenarchaeum massiliensisK8823_125	108	phosphoribosyl-AMP cyclohydrolase (hisIE)	K11755	IPR002496;IPR002496;IPR026660	OG5_131046	Amino acid metabolism	Cenarchaeum Nitrosoarchaeum Nitrosopelagicus Nitrosopumilus Nitrososphaera
Candidatus Cenarchaeum massiliensisK8823_1335	185	Transferase		IPR001451;IPR018357	OG5_128273	Amino acid metabolism	Cenarchaeum Nitrosoarchaeum Nitrosopelagicus Nitrosopumilus Nitrososphaera
Candidatus Cenarchaeum massiliensisK8823_1336	304	NAD-dependent epimerase	K01784	IPR001509	OG5_126853	Carbohydrate metabolism	Cenarchaeum Nitrosoarchaeum Nitrosopelagicus Nitrosopumilus Nitrososphaera
Candidatus Cenarchaeum massiliensisK8823_1337	178	Cob(I)alamin adenosyltransferase (cobO, btuR)	K19221	IPR003724;IPR003724	OG5_134037	Metabolism of cofactors and vitamins	Cenarchaeum Nitrosoarchaeum Nitrosopelagicus Nitrosopumilus Nitrososphaera
Candidatus Cenarchaeum massiliensisK8823_1339	261	translation initiation factor IF-2 subunit alpha	K03237	IPR022967;IPR003029;IPR011488;IPR003029	OG5_127433	Translation	Cenarchaeum Nitrosoarchaeum Nitrosopelagicus Nitrosopumilus Nitrososphaera
Candidatus Cenarchaeum massiliensisK8823_1340	51	ribosome biogenesis protein		IPR007264	OG5_127952	Translation	Cenarchaeum Nitrosoarchaeum Nitrosopelagicus Nitrosopumilus Nitrososphaera
Candidatus Cenarchaeum massiliensisK8823_1341	866	Membrane protein required for N-linked glycosylation	K03768	IPR002130;IPR003674;IPR024936;IPR002130	OG5_126840	Protein modification	Cenarchaeum Nitrosoarchaeum Nitrosopelagicus Nitrosopumilus Nitrososphaera
Candidatus Cenarchaeum massiliensisK8823_1342	339	hypothetical protein	K09726	IPR007152	OG5_157234	Lipid metabolism	Cenarchaeum Nitrosoarchaeum Nitrosopelagicus Nitrosopumilus Nitrososphaera
Candidatus Cenarchaeum massiliensisK8823_1343	333	glycosyl transferase family 4	K01001	IPR000715;IPR000715	OG5_131597	Misc	Cenarchaeum Nitrosoarchaeum Nitrosopelagicus Nitrosopumilus Nitrososphaera
Candidatus Cenarchaeum massiliensisK8823_126	266	Imidazole glycerol phosphate synthase subunit HisF	K02500	IPR006062;IPR004651;IPR004651	OG5_129302	Amino acid metabolism	Cenarchaeum Nitrosoarchaeum Nitrosopelagicus Nitrosopumilus Nitrososphaera
Candidatus Cenarchaeum massiliensisK8823_1346	388	S-adenosylmethionine synthetase	K00789	IPR022630;IPR022628;IPR022629;IPR002133;IPR002133;IPR022631;IPR022631;IPR002133	OG5_126734	Amino acid metabolism	Cenarchaeum Nitrosoarchaeum Nitrosopelagicus Nitrosopumilus Nitrososphaera
Candidatus Cenarchaeum massiliensisK8823_1347	84	snRNP Sm-like protein	K04796	IPR001163;IPR001163	OG5_128064	Misc	Cenarchaeum Nitrosoarchaeum Nitrosopelagicus Nitrosopumilus Nitrososphaera
Candidatus Cenarchaeum massiliensisK8823_1348	217	recombinase RecA	K04482	IPR013632;IPR020588	OG5_135350	DNA modification	Cenarchaeum Nitrosoarchaeum Nitrosopelagicus Nitrosopumilus Nitrososphaera
Candidatus Cenarchaeum massiliensisK8823_1349	911	DEAD/DEAH box helicase	K03724	IPR014001;IPR001650;IPR011545;IPR013701;IPR001650;IPR001650;IPR014001	OG5_132107	DNA modification	Cenarchaeum Nitrosoarchaeum Nitrosopelagicus Nitrosopumilus Nitrososphaera
Candidatus Cenarchaeum massiliensisK8823_1350	468	nucleic acid binding-protein			OG5_140194	DNA modification	Cenarchaeum Nitrosoarchaeum Nitrosopelagicus Nitrosopumilus Nitrososphaera
Candidatus Cenarchaeum massiliensisK8823_1351	379	hypothetical protein			nmar|YP_001581432	Unknown function	Cenarchaeum Nitrosoarchaeum Nitrosopelagicus Nitrosopumilus Nitrososphaera
Candidatus Cenarchaeum massiliensisK8823_1352	186	GrpE protein	K03687	IPR000740;IPR000740;IPR000740;IPR000740	OG5_126978	Protein modification	Cenarchaeum Nitrosoarchaeum Nitrosopelagicus Nitrosopumilus Nitrososphaera
Candidatus Cenarchaeum massiliensisK8823_1353	652	chaperone protein DnaK	K04043	IPR013126;IPR018181;IPR018181;IPR012725	OG5_126744	Protein modification	Cenarchaeum Nitrosoarchaeum Nitrosopelagicus Nitrosopumilus Nitrososphaera
Candidatus Cenarchaeum massiliensisK8823_127	62	1-(5-phosphoribosyl)-5-imidazole-4-carboxamide isomerase		IPR006062	OG5_132144	Amino acid metabolism	Cenarchaeum Nitrosoarchaeum Nitrosopelagicus Nitrosopumilus Nitrososphaera
Candidatus Cenarchaeum massiliensisK8823_1354	355	chaperone protein DnaJ	K03686	IPR001623;IPR001305;IPR001623;IPR002939;IPR018253;IPR012724;IPR001623;IPR001305	OG5_126952	Protein modification	Cenarchaeum Nitrosoarchaeum Nitrosopelagicus Nitrosopumilus Nitrososphaera
Candidatus Cenarchaeum massiliensisK8823_1355	61	hypothetical protein			STG_0031	Unknown function	Cenarchaeum Nitrosoarchaeum Nitrosopelagicus Nitrosopumilus Nitrososphaera
Candidatus Cenarchaeum massiliensisK8823_1357	635	glutamyl-tRNA(Gln) amidotransferase subunit E	K03330	IPR018027;IPR006075;IPR004414;IPR018027;IPR017959;IPR017959;IPR017959;IPR004414	OG5_139252	Translation	Cenarchaeum Nitrosoarchaeum Nitrosopelagicus Nitrosopumilus Nitrososphaera
Candidatus Cenarchaeum massiliensisK8823_1358	307	oxidoreductase domain-containing protein	K03810	IPR000683;IPR004104	OG5_128146	Oxidation-reduction	Cenarchaeum Nitrosoarchaeum Nitrosopelagicus Nitrosopumilus Nitrososphaera
Candidatus Cenarchaeum massiliensisK8823_1359	318	hypothetical exported protein			OG5_176014	Cell & development	Cenarchaeum Nitrosoarchaeum Nitrosopelagicus Nitrosopumilus Nitrososphaera
Candidatus Cenarchaeum massiliensisK8823_1360	153	hypothetical protein			nmar|YP_001581448	Unknown function	Cenarchaeum Nitrosoarchaeum Nitrosopelagicus Nitrosopumilus Nitrososphaera
Candidatus Cenarchaeum massiliensisK8823_1361	348	eRF1 domain-containing protein	K06965	IPR005142;IPR005140;IPR004405	OG5_127513	Translation	Cenarchaeum Nitrosoarchaeum Nitrosopelagicus Nitrosopumilus Nitrososphaera
Candidatus Cenarchaeum massiliensisK8823_1362	115	Divalent ion tolerance protein CutA1	K03926	IPR004323;IPR004323	OG5_128186	Stress & defense	Cenarchaeum Nitrosoarchaeum Nitrosopelagicus Nitrosopumilus Nitrososphaera
Candidatus Cenarchaeum massiliensisK8823_1363	398	peptidase M50		IPR001478;IPR001478;IPR008915;IPR001193	OG5_130354	Protein modification	Cenarchaeum Nitrosoarchaeum Nitrosopelagicus Nitrosopumilus Nitrososphaera
Candidatus Cenarchaeum massiliensisK8823_128	163	Histidine biosynthesis protein HisA	K01814	IPR006062	OG5_132144	Amino acid metabolism	Cenarchaeum Nitrosoarchaeum Nitrosopelagicus Nitrosopumilus Nitrososphaera
Candidatus Cenarchaeum massiliensisK8823_1364	301	PP-loop domain-containing protein	K14168	IPR011063;IPR000541	OG5_127415	Translation	Cenarchaeum Nitrosoarchaeum Nitrosopelagicus Nitrosopumilus Nitrososphaera
Candidatus Cenarchaeum massiliensisK8823_1365	94	acylphosphatase	K01512	IPR001792;IPR017968;IPR017968;IPR001792	OG5_127552	Pyruvate metabolism	Cenarchaeum Nitrosoarchaeum Nitrosopelagicus Nitrosopumilus Nitrososphaera
Candidatus Cenarchaeum massiliensisK8823_1366	266	ribosomal protein L2	K02886	IPR022666;IPR022669;IPR002171;IPR023672	OG5_126641	Translation	Cenarchaeum Nitrosoarchaeum Nitrosopelagicus Nitrosopumilus Nitrososphaera
Candidatus Cenarchaeum massiliensisK8823_1367	713	Cell division cycle protein 48-like protein	K13525	IPR003338;IPR004201;IPR003593;IPR003959;IPR003338;IPR005938;IPR004201;IPR003960;IPR003960	OG5_126926	Cell & development	Cenarchaeum Nitrosoarchaeum Nitrosopelagicus Nitrosopumilus Nitrososphaera
Candidatus Cenarchaeum massiliensisK8823_1368	314	glycosyl transferase	K00743	IPR001296	OG5_127105	Lipid metabolism	Cenarchaeum Nitrosoarchaeum Nitrosopelagicus Nitrosopumilus Nitrososphaera
Candidatus Cenarchaeum massiliensisK8823_1369	449	UDP-glucose/GDP-mannose dehydrogenase	K02472	IPR014027;IPR014027;IPR017476;IPR014026;IPR001732;IPR017476	OG5_126979	Amino sugar and nucleotide sugar metabolism	Cenarchaeum Nitrosoarchaeum Nitrosopelagicus Nitrosopumilus Nitrososphaera
Candidatus Cenarchaeum massiliensisK8823_1370	406	asparagine synthase	K01953	IPR001962	OG5_127204	Amino acid metabolism	Cenarchaeum Nitrosoarchaeum Nitrosopelagicus Nitrosopumilus Nitrososphaera
Candidatus Cenarchaeum massiliensisK8823_1371	350	glycosyl transferase family 1		IPR001296	OG5_127105	Lipid metabolism	Cenarchaeum Nitrosoarchaeum Nitrosopelagicus Nitrosopumilus Nitrososphaera
Candidatus Cenarchaeum massiliensisK8823_1372	424	glutamyl-tRNA(Gln) amidotransferase subunit D	K09482	IPR006034;IPR011878;IPR006033;IPR006034;IPR027475;IPR006034	OG5_127035	Translation	Cenarchaeum Nitrosoarchaeum Nitrosopelagicus Nitrosopumilus Nitrososphaera
Candidatus Cenarchaeum massiliensisK8823_129	203	imidazole glycerol phosphate synthase subunit HisH	K02501	IPR010139;IPR017926;IPR017926;IPR010139	OG5_131888	Amino acid metabolism	Cenarchaeum Nitrosoarchaeum Nitrosopelagicus Nitrosopumilus Nitrososphaera

Candidatus Cenarchaeum massiliensisK8823_1373	139	hypothetical protein		IPR001602;IPR001602	OG5_128277	Unknown function	Cenarchaeum Nitrosoarchaeum Nitrosopelagicus Nitrosopumilus Nitrososphaera
Candidatus Cenarchaeum massiliensisK8823_1375	150	transcriptional regulator	K03709	IPR022689;IPR022687;IPR001367;IPR022687	OG5_132027	Transcription	Cenarchaeum Nitrosoarchaeum Nitrosopelagicus Nitrosopumilus Nitrososphaera
Candidatus Cenarchaeum massiliensisK8823_1376	267	transcriptional regulator TrmB		IPR002831	OG5_134246	Transcription	Cenarchaeum Nitrosoarchaeum Nitrosopelagicus Nitrosopumilus Nitrososphaera
Candidatus Cenarchaeum massiliensisK8823_1377	541	radical SAM domain protein	K06937	IPR007197	OG5_140187	Oxidation-reduction	Cenarchaeum Nitrosoarchaeum Nitrosopelagicus Nitrosopumilus Nitrososphaera
Candidatus Cenarchaeum massiliensisK8823_1378	304	branched chain amino acid aminotransferase	K00826	IPR001544;IPR005785;IPR001544;IPR018300	OG5_126731	Amino acid metabolism	Cenarchaeum Nitrosoarchaeum Nitrosopelagicus Nitrosopumilus Nitrososphaera
Candidatus Cenarchaeum massiliensisK8823_130	198	imidazoleglycerol-phosphate dehydratase	K01693	IPR000807;IPR000807;IPR000807	OG5_129258	Amino acid metabolism	Cenarchaeum Nitrosoarchaeum Nitrosopelagicus Nitrosopumilus Nitrososphaera
Candidatus Cenarchaeum massiliensisK8823_1380	116	hypothetical protein			nmar|YP_001582875	Unknown function	Cenarchaeum Nitrosoarchaeum Nitrosopelagicus Nitrosopumilus Nitrososphaera
Candidatus Cenarchaeum massiliensisK8823_1381	155	copper-binding protein		IPR000923	OG5_241580	Misc	Cenarchaeum Nitrosoarchaeum Nitrosopelagicus Nitrosopumilus Nitrososphaera
Candidatus Cenarchaeum massiliensisK8823_1386	270	DNA methylase	K13581	IPR002941;IPR002052	OG5_135744	DNA modification	Cenarchaeum Nitrosoarchaeum Nitrosopelagicus Nitrosopumilus Nitrososphaera
Candidatus Cenarchaeum massiliensisK8823_1389	346	S-methyl-5-thioribose-1-phosphate isomerase	K08963	IPR011559;IPR005251;IPR000649;IPR005251	OG5_127565	Translation	Cenarchaeum Nitrosoarchaeum Nitrosopelagicus Nitrosopumilus Nitrososphaera
Candidatus Cenarchaeum massiliensisK8823_1390	259	NAD(+) synthetase	K01916	IPR003694;IPR022310;IPR022926	OG5_127076	Metabolism of cofactors and vitamins	Cenarchaeum Nitrosoarchaeum Nitrosopelagicus Nitrosopumilus Nitrososphaera
Candidatus Cenarchaeum massiliensisK8823_1391	478	amino acid kinase	K00928	IPR001048	nmar|YP_001581527	Amino acid metabolism	Cenarchaeum Nitrosoarchaeum Nitrosopelagicus Nitrosopumilus Nitrososphaera
Candidatus Cenarchaeum massiliensisK8823_131	347	Membrane protein		IPR022791;IPR022791	OG5_141286	Unknown function	Cenarchaeum Nitrosoarchaeum Nitrosopelagicus Nitrosopumilus Nitrososphaera
Candidatus Cenarchaeum massiliensisK8823_1399	363	pyridoxal-5'-phosphate-dependent protein	K13010	IPR000653	OG5_129403	Amino sugar and nucleotide sugar metabolism	Cenarchaeum Nitrosoarchaeum Nitrosopelagicus Nitrosopumilus Nitrososphaera
Candidatus Cenarchaeum massiliensisK8823_1401	186	TATA box-binding protein	K03120	IPR000814;IPR000814;IPR030491;IPR030491;IPR000814	OG5_126912	Transcription	Cenarchaeum Nitrosoarchaeum Nitrosopelagicus Nitrosopumilus Nitrososphaera
Candidatus Cenarchaeum massiliensisK8823_132	252	GTPase	K06883	IPR004130;IPR030230;IPR004130	OG5_127563	Signaling	Cenarchaeum Nitrosoarchaeum Nitrosopelagicus Nitrosopumilus Nitrososphaera
Candidatus Cenarchaeum massiliensisK8823_1404	324	protoheme IX farnesyltransferase	K02301	IPR006369;IPR000537;IPR006369	OG5_127401	Metabolism of cofactors and vitamins	Cenarchaeum Nitrosoarchaeum Nitrosopelagicus Nitrosopumilus Nitrososphaera
Candidatus Cenarchaeum massiliensisK8823_1405	152	transcriptional regulator	K03718	IPR019888;IPR019887;IPR000485	OG5_129697	Transcription	Cenarchaeum Nitrosoarchaeum Nitrosopelagicus Nitrosopumilus Nitrososphaera
Candidatus Cenarchaeum massiliensisK8823_1406	359	semialdehyde dehydrogenase	K00133	IPR000534;IPR012280;IPR005676;IPR000534;IPR000319	OG5_128664	Amino acid metabolism	Cenarchaeum Nitrosoarchaeum Nitrosopelagicus Nitrosopumilus Nitrososphaera
Candidatus Cenarchaeum massiliensisK8823_1407	298	radical SAM protein		IPR006638;IPR007197;IPR023970	OG5_163299	Misc	Cenarchaeum Nitrosoarchaeum Nitrosopelagicus Nitrosopumilus Nitrososphaera
Candidatus Cenarchaeum massiliensisK8823_133	311	homoserine kinase	K00872	IPR013750;IPR006204;IPR000870;IPR006203;IPR000870	OG5_129171	Amino acid metabolism	Cenarchaeum Nitrosoarchaeum Nitrosopelagicus Nitrosopumilus Nitrososphaera
Candidatus Cenarchaeum massiliensisK8823_1415	677	glycosyl transferase	K13680		OG5_132020	Misc	Cenarchaeum Nitrosoarchaeum Nitrosopelagicus Nitrosopumilus Nitrososphaera
Candidatus Cenarchaeum massiliensisK8823_1416	307	ferredoxin		IPR017896;IPR017900;IPR017896;IPR017896	OG5_142686	Misc	Cenarchaeum Nitrosoarchaeum Nitrosopelagicus Nitrosopumilus Nitrososphaera
Candidatus Cenarchaeum massiliensisK8823_134	224	7-cyano-7-deazaguanine synthase	K06920	IPR018317	OG5_132019	Misc	Cenarchaeum Nitrosoarchaeum Nitrosopelagicus Nitrosopumilus Nitrososphaera
Candidatus Cenarchaeum massiliensisK8823_1419	271	ATP-NAD kinase	K00858	IPR002504;IPR002504;IPR002504	OG5_127041	Metabolism of cofactors and vitamins	Cenarchaeum Nitrosoarchaeum Nitrosopelagicus Nitrosopumilus Nitrososphaera
Candidatus Cenarchaeum massiliensisK8823_1420	161	nucleotide-binding protein	K07060		OG5_137279	RNA modification	Cenarchaeum Nitrosoarchaeum Nitrosopelagicus Nitrosopumilus Nitrososphaera
Candidatus Cenarchaeum massiliensisK8823_1421	251	rhodanese-like protein	K01011	IPR001763;IPR001763;IPR001763;IPR001763	OG5_127129	Sulfur metabolism	Cenarchaeum Nitrosoarchaeum Nitrosopelagicus Nitrosopumilus Nitrososphaera
Candidatus Cenarchaeum massiliensisK8823_1422	227	ERCC4 domain protein	K10848	IPR006166;IPR003583;IPR006166	OG5_127977	DNA modification	Cenarchaeum Nitrosoarchaeum Nitrosopelagicus Nitrosopumilus Nitrososphaera
Candidatus Cenarchaeum massiliensisK8823_1423	126	Prefoldin beta subunit	K04798	IPR012713;IPR002777;IPR012713	OG5_127549	Protein modification	Cenarchaeum Nitrosoarchaeum Nitrosopelagicus Nitrosopumilus Nitrososphaera
Candidatus Cenarchaeum massiliensisK8823_1425	69	ribosomal protein L37ae		IPR002674	OG5_127166	Translation	Cenarchaeum Nitrosoarchaeum Nitrosopelagicus Nitrosopumilus Nitrososphaera
Candidatus Cenarchaeum massiliensisK8823_1426	272	RNA-binding protein	K12589	IPR015847;IPR001247	OG5_126882	RNA modification	Cenarchaeum Nitrosoarchaeum Nitrosopelagicus Nitrosopumilus Nitrososphaera
Candidatus Cenarchaeum massiliensisK8823_1427	245	RNase PH, exosome complex component RRP41	K11600	IPR015847;IPR011807;IPR001247;IPR011807	OG5_127234	RNA modification	Cenarchaeum Nitrosoarchaeum Nitrosopelagicus Nitrosopumilus Nitrososphaera
Candidatus Cenarchaeum massiliensisK8823_1428	223	exosome complex RNA-binding protein Rrp4	K03679	IPR004087;IPR022967;IPR004088;IPR026699;IPR003029;IPR004088;IPR023474	OG5_127653	RNA modification	Cenarchaeum Nitrosoarchaeum Nitrosopelagicus Nitrosopumilus Nitrososphaera
Candidatus Cenarchaeum massiliensisK8823_1429	228	rRNA metabolism protein	K14574	IPR002140;IPR018978;IPR019783;IPR002140	OG5_127367	Translation	Cenarchaeum Nitrosoarchaeum Nitrosopelagicus Nitrosopumilus Nitrososphaera
Candidatus Cenarchaeum massiliensisK8823_1430	217	2-phospho-L-lactate guanylyltransferase (cofC)	K14941	IPR002835;IPR002835	OG5_159053	Methane metabolism	Cenarchaeum Nitrosoarchaeum Nitrosopelagicus Nitrosopumilus Nitrososphaera
Candidatus Cenarchaeum massiliensisK8823_1431	306	2-phospho-L-lactate transferase (cofD)	K11212	IPR010115;IPR002882;IPR010115	OG5_152544	Methane metabolism	Cenarchaeum Nitrosoarchaeum Nitrosopelagicus Nitrosopumilus Nitrososphaera
Candidatus Cenarchaeum massiliensisK8823_1432	429	hypothetical exported protein			nmar|YP_001581961	Unknown function	Cenarchaeum Nitrosoarchaeum Nitrosopelagicus Nitrosopumilus Nitrososphaera
Candidatus Cenarchaeum massiliensisK8823_1435	271	hypothetical protein			OG5_241568	Unknown function	Cenarchaeum Nitrosoarchaeum Nitrosopelagicus Nitrosopumilus Nitrososphaera
Candidatus Cenarchaeum massiliensisK8823_1436	631	Acetyl-coenzyme A synthetase	K01895	IPR000873;IPR011904;IPR025110;IPR020845	OG5_126680	Energy	Cenarchaeum Nitrosoarchaeum Nitrosopelagicus Nitrosopumilus Nitrososphaera
Candidatus Cenarchaeum massiliensisK8823_136	188	GTP cyclohydrolase I	K01495	IPR001474;IPR020602;IPR001474;IPR018234;IPR018234;IPR001474	OG5_127507	Metabolism of cofactors and vitamins	Cenarchaeum Nitrosoarchaeum Nitrosopelagicus Nitrosopumilus Nitrososphaera
Candidatus Cenarchaeum massiliensisK8823_1437	261	hypothetical protein			OG5_160272	Cell & development	Cenarchaeum Nitrosoarchaeum Nitrosopelagicus Nitrosopumilus Nitrososphaera
Candidatus Cenarchaeum massiliensisK8823_1439	550	thymidylate synthase ThyX		IPR003669;IPR003669;IPR003669	OG5_193908	Nucleotide metabolism	Cenarchaeum Nitrosoarchaeum Nitrosopelagicus Nitrosopumilus Nitrososphaera
Candidatus Cenarchaeum massiliensisK8823_1440	127	methionine-R-sulfoxide reductase	K07305	IPR002579;IPR002579;IPR028427	OG5_127019	Oxidation-reduction	Cenarchaeum Nitrosoarchaeum Nitrosopelagicus Nitrosopumilus Nitrososphaera
Candidatus Cenarchaeum massiliensisK8823_1441	339	homoserine dehydrogenase	K00003	IPR001342;IPR005106	OG5_128063	Amino acid metabolism	Cenarchaeum Nitrosoarchaeum Nitrosopelagicus Nitrosopumilus Nitrososphaera
Candidatus Cenarchaeum massiliensisK8823_1442	159	RNA-binding protein	K07575	IPR002478;IPR002478;IPR004521;IPR016437;IPR002478	OG5_127512	RNA modification	Cenarchaeum Nitrosoarchaeum Nitrosopelagicus Nitrosopumilus Nitrososphaera
Candidatus Cenarchaeum massiliensisK8823_1443	195	proteasome endopeptidase complex subunit (psmB, prcB)	K03433	IPR001353;IPR016050;IPR023333;IPR019983	OG5_152189	Protein modification	Cenarchaeum Nitrosoarchaeum Nitrosopelagicus Nitrosopumilus Nitrososphaera
Candidatus Cenarchaeum massiliensisK8823_1445	447	Peptidase M20	K01439	IPR011650;IPR002933	OG5_127342	Amino acid metabolism	Cenarchaeum Nitrosoarchaeum Nitrosopelagicus Nitrosopumilus Nitrososphaera
Candidatus Cenarchaeum massiliensisK8823_1446	290	carbohydrate kinase	K17758	IPR000631;IPR000631;IPR000631;IPR000631	OG5_127151	Metabolism of cofactors and vitamins	Cenarchaeum Nitrosoarchaeum Nitrosopelagicus Nitrosopumilus Nitrososphaera
Candidatus Cenarchaeum massiliensisK8823_137	237	organic radical-activating protein		IPR024924	OG5_132584	Misc	Cenarchaeum Nitrosoarchaeum Nitrosopelagicus Nitrosopumilus Nitrososphaera
Candidatus Cenarchaeum massiliensisK8823_1447	281	tRNA methyltransferase complex GCD14 subunit	K07442	IPR014816;IPR014816	OG5_127534	Translation	Cenarchaeum Nitrosoarchaeum Nitrosopelagicus Nitrosopumilus Nitrososphaera
Candidatus Cenarchaeum massiliensisK8823_1448	624	arginine--tRNA ligase	K01887	IPR005148;IPR008909;IPR008909;IPR005148;IPR001278;IPR001278;IPR001278;IPR001412	OG5_126752	Translation	Cenarchaeum Nitrosoarchaeum Nitrosopelagicus Nitrosopumilus Nitrososphaera
Candidatus Cenarchaeum massiliensisK8823_1449	233	phosphoglycolate phosphatase		IPR006382;IPR023214;IPR006378;IPR006379	OG5_127435	Energy	Cenarchaeum Nitrosoarchaeum Nitrosopelagicus Nitrosopumilus Nitrososphaera
Candidatus Cenarchaeum massiliensisK8823_1450	367	small GTP-binding protein	K06944	IPR006073;IPR004095;IPR005225;IPR006074;IPR031167	OG5_127528	Signaling	Cenarchaeum Nitrosoarchaeum Nitrosopelagicus Nitrosopumilus Nitrososphaera
Candidatus Cenarchaeum massiliensisK8823_1454	372	Tetratricopeptide repeat-containing protein		IPR019734;IPR001440;IPR019734;IPR019734;IPR019734;IPR019734;IPR013026	OG5_128289	Unknown function	Cenarchaeum Nitrosoarchaeum Nitrosopelagicus Nitrosopumilus Nitrososphaera
Candidatus Cenarchaeum massiliensisK8823_1455	263	rhodanese domain protein	K01011	IPR001763;IPR001763;IPR001763;IPR001763	OG5_174952	Sulfur metabolism	Cenarchaeum Nitrosoarchaeum Nitrosopelagicus Nitrosopumilus Nitrososphaera
Candidatus Cenarchaeum massiliensisK8823_1456	594	ferredoxin-nitrite/sulfite reductase	K00381	IPR006067;IPR005117	OG5_132911	Nitrogen metabolism	Cenarchaeum Nitrosoarchaeum Nitrosopelagicus Nitrosopumilus Nitrososphaera
Candidatus Cenarchaeum massiliensisK8823_1457	271	thiazole biosynthesis enzyme	K03146	IPR002922;IPR022828	OG5_131050	Carbohydrate metabolism	Cenarchaeum Nitrosoarchaeum Nitrosopelagicus Nitrosopumilus Nitrososphaera
Candidatus Cenarchaeum massiliensisK8823_1458	231	hypothetical protein			nmar|YP_001582011	Unknown function	Cenarchaeum Nitrosoarchaeum Nitrosopelagicus Nitrosopumilus Nitrososphaera
Candidatus Cenarchaeum massiliensisK8823_1459	326	thioredoxin-disulfide reductase	K00384	IPR023753;IPR005982;IPR008255	OG5_127532	Nucleotide metabolism	Cenarchaeum Nitrosoarchaeum Nitrosopelagicus Nitrosopumilus Nitrososphaera
Candidatus Cenarchaeum massiliensisK8823_1461	78	hypothetical protein	K04085	IPR001455;IPR001455	OG5_131640	Unknown function	Cenarchaeum Nitrosoarchaeum Nitrosopelagicus Nitrosopumilus Nitrososphaera
Candidatus Cenarchaeum massiliensisK8823_1462	243	F420-0--gamma-glutamyl ligase	K12234	IPR008225;IPR002847	OG5_144298	Methane metabolism	Cenarchaeum Nitrosoarchaeum Nitrosopelagicus Nitrosopumilus Nitrososphaera
Candidatus Cenarchaeum massiliensisK8823_1463	215	phosphoserine phosphatase	K01079	IPR023214;IPR004469;IPR006383;IPR004469	OG5_128110	Amino acid metabolism	Cenarchaeum Nitrosoarchaeum Nitrosopelagicus Nitrosopumilus Nitrososphaera
Candidatus Cenarchaeum massiliensisK8823_1464	94	hypothetical protein			OG5_224775	Unknown function	Cenarchaeum Nitrosoarchaeum Nitrosopelagicus Nitrosopumilus Nitrososphaera
Candidatus Cenarchaeum massiliensisK8823_1465	488	Proline--tRNA ligase	K01881	IPR016061;IPR004154;IPR004499;IPR002314;IPR016061;IPR004499;IPR004499;IPR006195	OG5_127154	Translation	Cenarchaeum Nitrosoarchaeum Nitrosopelagicus Nitrosopumilus Nitrososphaera
Candidatus Cenarchaeum massiliensisK8823_1468	325	oligopeptide ABC transporter ATP-binding protein	K02032	IPR003593;IPR003439;IPR013563;IPR013563;IPR025662;IPR017871;IPR003439	OG5_128913	Transport	Cenarchaeum Nitrosoarchaeum Nitrosopelagicus Nitrosopumilus Nitrososphaera
Candidatus Cenarchaeum massiliensisK8823_1472	676	hypothetical protein	K06888	IPR004879	OG5_129852	Oxidation-reduction	Cenarchaeum Nitrosoarchaeum Nitrosopelagicus Nitrosopumilus Nitrososphaera
Candidatus Cenarchaeum massiliensisK8823_1473	108	hypothetical protein			nmar|YP_001582524	Unknown function	Cenarchaeum Nitrosoarchaeum Nitrosopelagicus Nitrosopumilus Nitrososphaera
Candidatus Cenarchaeum massiliensisK8823_140	214	Snf7 domain-containing protein			OG5_150062	Transport	Cenarchaeum Nitrosoarchaeum Nitrosopelagicus Nitrosopumilus Nitrososphaera
Candidatus Cenarchaeum massiliensisK8823_1476	343	Zn-dependent alcohol dehydrogenase	K13953	IPR013149;IPR013154;IPR002085;IPR002328	OG5_126661	Oxidation-reduction	Cenarchaeum Nitrosoarchaeum Nitrosopelagicus Nitrosopumilus Nitrososphaera
Candidatus Cenarchaeum massiliensisK8823_1480	401	tRNA pseudouridine synthase D	K06176	IPR001656;IPR001656;IPR001656;IPR011760	OG5_127405	Translation	Cenarchaeum Nitrosoarchaeum Nitrosopelagicus Nitrosopumilus Nitrososphaera
Candidatus Cenarchaeum massiliensisK8823_1481	520	lyase			OG5_241582	Unknown function	Cenarchaeum Nitrosoarchaeum Nitrosopelagicus Nitrosopumilus Nitrososphaera
Candidatus Cenarchaeum massiliensisK8823_1482	115	peptidyl-tRNA hydrolase	K04794	IPR002833;IPR002833	OG5_127155	Misc	Cenarchaeum Nitrosoarchaeum Nitrosopelagicus Nitrosopumilus Nitrososphaera
Candidatus Cenarchaeum massiliensisK8823_1483	377	dehydrogenase (flavoprotein)-like protein	K17830		OG5_142646	Oxidation-reduction	Cenarchaeum Nitrosoarchaeum Nitrosopelagicus Nitrosopumilus Nitrososphaera
Candidatus Cenarchaeum massiliensisK8823_1484	93	translation elongation factor aEF-1 beta		IPR014038;IPR014038;IPR004542;IPR004542	OG5_139282	Translation	Cenarchaeum Nitrosoarchaeum Nitrosopelagicus Nitrosopumilus Nitrososphaera
Candidatus Cenarchaeum massiliensisK8823_1485	724	ATPase AAA	K13525	IPR004201;IPR003593;IPR003338;IPR003959;IPR004201;IPR005938;IPR003338;IPR003960	OG5_126926	Protein modification	Cenarchaeum Nitrosoarchaeum Nitrosopelagicus Nitrosopumilus Nitrososphaera
Candidatus Cenarchaeum massiliensisK8823_1486	96	hypothetical protein			nmar|YP_001582558	Unknown function	Cenarchaeum Nitrosoarchaeum Nitrosopelagicus Nitrosopumilus Nitrososphaera
Candidatus Cenarchaeum massiliensisK8823_1487	292	protoheme IX farnesyltransferase	K02301	IPR006369;IPR000537;IPR030470;IPR006369	OG5_127401	Metabolism of cofactors and vitamins	Cenarchaeum Nitrosoarchaeum Nitrosopelagicus Nitrosopumilus Nitrososphaera
Candidatus Cenarchaeum massiliensisK8823_1488	261	blue (type1) copper domain-containing protein			OG5_173303	Misc	Cenarchaeum Nitrosoarchaeum Nitrosopelagicus Nitrosopumilus Nitrososphaera
Candidatus Cenarchaeum massiliensisK8823_1489	148	MPN family protein		IPR000555;IPR028090	OG5_149669	Protein modification	Cenarchaeum Nitrosoarchaeum Nitrosopelagicus Nitrosopumilus Nitrososphaera
Candidatus Cenarchaeum massiliensisK8823_1491	258	Gamma-glutamyl ligase	K12234	IPR002847	OG5_144298	Methane metabolism	Cenarchaeum Nitrosoarchaeum Nitrosopelagicus Nitrosopumilus Nitrososphaera
Candidatus Cenarchaeum massiliensisK8823_1492	183	hypothetical protein			OG5_204909	Unknown function	Cenarchaeum Nitrosoarchaeum Nitrosopelagicus Nitrosopumilus Nitrososphaera
Candidatus Cenarchaeum massiliensisK8823_142	513	bifunctional GMP synthase/glutamine amidotransferase protein (guaA)	K01951	IPR017926;IPR022310;IPR001674;IPR004739;IPR001674;IPR025777;IPR017926;IPR022955	OG5_127152	Nucleotide metabolism	Cenarchaeum Nitrosoarchaeum Nitrosopelagicus Nitrosopumilus Nitrososphaera
Candidatus Cenarchaeum massiliensisK8823_1493	183	hypothetical protein			nmar|YP_001581750	Unknown function	Cenarchaeum Nitrosoarchaeum Nitrosopelagicus Nitrosopumilus Nitrososphaera
Candidatus Cenarchaeum massiliensisK8823_1494	317	pyruvate ferredoxin oxidoreductase beta subunit	K00175	IPR011896;IPR011766	OG5_133522	Energy	Cenarchaeum Nitrosoarchaeum Nitrosopelagicus Nitrosopumilus Nitrososphaera
Candidatus Cenarchaeum massiliensisK8823_1495	631	pyruvate ferredoxin oxidoreductase alpha subunit	K00174	IPR002880;IPR022367;IPR019752	OG5_134497	Energy	Cenarchaeum Nitrosoarchaeum Nitrosopelagicus Nitrosopumilus Nitrososphaera
Candidatus Cenarchaeum massiliensisK8823_1497	324	D-glycerate dehydrogenase	K00090	IPR006140;IPR006139;IPR029753	OG5_126787	Pyruvate metabolism	Cenarchaeum Nitrosoarchaeum Nitrosopelagicus Nitrosopumilus Nitrososphaera
Candidatus Cenarchaeum massiliensisK8823_1498	79	hypothetical protein			nmar|YP_001581744	Unknown function	Cenarchaeum Nitrosoarchaeum Nitrosopelagicus Nitrosopumilus Nitrososphaera
Candidatus Cenarchaeum massiliensisK8823_1499	430	FAD-dependent oxidoreductase	K15736	IPR006076	OG5_127956	Oxidation-reduction	Cenarchaeum Nitrosoarchaeum Nitrosopelagicus Nitrosopumilus Nitrososphaera
Candidatus Cenarchaeum massiliensisK8823_143	189	hypothetical protein			OG5_225060	Unknown function	Cenarchaeum Nitrosoarchaeum Nitrosopelagicus Nitrosopumilus Nitrososphaera
Candidatus Cenarchaeum massiliensisK8823_1501	131	hypothetical protein			nmar|YP_001581741	Unknown function	Cenarchaeum Nitrosoarchaeum Nitrosopelagicus Nitrosopumilus Nitrososphaera
Candidatus Cenarchaeum massiliensisK8823_1502	338	tRNA pseudouridine synthase	K11131	IPR002478;IPR004521;IPR004802;IPR012960;IPR002478;IPR002501;IPR004802;IPR026326;IPR002478	OG5_126700	Translation	Cenarchaeum Nitrosoarchaeum Nitrosopelagicus Nitrosopumilus Nitrososphaera
Candidatus Cenarchaeum massiliensisK8823_1503	185	Nucleoside/nucleotide kinase	K00945		OG5_137352	Nucleotide metabolism	Cenarchaeum Nitrosoarchaeum Nitrosopelagicus Nitrosopumilus Nitrososphaera
Candidatus Cenarchaeum massiliensisK8823_1504	211	Membrane protein		IPR002809	OG5_152630	Unknown function	Cenarchaeum Nitrosoarchaeum Nitrosopelagicus Nitrosopumilus Nitrososphaera
Candidatus Cenarchaeum massiliensisK8823_1505	191	adenylate kinase	K00939	IPR003593;IPR023477	OG5_142678	Nucleotide metabolism	Cenarchaeum Nitrosoarchaeum Nitrosopelagicus Nitrosopumilus Nitrososphaera
Candidatus Cenarchaeum massiliensisK8823_1506	476	preprotein translocase subunit SecY	K10956	IPR002208;IPR019561;IPR002208;IPR002208;IPR030659;IPR030659;IPR026593	OG5_127102	Misc	Cenarchaeum Nitrosoarchaeum Nitrosopelagicus Nitrosopumilus Nitrososphaera
Candidatus Cenarchaeum massiliensisK8823_1507	152	ribosomal protein L15		IPR021131;IPR030878	OG5_127016	Translation	Cenarchaeum Nitrosoarchaeum Nitrosopelagicus Nitrosopumilus Nitrososphaera
Candidatus Cenarchaeum massiliensisK8823_1508	157	ribosomal protein L30P	K02907	IPR016082	OG5_126953	Translation	Cenarchaeum Nitrosoarchaeum Nitrosopelagicus Nitrosopumilus Nitrososphaera
Candidatus Cenarchaeum massiliensisK8823_1509	228	ribosomal protein S5	K02988	IPR005324;IPR013810;IPR005711;IPR000851;IPR018192;IPR013810	OG5_126722	Translation	Cenarchaeum Nitrosoarchaeum Nitrosopelagicus Nitrosopumilus Nitrososphaera

Candidatus Cenarchaeum massiliensis	K8823_144	242	hypothetical Rossmann fold-containing protein	K07142	IPR002826;IPR027510	OG5_141323	Metabolism of cofactors and vitamins	Cenarchaeum Nitrosoarchaeum Nitrosopelagicus Nitrosopumilus Nitrososphaera
Candidatus Cenarchaeum massiliensis	K8823_1510	160	ribosomal protein L18P	K02881	IPR005484;IPR005485;IPR005485	OG5_127090	Translation	Cenarchaeum Nitrosoarchaeum Nitrosopelagicus Nitrosopumilus Nitrososphaera
Candidatus Cenarchaeum massiliensis	K8823_1511	304	ornithine carbamoyltransferase	K00611	IPR002292;IPR006131;IPR006132;IPR006130;IPR024904	OG5_127715	Amino acid metabolism	Cenarchaeum Nitrosoarchaeum Nitrosopelagicus Nitrosopumilus Nitrososphaera
Candidatus Cenarchaeum massiliensis	K8823_1512	422	signal recognition particle-docking protein	K03110	IPR000897;IPR003593;IPR013822;IPR013822;IPR000897;IPR004390;IPR000897	OG5_126932	Misc	Cenarchaeum Nitrosoarchaeum Nitrosopelagicus Nitrosopumilus Nitrososphaera
Candidatus Cenarchaeum massiliensis	K8823_1513	144	prefoldin alpha subunit	K04797	IPR011599;IPR004127;IPR011599	OG5_127820	Protein modification	Cenarchaeum Nitrosoarchaeum Nitrosopelagicus Nitrosopumilus Nitrososphaera
Candidatus Cenarchaeum massiliensis	K8823_1514	206	superoxide dismutase	K04564	IPR019831;IPR019832;IPR001189;IPR019833	OG5_126676	Stress & defense	Cenarchaeum Nitrosoarchaeum Nitrosopelagicus Nitrosopumilus Nitrososphaera
Candidatus Cenarchaeum massiliensis	K8823_1515	899	Alanyl-tRNA synthetase	K01872	IPR012947;IPR018164;IPR022429;IPR012947;IPR003156;IPR022429;IPR018165	OG5_126799	Translation	Cenarchaeum Nitrosoarchaeum Nitrosopelagicus Nitrosopumilus Nitrososphaera
Candidatus Cenarchaeum massiliensis	K8823_145	277	dihydropteroate synthase (folP)	K00796	IPR000489;IPR006390;IPR000489;IPR000489	OG5_128803	Metabolism of cofactors and vitamins	Cenarchaeum Nitrosoarchaeum Nitrosopelagicus Nitrosopumilus Nitrososphaera
Candidatus Cenarchaeum massiliensis	K8823_148	182	hypothetical protein		IPR012312	OG5_158948	Stress & defense	Cenarchaeum Nitrosoarchaeum Nitrosopelagicus Nitrosopumilus Nitrososphaera
Candidatus Cenarchaeum massiliensis	K8823_149	169	adenylyl-sulfate kinase	K00860	IPR002891;IPR002891	OG5_127271	Nucleotide metabolism	Cenarchaeum Nitrosoarchaeum Nitrosopelagicus Nitrosopumilus Nitrososphaera
Candidatus Cenarchaeum massiliensis	K8823_1538	697	ATPase	K02212	IPR031327;IPR001208;IPR001208	OG5_127631	Transcription	Cenarchaeum Nitrosoarchaeum Nitrosopelagicus Nitrosopumilus Nitrososphaera
Candidatus Cenarchaeum massiliensis	K8823_1539	234	proliferating cell nuclear antigen (pcna)			OG5_127352	DNA modification	Cenarchaeum Nitrosoarchaeum Nitrosopelagicus Nitrosopumilus Nitrososphaera
Candidatus Cenarchaeum massiliensis	K8823_150	418	Sodium/hydrogen exchanger		IPR006153;IPR030151	OG5_134073	Transport	Cenarchaeum Nitrosoarchaeum Nitrosopelagicus Nitrosopumilus Nitrososphaera
Candidatus Cenarchaeum massiliensis	K8823_1545	125	hypothetical protein			nmar|YP_001582832	Unknown function	Cenarchaeum Nitrosoarchaeum Nitrosopelagicus Nitrosopumilus Nitrososphaera
Candidatus Cenarchaeum massiliensis	K8823_151	302	transcription initiation factor IIB	K03124	IPR013763;IPR013137;IPR013150;IPR000812;IPR023486;IPR023486;IPR023484;IPR013137	OG5_127288	Transcription	Cenarchaeum Nitrosoarchaeum Nitrosopelagicus Nitrosopumilus Nitrososphaera
Candidatus Cenarchaeum massiliensis	K8823_152	184	RNA-binding protein		IPR030850	OG5_128555	RNA modification	Cenarchaeum Nitrosoarchaeum Nitrosopelagicus Nitrosopumilus Nitrososphaera
Candidatus Cenarchaeum massiliensis	K8823_153	273	prephenate dehydratase	K04518	IPR001086;IPR002912;IPR001086	OG5_128434	Amino acid metabolism	Cenarchaeum Nitrosoarchaeum Nitrosopelagicus Nitrosopumilus Nitrososphaera
Candidatus Cenarchaeum massiliensis	K8823_154	151	hypothetical membrane protein			nmar|YP_001582902	Unknown function	Cenarchaeum Nitrosoarchaeum Nitrosopelagicus Nitrosopumilus Nitrososphaera
Candidatus Cenarchaeum massiliensis	K8823_155	479	inosine-5'-monophosphate dehydrogenase	K00088	IPR000644;IPR005990;IPR001093;IPR000644;IPR015875;IPR000644;IPR005990;IPR000644	OG5_126887	Nucleotide metabolism	Cenarchaeum Nitrosoarchaeum Nitrosopelagicus Nitrosopumilus Nitrososphaera
Candidatus Cenarchaeum massiliensis	K8823_1580	348	aminotransferase	K00817	IPR004839;IPR004838	OG5_127656	Amino acid metabolism	Cenarchaeum Nitrosoarchaeum Nitrosopelagicus Nitrosopumilus Nitrososphaera
Candidatus Cenarchaeum massiliensis	K8823_1581	272	cobalamin biosynthesis protein CobQ	K02232	IPR002586;IPR004459	OG5_133997	Metabolism of cofactors and vitamins	Cenarchaeum Nitrosoarchaeum Nitrosopelagicus Nitrosopumilus Nitrososphaera
Candidatus Cenarchaeum massiliensis	K8823_1582	324	cobalamin biosynthesis protein CobD	K02227	IPR004485;IPR004485;IPR004485	OG5_133537	Metabolism of cofactors and vitamins	Cenarchaeum Nitrosoarchaeum Nitrosopelagicus Nitrosopumilus Nitrososphaera
Candidatus Cenarchaeum massiliensis	K8823_1583	243	cobalamin 5'-phosphate synthase (cobS, cobV)	K02233	IPR003805;IPR003805;IPR003805	OG5_133122	Metabolism of cofactors and vitamins	Cenarchaeum Nitrosoarchaeum Nitrosopelagicus Nitrosopumilus Nitrososphaera
Candidatus Cenarchaeum massiliensis	K8823_1584	195	nucleotidyltransferase	K19712	IPR025877	OG5_146703	Metabolism of cofactors and vitamins	Cenarchaeum Nitrosoarchaeum Nitrosopelagicus Nitrosopumilus Nitrososphaera
Candidatus Cenarchaeum massiliensis	K8823_1585	65	ribosomal protein S27E	K02978	IPR000592;IPR000592;IPR000592	OG5_127008	Translation	Cenarchaeum Nitrosoarchaeum Nitrosopelagicus Nitrosopumilus Nitrososphaera
Candidatus Cenarchaeum massiliensis	K8823_1586	93	ribosomal protein L44E	K02929	IPR000552;IPR000552;IPR000552;IPR000552	OG5_126988	Translation	Cenarchaeum Nitrosoarchaeum Nitrosopelagicus Nitrosopumilus Nitrososphaera
Candidatus Cenarchaeum massiliensis	K8823_1587	280	hypothetical exported protein		IPR002890;IPR027560	OG5_184415	Unknown function	Cenarchaeum Nitrosoarchaeum Nitrosopelagicus Nitrosopumilus Nitrososphaera
Candidatus Cenarchaeum massiliensis	K8823_156	472	archaeal DNA polymerase II, small subunit	K02323	IPR007185;IPR024826;IPR011149	OG5_144240	DNA modification	Cenarchaeum Nitrosoarchaeum Nitrosopelagicus Nitrosopumilus Nitrososphaera
Candidatus Cenarchaeum massiliensis	K8823_1588	83	DNA-directed RNA polymerase Rpb5	K03053	IPR000783;IPR020608;IPR000783;IPR020609	OG5_127455	Transcription	Cenarchaeum Nitrosoarchaeum Nitrosopelagicus Nitrosopumilus Nitrososphaera
Candidatus Cenarchaeum massiliensis	K8823_1589	1115	DNA-directed RNA polymerase Rpb2	K13798	IPR007642;IPR007645;IPR019969;IPR007641;IPR007644;IPR007647;IPR007646;IPR007120;IPR015712	OG5_126694	Transcription	Cenarchaeum Nitrosoarchaeum Nitrosopelagicus Nitrosopumilus Nitrososphaera
Candidatus Cenarchaeum massiliensis	K8823_1590	1261	DNA-directed RNA polymerase subunit A'	K03041	IPR006592;IPR007081;IPR007083;IPR012758;IPR007080;IPR007066;IPR000722;IPR012757	OG5_126637	Transcription	Cenarchaeum Nitrosoarchaeum Nitrosopelagicus Nitrosopumilus Nitrososphaera
Candidatus Cenarchaeum massiliensis	K8823_157	405	cell division control protein Cdc6	K10725	IPR015163;IPR003593;IPR014277;IPR015163;IPR014277	OG5_126808	Cell & development	Cenarchaeum Nitrosoarchaeum Nitrosopelagicus Nitrosopumilus Nitrososphaera
Candidatus Cenarchaeum massiliensis	K8823_158	130	hypothetical protein			nmar|YP_001583133	Unknown function	Cenarchaeum Nitrosoarchaeum Nitrosopelagicus Nitrosopumilus Nitrososphaera
Candidatus Cenarchaeum massiliensis	K8823_1604	336	phosphoesterase DHHA1		IPR003156	OG5_161654	Misc	Cenarchaeum Nitrosoarchaeum Nitrosopelagicus Nitrosopumilus Nitrososphaera
Candidatus Cenarchaeum massiliensis	K8823_1605	138	Glyoxalase-like domain-containing protein		IPR025870	OG5_143131	Pyruvate metabolism	Cenarchaeum Nitrosoarchaeum Nitrosopelagicus Nitrosopumilus Nitrososphaera
Candidatus Cenarchaeum massiliensis	K8823_1606	74	transcriptional regulator AsnC family		IPR019887	OG5_135100	Transcription	Cenarchaeum Nitrosoarchaeum Nitrosopelagicus Nitrosopumilus Nitrososphaera
Candidatus Cenarchaeum massiliensis	K8823_1624	435	N-4 cytosine-specific DNA methylase	K00571	IPR002941;IPR017985	OG5_135744	DNA modification	Cenarchaeum Nitrosoarchaeum Nitrosopelagicus Nitrosopumilus Nitrososphaera
Candidatus Cenarchaeum massiliensis	K8823_15	275	hypothetical protein		IPR002500	OG5_129962	Sulfur metabolism	Cenarchaeum Nitrosoarchaeum Nitrosopelagicus Nitrosopumilus Nitrososphaera
Candidatus Cenarchaeum massiliensis	K8823_1632	377	N-4 cytosine-specific DNA methylase	K00571	IPR002941;IPR017985	OG5_135744	DNA modification	Cenarchaeum Nitrosoarchaeum Nitrosopelagicus Nitrosopumilus Nitrososphaera
Candidatus Cenarchaeum massiliensis	K8823_1638	134	pyridoxamine 5'-phosphate oxidase		IPR011576;IPR019920	OG5_204576	Metabolism of cofactors and vitamins	Cenarchaeum Nitrosoarchaeum Nitrosopelagicus Nitrosopumilus Nitrososphaera
Candidatus Cenarchaeum massiliensis	K8823_1639	188	transcriptional regulator			OG5_178660	Transcription	Cenarchaeum Nitrosoarchaeum Nitrosopelagicus Nitrosopumilus Nitrososphaera
Candidatus Cenarchaeum massiliensis	K8823_1640	106	ribosomal protein L7ae			OG5_127005	Translation	Cenarchaeum Nitrosoarchaeum Nitrosopelagicus Nitrosopumilus Nitrososphaera
Candidatus Cenarchaeum massiliensis	K8823_1641	154	transcription elongation factor NusA	K02600	IPR025249;IPR010212;IPR010212	OG5_139247	Transcription	Cenarchaeum Nitrosoarchaeum Nitrosopelagicus Nitrosopumilus Nitrososphaera
Candidatus Cenarchaeum massiliensis	K8823_1642	145	ribosomal protein S12	K02973	IPR006032;IPR005680;IPR006032;IPR006032;IPR022863	OG5_127029	Translation	Cenarchaeum Nitrosoarchaeum Nitrosopelagicus Nitrosopumilus Nitrososphaera
Candidatus Cenarchaeum massiliensis	K8823_1643	199	ribosomal protein S7	K02992	IPR023798;IPR005716;IPR000235	OG5_127064	Translation	Cenarchaeum Nitrosoarchaeum Nitrosopelagicus Nitrosopumilus Nitrososphaera
Candidatus Cenarchaeum massiliensis	K8823_1645	127	hypothetical protein			nmar|YP_001582505	Unknown function	Cenarchaeum Nitrosoarchaeum Nitrosopelagicus Nitrosopumilus Nitrososphaera
Candidatus Cenarchaeum massiliensis	K8823_1646	554	ribulose-phosphate 3-epimerase	K00615	IPR000056;IPR005474;IPR000056	OG5_126720	Energy	Cenarchaeum Nitrosoarchaeum Nitrosopelagicus Nitrosopumilus Nitrososphaera
Candidatus Cenarchaeum massiliensis	K8823_1647	322	Transketolase C-terminal subunit (tktA, tktB)	K00615	IPR005475;IPR009014;IPR005475	OG5_129068	Energy	Cenarchaeum Nitrosoarchaeum Nitrosopelagicus Nitrosopumilus Nitrososphaera
Candidatus Cenarchaeum massiliensis	K8823_1648	215	transaldolase	K00616	IPR001585;IPR004731;IPR001585;IPR018225;IPR022999	OG5_127238	Energy	Cenarchaeum Nitrosoarchaeum Nitrosopelagicus Nitrosopumilus Nitrososphaera
Candidatus Cenarchaeum massiliensis	K8823_1649	103	hypothetical protein			nmar|YP_001581751	Unknown function	Cenarchaeum Nitrosoarchaeum Nitrosopelagicus Nitrosopumilus Nitrososphaera
Candidatus Cenarchaeum massiliensis	K8823_1656	103	hypothetical protein			nmar|YP_001581836	Unknown function	Cenarchaeum Nitrosoarchaeum Nitrosopelagicus Nitrosopumilus Nitrososphaera
Candidatus Cenarchaeum massiliensis	K8823_1657	220	phosphatase PAP2 family protein		IPR000326;IPR000326	OG5_133119	Misc	Cenarchaeum Nitrosoarchaeum Nitrosopelagicus Nitrosopumilus Nitrososphaera
Candidatus Cenarchaeum massiliensis	K8823_1659	782	valine--tRNA ligase	K01873	IPR002303;IPR013155;IPR002300	OG5_126723	Translation	Cenarchaeum Nitrosoarchaeum Nitrosopelagicus Nitrosopumilus Nitrososphaera
Candidatus Cenarchaeum massiliensis	K8823_1662	271	hypothetical protein		IPR027560	OG5_184415	Unknown function	Cenarchaeum Nitrosoarchaeum Nitrosopelagicus Nitrosopumilus Nitrososphaera
Candidatus Cenarchaeum massiliensis	K8823_1664	103	ATP-utilizing protein	K06864		OG5_137379	Amino acid metabolism	Cenarchaeum Nitrosoarchaeum Nitrosopelagicus Nitrosopumilus Nitrososphaera
Candidatus Cenarchaeum massiliensis	K8823_1666	270	PEFG-CTERM sorting domain-containing protein		IPR027560	OG5_184415	Unknown function	Cenarchaeum Nitrosoarchaeum Nitrosopelagicus Nitrosopumilus Nitrososphaera
Candidatus Cenarchaeum massiliensis	K8823_167	521	ammonia permease	K03320	IPR001905;IPR024041;IPR001905;IPR018047	OG5_126686	Nitrogen metabolism	Cenarchaeum Nitrosoarchaeum Nitrosopelagicus Nitrosopumilus Nitrososphaera
Candidatus Cenarchaeum massiliensis	K8823_1667	260	PEFG-CTERM sorting domain-containing protein		IPR027560	OG5_184415	Unknown function	Cenarchaeum Nitrosoarchaeum Nitrosopelagicus Nitrosopumilus Nitrososphaera
Candidatus Cenarchaeum massiliensis	K8823_1669	271	PEFG-CTERM sorting domain-containing protein		IPR027560	OG5_184415	Unknown function	Cenarchaeum Nitrosoarchaeum Nitrosopelagicus Nitrosopumilus Nitrososphaera
Candidatus Cenarchaeum massiliensis	K8823_1671	133	ATP-utilizing protein	K06864		OG5_137379	Amino acid metabolism	Cenarchaeum Nitrosoarchaeum Nitrosopelagicus Nitrosopumilus Nitrososphaera
Candidatus Cenarchaeum massiliensis	K8823_168	223	hypothetical protein			nmar|YP_001583033	Unknown function	Cenarchaeum Nitrosoarchaeum Nitrosopelagicus Nitrosopumilus Nitrososphaera
Candidatus Cenarchaeum massiliensis	K8823_169	348	ATPase	K02119	IPR002843	OG5_141332	Misc	Cenarchaeum Nitrosoarchaeum Nitrosopelagicus Nitrosopumilus Nitrososphaera
Candidatus Cenarchaeum massiliensis	K8823_170	84	hypothetical membrane protein			STG_0029	Unknown function	Cenarchaeum Nitrosoarchaeum Nitrosopelagicus Nitrosopumilus Nitrososphaera
Candidatus Cenarchaeum massiliensis	K8823_171	213	hypothetical protein			nmar|YP_001583035	Unknown function	Cenarchaeum Nitrosoarchaeum Nitrosopelagicus Nitrosopumilus Nitrososphaera
Candidatus Cenarchaeum massiliensis	K8823_177	205	hypothetical membrane protein			OG5_241590	Unknown function	Cenarchaeum Nitrosoarchaeum Nitrosopelagicus Nitrosopumilus Nitrososphaera
Candidatus Cenarchaeum massiliensis	K8823_178	114	hypothetical protein			OG5_241574	Unknown function	Cenarchaeum Nitrosoarchaeum Nitrosopelagicus Nitrosopumilus Nitrososphaera
Candidatus Cenarchaeum massiliensis	K8823_179	231	uridylate kinase	K09903	IPR011818;IPR001048;IPR011817;IPR011818	OG5_130032	Nucleotide metabolism	Cenarchaeum Nitrosoarchaeum Nitrosopelagicus Nitrosopumilus Nitrososphaera
Candidatus Cenarchaeum massiliensis	K8823_180	422	Metal dependent phosphohydrolase	K06885	IPR003607;IPR006674	OG5_127247	Misc	Cenarchaeum Nitrosoarchaeum Nitrosopelagicus Nitrosopumilus Nitrososphaera
Candidatus Cenarchaeum massiliensis	K8823_181	195	thymidylate kinase	K00943	IPR018094;IPR018094	OG5_126920	Nucleotide metabolism	Cenarchaeum Nitrosoarchaeum Nitrosopelagicus Nitrosopumilus Nitrososphaera
Candidatus Cenarchaeum massiliensis	K8823_183	60	cation-binding protein			OG5_158948	Stress & defense	Cenarchaeum Nitrosoarchaeum Nitrosopelagicus Nitrosopumilus Nitrososphaera
Candidatus Cenarchaeum massiliensis	K8823_184	226	deaminase	K11752	IPR011549;IPR002734	OG5_127122	Metabolism of cofactors and vitamins	Cenarchaeum Nitrosoarchaeum Nitrosopelagicus Nitrosopumilus Nitrososphaera
Candidatus Cenarchaeum massiliensis	K8823_185	396	cytosine deaminase		IPR006680	OG5_142655	Misc	Cenarchaeum Nitrosoarchaeum Nitrosopelagicus Nitrosopumilus Nitrososphaera
Candidatus Cenarchaeum massiliensis	K8823_186	248	GTP cyclohydrolase	K08096	IPR007839;IPR007839;IPR007839;IPR000160	OG5_148182	Metabolism of cofactors and vitamins	Cenarchaeum Nitrosoarchaeum Nitrosopelagicus Nitrosopumilus Nitrososphaera
Candidatus Cenarchaeum massiliensis	K8823_187	138	6,7-dimethyl-8-ribityllumazine synthase	K00794	IPR002180;IPR002180;IPR002180	OG5_128902	Metabolism of cofactors and vitamins	Cenarchaeum Nitrosoarchaeum Nitrosopelagicus Nitrosopumilus Nitrososphaera
Candidatus Cenarchaeum massiliensis	K8823_188	221	3,4-dihydroxy-2-butanone-4-phosphate synthase	K14652	IPR000422;IPR000422	OG5_127275	Metabolism of cofactors and vitamins	Cenarchaeum Nitrosoarchaeum Nitrosopelagicus Nitrosopumilus Nitrososphaera
Candidatus Cenarchaeum massiliensis	K8823_189	194	riboflavin synthase subunit alpha	K00793	IPR026017;IPR001783;IPR001783;IPR026017;IPR026017	OG5_129511	Metabolism of cofactors and vitamins	Cenarchaeum Nitrosoarchaeum Nitrosopelagicus Nitrosopumilus Nitrososphaera
Candidatus Cenarchaeum massiliensis	K8823_193	564	hypothetical protein			OG5_151395	Carbohydrate metabolism	Cenarchaeum Nitrosoarchaeum Nitrosopelagicus Nitrosopumilus Nitrososphaera
Candidatus Cenarchaeum massiliensis	K8823_194	271	hypothetical exported protein			OG5_173168	Unknown function	Cenarchaeum Nitrosoarchaeum Nitrosopelagicus Nitrosopumilus Nitrososphaera
Candidatus Cenarchaeum massiliensis	K8823_195	511	amicyanin		IPR000923	OG5_144955	Misc	Cenarchaeum Nitrosoarchaeum Nitrosopelagicus Nitrosopumilus Nitrososphaera
Candidatus Cenarchaeum massiliensis	K8823_197	287	PEFG-CTERM sorting domain-containing protein		IPR027560;IPR000923	OG5_144955	Misc	Cenarchaeum Nitrosoarchaeum Nitrosopelagicus Nitrosopumilus Nitrososphaera
Candidatus Cenarchaeum massiliensis	K8823_198	209	hypothetical exported protein			OG5_241589	Unknown function	Cenarchaeum Nitrosoarchaeum Nitrosopelagicus Nitrosopumilus Nitrososphaera
Candidatus Cenarchaeum massiliensis	K8823_199	920	Copper resistance D domain-containing protein	K14166	IPR008457;IPR013658;IPR007348	OG5_138801	Transport	Cenarchaeum Nitrosoarchaeum Nitrosopelagicus Nitrosopumilus Nitrososphaera
Candidatus Cenarchaeum massiliensis	K8823_200	443	hypothetical exported protein			OG5_151395	Carbohydrate metabolism	Cenarchaeum Nitrosoarchaeum Nitrosopelagicus Nitrosopumilus Nitrososphaera
Candidatus Cenarchaeum massiliensis	K8823_201	308	aspartate carbamoyltransferase	K00609	IPR006132;IPR002082;IPR006131;IPR006130;IPR002082	OG5_128535	Nucleotide metabolism	Cenarchaeum Nitrosoarchaeum Nitrosopelagicus Nitrosopumilus Nitrososphaera
Candidatus Cenarchaeum massiliensis	K8823_204	169	Copper binding protein		IPR000923	OG5_144955	Misc	Cenarchaeum Nitrosoarchaeum Nitrosopelagicus Nitrosopumilus Nitrososphaera
Candidatus Cenarchaeum massiliensis	K8823_206	442	3-polyprenyl-4-hydroxybenzoate decarboxylase		IPR002830;IPR002830	OG5_130277	Metabolism of cofactors and vitamins	Cenarchaeum Nitrosoarchaeum Nitrosopelagicus Nitrosopumilus Nitrososphaera
Candidatus Cenarchaeum massiliensis	K8823_207	115	hypothetical protein			nmar|YP_001582932	Unknown function	Cenarchaeum Nitrosoarchaeum Nitrosopelagicus Nitrosopumilus Nitrososphaera
Candidatus Cenarchaeum massiliensis	K8823_208	80	hypothetical protein			nmar|YP_001582933	Unknown function	Cenarchaeum Nitrosoarchaeum Nitrosopelagicus Nitrosopumilus Nitrososphaera
Candidatus Cenarchaeum massiliensis	K8823_209	426	hypothetical exported protein			OG5_168846	Unknown function	Cenarchaeum Nitrosoarchaeum Nitrosopelagicus Nitrosopumilus Nitrososphaera
Candidatus Cenarchaeum massiliensis	K8823_210	236	ABC transporter	K09810	IPR003593;IPR003439;IPR017871;IPR003439	OG5_126906	Transport	Cenarchaeum Nitrosoarchaeum Nitrosopelagicus Nitrosopumilus Nitrososphaera
Candidatus Cenarchaeum massiliensis	K8823_212	170	NUDIX hydrolase	K01515	IPR000086;IPR020084;IPR000086	OG5_127505	Nucleotide metabolism	Cenarchaeum Nitrosoarchaeum Nitrosopelagicus Nitrosopumilus Nitrososphaera
Candidatus Cenarchaeum massiliensis	K8823_213	256	hypothetical protein	K06898	IPR000031;IPR000031	OG5_137909	Nucleotide metabolism	Cenarchaeum Nitrosoarchaeum Nitrosopelagicus Nitrosopumilus Nitrososphaera
Candidatus Cenarchaeum massiliensis	K8823_214	529	radical SAM domain protein		IPR006638;IPR007197;IPR023970	OG5_172128	Misc	Cenarchaeum Nitrosoarchaeum Nitrosopelagicus Nitrosopumilus Nitrososphaera
Candidatus Cenarchaeum massiliensis	K8823_215	126	hypothetical protein			OG5_241561	Unknown function	Cenarchaeum Nitrosoarchaeum Nitrosopelagicus Nitrosopumilus Nitrososphaera
Candidatus Cenarchaeum massiliensis	K8823_216	360	hypothetical exported protein			OG5_173168	Unknown function	Cenarchaeum Nitrosoarchaeum Nitrosopelagicus Nitrosopumilus Nitrososphaera
Candidatus Cenarchaeum massiliensis	K8823_217	428	hydroxymethylglutaryl-CoA reductase	K00054	IPR002202;IPR004553;IPR002202;IPR002202	OG5_137804	Lipid metabolism	Cenarchaeum Nitrosoarchaeum Nitrosopelagicus Nitrosopumilus Nitrososphaera
Candidatus Cenarchaeum massiliensis	K8823_218	185	hypothetical membrane protein			nmar|YP_001583131	Unknown function	Cenarchaeum Nitrosoarchaeum Nitrosopelagicus Nitrosopumilus Nitrososphaera
Candidatus Cenarchaeum massiliensis	K8823_219	139	hypothetical protein			nmar|YP_001583130	Unknown function	Cenarchaeum Nitrosoarchaeum Nitrosopelagicus Nitrosopumilus Nitrososphaera

Candidatus Cenarchaeum massiliensisK8823_220	250	Geranylgeranylglyceryl phosphate synthase-like protein (pcrB)	K17104	IPR010946;IPR008205;IPR008205;IPR008205	OG5_137898	Lipid metabolism	Cenarchaeum Nitrosoarchaeum Nitrosopelagicus Nitrosopumilus Nitrososphaera
Candidatus Cenarchaeum massiliensisK8823_221	1120	DNA polymerase II large subunit	K02322	IPR016033;IPR004475;IPR004475	OG5_149716	DNA modification	Cenarchaeum Nitrosoarchaeum Nitrosopelagicus Nitrosopumilus Nitrososphaera
Candidatus Cenarchaeum massiliensisK8823_222	425	serine hydroxymethyltransferase	K00600	IPR001085;IPR001085;IPR001085	OG5_126679	Amino acid metabolism	Cenarchaeum Nitrosoarchaeum Nitrosopelagicus Nitrosopumilus Nitrososphaera
Candidatus Cenarchaeum massiliensisK8823_223	561	thermosome		IPR002423;IPR012714;IPR002423;IPR002194	OG5_127343	Protein modification	Cenarchaeum Nitrosoarchaeum Nitrosopelagicus Nitrosopumilus Nitrososphaera
Candidatus Cenarchaeum massiliensisK8823_224	486	glutamine synthetase	K01915	IPR004809;IPR008146;IPR008147;IPR027302	OG5_127086	Nitrogen metabolism	Cenarchaeum Nitrosoarchaeum Nitrosopelagicus Nitrosopumilus Nitrososphaera
Candidatus Cenarchaeum massiliensisK8823_225	176	hypothetical protein			OG5_196289	Unknown function	Cenarchaeum Nitrosoarchaeum Nitrosopelagicus Nitrosopumilus Nitrososphaera
Candidatus Cenarchaeum massiliensisK8823_226	91	hypothetical membrane protein			nmar|YP_001583122	Unknown function	Cenarchaeum Nitrosoarchaeum Nitrosopelagicus Nitrosopumilus Nitrososphaera
Candidatus Cenarchaeum massiliensisK8823_228	250	oxidoreductase		IPR002198;IPR020904	OG5_127267	Metabolism of cofactors and vitamins	Cenarchaeum Nitrosoarchaeum Nitrosopelagicus Nitrosopumilus Nitrososphaera
Candidatus Cenarchaeum massiliensisK8823_229	243	cobalt-precorrin-7 (C(5))-methyltransferase	K03399	IPR000878;IPR012818	OG5_137863	Metabolism of cofactors and vitamins	Cenarchaeum Nitrosoarchaeum Nitrosopelagicus Nitrosopumilus Nitrososphaera
Candidatus Cenarchaeum massiliensisK8823_230	163	hypothetical protein			nmar|YP_001581344	Unknown function	Cenarchaeum Nitrosoarchaeum Nitrosopelagicus Nitrosopumilus Nitrososphaera
Candidatus Cenarchaeum massiliensisK8823_231	342	peptidyl-prolyl cis-trans isomerase	K03768	IPR002130;IPR024936;IPR020892;IPR002130	OG5_127991	Protein modification	Cenarchaeum Nitrosoarchaeum Nitrosopelagicus Nitrosopumilus Nitrososphaera
Candidatus Cenarchaeum massiliensisK8823_232	439	DNA methylase	K00571	IPR003615;IPR002711;IPR002941;IPR002052	OG5_135744	DNA modification	Cenarchaeum Nitrosoarchaeum Nitrosopelagicus Nitrosopumilus Nitrososphaera
Candidatus Cenarchaeum massiliensisK8823_235	217	hypothetical protein			OG5_172361	Unknown function	Cenarchaeum Nitrosoarchaeum Nitrosopelagicus Nitrosopumilus Nitrososphaera
Candidatus Cenarchaeum massiliensisK8823_236	161	molecular chaperone	K13993	IPR002068	OG5_139264	Stress & defense	Cenarchaeum Nitrosoarchaeum Nitrosopelagicus Nitrosopumilus Nitrososphaera
Candidatus Cenarchaeum massiliensisK8823_237	714	ATPase AAA	K13525	IPR003593;IPR003338;IPR005938;IPR003338;IPR004201;IPR003959;IPR003960;IPR003960	OG5_126926	Protein modification	Cenarchaeum Nitrosoarchaeum Nitrosopelagicus Nitrosopumilus Nitrososphaera
Candidatus Cenarchaeum massiliensisK8823_240	99	hypothetical protein		IPR017896;IPR017896;IPR017896	OG5_159009	Misc	Cenarchaeum Nitrosoarchaeum Nitrosopelagicus Nitrosopumilus Nitrososphaera
Candidatus Cenarchaeum massiliensisK8823_241	193	hypothetical protein			nmar|YP_001583098	Unknown function	Cenarchaeum Nitrosoarchaeum Nitrosopelagicus Nitrosopumilus Nitrososphaera
Candidatus Cenarchaeum massiliensisK8823_244	300	ribonuclease Z	K00784	IPR013471;IPR013471	OG5_126816	Transport	Cenarchaeum Nitrosoarchaeum Nitrosopelagicus Nitrosopumilus Nitrososphaera
Candidatus Cenarchaeum massiliensisK8823_245	305	Ribose-phosphate pyrophosphokinase	K00948	IPR029099;IPR005946;IPR000836	OG5_126730	Energy	Cenarchaeum Nitrosoarchaeum Nitrosopelagicus Nitrosopumilus Nitrososphaera
Candidatus Cenarchaeum massiliensisK8823_248	336	diphthamide biosynthesis protein	K07561	IPR022428;IPR016435;IPR016435;IPR016435	OG5_127598	Amino acid metabolism	Cenarchaeum Nitrosoarchaeum Nitrosopelagicus Nitrosopumilus Nitrososphaera
Candidatus Cenarchaeum massiliensisK8823_249	444	DNA-binding protein	K06932	IPR013696;IPR024913	OG5_141347	RNA modification	Cenarchaeum Nitrosoarchaeum Nitrosopelagicus Nitrosopumilus Nitrososphaera
Candidatus Cenarchaeum massiliensisK8823_250	128	hypothetical protein	K09128	IPR002840	OG5_152580	Unknown function	Cenarchaeum Nitrosoarchaeum Nitrosopelagicus Nitrosopumilus Nitrososphaera
Candidatus Cenarchaeum massiliensisK8823_251	385	hypothetical protein	K09123	IPR007506	OG5_138574	Unknown function	Cenarchaeum Nitrosoarchaeum Nitrosopelagicus Nitrosopumilus Nitrososphaera
Candidatus Cenarchaeum massiliensisK8823_252	222	DNA lyase	K10773	IPR003265;IPR003265;IPR000445;IPR004036;IPR005759	OG5_126913	DNA modification	Cenarchaeum Nitrosoarchaeum Nitrosopelagicus Nitrosopumilus Nitrososphaera
Candidatus Cenarchaeum massiliensisK8823_253	236	hypothetical exported protein			OG5_241555	Unknown function	Cenarchaeum Nitrosoarchaeum Nitrosopelagicus Nitrosopumilus Nitrososphaera
Candidatus Cenarchaeum massiliensisK8823_254	698	V-type ATPase subunit I	K02123	IPR002490;IPR002490	OG5_135299	Transport	Cenarchaeum Nitrosoarchaeum Nitrosopelagicus Nitrosopumilus Nitrososphaera
Candidatus Cenarchaeum massiliensisK8823_255	199	V-type ATP synthase subunit E	K02121	IPR002842	OG5_127015	Oxidation-reduction	Cenarchaeum Nitrosoarchaeum Nitrosopelagicus Nitrosopumilus Nitrososphaera
Candidatus Cenarchaeum massiliensisK8823_256	592	V-type ATP synthase subunit A	K02117	IPR003593;IPR004100;IPR000793;IPR000194;IPR020003;IPR022878	OG5_127237	Oxidation-reduction	Cenarchaeum Nitrosoarchaeum Nitrosopelagicus Nitrosopumilus Nitrososphaera
Candidatus Cenarchaeum massiliensisK8823_257	460	V-type ATP synthase subunit B	K02118	IPR000793;IPR000194;IPR004100;IPR020003;IPR022879	OG5_127061	Oxidation-reduction	Cenarchaeum Nitrosoarchaeum Nitrosopelagicus Nitrosopumilus Nitrososphaera
Candidatus Cenarchaeum massiliensisK8823_258	214	ATP synthase subunit D	K02120	IPR002699;IPR002699;IPR002699;IPR002699	OG5_127301	Oxidation-reduction	Cenarchaeum Nitrosoarchaeum Nitrosopelagicus Nitrosopumilus Nitrososphaera
Candidatus Cenarchaeum massiliensisK8823_259	100	ATP synthase subunit C		IPR002379	OG5_126803	Transport	Cenarchaeum Nitrosoarchaeum Nitrosopelagicus Nitrosopumilus Nitrososphaera
Candidatus Cenarchaeum massiliensisK8823_260	251	rhomboid family intramembrane serine protease		IPR022764;IPR002610	OG5_127742	Protein modification	Cenarchaeum Nitrosoarchaeum Nitrosopelagicus Nitrosopumilus Nitrososphaera
Candidatus Cenarchaeum massiliensisK8823_261	58	4-oxalocrotonate tautomerase		IPR004370	OG5_145066	Misc	Cenarchaeum Nitrosoarchaeum Nitrosopelagicus Nitrosopumilus Nitrososphaera
Candidatus Cenarchaeum massiliensisK8823_262	270	amidohydrolase		IPR003010;IPR001110;IPR003010	OG5_126651	Nitrogen metabolism	Cenarchaeum Nitrosoarchaeum Nitrosopelagicus Nitrosopumilus Nitrososphaera
Candidatus Cenarchaeum massiliensisK8823_263	143	topoisomerase type IA Zn finger domain-containing protein			OG5_126736	DNA modification	Cenarchaeum Nitrosoarchaeum Nitrosopelagicus Nitrosopumilus Nitrososphaera
Candidatus Cenarchaeum massiliensisK8823_264	392	propanoyl-CoA C-acyltransferase	K00626	IPR020617;IPR020616	OG5_128557	Lipid metabolism	Cenarchaeum Nitrosoarchaeum Nitrosopelagicus Nitrosopumilus Nitrososphaera
Candidatus Cenarchaeum massiliensisK8823_265	121	nucleotide-binding protein		IPR002878	OG5_137910	Unknown function	Cenarchaeum Nitrosoarchaeum Nitrosopelagicus Nitrosopumilus Nitrososphaera
Candidatus Cenarchaeum massiliensisK8823_266	47	hypothetical protein			nmar|YP_001582963	Unknown function	Cenarchaeum Nitrosoarchaeum Nitrosopelagicus Nitrosopumilus Nitrososphaera
Candidatus Cenarchaeum massiliensisK8823_268	884	ribonucleoside-diphosphate reductase, adenosylcobalamin-dependent	K00525	IPR013509;IPR000788;IPR013344;IPR005144;IPR005144	OG5_126839	Nucleotide metabolism	Cenarchaeum Nitrosoarchaeum Nitrosopelagicus Nitrosopumilus Nitrososphaera
Candidatus Cenarchaeum massiliensisK8823_269	225	ribosome biogenesis protein	K14568	IPR005304;IPR005304;IPR023503	OG5_127748	Translation	Cenarchaeum Nitrosoarchaeum Nitrosopelagicus Nitrosopumilus Nitrososphaera
Candidatus Cenarchaeum massiliensisK8823_270	1152	peptidase S8		IPR000209;IPR015500;IPR015500;IPR015500;IPR015500;IPR023827;IPR023828	OG5_182769	Protein modification	Cenarchaeum Nitrosoarchaeum Nitrosopelagicus Nitrosopumilus Nitrososphaera
Candidatus Cenarchaeum massiliensisK8823_271	202	rieske iron sulfur protein		IPR019546;IPR017941;IPR017941;IPR006311	OG5_135583	Oxidation-reduction	Cenarchaeum Nitrosoarchaeum Nitrosopelagicus Nitrosopumilus Nitrososphaera
Candidatus Cenarchaeum massiliensisK8823_272	491	cytochrome b/b6 domain-containing protein	K00412	IPR005797;IPR005798;IPR005797;IPR005798	OG5_128504	Oxidation-reduction	Cenarchaeum Nitrosoarchaeum Nitrosopelagicus Nitrosopumilus Nitrososphaera
Candidatus Cenarchaeum massiliensisK8823_275	442	DNA Methylase	K00571	IPR003615;IPR002711;IPR002941	OG5_135744	DNA modification	Cenarchaeum Nitrosoarchaeum Nitrosopelagicus Nitrosopumilus Nitrososphaera
Candidatus Cenarchaeum massiliensisK8823_276	151	hypothetical membrane protein			nmar|YP_001582740	Unknown function	Cenarchaeum Nitrosoarchaeum Nitrosopelagicus Nitrosopumilus Nitrososphaera
Candidatus Cenarchaeum massiliensisK8823_277	395	DNA topoisomerase	K03166	IPR013049;IPR002815;IPR004085	OG5_127274	DNA modification	Cenarchaeum Nitrosoarchaeum Nitrosopelagicus Nitrosopumilus Nitrososphaera
Candidatus Cenarchaeum massiliensisK8823_278	613	DNA topoisomerase VI subunit B	K03167	IPR003594;IPR015320;IPR005734;IPR015320;IPR005734	OG5_134782	DNA modification	Cenarchaeum Nitrosoarchaeum Nitrosopelagicus Nitrosopumilus Nitrososphaera
Candidatus Cenarchaeum massiliensisK8823_279	190	RNA-processing protein	K06961	IPR004087;IPR019964;IPR004088;IPR004088	OG5_127464	Translation	Cenarchaeum Nitrosoarchaeum Nitrosopelagicus Nitrosopumilus Nitrososphaera
Candidatus Cenarchaeum massiliensisK8823_280	264	non-specific serine/threonine protein kinase, RIO kinase 1	K07178	IPR000687;IPR017407	OG5_127476	Translation	Cenarchaeum Nitrosoarchaeum Nitrosopelagicus Nitrosopumilus Nitrososphaera
Candidatus Cenarchaeum massiliensisK8823_281	103	translation initiation factor 1	K03236	IPR001253;IPR001253;IPR006196;IPR001253;IPR001253;IPR001253;IPR006196	OG5_127127	Translation	Cenarchaeum Nitrosoarchaeum Nitrosopelagicus Nitrosopumilus Nitrososphaera
Candidatus Cenarchaeum massiliensisK8823_282	99	hypothetical protein		IPR007355	OG5_149729	Unknown function	Cenarchaeum Nitrosoarchaeum Nitrosopelagicus Nitrosopumilus Nitrososphaera
Candidatus Cenarchaeum massiliensisK8823_283	140	translation initiation factor IF-2	K03238	IPR002735;IPR002735	OG5_127339	Translation	Cenarchaeum Nitrosoarchaeum Nitrosopelagicus Nitrosopumilus Nitrososphaera
Candidatus Cenarchaeum massiliensisK8823_284	47	hypothetical protein			OG5_197677	Unknown function	Cenarchaeum Nitrosoarchaeum Nitrosopelagicus Nitrosopumilus Nitrososphaera
Candidatus Cenarchaeum massiliensisK8823_285	162	hypothetical membrane protein			nmar|YP_001582748	Unknown function	Cenarchaeum Nitrosoarchaeum Nitrosopelagicus Nitrosopumilus Nitrososphaera
Candidatus Cenarchaeum massiliensisK8823_286	378	membrane protein	K08975	IPR007254	OG5_139301	Unknown function	Cenarchaeum Nitrosoarchaeum Nitrosopelagicus Nitrosopumilus Nitrososphaera
Candidatus Cenarchaeum massiliensisK8823_287	246	di-trans,poly-cis-decaprenylcistransferase	K00806	IPR001441;IPR001441;IPR001441;IPR018520;IPR001441	OG5_126777	Lipid metabolism	Cenarchaeum Nitrosoarchaeum Nitrosopelagicus Nitrosopumilus Nitrososphaera
Candidatus Cenarchaeum massiliensisK8823_288	140	NUDIX hydrolase	K01518	IPR000086;IPR020084;IPR000086	OG5_130658	Misc	Cenarchaeum Nitrosoarchaeum Nitrosopelagicus Nitrosopumilus Nitrososphaera
Candidatus Cenarchaeum massiliensisK8823_289	227	orotidine 5-phosphate decarboxylase	K01591	IPR001754;IPR001754	OG5_126793	Nucleotide metabolism	Cenarchaeum Nitrosoarchaeum Nitrosopelagicus Nitrosopumilus Nitrososphaera
Candidatus Cenarchaeum massiliensisK8823_290	230	hypothetical protein			OG5_241587	Unknown function	Cenarchaeum Nitrosoarchaeum Nitrosopelagicus Nitrosopumilus Nitrososphaera
Candidatus Cenarchaeum massiliensisK8823_291	335	Adenylosuccinate synthetase	K01939	IPR001114;IPR001114;IPR001114;IPR018220;IPR001114	OG5_127011	Nucleotide metabolism	Cenarchaeum Nitrosoarchaeum Nitrosopelagicus Nitrosopumilus Nitrososphaera
Candidatus Cenarchaeum massiliensisK8823_292	153	hypothetical protein			nmar|YP_001582755	Unknown function	Cenarchaeum Nitrosoarchaeum Nitrosopelagicus Nitrosopumilus Nitrososphaera
Candidatus Cenarchaeum massiliensisK8823_293	89	Iron traffic domain-containing protein		IPR007457;IPR007457	OG5_145167	Transport	Cenarchaeum Nitrosoarchaeum Nitrosopelagicus Nitrosopumilus Nitrososphaera
Candidatus Cenarchaeum massiliensisK8823_294	69	hypothetical protein			nmar|YP_001582758	Unknown function	Cenarchaeum Nitrosoarchaeum Nitrosopelagicus Nitrosopumilus Nitrososphaera
Candidatus Cenarchaeum massiliensisK8823_295	211	hypothetical membrane protein			nmar|YP_001582759	Unknown function	Cenarchaeum Nitrosoarchaeum Nitrosopelagicus Nitrosopumilus Nitrososphaera
Candidatus Cenarchaeum massiliensisK8823_296	95	transcriptional regulator			OG5_163303	Transcription	Cenarchaeum Nitrosoarchaeum Nitrosopelagicus Nitrosopumilus Nitrososphaera
Candidatus Cenarchaeum massiliensisK8823_297	202	Sua5/YciO/YrdC/YwlC family protein	K07566	IPR006070;IPR006070;IPR006070	OG5_126848	Translation	Cenarchaeum Nitrosoarchaeum Nitrosopelagicus Nitrosopumilus Nitrososphaera
Candidatus Cenarchaeum massiliensisK8823_298	175	RNA methyltransferase		IPR004114	OG5_163330	RNA modification	Cenarchaeum Nitrosoarchaeum Nitrosopelagicus Nitrosopumilus Nitrososphaera
Candidatus Cenarchaeum massiliensisK8823_299	256	sulfur carrier protein ThiS		IPR003749	nmar|YP_001582763	Sulfur metabolism	Cenarchaeum Nitrosoarchaeum Nitrosopelagicus Nitrosopumilus Nitrososphaera
Candidatus Cenarchaeum massiliensisK8823_300	88	Ribosomal protein S17E		IPR001210	OG5_168194	Translation	Cenarchaeum Nitrosoarchaeum Nitrosopelagicus Nitrosopumilus Nitrososphaera
Candidatus Cenarchaeum massiliensisK8823_301	273	TatD-related DNase	K03424	IPR001130;IPR001130	OG5_126665	Misc	Cenarchaeum Nitrosoarchaeum Nitrosopelagicus Nitrosopumilus Nitrososphaera
Candidatus Cenarchaeum massiliensisK8823_302	73	Archaeal histone		IPR003958	OG5_137375	Transcription	Cenarchaeum Nitrosoarchaeum Nitrosopelagicus Nitrosopumilus Nitrososphaera
Candidatus Cenarchaeum massiliensisK8823_303	219	hypothetical protein			nmar|YP_001582779	Unknown function	Cenarchaeum Nitrosoarchaeum Nitrosopelagicus Nitrosopumilus Nitrososphaera
Candidatus Cenarchaeum massiliensisK8823_304	86	Winged helix-turn-helix protein			OG5_163303	Transcription	Cenarchaeum Nitrosoarchaeum Nitrosopelagicus Nitrosopumilus Nitrososphaera
Candidatus Cenarchaeum massiliensisK8823_306	427	phosphoglucomutase/phosphomannomutase alpha/beta/alpha domain I	K03431	IPR005843;IPR005846;IPR005844;IPR005845	OG5_128112	Carbonydrate metabolism	Cenarchaeum Nitrosoarchaeum Nitrosopelagicus Nitrosopumilus Nitrososphaera
Candidatus Cenarchaeum massiliensisK8823_307	310	thiamine-monophosphate kinase (thiL)	K00946	IPR006283;IPR016188;IPR006283	OG5_132585	Metabolism of cofactors and vitamins	Cenarchaeum Nitrosoarchaeum Nitrosopelagicus Nitrosopumilus Nitrososphaera
Candidatus Cenarchaeum massiliensisK8823_308	91	enoyl-CoA hydratase	K15016	IPR001753	OG5_128562	Energy	Cenarchaeum Nitrosoarchaeum Nitrosopelagicus Nitrosopumilus Nitrososphaera
Candidatus Cenarchaeum massiliensisK8823_309	132	enoyl-CoA hydratase	K01715	IPR001753	OG5_128562	Energy	Cenarchaeum Nitrosoarchaeum Nitrosopelagicus Nitrosopumilus Nitrososphaera
Candidatus Cenarchaeum massiliensisK8823_310	127	hypothetical membrane protein			OG5_241559	Unknown function	Cenarchaeum Nitrosoarchaeum Nitrosopelagicus Nitrosopumilus Nitrososphaera
Candidatus Cenarchaeum massiliensisK8823_311	724	CoA-binding domain-containing protein	K09181	IPR003781;IPR003781;IPR011761	OG5_131604	Misc	Cenarchaeum Nitrosoarchaeum Nitrosopelagicus Nitrosopumilus Nitrososphaera
Candidatus Cenarchaeum massiliensisK8823_313	499	4-hydroxybutyryl-CoA dehydratase	K14534	IPR024719;IPR024674	OG5_168811	Lipid metabolism	Cenarchaeum Nitrosoarchaeum Nitrosopelagicus Nitrosopumilus Nitrososphaera
Candidatus Cenarchaeum massiliensisK8823_314	231	peptidase S26B, signal peptidase		IPR019759;IPR001733;IPR001733	OG5_127356	Protein modification	Cenarchaeum Nitrosoarchaeum Nitrosopelagicus Nitrosopumilus Nitrososphaera
Candidatus Cenarchaeum massiliensisK8823_315	235	hypothetical protein		IPR026870	OG5_241560	Unknown function	Cenarchaeum Nitrosoarchaeum Nitrosopelagicus Nitrosopumilus Nitrososphaera
Candidatus Cenarchaeum massiliensisK8823_316	107	Thioredoxin	K03671	IPR013766;IPR005746;IPR005746;IPR017937;IPR012336	OG5_126613	Protein modification	Cenarchaeum Nitrosoarchaeum Nitrosopelagicus Nitrosopumilus Nitrososphaera
Candidatus Cenarchaeum massiliensisK8823_317	174	Zinc-ribbon domain-containing protein			nmar|YP_001581546	Unknown function	Cenarchaeum Nitrosoarchaeum Nitrosopelagicus Nitrosopumilus Nitrososphaera
Candidatus Cenarchaeum massiliensisK8823_318	120	hypothetical protein			nmar|YP_001581545	Unknown function	Cenarchaeum Nitrosoarchaeum Nitrosopelagicus Nitrosopumilus Nitrososphaera
Candidatus Cenarchaeum massiliensisK8823_319	469	Dihydropyrimidinase	K01464	IPR006680	OG5_127801	Nucleotide metabolism	Cenarchaeum Nitrosoarchaeum Nitrosopelagicus Nitrosopumilus Nitrososphaera
Candidatus Cenarchaeum massiliensisK8823_320	185	ribose 5-phosphate isomerase A	K01807	IPR004788;IPR004788;IPR004788	OG5_127414	Energy	Cenarchaeum Nitrosoarchaeum Nitrosopelagicus Nitrosopumilus Nitrososphaera
Candidatus Cenarchaeum massiliensisK8823_321	295	hypothetical protein			OG5_171260	Unknown function	Cenarchaeum Nitrosoarchaeum Nitrosopelagicus Nitrosopumilus Nitrososphaera
Candidatus Cenarchaeum massiliensisK8823_323	77	small nuclear ribonucleoprotein	K04796	IPR001163;IPR001163	OG5_128490	Misc	Cenarchaeum Nitrosoarchaeum Nitrosopelagicus Nitrosopumilus Nitrososphaera
Candidatus Cenarchaeum massiliensisK8823_324	247	creatininase	K01470	IPR003785	OG5_134861	Amino acid metabolism	Cenarchaeum Nitrosoarchaeum Nitrosopelagicus Nitrosopumilus Nitrososphaera
Candidatus Cenarchaeum massiliensisK8823_325	277	formyl transferase domain-containing protein	K01433	IPR002376;IPR004810	OG5_131386	Energy	Cenarchaeum Nitrosoarchaeum Nitrosopelagicus Nitrosopumilus Nitrososphaera
Candidatus Cenarchaeum massiliensisK8823_326	494	phosphoglucosamine mutase	K15778	IPR005844;IPR005845;IPR024086;IPR005843;IPR005846;IPR016066	OG5_128112	Carbonydrate metabolism	Cenarchaeum Nitrosoarchaeum Nitrosopelagicus Nitrosopumilus Nitrososphaera
Candidatus Cenarchaeum massiliensisK8823_327	128	Ribosomal protein L7Ae	K02936	IPR022481;IPR004038;IPR022481	OG5_127322	Translation	Cenarchaeum Nitrosoarchaeum Nitrosopelagicus Nitrosopumilus Nitrososphaera
Candidatus Cenarchaeum massiliensisK8823_328	71	Ribosomal protein S28E/S33		IPR000289;IPR000289;IPR000289	OG5_127046	Translation	Cenarchaeum Nitrosoarchaeum Nitrosopelagicus Nitrosopumilus Nitrososphaera
Candidatus Cenarchaeum massiliensisK8823_329	45	ribosomal protein L24e		IPR000988	OG5_127497	Translation	Cenarchaeum Nitrosoarchaeum Nitrosopelagicus Nitrosopumilus Nitrososphaera
Candidatus Cenarchaeum massiliensisK8823_330	133	nucleoside-diphosphate kinase (ndk)	K00940	IPR001564;IPR001564	OG5_126708	Nucleotide metabolism	Cenarchaeum Nitrosoarchaeum Nitrosopelagicus Nitrosopumilus Nitrososphaera
Candidatus Cenarchaeum massiliensisK8823_331	593	Translation initiation factor aIF-2	K03243	IPR004544;IPR005225;IPR029459;IPR023115;IPR004544;IPR000795	OG5_127312	Translation	Cenarchaeum Nitrosoarchaeum Nitrosopelagicus Nitrosopumilus Nitrososphaera
Candidatus Cenarchaeum massiliensisK8823_332	136	thiol-disulfide isomerase	K03671	IPR013766;IPR005746;IPR005746;IPR017937;IPR012336	OG5_126613	Protein modification	Cenarchaeum Nitrosoarchaeum Nitrosopelagicus Nitrosopumilus Nitrososphaera

Candidatus Cenarchaeum massiliensisK8823_333	134	hypothetical protein		IPR023572;IPR002804;IPR023572	OG5_129252	Unknown function	Cenarchaeum Nitrosoarchaeum Nitrosopelagicus Nitrosopumilus Nitrososphaera
Candidatus Cenarchaeum massiliensisK8823_334	424	histidine--tRNA ligase	K01892	IPR004154;IPR015807;IPR004516;IPR006195	OG5_126806	Translation	Cenarchaeum Nitrosoarchaeum Nitrosopelagicus Nitrosopumilus Nitrososphaera
Candidatus Cenarchaeum massiliensisK8823_335	220	translation initiation factor 6	K03264	IPR002769;IPR002769;IPR002769;IPR002769;IPR002769	OG5_127488	Translation	Cenarchaeum Nitrosoarchaeum Nitrosopelagicus Nitrosopumilus Nitrososphaera
Candidatus Cenarchaeum massiliensisK8823_336	181	ferredoxin		IPR017896;IPR017900;IPR017896;IPR017896	OG5_187395	Misc	Cenarchaeum Nitrosoarchaeum Nitrosopelagicus Nitrosopumilus Nitrososphaera
Candidatus Cenarchaeum massiliensisK8823_338	320	replication factor C small subunit	K04801	IPR003593;IPR008824;IPR013748	OG5_127600	Transcription	Cenarchaeum Nitrosoarchaeum Nitrosopelagicus Nitrosopumilus Nitrososphaera
Candidatus Cenarchaeum massiliensisK8823_339	172	hypothetical protein			nmar|YP_001581575	Unknown function	Cenarchaeum Nitrosoarchaeum Nitrosopelagicus Nitrosopumilus Nitrososphaera
Candidatus Cenarchaeum massiliensisK8823_340	695	MCM family protein	K10726	IPR003593;IPR031327;IPR001208;IPR027925;IPR001208	OG5_127631	Transcription	Cenarchaeum Nitrosoarchaeum Nitrosopelagicus Nitrosopumilus Nitrososphaera
Candidatus Cenarchaeum massiliensisK8823_341	712	DEAD/DEAH box helicase	K03726	IPR014001;IPR001650;IPR001650;IPR011545;IPR022965;IPR014001;IPR001650	OG5_126701	DNA modification	Cenarchaeum Nitrosoarchaeum Nitrosopelagicus Nitrosopumilus Nitrososphaera
Candidatus Cenarchaeum massiliensisK8823_342	78	hypothetical protein		IPR005651	OG5_150845	Unknown function	Cenarchaeum Nitrosoarchaeum Nitrosopelagicus Nitrosopumilus Nitrososphaera
Candidatus Cenarchaeum massiliensisK8823_343	302	branched-chain amino acid aminotransferase	K00826	IPR005785;IPR001544;IPR001544;IPR018300	OG5_126731	Amino acid metabolism	Cenarchaeum Nitrosoarchaeum Nitrosopelagicus Nitrosopumilus Nitrososphaera
Candidatus Cenarchaeum massiliensisK8823_344	200	hypothetical protein			nmar|YP_001581586	Unknown function	Cenarchaeum Nitrosoarchaeum Nitrosopelagicus Nitrosopumilus Nitrososphaera
Candidatus Cenarchaeum massiliensisK8823_345	281	oxidoreductase FAD/NAD(P)-binding subunit	K00528	IPR008333;IPR001433;IPR017927	OG5_137342	Energy	Cenarchaeum Nitrosoarchaeum Nitrosopelagicus Nitrosopumilus Nitrososphaera
Candidatus Cenarchaeum massiliensisK8823_348	94	DNA-binding protein	K03622	IPR002775;IPR013795	OG5_139287	Misc	Cenarchaeum Nitrosoarchaeum Nitrosopelagicus Nitrosopumilus Nitrososphaera
Candidatus Cenarchaeum massiliensisK8823_350	160	transcriptional regulator PadR-like family		IPR005149	OG5_130800	Transcription	Cenarchaeum Nitrosoarchaeum Nitrosopelagicus Nitrosopumilus Nitrososphaera
Candidatus Cenarchaeum massiliensisK8823_351	566	fumarate reductase flavoprotein subunit	K00239	IPR014006;IPR015939;IPR003953;IPR003952	OG5_126927	Energy	Cenarchaeum Nitrosoarchaeum Nitrosopelagicus Nitrosopumilus Nitrososphaera
Candidatus Cenarchaeum massiliensisK8823_352	144	succinate dehydrogenase transmembrane subunit		IPR000701	OG5_144477	Oxidation-reduction	Cenarchaeum Nitrosoarchaeum Nitrosopelagicus Nitrosopumilus Nitrososphaera
Candidatus Cenarchaeum massiliensisK8823_353	108	succinate dehydrogenase transmembrane subunit		IPR000701	OG5_172140	Misc	Cenarchaeum Nitrosoarchaeum Nitrosopelagicus Nitrosopumilus Nitrososphaera
Candidatus Cenarchaeum massiliensisK8823_354	245	succinate dehydrogenase	K00240	IPR017896;IPR025192;IPR004489;IPR006058;IPR017900;IPR001041;IPR017896	OG5_126893	Energy	Cenarchaeum Nitrosoarchaeum Nitrosopelagicus Nitrosopumilus Nitrososphaera
Candidatus Cenarchaeum massiliensisK8823_355	117	metal-sulfur cluster biosynthetic enzyme		IPR002744	OG5_203664	Unknown function	Cenarchaeum Nitrosoarchaeum Nitrosopelagicus Nitrosopumilus Nitrososphaera
Candidatus Cenarchaeum massiliensisK8823_356	167	argininosuccinate lyase	K01755	IPR022761;IPR009049;IPR000362	OG5_127862	Amino acid metabolism	Cenarchaeum Nitrosoarchaeum Nitrosopelagicus Nitrosopumilus Nitrososphaera
Candidatus Cenarchaeum massiliensisK8823_357	294	argininosuccinate lyase	K01755	IPR029419;IPR022761;IPR009049;IPR000362	OG5_127862	Amino acid metabolism	Cenarchaeum Nitrosoarchaeum Nitrosopelagicus Nitrosopumilus Nitrososphaera
Candidatus Cenarchaeum massiliensisK8823_358	292	electron transporter		IPR019545;IPR019545	OG5_151931	Protein modification	Cenarchaeum Nitrosoarchaeum Nitrosopelagicus Nitrosopumilus Nitrososphaera
Candidatus Cenarchaeum massiliensisK8823_359	100	rhodanese domain containing protein		IPR001763;IPR001763;IPR001763	OG5_127147	Misc	Cenarchaeum Nitrosoarchaeum Nitrosopelagicus Nitrosopumilus Nitrososphaera
Candidatus Cenarchaeum massiliensisK8823_361	352	Peptide chain release factor subunit 1 protein	K03265	IPR005141	OG5_127250	Translation	Cenarchaeum Nitrosoarchaeum Nitrosopelagicus Nitrosopumilus Nitrososphaera
Candidatus Cenarchaeum massiliensisK8823_362	127	transcriptional regulator	K07722	IPR002145;IPR014864	OG5_136505	Transcription	Cenarchaeum Nitrosoarchaeum Nitrosopelagicus Nitrosopumilus Nitrososphaera
Candidatus Cenarchaeum massiliensisK8823_363	304	periplasmic solute-binding family protein	K09815	IPR006127;IPR006127	OG5_129567	Transport	Cenarchaeum Nitrosoarchaeum Nitrosopelagicus Nitrosopumilus Nitrososphaera
Candidatus Cenarchaeum massiliensisK8823_364	269	ABC-type Mn2 /Zn2 transport system, ATPase component (znuC)	K09817	IPR003593;IPR003439;IPR017871;IPR003439	OG5_130330	Transport	Cenarchaeum Nitrosoarchaeum Nitrosopelagicus Nitrosopumilus Nitrososphaera
Candidatus Cenarchaeum massiliensisK8823_365	281	ABC 3 transport family protein	K09816	IPR001626	OG5_129762	Transport	Cenarchaeum Nitrosoarchaeum Nitrosopelagicus Nitrosopumilus Nitrososphaera
Candidatus Cenarchaeum massiliensisK8823_386	269	inositol-phosphate phosphatase	K01092	IPR000760;IPR000760	OG5_126810	Stress & defense	Cenarchaeum Nitrosoarchaeum Nitrosopelagicus Nitrosopumilus Nitrososphaera
Candidatus Cenarchaeum massiliensisK8823_387	373	hypothetical protein			nmar|YP_001581603	Unknown function	Cenarchaeum Nitrosoarchaeum Nitrosopelagicus Nitrosopumilus Nitrososphaera
Candidatus Cenarchaeum massiliensisK8823_388	385	ATPase involved in DNA replication	K04800	IPR003593;IPR003959	OG5_127465	DNA modification	Cenarchaeum Nitrosoarchaeum Nitrosopelagicus Nitrosopumilus Nitrososphaera
Candidatus Cenarchaeum massiliensisK8823_389	105	hypothetical protein			nmar|YP_001581605	Unknown function	Cenarchaeum Nitrosoarchaeum Nitrosopelagicus Nitrosopumilus Nitrososphaera
Candidatus Cenarchaeum massiliensisK8823_390	137	hypothetical protein			nmar|YP_001581598	Unknown function	Cenarchaeum Nitrosoarchaeum Nitrosopelagicus Nitrosopumilus Nitrososphaera
Candidatus Cenarchaeum massiliensisK8823_391	514	carboxyl transferase (PCCB, pccB)	K01966	IPR000022;IPR011762;IPR011763	OG5_130273	Lipid metabolism	Cenarchaeum Nitrosoarchaeum Nitrosopelagicus Nitrosopumilus Nitrososphaera
Candidatus Cenarchaeum massiliensisK8823_392	494	acetyl-CoA carboxylase	K01961	IPR005482;IPR005479;IPR005481;IPR005482;IPR005479;IPR011761;IPR011764	OG5_126626	Metabolism of cofactors and vitamins	Cenarchaeum Nitrosoarchaeum Nitrosopelagicus Nitrosopumilus Nitrososphaera
Candidatus Cenarchaeum massiliensisK8823_393	170	acetyl-CoA carboxylase biotin carboxyl carrier protein subunit	K01965	IPR000089;IPR000089	OG5_126626	Metabolism of cofactors and vitamins	Cenarchaeum Nitrosoarchaeum Nitrosopelagicus Nitrosopumilus Nitrososphaera
Candidatus Cenarchaeum massiliensisK8823_400	159	peroxiredoxin	K03564	IPR000866;IPR012336	OG5_133303	Oxidation-reduction	Cenarchaeum Nitrosoarchaeum Nitrosopelagicus Nitrosopumilus Nitrososphaera
Candidatus Cenarchaeum massiliensisK8823_401	123	NADH-ubiquinone oxidoreductase subunit A		IPR000440;IPR000440	OG5_130130	Oxidation-reduction	Cenarchaeum Nitrosoarchaeum Nitrosopelagicus Nitrosopumilus Nitrososphaera
Candidatus Cenarchaeum massiliensisK8823_402	189	NADH-quinone oxidoreductase subunit B	K00331	IPR006138;IPR006137	OG5_127327	Oxidation-reduction	Cenarchaeum Nitrosoarchaeum Nitrosopelagicus Nitrosopumilus Nitrososphaera
Candidatus Cenarchaeum massiliensisK8823_403	180	NADH-ubiquinone oxidoreductase subunit C	K00332	IPR001268;IPR020396;IPR001268	OG5_128842	Oxidation-reduction	Cenarchaeum Nitrosoarchaeum Nitrosopelagicus Nitrosopumilus Nitrososphaera
Candidatus Cenarchaeum massiliensisK8823_404	377	NADH-quinone oxidoreductase subunit D	K00333	IPR001135;IPR014029	OG5_127839	Oxidation-reduction	Cenarchaeum Nitrosoarchaeum Nitrosopelagicus Nitrosopumilus Nitrososphaera
Candidatus Cenarchaeum massiliensisK8823_405	432	NADH-ubiquinone oxidoreductase subunit H	K00337	IPR001694;IPR001694;IPR018086;IPR018086;IPR001694	OG5_128714	Oxidation-reduction	Cenarchaeum Nitrosoarchaeum Nitrosopelagicus Nitrosopumilus Nitrososphaera
Candidatus Cenarchaeum massiliensisK8823_406	165	NADH-ubiquinone oxidoreductase subunit I	K00338	IPR017896;IPR017900;IPR017900;IPR017896;IPR017896	OG5_128019	Oxidation-reduction	Cenarchaeum Nitrosoarchaeum Nitrosopelagicus Nitrosopumilus Nitrososphaera
Candidatus Cenarchaeum massiliensisK8823_407	170	NADH-ubiquinone oxidoreductase	K05578	IPR001457	OG5_130964	Oxidation-reduction	Cenarchaeum Nitrosoarchaeum Nitrosopelagicus Nitrosopumilus Nitrososphaera
Candidatus Cenarchaeum massiliensisK8823_408	70	NADH-ubiquinone oxidoreductase subunit 4L	K00340	IPR001133;IPR001133	OG5_130712	Transport	Cenarchaeum Nitrosoarchaeum Nitrosopelagicus Nitrosopumilus Nitrososphaera
Candidatus Cenarchaeum massiliensisK8823_409	501	Proton-translocating NADH-quinone oxidoreductase, chain M	K00342	IPR010227;IPR001750	OG5_129156	Oxidation-reduction	Cenarchaeum Nitrosoarchaeum Nitrosopelagicus Nitrosopumilus Nitrososphaera
Candidatus Cenarchaeum massiliensisK8823_410	690	Proton-translocating NADH-quinone oxidoreductase subunit L	K00341	IPR001750;IPR001516;IPR003945	OG5_128850	Oxidation-reduction	Cenarchaeum Nitrosoarchaeum Nitrosopelagicus Nitrosopumilus Nitrososphaera
Candidatus Cenarchaeum massiliensisK8823_411	492	NADH-ubiquinone oxidoreductase subunit N	K00343	IPR001750;IPR010096	OG5_129510	Transport	Cenarchaeum Nitrosoarchaeum Nitrosopelagicus Nitrosopumilus Nitrososphaera
Candidatus Cenarchaeum massiliensisK8823_412	287	polyprenyl synthetase	K02523	IPR000092;IPR017446;IPR000092	OG5_126937	Lipid metabolism	Cenarchaeum Nitrosoarchaeum Nitrosopelagicus Nitrosopumilus Nitrososphaera
Candidatus Cenarchaeum massiliensisK8823_33	376	Sulfate adenylyltransferase	K00958	IPR024951;IPR025980;IPR002650;IPR020792	OG5_130587	Sulfur metabolism	Cenarchaeum Nitrosoarchaeum Nitrosopelagicus Nitrosopumilus Nitrososphaera
Candidatus Cenarchaeum massiliensisK8823_413	215	TENA/THI-4 domain-containing protein, pyrroloquinoline-quinone synthase	K06137	IPR028201	OG5_171862	Oxidation-reduction	Cenarchaeum Nitrosoarchaeum Nitrosopelagicus Nitrosopumilus Nitrososphaera
Candidatus Cenarchaeum massiliensisK8823_34	272	Phosphoadenosine phosphosulfate reductase	K00390	IPR011798;IPR004511;IPR002500	OG5_129962	Sulfur metabolism	Cenarchaeum Nitrosoarchaeum Nitrosopelagicus Nitrosopumilus Nitrososphaera
Candidatus Cenarchaeum massiliensisK8823_425	369	hypothetical protein			nmar|YP_001581624	Transcription	Cenarchaeum Nitrosoarchaeum Nitrosopelagicus Nitrosopumilus Nitrososphaera
Candidatus Cenarchaeum massiliensisK8823_426	378	membrane-associated Zn-dependent protease		IPR008915	OG5_135098	Protein modification	Cenarchaeum Nitrosoarchaeum Nitrosopelagicus Nitrosopumilus Nitrososphaera
Candidatus Cenarchaeum massiliensisK8823_427	167	GNAT family N-acetyltransferase	K03789	IPR000182;IPR000182	OG5_128097	Translation	Cenarchaeum Nitrosoarchaeum Nitrosopelagicus Nitrosopumilus Nitrososphaera
Candidatus Cenarchaeum massiliensisK8823_35	364	Thiamine biosynthesis protein			nmar|YP_001581386	Metabolism of cofactors and vitamins	Cenarchaeum Nitrosoarchaeum Nitrosopelagicus Nitrosopumilus Nitrososphaera
Candidatus Cenarchaeum massiliensisK8823_429	276	tRNA (guanine(37)-N1)-methyltransferase Trm5b	K15429	IPR030382;IPR030382	OG5_127369	Translation	Cenarchaeum Nitrosoarchaeum Nitrosopelagicus Nitrosopumilus Nitrososphaera
Candidatus Cenarchaeum massiliensisK8823_430	587	ATPase	K06174	IPR003593;IPR003439;IPR007209;IPR017896;IPR013283;IPR017900;IPR017871;IPR003439;IPR017896;IPR017896;IPR0034	OG5_127149	Nucleotide metabolism	Cenarchaeum Nitrosoarchaeum Nitrosopelagicus Nitrosopumilus Nitrososphaera
Candidatus Cenarchaeum massiliensisK8823_431	138	exosome subunit		IPR002739	OG5_140213	Unknown function	Cenarchaeum Nitrosoarchaeum Nitrosopelagicus Nitrosopumilus Nitrososphaera
Candidatus Cenarchaeum massiliensisK8823_432	204	Ribosomal protein S3Ae	K02984	IPR001593;IPR018281;IPR030838	OG5_126852	Translation	Cenarchaeum Nitrosoarchaeum Nitrosopelagicus Nitrosopumilus Nitrososphaera
Candidatus Cenarchaeum massiliensisK8823_433	420	serine--tRNA ligase	K01875	IPR002317;IPR015866;IPR002314;IPR002317;IPR002317;IPR006195	OG5_126677	Translation	Cenarchaeum Nitrosoarchaeum Nitrosopelagicus Nitrosopumilus Nitrososphaera
Candidatus Cenarchaeum massiliensisK8823_434	68	hypothetical protein			nmar|YP_001582844	Unknown function	Cenarchaeum Nitrosoarchaeum Nitrosopelagicus Nitrosopumilus Nitrososphaera
Candidatus Cenarchaeum massiliensisK8823_435	470	Phosphoesterase DHHA1	K07463	IPR003156	OG5_139284	Misc	Cenarchaeum Nitrosoarchaeum Nitrosopelagicus Nitrosopumilus Nitrososphaera
Candidatus Cenarchaeum massiliensisK8823_436	149	ribosomal protein S15P	K02953	IPR000589;IPR012606;IPR000589;IPR023029	OG5_127089	Translation	Cenarchaeum Nitrosoarchaeum Nitrosopelagicus Nitrosopumilus Nitrososphaera
Candidatus Cenarchaeum massiliensisK8823_437	108	hypothetical exported protein			nmar|YP_001582841	Unknown function	Cenarchaeum Nitrosoarchaeum Nitrosopelagicus Nitrosopumilus Nitrososphaera
Candidatus Cenarchaeum massiliensisK8823_438	175	hypothetical protein			nmar|YP_001582839	Unknown function	Cenarchaeum Nitrosoarchaeum Nitrosopelagicus Nitrosopumilus Nitrososphaera
Candidatus Cenarchaeum massiliensisK8823_36	410	Glycosyl transferase			OG5_155506	Carbohydrate metabolism	Cenarchaeum Nitrosoarchaeum Nitrosopelagicus Nitrosopumilus Nitrososphaera
Candidatus Cenarchaeum massiliensisK8823_439	89	hypothetical membrane protein			nmar|YP_001582840	Unknown function	Cenarchaeum Nitrosoarchaeum Nitrosopelagicus Nitrosopumilus Nitrososphaera
Candidatus Cenarchaeum massiliensisK8823_444	189	ammonia monooxygenase subunit B		IPR006833	nmar|YP_001582837	Nitrogen metabolism	Cenarchaeum Nitrosoarchaeum Nitrosopelagicus Nitrosopumilus Nitrososphaera
Candidatus Cenarchaeum massiliensisK8823_445	184	ammonia monooxygenase subunit C		IPR006980	nmar|YP_001582836	Nitrogen metabolism	Cenarchaeum Nitrosoarchaeum Nitrosopelagicus Nitrosopumilus Nitrososphaera
Candidatus Cenarchaeum massiliensisK8823_446	123	hypothetical protein			nmar|YP_001582835	Nitrogen metabolism	Cenarchaeum Nitrosoarchaeum Nitrosopelagicus Nitrosopumilus Nitrososphaera
Candidatus Cenarchaeum massiliensisK8823_37	94	hypothetical protein			nmar|YP_001581384	Transcription	Cenarchaeum Nitrosoarchaeum Nitrosopelagicus Nitrosopumilus Nitrososphaera
Candidatus Cenarchaeum massiliensisK8823_448	166	ammonia monooxygenase subunit A		IPR024656	nmar|YP_001582834	Nitrogen metabolism	Cenarchaeum Nitrosoarchaeum Nitrosopelagicus Nitrosopumilus Nitrososphaera
Candidatus Cenarchaeum massiliensisK8823_449	285	DNA adenine methylase	K06223	IPR012327;IPR012327;IPR002052	OG5_138839	DNA modification	Cenarchaeum Nitrosoarchaeum Nitrosopelagicus Nitrosopumilus Nitrososphaera
Candidatus Cenarchaeum massiliensisK8823_450	128	hypothetical protein			nmar|YP_001582832	Unknown function	Cenarchaeum Nitrosoarchaeum Nitrosopelagicus Nitrosopumilus Nitrososphaera
Candidatus Cenarchaeum massiliensisK8823_451	320	transcriptional regulator			nmar|YP_001582831	Transcription	Cenarchaeum Nitrosoarchaeum Nitrosopelagicus Nitrosopumilus Nitrososphaera
Candidatus Cenarchaeum massiliensisK8823_452	184	thioredoxin family protein		IPR000866;IPR012336	OG5_136641	Oxidation-reduction	Cenarchaeum Nitrosoarchaeum Nitrosopelagicus Nitrosopumilus Nitrososphaera
Candidatus Cenarchaeum massiliensisK8823_454	154	cytidyltransferase	K02201	IPR004821;IPR004821	OG5_128320	Metabolism of cofactors and vitamins	Cenarchaeum Nitrosoarchaeum Nitrosopelagicus Nitrosopumilus Nitrososphaera
Candidatus Cenarchaeum massiliensisK8823_455	108	hypothetical protein			nmar|YP_001582827	Unknown function	Cenarchaeum Nitrosoarchaeum Nitrosopelagicus Nitrosopumilus Nitrososphaera
Candidatus Cenarchaeum massiliensisK8823_38	120	hypothetical protein			nmar|YP_001581383	Unknown function	Cenarchaeum Nitrosoarchaeum Nitrosopelagicus Nitrosopumilus Nitrososphaera
Candidatus Cenarchaeum massiliensisK8823_456	99	hypothetical protein			nmar|YP_001582825	Unknown function	Cenarchaeum Nitrosoarchaeum Nitrosopelagicus Nitrosopumilus Nitrososphaera
Candidatus Cenarchaeum massiliensisK8823_457	370	tryptophan--tRNA ligase	K01867	IPR002305;IPR002306;IPR002306	OG5_127096	Translation	Cenarchaeum Nitrosoarchaeum Nitrosopelagicus Nitrosopumilus Nitrososphaera
Candidatus Cenarchaeum massiliensisK8823_458	470	phenylalanyl-tRNA synthetase, alpha subunit	K01889	IPR004529;IPR002319;IPR006195	OG5_127036	Translation	Cenarchaeum Nitrosoarchaeum Nitrosopelagicus Nitrosopumilus Nitrososphaera
Candidatus Cenarchaeum massiliensisK8823_459	535	phenylalanine--tRNA ligase subunit beta	K01890	IPR005147;IPR005146;IPR005147;IPR005146;IPR004531;IPR005147	OG5_127490	Translation	Cenarchaeum Nitrosoarchaeum Nitrosopelagicus Nitrosopumilus Nitrososphaera
Candidatus Cenarchaeum massiliensisK8823_460	165	EVE domain-containing protein		IPR002740	OG5_130215	Unknown function	Cenarchaeum Nitrosoarchaeum Nitrosopelagicus Nitrosopumilus Nitrososphaera
Candidatus Cenarchaeum massiliensisK8823_461	79	AsnC family transcriptional regulator		IPR019887	OG5_135100	Transcription	Cenarchaeum Nitrosoarchaeum Nitrosopelagicus Nitrosopumilus Nitrososphaera
Candidatus Cenarchaeum massiliensisK8823_462	185	hypothetical protein			nmar|YP_001582818	Unknown function	Cenarchaeum Nitrosoarchaeum Nitrosopelagicus Nitrosopumilus Nitrososphaera
Candidatus Cenarchaeum massiliensisK8823_465	754	aconitate hydratase	K01681	IPR006248;IPR000573;IPR001030;IPR015937;IPR018136;IPR018136	OG5_126691	Energy	Cenarchaeum Nitrosoarchaeum Nitrosopelagicus Nitrosopumilus Nitrososphaera
Candidatus Cenarchaeum massiliensisK8823_466	175	hypothetical exported protein			nmar|YP_001582814	Unknown function	Cenarchaeum Nitrosoarchaeum Nitrosopelagicus Nitrosopumilus Nitrososphaera
Candidatus Cenarchaeum massiliensisK8823_467	318	pyridoxal biosynthesis lyase	K06215	IPR001852;IPR001852	OG5_128957	Metabolism of cofactors and vitamins	Cenarchaeum Nitrosoarchaeum Nitrosopelagicus Nitrosopumilus Nitrososphaera
Candidatus Cenarchaeum massiliensisK8823_468	199	SNO glutamine amidotransferase	K08681	IPR002161;IPR002161;IPR021196;IPR002161	OG5_129260	Metabolism of cofactors and vitamins	Cenarchaeum Nitrosoarchaeum Nitrosopelagicus Nitrosopumilus Nitrososphaera
Candidatus Cenarchaeum massiliensisK8823_469	216	hypothetical protein			nmar|YP_001581862	Unknown function	Cenarchaeum Nitrosoarchaeum Nitrosopelagicus Nitrosopumilus Nitrososphaera
Candidatus Cenarchaeum massiliensisK8823_470	169	hypothetical protein			nmar|YP_001581707	Unknown function	Cenarchaeum Nitrosoarchaeum Nitrosopelagicus Nitrosopumilus Nitrososphaera
Candidatus Cenarchaeum massiliensisK8823_471	100	Ribosomal protein L12	K02869	IPR022295	OG5_126872	Translation	Cenarchaeum Nitrosoarchaeum Nitrosopelagicus Nitrosopumilus Nitrososphaera
Candidatus Cenarchaeum massiliensisK8823_475	284	ribosomal protein L10	K02941	IPR001790;IPR022909	OG5_127051	Translation	Cenarchaeum Nitrosoarchaeum Nitrosopelagicus Nitrosopumilus Nitrososphaera
Candidatus Cenarchaeum massiliensisK8823_476	219	ribosomal protein L1	K02863	IPR028364;IPR023673;IPR023669	OG5_127114	Translation	Cenarchaeum Nitrosoarchaeum Nitrosopelagicus Nitrosopumilus Nitrososphaera
Candidatus Cenarchaeum massiliensisK8823_477	149	AsnC family transcriptional regulator	K03718	IPR019888;IPR019887;IPR000485	OG5_129697	Transcription	Cenarchaeum Nitrosoarchaeum Nitrosopelagicus Nitrosopumilus Nitrososphaera
Candidatus Cenarchaeum massiliensisK8823_478	160	ribosomal protein L11	K02867	IPR000911;IPR020784;IPR020783;IPR000911;IPR000911	OG5_127022	Translation	Cenarchaeum Nitrosoarchaeum Nitrosopelagicus Nitrosopumilus Nitrososphaera

Candidatus Cenarchaeum massiliensis	K8823_479	156	ribosomal protein L24	K02601	IPR006645;IPR005824;IPR011590;IPR005100;IPR005824;IPR011590	OG5_139275	Transcription	Cenarchaeum Nitrosoarchaeum Nitrosopelagicus Nitrosopumilus Nitrososphaera
Candidatus Cenarchaeum massiliensis	K8823_481	269	D-aminoacyl-tRNA deacylase	K09716	IPR007508	OG5_137380	Misc	Cenarchaeum Nitrosoarchaeum Nitrosopelagicus Nitrosopumilus Nitrososphaera
Candidatus Cenarchaeum massiliensis	K8823_482	100	cysteine methyltransferase	K00567	IPR014048;IPR014048;IPR001497	OG5_127355	DNA modification	Cenarchaeum Nitrosoarchaeum Nitrosopelagicus Nitrosopumilus Nitrososphaera
Candidatus Cenarchaeum massiliensis	K8823_483	151	ribosomal protein L19e		IPR000196	OG5_126940	Translation	Cenarchaeum Nitrosoarchaeum Nitrosopelagicus Nitrosopumilus Nitrososphaera
Candidatus Cenarchaeum massiliensis	K8823_484	134	ribosomal protein L32e	K02912	IPR001515;IPR018263;IPR001515	OG5_126965	Translation	Cenarchaeum Nitrosoarchaeum Nitrosopelagicus Nitrosopumilus Nitrososphaera
Candidatus Cenarchaeum massiliensis	K8823_485	533	phosphoenolpyruvate carboxykinase (ATP)	K01610	IPR001272;IPR001272;IPR001272	OG5_128435	Energy	Cenarchaeum Nitrosoarchaeum Nitrosopelagicus Nitrosopumilus Nitrososphaera
Candidatus Cenarchaeum massiliensis	K8823_487	350	alcohol dehydrogenase	K13953	IPR020843;IPR013154;IPR013149;IPR002085;IPR002328	OG5_136190	Oxidation-reduction	Cenarchaeum Nitrosoarchaeum Nitrosopelagicus Nitrosopumilus Nitrososphaera
Candidatus Cenarchaeum massiliensis	K8823_489	222	chromosome segregation protein ScpA			OG5_155479	Cell & development	Cenarchaeum Nitrosoarchaeum Nitrosopelagicus Nitrosopumilus Nitrososphaera
Candidatus Cenarchaeum massiliensis	K8823_491	269	metallophosphoesterase		IPR004843	OG5_134492	Misc	Cenarchaeum Nitrosoarchaeum Nitrosopelagicus Nitrosopumilus Nitrososphaera
Candidatus Cenarchaeum massiliensis	K8823_492	344	isocitrate dehydrogenase	K00052	IPR024084;IPR001804	nmar|YP_001582713	Energy	Cenarchaeum Nitrosoarchaeum Nitrosopelagicus Nitrosopumilus Nitrososphaera
Candidatus Cenarchaeum massiliensis	K8823_493	171	methylase			OG5_127713	Protein modification	Cenarchaeum Nitrosoarchaeum Nitrosopelagicus Nitrosopumilus Nitrososphaera
Candidatus Cenarchaeum massiliensis	K8823_494	228	ribosomal RNA adenine methyltransferase	K02528	IPR020598;IPR001737;IPR001737;IPR001737	OG5_126745	RNA modification	Cenarchaeum Nitrosoarchaeum Nitrosopelagicus Nitrosopumilus Nitrososphaera
Candidatus Cenarchaeum massiliensis	K8823_495	188	hypothetical protein	K07572	IPR007003	OG5_139294	Unknown function	Cenarchaeum Nitrosoarchaeum Nitrosopelagicus Nitrosopumilus Nitrososphaera
Candidatus Cenarchaeum massiliensis	K8823_496	108	RNA polymerase Rpb4		IPR005574	OG5_141337	Transcription	Cenarchaeum Nitrosoarchaeum Nitrosopelagicus Nitrosopumilus Nitrososphaera
Candidatus Cenarchaeum massiliensis	K8823_497	99	ribosomal protein L21e	K02889	IPR001147;IPR022856	OG5_126766	Translation	Cenarchaeum Nitrosoarchaeum Nitrosopelagicus Nitrosopumilus Nitrososphaera
Candidatus Cenarchaeum massiliensis	K8823_498	382	DNA repair and recombination protein RadA	K04483	IPR003593;IPR011938;IPR013632;IPR011938;IPR020587;IPR020588	OG5_126834	DNA modification	Cenarchaeum Nitrosoarchaeum Nitrosopelagicus Nitrosopumilus Nitrososphaera
Candidatus Cenarchaeum massiliensis	K8823_499	106	DNA-binding protein			OG5_140194	DNA modification	Cenarchaeum Nitrosoarchaeum Nitrosopelagicus Nitrosopumilus Nitrososphaera
Candidatus Cenarchaeum massiliensis	K8823_501	205	hypothetical membrane protein			OG5_193500	Misc	Cenarchaeum Nitrosoarchaeum Nitrosopelagicus Nitrosopumilus Nitrososphaera
Candidatus Cenarchaeum massiliensis	K8823_502	103	hypothetical protein			nmar|YP_001581842	Unknown function	Cenarchaeum Nitrosoarchaeum Nitrosopelagicus Nitrosopumilus Nitrososphaera
Candidatus Cenarchaeum massiliensis	K8823_503	311	delta-aminolevulinic acid dehydratase	K01698	IPR001731;IPR001731;IPR001731;IPR030656	OG5_127486	Metabolism of cofactors and vitamins	Cenarchaeum Nitrosoarchaeum Nitrosopelagicus Nitrosopumilus Nitrososphaera
Candidatus Cenarchaeum massiliensis	K8823_504	420	Glutamyl-tRNA reductase	K02492	IPR015896;IPR006151;IPR000343;IPR015895;IPR018214;IPR000343	OG5_130465	Metabolism of cofactors and vitamins	Cenarchaeum Nitrosoarchaeum Nitrosopelagicus Nitrosopumilus Nitrososphaera
Candidatus Cenarchaeum massiliensis	K8823_505	218	siroheme synthase/precorrin-2 oxidase (MET8)		IPR006367;IPR006367;IPR028281	OG5_131637	Metabolism of cofactors and vitamins	Cenarchaeum Nitrosoarchaeum Nitrosopelagicus Nitrosopumilus Nitrososphaera
Candidatus Cenarchaeum massiliensis	K8823_506	333	transcriptional regulator AsnC family		IPR019888	OG5_146708	Transcription	Cenarchaeum Nitrosoarchaeum Nitrosopelagicus Nitrosopumilus Nitrososphaera
Candidatus Cenarchaeum massiliensis	K8823_508	331	oxidoreductase		IPR023210;IPR001395	OG5_126648	Misc	Cenarchaeum Nitrosoarchaeum Nitrosopelagicus Nitrosopumilus Nitrososphaera
Candidatus Cenarchaeum massiliensis	K8823_510	234	chlorite dismutase		IPR010644	OG5_139600	Oxidation-reduction	Cenarchaeum Nitrosoarchaeum Nitrosopelagicus Nitrosopumilus Nitrososphaera
Candidatus Cenarchaeum massiliensis	K8823_512	401	geranylgeranyl reductase	K17830		OG5_130321	Oxidation-reduction	Cenarchaeum Nitrosoarchaeum Nitrosopelagicus Nitrosopumilus Nitrososphaera
Candidatus Cenarchaeum massiliensis	K8823_513	440	Sodium/hydrogen exchanger	K03455	IPR006153	OG5_128017	Transport	Cenarchaeum Nitrosoarchaeum Nitrosopelagicus Nitrosopumilus Nitrososphaera
Candidatus Cenarchaeum massiliensis	K8823_514	144	hypothetical protein			nmar|YP_001581781	Unknown function	Cenarchaeum Nitrosoarchaeum Nitrosopelagicus Nitrosopumilus Nitrososphaera
Candidatus Cenarchaeum massiliensis	K8823_515	164	ribosomal protein L16/L10E	K02866	IPR016180;IPR018255	OG5_126951	Translation	Cenarchaeum Nitrosoarchaeum Nitrosopelagicus Nitrosopumilus Nitrososphaera
Candidatus Cenarchaeum massiliensis	K8823_516	193	tRNA intron endonuclease	K01170	IPR006677;IPR006676;IPR006678	OG5_128736	Translation	Cenarchaeum Nitrosoarchaeum Nitrosopelagicus Nitrosopumilus Nitrososphaera
Candidatus Cenarchaeum massiliensis	K8823_519	264	hypothetical protein		IPR007497	OG5_136663	Unknown function	Cenarchaeum Nitrosoarchaeum Nitrosopelagicus Nitrosopumilus Nitrososphaera
Candidatus Cenarchaeum massiliensis	K8823_520	186	5-(carboxyamino)imidazole ribonucleotide mutase	K01588	IPR000031;IPR000031;IPR000031;IPR000031	OG5_127668	Nucleotide metabolism	Cenarchaeum Nitrosoarchaeum Nitrosopelagicus Nitrosopumilus Nitrososphaera
Candidatus Cenarchaeum massiliensis	K8823_522	295	hypothetical protein			nmar|YP_001581800	Unknown function	Cenarchaeum Nitrosoarchaeum Nitrosopelagicus Nitrosopumilus Nitrososphaera
Candidatus Cenarchaeum massiliensis	K8823_523	485	hypothetical protein			OG5_151395	Carbohydrate metabolism	Cenarchaeum Nitrosoarchaeum Nitrosopelagicus Nitrosopumilus Nitrososphaera
Candidatus Cenarchaeum massiliensis	K8823_525	186	hypothetical protein			nmar|YP_001581803	Misc	Cenarchaeum Nitrosoarchaeum Nitrosopelagicus Nitrosopumilus Nitrososphaera
Candidatus Cenarchaeum massiliensis	K8823_526	212	hypothetical protein			nmar|YP_001581804	Unknown function	Cenarchaeum Nitrosoarchaeum Nitrosopelagicus Nitrosopumilus Nitrososphaera
Candidatus Cenarchaeum massiliensis	K8823_529	330	ABC transporter ATP-binding protein	K01990	IPR003593;IPR005894;IPR003439;IPR017871;IPR003439	OG5_126568	Transport	Cenarchaeum Nitrosoarchaeum Nitrosopelagicus Nitrosopumilus Nitrososphaera
Candidatus Cenarchaeum massiliensis	K8823_530	258	ABC transporter	K01992	IPR013525;IPR000412	OG5_131341	Transport	Cenarchaeum Nitrosoarchaeum Nitrosopelagicus Nitrosopumilus Nitrososphaera
Candidatus Cenarchaeum massiliensis	K8823_534	273	Transglutaminase-like superfamily protein		IPR013026	OG5_140373	Unknown function	Cenarchaeum Nitrosoarchaeum Nitrosopelagicus Nitrosopumilus Nitrososphaera
Candidatus Cenarchaeum massiliensis	K8823_536	530	DNA topoisomerase I B	K03163	IPR013499;IPR008336;IPR025834;IPR013500	OG5_127702	DNA modification	Cenarchaeum Nitrosoarchaeum Nitrosopelagicus Nitrosopumilus Nitrososphaera
Candidatus Cenarchaeum massiliensis	K8823_539	304	short-chain dehydrogenase/reductase		IPR002198;IPR020904	OG5_126778	Oxidation-reduction	Cenarchaeum Nitrosoarchaeum Nitrosopelagicus Nitrosopumilus Nitrososphaera
Candidatus Cenarchaeum massiliensis	K8823_542	258	DNA methylase N-4/N-6 domain-containing protein (ccrM)	K13581	IPR002941;IPR002052	OG5_135744	DNA modification	Cenarchaeum Nitrosoarchaeum Nitrosopelagicus Nitrosopumilus Nitrososphaera
Candidatus Cenarchaeum massiliensis	K8823_543	109	sec-independent translocation protein (mttA/Hcf106, tatA)		IPR003369	OG5_129693	Transport	Cenarchaeum Nitrosoarchaeum Nitrosopelagicus Nitrosopumilus Nitrososphaera
Candidatus Cenarchaeum massiliensis	K8823_544	384	major facilitator transporter family protein	K19576	IPR011701;IPR020846	OG5_168755	Transport	Cenarchaeum Nitrosoarchaeum Nitrosopelagicus Nitrosopumilus Nitrososphaera
Candidatus Cenarchaeum massiliensis	K8823_545	229	methylase	K03183	IPR004033	OG5_127211	Metabolism of cofactors and vitamins	Cenarchaeum Nitrosoarchaeum Nitrosopelagicus Nitrosopumilus Nitrososphaera
Candidatus Cenarchaeum massiliensis	K8823_546	241	proteasome subunit alpha	K03432	IPR000426;IPR001353;IPR000426;IPR023332	OG5_127424	Protein modification	Cenarchaeum Nitrosoarchaeum Nitrosopelagicus Nitrosopumilus Nitrososphaera
Candidatus Cenarchaeum massiliensis	K8823_548	424	glutamate dehydrogenase	K00261	IPR006096;IPR006096;IPR006097;IPR006095	OG5_126857	Nitrogen metabolism	Cenarchaeum Nitrosoarchaeum Nitrosopelagicus Nitrosopumilus Nitrososphaera
Candidatus Cenarchaeum massiliensis	K8823_550	704	acyl-CoA synthetase		IPR003781;IPR003781;IPR011761	OG5_146682	Metabolism of cofactors and vitamins	Cenarchaeum Nitrosoarchaeum Nitrosopelagicus Nitrosopumilus Nitrososphaera
Candidatus Cenarchaeum massiliensis	K8823_551	287	3-hydroxyisobutyrate dehydrogenase	K00020	IPR029154;IPR006115	OG5_126809	Amino acid metabolism	Cenarchaeum Nitrosoarchaeum Nitrosopelagicus Nitrosopumilus Nitrososphaera
Candidatus Cenarchaeum massiliensis	K8823_552	128	hypothetical protein			nmar|YP_001581970	Unknown function	Cenarchaeum Nitrosoarchaeum Nitrosopelagicus Nitrosopumilus Nitrososphaera
Candidatus Cenarchaeum massiliensis	K8823_554	306	Iron siderophore/cobalamin periplasmic-binding domain-containing protein	K02016	IPR002491;IPR002491	OG5_135746	Transport	Cenarchaeum Nitrosoarchaeum Nitrosopelagicus Nitrosopumilus Nitrososphaera
Candidatus Cenarchaeum massiliensis	K8823_555	384	hypothetical protein		IPR006978;IPR006979	OG5_137387	Unknown function	Cenarchaeum Nitrosoarchaeum Nitrosopelagicus Nitrosopumilus Nitrososphaera
Candidatus Cenarchaeum massiliensis	K8823_560	311	hypothetical membrane protein		IPR027561;IPR026392	nmar|YP_001581975	Unknown function	Cenarchaeum Nitrosoarchaeum Nitrosopelagicus Nitrosopumilus Nitrososphaera
Candidatus Cenarchaeum massiliensis	K8823_561	364	DNA polymerase IV	K02346	IPR001126;IPR017961;IPR024728;IPR001126;IPR022880	OG5_127393	DNA modification	Cenarchaeum Nitrosoarchaeum Nitrosopelagicus Nitrosopumilus Nitrososphaera
Candidatus Cenarchaeum massiliensis	K8823_562	84	hypothetical protein			nmar|YP_001581977	Unknown function	Cenarchaeum Nitrosoarchaeum Nitrosopelagicus Nitrosopumilus Nitrososphaera
Candidatus Cenarchaeum massiliensis	K8823_563	479	DNA-RNA-binding protein Alba	K03622	IPR002775	OG5_139287	Misc	Cenarchaeum Nitrosoarchaeum Nitrosopelagicus Nitrosopumilus Nitrososphaera
Candidatus Cenarchaeum massiliensis	K8823_564	218	lysine transporter LysE		IPR001123	OG5_137390	Transport	Cenarchaeum Nitrosoarchaeum Nitrosopelagicus Nitrosopumilus Nitrososphaera
Candidatus Cenarchaeum massiliensis	K8823_567	209	pyridoxamine 5'-phosphate oxidase		IPR024747	OG5_139599	Oxidation-reduction	Cenarchaeum Nitrosoarchaeum Nitrosopelagicus Nitrosopumilus Nitrososphaera
Candidatus Cenarchaeum massiliensis	K8823_574	365	radical SAM protein	K11784	IPR007197;IPR020050	OG5_130963	Metabolism of cofactors and vitamins	Cenarchaeum Nitrosoarchaeum Nitrosopelagicus Nitrosopumilus Nitrososphaera
Candidatus Cenarchaeum massiliensis	K8823_575	271	Menaquinone biosynthesis protein	K11785	IPR003773;IPR030869	OG5_141278	Metabolism of cofactors and vitamins	Cenarchaeum Nitrosoarchaeum Nitrosopelagicus Nitrosopumilus Nitrososphaera
Candidatus Cenarchaeum massiliensis	K8823_577	730	Elongation factor 2	K03234	IPR005517;IPR000640;IPR005517;IPR004161;IPR005225;IPR000640;IPR009022;IPR004543;IPR031157;IPR000795;IPR004[illegible]	OG5_126923	Translation	Cenarchaeum Nitrosoarchaeum Nitrosopelagicus Nitrosopumilus Nitrososphaera
Candidatus Cenarchaeum massiliensis	K8823_578	139	hypothetical protein			OG5_137677	Unknown function	Cenarchaeum Nitrosoarchaeum Nitrosopelagicus Nitrosopumilus Nitrososphaera
Candidatus Cenarchaeum massiliensis	K8823_580	264	hypothetical membrane protein			nmar|YP_001582097	Unknown function	Cenarchaeum Nitrosoarchaeum Nitrosopelagicus Nitrosopumilus Nitrososphaera
Candidatus Cenarchaeum massiliensis	K8823_583	140	Bifunctional nuclease		IPR003729;IPR003729	nmar|YP_001582099	Misc	Cenarchaeum Nitrosoarchaeum Nitrosopelagicus Nitrosopumilus Nitrososphaera
Candidatus Cenarchaeum massiliensis	K8823_584	360	formate--phosphoribosylaminoimidazolecarboxamide ligase	K06863	IPR009720;IPR010672	OG5_137370	Nucleotide metabolism	Cenarchaeum Nitrosoarchaeum Nitrosopelagicus Nitrosopumilus Nitrososphaera
Candidatus Cenarchaeum massiliensis	K8823_47	177	Nucleoside kinase	K18532		OG5_127564	Nucleotide metabolism	Cenarchaeum Nitrosoarchaeum Nitrosopelagicus Nitrosopumilus Nitrososphaera
Candidatus Cenarchaeum massiliensis	K8823_585	176	Rossmann fold nucleotide-binding protein	K06966	IPR005268	OG5_137354	Unknown function	Cenarchaeum Nitrosoarchaeum Nitrosopelagicus Nitrosopumilus Nitrososphaera
Candidatus Cenarchaeum massiliensis	K8823_586	380	3-hydroxyacyl-CoA dehydrogenase	K15016	IPR006108;IPR006176	OG5_127943	Lipid metabolism	Cenarchaeum Nitrosoarchaeum Nitrosopelagicus Nitrosopumilus Nitrososphaera
Candidatus Cenarchaeum massiliensis	K8823_588	89	hypothetical membrane protein			nmar|YP_001582360	Unknown function	Cenarchaeum Nitrosoarchaeum Nitrosopelagicus Nitrosopumilus Nitrososphaera
Candidatus Cenarchaeum massiliensis	K8823_589	139	HIT family protein	K02503	IPR001310;IPR001310;IPR011146	OG5_126794	RNA modification	Cenarchaeum Nitrosoarchaeum Nitrosopelagicus Nitrosopumilus Nitrososphaera
Candidatus Cenarchaeum massiliensis	K8823_590	210	molecular chaperone		IPR001623;IPR001623;IPR001623;IPR017896	OG5_145894	Stress & defense	Cenarchaeum Nitrosoarchaeum Nitrosopelagicus Nitrosopumilus Nitrososphaera
Candidatus Cenarchaeum massiliensis	K8823_591	149	cupin		IPR006045	OG5_132292	Misc	Cenarchaeum Nitrosoarchaeum Nitrosopelagicus Nitrosopumilus Nitrososphaera
Candidatus Cenarchaeum massiliensis	K8823_593	269	TPR-repeat protein	K09667	IPR019734;IPR001440;IPR019734;IPR019734;IPR013026;IPR019734;IPR019734;IPR019734;IPR019734;IPR019734	OG5_127266	Unknown function	Cenarchaeum Nitrosoarchaeum Nitrosopelagicus Nitrosopumilus Nitrososphaera
Candidatus Cenarchaeum massiliensis	K8823_48	505	tRNA-guanine transglycosylase	K18779	IPR004804;IPR002616;IPR002616;IPR002616	OG5_126946	Translation	Cenarchaeum Nitrosoarchaeum Nitrosopelagicus Nitrosopumilus Nitrososphaera
Candidatus Cenarchaeum massiliensis	K8823_594	448	Aminotransferase class-III	K01845	IPR005814;IPR005814;IPR005814	OG5_129427	Metabolism of cofactors and vitamins	Cenarchaeum Nitrosoarchaeum Nitrosopelagicus Nitrosopumilus Nitrososphaera
Candidatus Cenarchaeum massiliensis	K8823_597	115	hypothetical protein			OG5_137677	Unknown function	Cenarchaeum Nitrosoarchaeum Nitrosopelagicus Nitrosopumilus Nitrososphaera
Candidatus Cenarchaeum massiliensis	K8823_599	125	hypothetical protein			OG5_137677	Unknown function	Cenarchaeum Nitrosoarchaeum Nitrosopelagicus Nitrosopumilus Nitrososphaera
Candidatus Cenarchaeum massiliensis	K8823_600	82	hypothetical protein		IPR019887	OG5_135100	Transcription	Cenarchaeum Nitrosoarchaeum Nitrosopelagicus Nitrosopumilus Nitrososphaera
Candidatus Cenarchaeum massiliensis	K8823_49	100	4Fe-4S ferredoxin		IPR017896;IPR017896;IPR017896	OG5_159009	Misc	Cenarchaeum Nitrosoarchaeum Nitrosopelagicus Nitrosopumilus Nitrososphaera
Candidatus Cenarchaeum massiliensis	K8823_603	140	universal stress protein		IPR006016	OG5_126997	Stress & defense	Cenarchaeum Nitrosoarchaeum Nitrosopelagicus Nitrosopumilus Nitrososphaera
Candidatus Cenarchaeum massiliensis	K8823_606	560	Thiamine pyrophosphate-requiring enzyme	K01652	IPR011766;IPR012846;IPR012001;IPR012000;IPR000399	OG5_126899	Amino acid metabolism	Cenarchaeum Nitrosoarchaeum Nitrosopelagicus Nitrosopumilus Nitrososphaera
Candidatus Cenarchaeum massiliensis	K8823_607	165	acetolactate synthase small subunit	K01653	IPR004789;IPR019455;IPR002912	OG5_129345	Amino acid metabolism	Cenarchaeum Nitrosoarchaeum Nitrosopelagicus Nitrosopumilus Nitrososphaera
Candidatus Cenarchaeum massiliensis	K8823_608	307	isopropylmalate/citramalate/homocitrate synthase (leuA)	K01649	IPR000891;IPR002034;IPR000891	OG5_128072	Misc	Cenarchaeum Nitrosoarchaeum Nitrosopelagicus Nitrosopumilus Nitrososphaera
Candidatus Cenarchaeum massiliensis	K8823_609	186	2-isopropylmalate synthase	K01649	IPR013709;IPR013709	OG5_128072	Misc	Cenarchaeum Nitrosoarchaeum Nitrosopelagicus Nitrosopumilus Nitrososphaera
Candidatus Cenarchaeum massiliensis	K8823_610	131	3-isopropylmalate dehydrogenase	K00052	IPR024084;IPR001804	OG5_126870	Amino acid metabolism	Cenarchaeum Nitrosoarchaeum Nitrosopelagicus Nitrosopumilus Nitrososphaera
Candidatus Cenarchaeum massiliensis	K8823_611	211	isopropylmalate/isohomocitrate dehydrogenase	K00052	IPR024084;IPR001804;IPR019818	OG5_126870	Amino acid metabolism	Cenarchaeum Nitrosoarchaeum Nitrosopelagicus Nitrosopumilus Nitrososphaera
Candidatus Cenarchaeum massiliensis	K8823_612	135	aspartyl-tRNA synthetase	K01876	IPR004364;IPR018150;IPR006195	OG5_127227	Translation	Cenarchaeum Nitrosoarchaeum Nitrosopelagicus Nitrosopumilus Nitrososphaera
Candidatus Cenarchaeum massiliensis	K8823_50	70	hypothetical protein			nmar|YP_001582872	Unknown function	Cenarchaeum Nitrosoarchaeum Nitrosopelagicus Nitrosopumilus Nitrososphaera
Candidatus Cenarchaeum massiliensis	K8823_613	315	aspartate--tRNA(Asn) ligase	K01876	IPR004365;IPR004364;IPR018150;IPR006195	OG5_127227	Translation	Cenarchaeum Nitrosoarchaeum Nitrosopelagicus Nitrosopumilus Nitrososphaera
Candidatus Cenarchaeum massiliensis	K8823_614	312	oligopeptide/dipeptide ABC transporter ATPase	K02031	IPR003593;IPR003439;IPR013563;IPR013563;IPR017871;IPR003439	OG5_128716	Transport	Cenarchaeum Nitrosoarchaeum Nitrosopelagicus Nitrosopumilus Nitrososphaera
Candidatus Cenarchaeum massiliensis	K8823_615	124	hypothetical protein			OG5_137677	Unknown function	Cenarchaeum Nitrosoarchaeum Nitrosopelagicus Nitrosopumilus Nitrososphaera
Candidatus Cenarchaeum massiliensis	K8823_616	162	SNARE associated protein			OG5_152577	Misc	Cenarchaeum Nitrosoarchaeum Nitrosopelagicus Nitrosopumilus Nitrososphaera
Candidatus Cenarchaeum massiliensis	K8823_617	391	uracil-DNA glycosylase	K03652	IPR005122;IPR003180;IPR005273;IPR005122;IPR003180;IPR003180;IPR003180	OG5_130143	DNA modification	Cenarchaeum Nitrosoarchaeum Nitrosopelagicus Nitrosopumilus Nitrososphaera
Candidatus Cenarchaeum massiliensis	K8823_619	614	Type II secretion system protein E	K06865	IPR002716;IPR001482;IPR004088;IPR004088	OG5_139256	Metabolism of cofactors and vitamins	Cenarchaeum Nitrosoarchaeum Nitrosopelagicus Nitrosopumilus Nitrososphaera
Candidatus Cenarchaeum massiliensis	K8823_620	154	iron dependent repressor, DtxR family	K03709	IPR022689;IPR022687;IPR001367;IPR022687	OG5_132027	Transcription	Cenarchaeum Nitrosoarchaeum Nitrosopelagicus Nitrosopumilus Nitrososphaera
Candidatus Cenarchaeum massiliensis	K8823_51	424	Beta-lactamase domain protein	K07577	IPR022712;IPR001279;IPR011108;IPR022712	OG5_141315	RNA modification	Cenarchaeum Nitrosoarchaeum Nitrosopelagicus Nitrosopumilus Nitrososphaera
Candidatus Cenarchaeum massiliensis	K8823_622	164	hypothetical exported protein	K09005	IPR003795	OG5_145046	Unknown function	Cenarchaeum Nitrosoarchaeum Nitrosopelagicus Nitrosopumilus Nitrososphaera
Candidatus Cenarchaeum massiliensis	K8823_623	251	permease	K07090	IPR002781;IPR002781	OG5_130200	Transport	Cenarchaeum Nitrosoarchaeum Nitrosopelagicus Nitrosopumilus Nitrososphaera

Candidatus Cenarchaeum massiliensisK8823_624	588	alcohol dehydrogenase	K00059	IPR002198	OG5_179511	Oxidation-reduction	Cenarchaeum Nitrosoarchaeum Nitrosopelagicus Nitrosopumilus Nitrososphaera
Candidatus Cenarchaeum massiliensisK8823_625	166	hypothetical membrane protein			nmar|YP_001581974	Unknown function	Cenarchaeum Nitrosoarchaeum Nitrosopelagicus Nitrosopumilus Nitrososphaera
Candidatus Cenarchaeum massiliensisK8823_628	593	pepF/M3 family oligoendopeptidase (pepF, pepB)	K08602	IPR013647;IPR001567	OG5_134573	Protein modification	Cenarchaeum Nitrosoarchaeum Nitrosopelagicus Nitrosopumilus Nitrososphaera
Candidatus Cenarchaeum massiliensisK8823_630	191	pyruvoyl-dependent arginine decarboxylase	K02626	IPR002724;IPR002724;IPR002724;IPR002724	OG5_152625	Amino acid metabolism	Cenarchaeum Nitrosoarchaeum Nitrosopelagicus Nitrosopumilus Nitrososphaera
Candidatus Cenarchaeum massiliensisK8823_52	280	DNA repair protein	K03660	IPR003265;IPR003265	OG5_127994	DNA modification	Cenarchaeum Nitrosoarchaeum Nitrosopelagicus Nitrosopumilus Nitrososphaera
Candidatus Cenarchaeum massiliensisK8823_635	348	flap endonuclease 1	K04799	IPR008918;IPR006086;IPR006085;IPR006085;IPR006086;IPR019973;IPR006084;IPR019974;IPR023426	OG5_127472	DNA modification	Cenarchaeum Nitrosoarchaeum Nitrosopelagicus Nitrosopumilus Nitrososphaera
Candidatus Cenarchaeum massiliensisK8823_636	363	myo-inositol-1-phosphate synthase (INO1)	K01858	IPR013021;IPR002587	OG5_139953	Lipid metabolism	Cenarchaeum Nitrosoarchaeum Nitrosopelagicus Nitrosopumilus Nitrososphaera
Candidatus Cenarchaeum massiliensisK8823_53	90	DNA-binding protein		IPR002836;IPR002836	OG5_127711	Unknown function	Cenarchaeum Nitrosoarchaeum Nitrosopelagicus Nitrosopumilus Nitrososphaera
Candidatus Cenarchaeum massiliensisK8823_637	207	DNA-directed RNA polymerase	K03047	IPR011263;IPR009025;IPR011262;IPR001514	OG5_127622	Transcription	Cenarchaeum Nitrosoarchaeum Nitrosopelagicus Nitrosopumilus Nitrososphaera
Candidatus Cenarchaeum massiliensisK8823_638	116	ribosomal protein L18e/L15	K02883	IPR021131;IPR001196	OG5_139272	Translation	Cenarchaeum Nitrosoarchaeum Nitrosopelagicus Nitrosopumilus Nitrososphaera
Candidatus Cenarchaeum massiliensisK8823_639	149	ribosomal protein L13	K02872	IPR005755;IPR005822;IPR005755;IPR005822;IPR023563;IPR005822	OG5_126957	Translation	Cenarchaeum Nitrosoarchaeum Nitrosopelagicus Nitrosopumilus Nitrososphaera
Candidatus Cenarchaeum massiliensisK8823_640	149	ribosomal protein S9	K02996	IPR000754;IPR000754;IPR020574	OG5_126930	Translation	Cenarchaeum Nitrosoarchaeum Nitrosopelagicus Nitrosopumilus Nitrososphaera
Candidatus Cenarchaeum massiliensisK8823_642	202	3-isopropylmalate dehydratase small subunit	K01704	IPR000573;IPR004431;IPR015937;IPR004431	OG5_130205	Amino acid metabolism	Cenarchaeum Nitrosoarchaeum Nitrosopelagicus Nitrosopumilus Nitrososphaera
Candidatus Cenarchaeum massiliensisK8823_643	471	3-isopropylmalate dehydratase large subunit	K01703	IPR004430;IPR001030;IPR015937;IPR018136;IPR018136;IPR004430	OG5_127864	Amino acid metabolism	Cenarchaeum Nitrosoarchaeum Nitrosopelagicus Nitrosopumilus Nitrososphaera
Candidatus Cenarchaeum massiliensisK8823_644	172	3-isopropylmalate dehydratase small subunit	K01704	IPR000573;IPR011827;IPR015937	OG5_130205	Amino acid metabolism	Cenarchaeum Nitrosoarchaeum Nitrosopelagicus Nitrosopumilus Nitrososphaera
Candidatus Cenarchaeum massiliensisK8823_645	423	3-isopropylmalate dehydratase	K01703	IPR011826;IPR006251;IPR001030;IPR015937;IPR011826	OG5_127864	Amino acid metabolism	Cenarchaeum Nitrosoarchaeum Nitrosopelagicus Nitrosopumilus Nitrososphaera
Candidatus Cenarchaeum massiliensisK8823_646	85	hypothetical protein		IPR018592	OG5_241583	Unknown function	Cenarchaeum Nitrosoarchaeum Nitrosopelagicus Nitrosopumilus Nitrososphaera
Candidatus Cenarchaeum massiliensisK8823_54	150	Ribosomal protein S19e	K02966	IPR001266;IPR001266;IPR001266;IPR027548	OG5_127080	Translation	Cenarchaeum Nitrosoarchaeum Nitrosopelagicus Nitrosopumilus Nitrososphaera
Candidatus Cenarchaeum massiliensisK8823_647	679	V-type H(+)-translocating pyrophosphatase	K15987	IPR004131;IPR004131;IPR004131	OG5_128905	Oxidation-reduction	Cenarchaeum Nitrosoarchaeum Nitrosopelagicus Nitrosopumilus Nitrososphaera
Candidatus Cenarchaeum massiliensisK8823_649	177	hypothetical protein	K09735	IPR007164;IPR007164	OG5_141331	Unknown function	Cenarchaeum Nitrosoarchaeum Nitrosopelagicus Nitrosopumilus Nitrososphaera
Candidatus Cenarchaeum massiliensisK8823_650	371	chromosome partitioning protein	K03593	IPR019591;IPR002744;IPR019591	OG5_126620	Transcription	Cenarchaeum Nitrosoarchaeum Nitrosopelagicus Nitrosopumilus Nitrososphaera
Candidatus Cenarchaeum massiliensisK8823_653	91	inositol-phosphate phosphatase	K01092	IPR000760	OG5_126810	Stress & defense	Cenarchaeum Nitrosoarchaeum Nitrosopelagicus Nitrosopumilus Nitrososphaera
Candidatus Cenarchaeum massiliensisK8823_55	98	RNase P subunit		IPR007175;IPR016432	OG5_144460	RNA modification	Cenarchaeum Nitrosoarchaeum Nitrosopelagicus Nitrosopumilus Nitrososphaera
Candidatus Cenarchaeum massiliensisK8823_56	1475	hypothetical protein			OG5_151931	Protein modification	Cenarchaeum Nitrosoarchaeum Nitrosopelagicus Nitrosopumilus Nitrososphaera
Candidatus Cenarchaeum massiliensisK8823_665	397	ATPase		IPR003593;IPR007330;IPR003959;IPR007330	OG5_127508	Transport	Cenarchaeum Nitrosoarchaeum Nitrosopelagicus Nitrosopumilus Nitrososphaera
Candidatus Cenarchaeum massiliensisK8823_667	1034	carbamoyl phosphate synthase large subunit	K01955	IPR011607;IPR005480;IPR005479;IPR006275;IPR005480;IPR011607;IPR005479;IPR005479;IPR011761;IPR011761	OG5_126835	Nucleotide metabolism	Cenarchaeum Nitrosoarchaeum Nitrosopelagicus Nitrosopumilus Nitrososphaera
Candidatus Cenarchaeum massiliensisK8823_57	100	(2Fe-2S)-binding protein	K05710	IPR017941;IPR017941	OG5_133960	Oxidation-reduction	Cenarchaeum Nitrosoarchaeum Nitrosopelagicus Nitrosopumilus Nitrososphaera
Candidatus Cenarchaeum massiliensisK8823_668	249	phosphoesterase	K06953	IPR004843	OG5_135389	Misc	Cenarchaeum Nitrosoarchaeum Nitrosopelagicus Nitrosopumilus Nitrososphaera
Candidatus Cenarchaeum massiliensisK8823_669	97	hypothetical protein			OG5_133488	Unknown function	Cenarchaeum Nitrosoarchaeum Nitrosopelagicus Nitrosopumilus Nitrososphaera
Candidatus Cenarchaeum massiliensisK8823_670	98	hypothetical protein			nmar|YP_001582415	Unknown function	Cenarchaeum Nitrosoarchaeum Nitrosopelagicus Nitrosopumilus Nitrososphaera
Candidatus Cenarchaeum massiliensisK8823_671	99	ATP synthase F subunit		IPR008218	OG5_165849	Transport	Cenarchaeum Nitrosoarchaeum Nitrosopelagicus Nitrosopumilus Nitrososphaera
Candidatus Cenarchaeum massiliensisK8823_672	387	hypothetical exported protein			STG_0027	Unknown function	Cenarchaeum Nitrosoarchaeum Nitrosopelagicus Nitrosopumilus Nitrososphaera
Candidatus Cenarchaeum massiliensisK8823_673	360	Alcohol dehydrogenase	K00008	IPR020843;IPR013154;IPR013149;IPR002085;IPR002328	OG5_126928	Oxidation-reduction	Cenarchaeum Nitrosoarchaeum Nitrosopelagicus Nitrosopumilus Nitrososphaera
Candidatus Cenarchaeum massiliensisK8823_674	167	hypothetical protein	K09140	IPR007177;IPR022968;IPR007177	OG5_127734	Translation	Cenarchaeum Nitrosoarchaeum Nitrosopelagicus Nitrosopumilus Nitrososphaera
Candidatus Cenarchaeum massiliensisK8823_675	309	Fructose-bisphosphate aldolase	K08321	IPR002915	OG5_135382	Energy	Cenarchaeum Nitrosoarchaeum Nitrosopelagicus Nitrosopumilus Nitrososphaera
Candidatus Cenarchaeum massiliensisK8823_676	136	iron-sulfur cluster assembly accessory protein (iscA, ISCA1)	K15724	IPR016092;IPR000361	OG5_127463	Misc	Cenarchaeum Nitrosoarchaeum Nitrosopelagicus Nitrosopumilus Nitrososphaera
Candidatus Cenarchaeum massiliensisK8823_58	89	hypothetical protein		IPR005354;IPR005354;IPR005354	OG5_142707	Unknown function	Cenarchaeum Nitrosoarchaeum Nitrosopelagicus Nitrosopumilus Nitrososphaera
Candidatus Cenarchaeum massiliensisK8823_677	377	8-amino-7-oxononanoate synthase protein	K00652	IPR004839	OG5_126783	Metabolism of cofactors and vitamins	Cenarchaeum Nitrosoarchaeum Nitrosopelagicus Nitrosopumilus Nitrososphaera
Candidatus Cenarchaeum massiliensisK8823_678	327	Biotin synthase	K01012	IPR006638;IPR010722;IPR007197;IPR010722;IPR002684;IPR002684	OG5_129979	Metabolism of cofactors and vitamins	Cenarchaeum Nitrosoarchaeum Nitrosopelagicus Nitrosopumilus Nitrososphaera
Candidatus Cenarchaeum massiliensisK8823_679	706	MG2 domain protein			OG5_204911	Misc	Cenarchaeum Nitrosoarchaeum Nitrosopelagicus Nitrosopumilus Nitrososphaera
Candidatus Cenarchaeum massiliensisK8823_683	197	23S rRNA methyltransferase	K02427	IPR002877;IPR015507;IPR015507	OG5_126871	Translation	Cenarchaeum Nitrosoarchaeum Nitrosopelagicus Nitrosopumilus Nitrososphaera
Candidatus Cenarchaeum massiliensisK8823_684	386	tRNA (Guanine-N(2)-)-methyltransferase	K00555	IPR002905;IPR002905;IPR002905	OG5_127349	Translation	Cenarchaeum Nitrosoarchaeum Nitrosopelagicus Nitrosopumilus Nitrososphaera
Candidatus Cenarchaeum massiliensisK8823_685	216	ribonuclease HII	K03470	IPR004649;IPR024567;IPR001352	OG5_126908	DNA modification	Cenarchaeum Nitrosoarchaeum Nitrosopelagicus Nitrosopumilus Nitrososphaera
Candidatus Cenarchaeum massiliensisK8823_59	248	SAM dependent methyltransferase			OG5_129042	Misc	Cenarchaeum Nitrosoarchaeum Nitrosopelagicus Nitrosopumilus Nitrososphaera
Candidatus Cenarchaeum massiliensisK8823_686	34	hypothetical protein			OG5_127191	RNA modification	Cenarchaeum Nitrosoarchaeum Nitrosopelagicus Nitrosopumilus Nitrososphaera
Candidatus Cenarchaeum massiliensisK8823_687	277	Fibrillarin	K04795	IPR000692	OG5_127191	RNA modification	Cenarchaeum Nitrosoarchaeum Nitrosopelagicus Nitrosopumilus Nitrososphaera
Candidatus Cenarchaeum massiliensisK8823_688	582	snoRNA binding domain protein	K14564	IPR012976;IPR002687;IPR002687	OG5_127592	Translation	Cenarchaeum Nitrosoarchaeum Nitrosopelagicus Nitrosopumilus Nitrososphaera
Candidatus Cenarchaeum massiliensisK8823_60	115	hypothetical protein			nmar|YP_001581639	Unknown function	Cenarchaeum Nitrosoarchaeum Nitrosopelagicus Nitrosopumilus Nitrososphaera
Candidatus Cenarchaeum massiliensisK8823_694	575	radical SAM protein		IPR006638;IPR007197;IPR023970	OG5_157233	Misc	Cenarchaeum Nitrosoarchaeum Nitrosopelagicus Nitrosopumilus Nitrososphaera
Candidatus Cenarchaeum massiliensisK8823_701	60	ribosomal protein S30e		IPR006846	OG5_127269	Translation	Cenarchaeum Nitrosoarchaeum Nitrosopelagicus Nitrosopumilus Nitrososphaera
Candidatus Cenarchaeum massiliensisK8823_702	194	Transcriptional regulator	K06024	IPR005234	OG5_134509	DNA modification	Cenarchaeum Nitrosoarchaeum Nitrosopelagicus Nitrosopumilus Nitrososphaera
Candidatus Cenarchaeum massiliensisK8823_703	125	ribosomal protein S8e	K02995	IPR022309;IPR001047;IPR020919	OG5_127039	Translation	Cenarchaeum Nitrosoarchaeum Nitrosopelagicus Nitrosopumilus Nitrososphaera
Candidatus Cenarchaeum massiliensisK8823_704	96	SRP19 protein		IPR002778;IPR002778	OG5_128121	Misc	Cenarchaeum Nitrosoarchaeum Nitrosopelagicus Nitrosopumilus Nitrososphaera
Candidatus Cenarchaeum massiliensisK8823_705	83	RNA binding protein Gar1		IPR007504	nmar|YP_001581852	Translation	Cenarchaeum Nitrosoarchaeum Nitrosopelagicus Nitrosopumilus Nitrososphaera
Candidatus Cenarchaeum massiliensisK8823_706	314	transcription factor IIB	K03124	IPR013763;IPR013137;IPR013150;IPR000812;IPR013137	OG5_127288	Transcription	Cenarchaeum Nitrosoarchaeum Nitrosopelagicus Nitrosopumilus Nitrososphaera
Candidatus Cenarchaeum massiliensisK8823_707	256	ABC transporter	K09013	IPR003593;IPR003439;IPR010230;IPR003439	OG5_130065	Nucleotide metabolism	Cenarchaeum Nitrosoarchaeum Nitrosopelagicus Nitrosopumilus Nitrososphaera
Candidatus Cenarchaeum massiliensisK8823_708	855	Extracellular solute binding protein	K02035	IPR000914	OG5_155059	Transport	Cenarchaeum Nitrosoarchaeum Nitrosopelagicus Nitrosopumilus Nitrososphaera
Candidatus Cenarchaeum massiliensisK8823_709	208	hypothetical protein		IPR007560	OG5_163334	DNA modification	Cenarchaeum Nitrosoarchaeum Nitrosopelagicus Nitrosopumilus Nitrososphaera
Candidatus Cenarchaeum massiliensisK8823_710	584	Membrane protein	K08979	IPR019204	OG5_146667	Unknown function	Cenarchaeum Nitrosoarchaeum Nitrosopelagicus Nitrosopumilus Nitrososphaera
Candidatus Cenarchaeum massiliensisK8823_714	107	Sjogren's syndrome/scleroderma autoantigen 1 protein		IPR009563	OG5_142704	Unknown function	Cenarchaeum Nitrosoarchaeum Nitrosopelagicus Nitrosopumilus Nitrososphaera
Candidatus Cenarchaeum massiliensisK8823_721	102	translation initiation factor 1 SUI1	K03113	IPR001950;IPR001950	OG5_133082	Translation	Cenarchaeum Nitrosoarchaeum Nitrosopelagicus Nitrosopumilus Nitrososphaera
Candidatus Cenarchaeum massiliensisK8823_64	342	tRNA-modifying enzyme	K15449	IPR013917;IPR023993;IPR007197;IPR023993	OG5_128065	Translation	Cenarchaeum Nitrosoarchaeum Nitrosopelagicus Nitrosopumilus Nitrososphaera
Candidatus Cenarchaeum massiliensisK8823_723	350	peptide ABC transporter permease	K02033	IPR000515;IPR000515	OG5_128014	Transport	Cenarchaeum Nitrosoarchaeum Nitrosopelagicus Nitrosopumilus Nitrososphaera
Candidatus Cenarchaeum massiliensisK8823_724	455	binding-protein-dependent transport system inner membrane protein	K02034	IPR000515;IPR000515	OG5_127607	Transport	Cenarchaeum Nitrosoarchaeum Nitrosopelagicus Nitrosopumilus Nitrososphaera
Candidatus Cenarchaeum massiliensisK8823_725	91	hypothetical membrane protein			nmar|YP_001582050	Unknown function	Cenarchaeum Nitrosoarchaeum Nitrosopelagicus Nitrosopumilus Nitrososphaera
Candidatus Cenarchaeum massiliensisK8823_726	132	Mov34/MPN/PAD-1 family protein		IPR028090	OG5_163328	Protein modification	Cenarchaeum Nitrosoarchaeum Nitrosopelagicus Nitrosopumilus Nitrososphaera
Candidatus Cenarchaeum massiliensisK8823_727	373	trypsin	K04771	IPR001478	OG5_126815	Protein modification	Cenarchaeum Nitrosoarchaeum Nitrosopelagicus Nitrosopumilus Nitrososphaera
Candidatus Cenarchaeum massiliensisK8823_728	384	7,8-didemethyl-8-hydroxy-5-deazariboflavin synthase subunit CofG	K11780	IPR006638;IPR007197;IPR019939;IPR019939	OG5_130963	Metabolism of cofactors and vitamins	Cenarchaeum Nitrosoarchaeum Nitrosopelagicus Nitrosopumilus Nitrososphaera
Candidatus Cenarchaeum massiliensisK8823_729	415	7,8-didemethyl-8-hydroxy-5-deazariboflavin synthase subunit CofH	K11781	IPR006638;IPR019940;IPR020050;IPR007197;IPR019940	OG5_130963	Metabolism of cofactors and vitamins	Cenarchaeum Nitrosoarchaeum Nitrosopelagicus Nitrosopumilus Nitrososphaera
Candidatus Cenarchaeum massiliensisK8823_730	304	sodium:calcium antiporter	K07301	IPR004837;IPR004481;IPR004481	OG5_132091	Transport	Cenarchaeum Nitrosoarchaeum Nitrosopelagicus Nitrosopumilus Nitrososphaera
Candidatus Cenarchaeum massiliensisK8823_731	433	hydroxymethylpyrimidine/phosphomethylpyrimidine kinase	K00941	IPR019293;IPR004399;IPR013749	OG5_128612	Metabolism of cofactors and vitamins	Cenarchaeum Nitrosoarchaeum Nitrosopelagicus Nitrosopumilus Nitrososphaera
Candidatus Cenarchaeum massiliensisK8823_65	184	non-canonical purine NTP pyrophosphatase	K02428	IPR002637;IPR002637;IPR002637	OG5_126970	Nucleotide metabolism	Cenarchaeum Nitrosoarchaeum Nitrosopelagicus Nitrosopumilus Nitrososphaera
Candidatus Cenarchaeum massiliensisK8823_732	451	thiamine biosynthesis protein ThiC	K03147	IPR002817;IPR002817;IPR002817	OG5_130886	Metabolism of cofactors and vitamins	Cenarchaeum Nitrosoarchaeum Nitrosopelagicus Nitrosopumilus Nitrososphaera
Candidatus Cenarchaeum massiliensisK8823_733	287	Prephenate dehydrogenase	K14187	IPR003099;IPR003099	OG5_133323	Amino acid metabolism	Cenarchaeum Nitrosoarchaeum Nitrosopelagicus Nitrosopumilus Nitrososphaera
Candidatus Cenarchaeum massiliensisK8823_734	451	Aspartate aminotransferase	K00812	IPR002701;IPR020822;IPR004839;IPR004838;IPR002701	OG5_126697	Amino acid metabolism	Cenarchaeum Nitrosoarchaeum Nitrosopelagicus Nitrosopumilus Nitrososphaera
Candidatus Cenarchaeum massiliensisK8823_735	370	chorismate synthase	K01736	IPR000453;IPR000453;IPR000453;IPR020541;IPR000453	OG5_128304	Amino acid metabolism	Cenarchaeum Nitrosoarchaeum Nitrosopelagicus Nitrosopumilus Nitrososphaera
Candidatus Cenarchaeum massiliensisK8823_736	248	proliferating cell nuclear antigen (pcna)	K04802	IPR022648;IPR000730;IPR022649;IPR000730;IPR000730	OG5_127352	DNA modification	Cenarchaeum Nitrosoarchaeum Nitrosopelagicus Nitrosopumilus Nitrososphaera
Candidatus Cenarchaeum massiliensisK8823_737	418	3-phosphoshikimate 1-carboxyvinyltransferase	K00800	IPR006264;IPR001986;IPR023193;IPR006264	OG5_127183	Amino acid metabolism	Cenarchaeum Nitrosoarchaeum Nitrosopelagicus Nitrosopumilus Nitrososphaera
Candidatus Cenarchaeum massiliensisK8823_738	281	shikimate kinase	K00891	IPR010189;IPR006204;IPR010189	OG5_142687	Amino acid metabolism	Cenarchaeum Nitrosoarchaeum Nitrosopelagicus Nitrosopumilus Nitrososphaera
Candidatus Cenarchaeum massiliensisK8823_739	268	shikimate dehydrogenase	K00014	IPR013708;IPR006151;IPR011342;IPR022893	OG5_128849	Amino acid metabolism	Cenarchaeum Nitrosoarchaeum Nitrosopelagicus Nitrosopumilus Nitrososphaera
Candidatus Cenarchaeum massiliensisK8823_740	221	3-dehydroquinate dehydratase	K03785	IPR001381;IPR001381;IPR001381	OG5_135712	Amino acid metabolism	Cenarchaeum Nitrosoarchaeum Nitrosopelagicus Nitrosopumilus Nitrososphaera
Candidatus Cenarchaeum massiliensisK8823_741	347	3-dehydroquinate synthase	K11646	IPR002812;IPR002812	OG5_136493	Amino acid metabolism	Cenarchaeum Nitrosoarchaeum Nitrosopelagicus Nitrosopumilus Nitrososphaera
Candidatus Cenarchaeum massiliensisK8823_66	207	Mn2+-dependent serine/threonine protein kinase	K08851	IPR022495;IPR022495;IPR008266;IPR000719	OG5_127673	Signaling	Cenarchaeum Nitrosoarchaeum Nitrosopelagicus Nitrosopumilus Nitrososphaera
Candidatus Cenarchaeum massiliensisK8823_742	238	fructose-bisphosphate aldolase	K11645	IPR002915	OG5_135382	Energy	Cenarchaeum Nitrosoarchaeum Nitrosopelagicus Nitrosopumilus Nitrososphaera
Candidatus Cenarchaeum massiliensisK8823_743	390	radical SAM protein	K11784	IPR020050;IPR007197;IPR022431	OG5_130963	Metabolism of cofactors and vitamins	Cenarchaeum Nitrosoarchaeum Nitrosopelagicus Nitrosopumilus Nitrososphaera
Candidatus Cenarchaeum massiliensisK8823_744	477	menaquinone biosynthesis decarboxylase	K03182	IPR002830;IPR022390;IPR002830	OG5_130277	Metabolism of cofactors and vitamins	Cenarchaeum Nitrosoarchaeum Nitrosopelagicus Nitrosopumilus Nitrososphaera
Candidatus Cenarchaeum massiliensisK8823_746	124	hypothetical protein			nmar|YP_001581891	Unknown function	Cenarchaeum Nitrosoarchaeum Nitrosopelagicus Nitrosopumilus Nitrososphaera
Candidatus Cenarchaeum massiliensisK8823_748	122	peroxiredoxin	K03564	IPR000866;IPR012336	OG5_127887	Oxidation-reduction	Cenarchaeum Nitrosoarchaeum Nitrosopelagicus Nitrosopumilus Nitrososphaera
Candidatus Cenarchaeum massiliensisK8823_750	236	MIP family channel protein	K06188	IPR000425;IPR000425	OG5_126615	Transport	Cenarchaeum Nitrosoarchaeum Nitrosopelagicus Nitrosopumilus Nitrososphaera
Candidatus Cenarchaeum massiliensisK8823_67	276	type 11 methyltransferase		IPR013216	OG5_132295	Misc	Cenarchaeum Nitrosoarchaeum Nitrosopelagicus Nitrosopumilus Nitrososphaera
Candidatus Cenarchaeum massiliensisK8823_68	353	aminopeptidase M24	K01262	IPR000994;IPR000587;IPR001131	OG5_126673	Protein modification	Cenarchaeum Nitrosoarchaeum Nitrosopelagicus Nitrosopumilus Nitrososphaera
Candidatus Cenarchaeum massiliensisK8823_780	319	deoxyhypusine synthase	K00809	IPR002773;IPR002773	OG5_127351	Misc	Cenarchaeum Nitrosoarchaeum Nitrosopelagicus Nitrosopumilus Nitrososphaera
Candidatus Cenarchaeum massiliensisK8823_799	185	lysyl-tRNA synthetase class I			OG5_137386	Translation	Cenarchaeum Nitrosoarchaeum Nitrosopelagicus Nitrosopumilus Nitrososphaera
Candidatus Cenarchaeum massiliensisK8823_800	331	lysine--tRNA ligase	K04566	IPR002904	OG5_137386	Translation	Cenarchaeum Nitrosoarchaeum Nitrosopelagicus Nitrosopumilus Nitrososphaera
Candidatus Cenarchaeum massiliensisK8823_801	526	tRNA uridine(34) 5-carboxymethylaminomethyl modification radical SAM/GNAT enzyme Elp3	K07739	IPR006638;IPR007197;IPR005910	OG5_127324	Translation	Cenarchaeum Nitrosoarchaeum Nitrosopelagicus Nitrosopumilus Nitrososphaera
Candidatus Cenarchaeum massiliensisK8823_803	418	potassium transporter Kef		IPR006153	OG5_128196	Transport	Cenarchaeum Nitrosoarchaeum Nitrosopelagicus Nitrosopumilus Nitrososphaera
Candidatus Cenarchaeum massiliensisK8823_804	66	phosphoribosylaminoimidazole carboxylase		IPR010918	OG5_126894	Nucleotide metabolism	Cenarchaeum Nitrosoarchaeum Nitrosopelagicus Nitrosopumilus Nitrososphaera
Candidatus Cenarchaeum massiliensisK8823_805	265	phosphoribosylformylglycinamidine cyclo-ligase	K01933	IPR004733;IPR016188;IPR010918	OG5_126894	Nucleotide metabolism	Cenarchaeum Nitrosoarchaeum Nitrosopelagicus Nitrosopumilus Nitrososphaera

Candidatus Cenarchaeum massiliensisK8823_806	241	PAC2 domain containing protein	K06869	IPR019151	OG5_140204	Amino acid metabolism	Cenarchaeum Nitrosoarchaeum Nitrosopelagicus Nitrosopumilus Nitrososphaera
Candidatus Cenarchaeum massiliensisK8823_812	373	DNA repair exonuclease	K03547	IPR004843	OG5_131553	DNA modification	Cenarchaeum Nitrosoarchaeum Nitrosopelagicus Nitrosopumilus Nitrososphaera
Candidatus Cenarchaeum massiliensisK8823_813	233	nucleoside-diphosphate-sugar pyrophosphorylase	K00966	IPR005835	OG5_127109	Carbohydrate metabolism	Cenarchaeum Nitrosoarchaeum Nitrosopelagicus Nitrosopumilus Nitrososphaera
Candidatus Cenarchaeum massiliensisK8823_814	143	acetyltransferase GNAT family	K00621	IPR000182;IPR000182	OG5_128161	Carbonydrate metabolism	Cenarchaeum Nitrosoarchaeum Nitrosopelagicus Nitrosopumilus Nitrososphaera
Candidatus Cenarchaeum massiliensisK8823_815	146	hypothetical protein			nmar|YP_001581873	Unknown function	Cenarchaeum Nitrosoarchaeum Nitrosopelagicus Nitrosopumilus Nitrososphaera
Candidatus Cenarchaeum massiliensisK8823_816	325	galactose-1-phosphate uridylyltransferase	K00965	IPR005849;IPR001937	OG5_129227	Amino sugar and nucleotide sugar metabolism	Cenarchaeum Nitrosoarchaeum Nitrosopelagicus Nitrosopumilus Nitrososphaera
Candidatus Cenarchaeum massiliensisK8823_817	427	phosphoglycerate mutase	K15635	IPR004456;IPR004456;IPR006124	OG5_131753	Energy	Cenarchaeum Nitrosoarchaeum Nitrosopelagicus Nitrosopumilus Nitrososphaera
Candidatus Cenarchaeum massiliensisK8823_818	185	hypothetical protein		IPR005500	OG5_172360	Unknown function	Cenarchaeum Nitrosoarchaeum Nitrosopelagicus Nitrosopumilus Nitrososphaera
Candidatus Cenarchaeum massiliensisK8823_819	178	hypothetical protein			nmar|YP_001581869	Translation	Cenarchaeum Nitrosoarchaeum Nitrosopelagicus Nitrosopumilus Nitrososphaera
Candidatus Cenarchaeum massiliensisK8823_820	56	ribosomal protein S27a	K02977	IPR002906;IPR022845	OG5_127221	Translation	Cenarchaeum Nitrosoarchaeum Nitrosopelagicus Nitrosopumilus Nitrososphaera
Candidatus Cenarchaeum massiliensisK8823_71	103	cell division protein SepF		IPR007561	OG5_196285	Cell & development	Cenarchaeum Nitrosoarchaeum Nitrosopelagicus Nitrosopumilus Nitrososphaera
Candidatus Cenarchaeum massiliensisK8823_821	235	hypothetical protein			nmar|YP_001581867	Unknown function	Cenarchaeum Nitrosoarchaeum Nitrosopelagicus Nitrosopumilus Nitrososphaera
Candidatus Cenarchaeum massiliensisK8823_822	382	pseudouridine synthase	K07583		OG5_128751	Translation	Cenarchaeum Nitrosoarchaeum Nitrosopelagicus Nitrosopumilus Nitrososphaera
Candidatus Cenarchaeum massiliensisK8823_823	440	signal recognition particle	K03106	IPR000897;IPR003593;IPR013822;IPR004125;IPR013822;IPR000897;IPR022941	OG5_126863	Misc	Cenarchaeum Nitrosoarchaeum Nitrosopelagicus Nitrosopumilus Nitrososphaera
Candidatus Cenarchaeum massiliensisK8823_824	237	ATP-binding protein	K06927	IPR002761;IPR022427;IPR002761;IPR030662	OG5_127536	Misc	Cenarchaeum Nitrosoarchaeum Nitrosopelagicus Nitrosopumilus Nitrososphaera
Candidatus Cenarchaeum massiliensisK8823_825	136	translation initiation factor IF-5A	K03263	IPR020189;IPR001884;IPR001884;IPR019769;IPR022847	OG5_126922	Translation	Cenarchaeum Nitrosoarchaeum Nitrosopelagicus Nitrosopumilus Nitrososphaera
Candidatus Cenarchaeum massiliensisK8823_826	449	glutamate-1-semialdehyde-2,1-aminomutase, glutamate-1-semialdehyde 2,1-aminomutase	K01845	IPR005814;IPR005814;IPR004639	OG5_129427	Metabolism of cofactors and vitamins	Cenarchaeum Nitrosoarchaeum Nitrosopelagicus Nitrosopumilus Nitrososphaera
Candidatus Cenarchaeum massiliensisK8823_827	311	hydroxymethylbilane synthase	K01749	IPR000860;IPR022417;IPR022418;IPR000860;IPR000860	OG5_127395	Metabolism of cofactors and vitamins	Cenarchaeum Nitrosoarchaeum Nitrosopelagicus Nitrosopumilus Nitrososphaera
Candidatus Cenarchaeum massiliensisK8823_829	270	Uroporphyrinogen-III synthase HemD	K13542	IPR003754	OG5_132804	Metabolism of cofactors and vitamins	Cenarchaeum Nitrosoarchaeum Nitrosopelagicus Nitrosopumilus Nitrososphaera
Candidatus Cenarchaeum massiliensisK8823_72	645	MBL fold metallo-hydrolase	K07041	IPR022712;IPR001279;IPR011108;IPR019975;IPR022712	OG5_127285	Misc	Cenarchaeum Nitrosoarchaeum Nitrosopelagicus Nitrosopumilus Nitrososphaera
Candidatus Cenarchaeum massiliensisK8823_830	466	component of SufBCD complex	K09014	IPR000825;IPR010231	OG5_129985	Misc	Cenarchaeum Nitrosoarchaeum Nitrosopelagicus Nitrosopumilus Nitrososphaera
Candidatus Cenarchaeum massiliensisK8823_831	462	Fe-S cluster assembly protein SufD	K09014	IPR000825	OG5_132298	Misc	Cenarchaeum Nitrosoarchaeum Nitrosopelagicus Nitrosopumilus Nitrososphaera
Candidatus Cenarchaeum massiliensisK8823_832	104	Naphthalene 12-dioxygenase system ferredoxin subunit protein	K05710	IPR017941;IPR017941	OG5_133960	Oxidation-reduction	Cenarchaeum Nitrosoarchaeum Nitrosopelagicus Nitrosopumilus Nitrososphaera
Candidatus Cenarchaeum massiliensisK8823_833	415	cysteine desulfurase	K11717	IPR010970;IPR000192;IPR020578	OG5_128800	Amino acid metabolism	Cenarchaeum Nitrosoarchaeum Nitrosopelagicus Nitrosopumilus Nitrososphaera
Candidatus Cenarchaeum massiliensisK8823_834	145	iron-sulfur cluster scaffold-like protein NifU family	K04488	IPR002871	OG5_127020	Misc	Cenarchaeum Nitrosoarchaeum Nitrosopelagicus Nitrosopumilus Nitrososphaera
Candidatus Cenarchaeum massiliensisK8823_835	73	hypothetical protein			nmar|YP_001581833	Unknown function	Cenarchaeum Nitrosoarchaeum Nitrosopelagicus Nitrosopumilus Nitrososphaera
Candidatus Cenarchaeum massiliensisK8823_836	398	phosphoglycerate kinase	K00927	IPR001576;IPR001576;IPR015911	OG5_126776	Energy	Cenarchaeum Nitrosoarchaeum Nitrosopelagicus Nitrosopumilus Nitrososphaera
Candidatus Cenarchaeum massiliensisK8823_837	341	ATP-utilizing protein	K06864	IPR018317;IPR005232	OG5_137379	Amino acid metabolism	Cenarchaeum Nitrosoarchaeum Nitrosopelagicus Nitrosopumilus Nitrososphaera
Candidatus Cenarchaeum massiliensisK8823_838	406	hypothetical protein	K09121	IPR002822;IPR002822	OG5_137915	Unknown function	Cenarchaeum Nitrosoarchaeum Nitrosopelagicus Nitrosopumilus Nitrososphaera
Candidatus Cenarchaeum massiliensisK8823_839	107	hypothetical protein			nmar|YP_001581690	Unknown function	Cenarchaeum Nitrosoarchaeum Nitrosopelagicus Nitrosopumilus Nitrososphaera
Candidatus Cenarchaeum massiliensisK8823_73	242	proteasome endopeptidase complex protein, proteasome beta subunit	K03433	IPR001353;IPR016050;IPR019983;IPR023333	OG5_127100	Protein modification	Cenarchaeum Nitrosoarchaeum Nitrosopelagicus Nitrosopumilus Nitrososphaera
Candidatus Cenarchaeum massiliensisK8823_840	238	hypothetical protein		IPR005358	nmar|YP_001581691	Unknown function	Cenarchaeum Nitrosoarchaeum Nitrosopelagicus Nitrosopumilus Nitrososphaera
Candidatus Cenarchaeum massiliensisK8823_841	335	dehydrogenase			OG5_163274	Unknown function	Cenarchaeum Nitrosoarchaeum Nitrosopelagicus Nitrosopumilus Nitrososphaera
Candidatus Cenarchaeum massiliensisK8823_842	944	leucine--tRNA ligase	K01869	IPR004493;IPR013155;IPR002300;IPR020791	OG5_127407	Translation	Cenarchaeum Nitrosoarchaeum Nitrosopelagicus Nitrosopumilus Nitrososphaera
Candidatus Cenarchaeum massiliensisK8823_844	122	twitching motility protein PilT		IPR006984	OG5_127679	Unknown function	Cenarchaeum Nitrosoarchaeum Nitrosopelagicus Nitrosopumilus Nitrososphaera
Candidatus Cenarchaeum massiliensisK8823_845	420	translation initiation factor IF-2 subunit gamma	K03242	IPR015256;IPR022424;IPR005225;IPR000795	OG5_127447	Translation	Cenarchaeum Nitrosoarchaeum Nitrosopelagicus Nitrosopumilus Nitrososphaera
Candidatus Cenarchaeum massiliensisK8823_846	142	ribosomal protein S6e	K02991	IPR001377;IPR001377;IPR018282;IPR020924	OG5_126964	Translation	Cenarchaeum Nitrosoarchaeum Nitrosopelagicus Nitrosopumilus Nitrososphaera
Candidatus Cenarchaeum massiliensisK8823_847	57	hypothetical protein			nmar|YP_001581408	Unknown function	Cenarchaeum Nitrosoarchaeum Nitrosopelagicus Nitrosopumilus Nitrososphaera
Candidatus Cenarchaeum massiliensisK8823_848	324	TPR repeat protein		IPR019734;IPR019734;IPR019734;IPR013026	OG5_130176	Unknown function	Cenarchaeum Nitrosoarchaeum Nitrosopelagicus Nitrosopumilus Nitrososphaera
Candidatus Cenarchaeum massiliensisK8823_849	176	glutamine amidotransferase class I (guaA)	K01951	IPR017926;IPR017926	OG5_130967	Amino acid metabolism	Cenarchaeum Nitrosoarchaeum Nitrosopelagicus Nitrosopumilus Nitrososphaera
Candidatus Cenarchaeum massiliensisK8823_74	330	glycerol-1-phosphate dehydrogenase	K00096	IPR023002	OG5_132294	Lipid metabolism	Cenarchaeum Nitrosoarchaeum Nitrosopelagicus Nitrosopumilus Nitrososphaera
Candidatus Cenarchaeum massiliensisK8823_850	946	hypothetical membrane protein	K09118	IPR005372	OG5_152620	Unknown function	Cenarchaeum Nitrosoarchaeum Nitrosopelagicus Nitrosopumilus Nitrososphaera
Candidatus Cenarchaeum massiliensisK8823_851	377	DNA primase small subunit	K02683	IPR002755;IPR023639	OG5_140147	DNA modification	Cenarchaeum Nitrosoarchaeum Nitrosopelagicus Nitrosopumilus Nitrososphaera
Candidatus Cenarchaeum massiliensisK8823_852	337	DNA primase large subunit	K18882	IPR007238;IPR023642	OG5_144462	DNA modification	Cenarchaeum Nitrosoarchaeum Nitrosopelagicus Nitrosopumilus Nitrososphaera
Candidatus Cenarchaeum massiliensisK8823_853	118	NAC domain-containing protein	K03626	IPR002715;IPR005231;IPR002715	OG5_141311	Transport	Cenarchaeum Nitrosoarchaeum Nitrosopelagicus Nitrosopumilus Nitrososphaera
Candidatus Cenarchaeum massiliensisK8823_854	151	PUA domain protein	K07398	IPR002478;IPR002478;IPR004521;IPR029402;IPR002478	OG5_149696	RNA modification	Cenarchaeum Nitrosoarchaeum Nitrosopelagicus Nitrosopumilus Nitrososphaera
Candidatus Cenarchaeum massiliensisK8823_855	154	aspartate carbamoyltransferase regulatory subunit	K00610	IPR020545;IPR002801;IPR020542;IPR020545;IPR002801	OG5_136082	Nucleotide metabolism	Cenarchaeum Nitrosoarchaeum Nitrosopelagicus Nitrosopumilus Nitrososphaera
Candidatus Cenarchaeum massiliensisK8823_856	205	translin family protein	K07477	IPR002848	OG5_137268	Misc	Cenarchaeum Nitrosoarchaeum Nitrosopelagicus Nitrosopumilus Nitrososphaera
Candidatus Cenarchaeum massiliensisK8823_857	84	DNA-directed RNA polymerase subunit N	K03058	IPR000268;IPR020789;IPR000268;IPR000268	OG5_127691	Transcription	Cenarchaeum Nitrosoarchaeum Nitrosopelagicus Nitrosopumilus Nitrososphaera
Candidatus Cenarchaeum massiliensisK8823_858	410	phosphopyruvate hydratase	K01689	IPR020811;IPR020810;IPR000941;IPR000941	OG5_126698	Energy	Cenarchaeum Nitrosoarchaeum Nitrosopelagicus Nitrosopumilus Nitrososphaera
Candidatus Cenarchaeum massiliensisK8823_859	216	ribosomal protein S2	K02967	IPR005707;IPR001865;IPR005707;IPR018130;IPR023454	OG5_127014	Translation	Cenarchaeum Nitrosoarchaeum Nitrosopelagicus Nitrosopumilus Nitrososphaera
Candidatus Cenarchaeum massiliensisK8823_75	334	peptidylprolyl isomerase	K03775	IPR001179;IPR001179	OG5_130574	Protein modification	Cenarchaeum Nitrosoarchaeum Nitrosopelagicus Nitrosopumilus Nitrososphaera
Candidatus Cenarchaeum massiliensisK8823_860	314	mevalonate kinase	K00869	IPR006204;IPR013750;IPR006205;IPR006203	OG5_128331	Lipid metabolism	Cenarchaeum Nitrosoarchaeum Nitrosopelagicus Nitrosopumilus Nitrososphaera
Candidatus Cenarchaeum massiliensisK8823_862	249	Amino acid kinase	K06981	IPR001048;IPR024192	OG5_133466	Lipid metabolism	Cenarchaeum Nitrosoarchaeum Nitrosopelagicus Nitrosopumilus Nitrososphaera
Candidatus Cenarchaeum massiliensisK8823_864	329	Geranylgeranyl diphosphate synthase	K13787	IPR000092;IPR017446;IPR000092	OG5_126937	Lipid metabolism	Cenarchaeum Nitrosoarchaeum Nitrosopelagicus Nitrosopumilus Nitrososphaera
Candidatus Cenarchaeum massiliensisK8823_865	569	glutamyl-tRNA synthetase	K01885	IPR020059;IPR004526;IPR020058;IPR000924;IPR001412	OG5_128638	Amino acid metabolism	Cenarchaeum Nitrosoarchaeum Nitrosopelagicus Nitrosopumilus Nitrososphaera
Candidatus Cenarchaeum massiliensisK8823_866	291	2-keto-4-pentenoate hydratase		IPR011234	OG5_126763	Amino acid metabolism	Cenarchaeum Nitrosoarchaeum Nitrosopelagicus Nitrosopumilus Nitrososphaera
Candidatus Cenarchaeum massiliensisK8823_868	540	thermosome subunit		IPR002423;IPR012714;IPR002423;IPR002194;IPR002194	OG5_127483	Protein modification	Cenarchaeum Nitrosoarchaeum Nitrosopelagicus Nitrosopumilus Nitrososphaera
Candidatus Cenarchaeum massiliensisK8823_76	378	Aspartate aminotransferase		IPR000192;IPR020578	OG5_127282	Misc	Cenarchaeum Nitrosoarchaeum Nitrosopelagicus Nitrosopumilus Nitrososphaera
Candidatus Cenarchaeum massiliensisK8823_870	2154	DEAD-box helicase	K05592	IPR001650;IPR014001;IPR001650;IPR011545;IPR001650;IPR014014;IPR014001	OG5_126772	RNA modification	Cenarchaeum Nitrosoarchaeum Nitrosopelagicus Nitrosopumilus Nitrososphaera
Candidatus Cenarchaeum massiliensisK8823_873	198	DNA-directed RNA polymerase subunit E'	K03049	IPR022967;IPR005576;IPR004519;IPR003029;IPR003029	OG5_127589	Transcription	Cenarchaeum Nitrosoarchaeum Nitrosopelagicus Nitrosopumilus Nitrososphaera
Candidatus Cenarchaeum massiliensisK8823_874	62	DNA-directed RNA polymerase subunit E"	K03050	IPR022800;IPR007178;IPR007178	OG5_139288	Transcription	Cenarchaeum Nitrosoarchaeum Nitrosopelagicus Nitrosopumilus Nitrososphaera
Candidatus Cenarchaeum massiliensisK8823_875	272	nicotinate-nucleotide pyrophosphorylase	K00767	IPR004393;IPR002638;IPR022412	OG5_128545	Metabolism of cofactors and vitamins	Cenarchaeum Nitrosoarchaeum Nitrosopelagicus Nitrosopumilus Nitrososphaera
Candidatus Cenarchaeum massiliensisK8823_876	318	quinolinate synthetase A	K03517	IPR003473;IPR003473;IPR023066	OG5_132446	Quinolinate metabolism	Cenarchaeum Nitrosoarchaeum Nitrosopelagicus Nitrosopumilus Nitrososphaera
Candidatus Cenarchaeum massiliensisK8823_877	271	aspartate dehydrogenase	K06989	IPR022487;IPR005106;IPR002811;IPR020626	OG5_135106	Metabolism of cofactors and vitamins	Cenarchaeum Nitrosoarchaeum Nitrosopelagicus Nitrosopumilus Nitrososphaera
Candidatus Cenarchaeum massiliensisK8823_878	282	phosphoribosylaminoimidazolesuccinocarboxamide synthase	K01923	IPR001636;IPR028923;IPR018236;IPR018236;IPR028923	OG5_131443	Nucleotide metabolism	Cenarchaeum Nitrosoarchaeum Nitrosopelagicus Nitrosopumilus Nitrososphaera
Candidatus Cenarchaeum massiliensisK8823_880	432	hypothetical protein			OG5_134989	Unknown function	Cenarchaeum Nitrosoarchaeum Nitrosopelagicus Nitrosopumilus Nitrososphaera
Candidatus Cenarchaeum massiliensisK8823_881	219	copper binding protein		IPR000923	OG5_144955	Misc	Cenarchaeum Nitrosoarchaeum Nitrosopelagicus Nitrosopumilus Nitrososphaera
Candidatus Cenarchaeum massiliensisK8823_882	537	excinuclease ABC subunit C	K03703	IPR000305;IPR001162;IPR001943;IPR004791;IPR000305;IPR000305;IPR001943;IPR001162	OG5_131519	DNA modification	Cenarchaeum Nitrosoarchaeum Nitrosopelagicus Nitrosopumilus Nitrososphaera
Candidatus Cenarchaeum massiliensisK8823_883	942	Excinuclease ABC subunit A	K03701	IPR003439;IPR004602;IPR017871;IPR017871;IPR003439	OG5_130794	Transport	Cenarchaeum Nitrosoarchaeum Nitrosopelagicus Nitrosopumilus Nitrososphaera
Candidatus Cenarchaeum massiliensisK8823_884	652	excinuclease ABC subunit B	K03702	IPR001650;IPR014001;IPR001943;IPR004807;IPR006935;IPR001650;IPR024759;IPR004807;IPR014001;IPR001943;IPR001[illegible]	OG5_131146	DNA modification	Cenarchaeum Nitrosoarchaeum Nitrosopelagicus Nitrosopumilus Nitrososphaera
Candidatus Cenarchaeum massiliensisK8823_885	555	DNA Helicase	K03722	IPR006554;IPR006555;IPR006555;IPR006935;IPR001650;IPR014013	OG5_178656	DNA modification	Cenarchaeum Nitrosoarchaeum Nitrosopelagicus Nitrosopumilus Nitrososphaera
Candidatus Cenarchaeum massiliensisK8823_509	180	hypothetical protein		IPR014923	OG5_161533	Unknown function	Cenarchaeum Nitrosoarchaeum Nitrosopelagicus Nitrososphaera
Candidatus Cenarchaeum massiliensisK8823_921	128	hypothetical protein			nmar|YP_001582319	Unknown function	Cenarchaeum Nitrosoarchaeum Nitrosopumilus
Candidatus Cenarchaeum massiliensisK8823_89	157	hypothetical protein			nmar|YP_001583078	Unknown function	Cenarchaeum Nitrosoarchaeum Nitrosopumilus
Candidatus Cenarchaeum massiliensisK8823_1011	102	hypothetical protein			nmar|YP_001582785	Unknown function	Cenarchaeum Nitrosoarchaeum Nitrosopumilus
Candidatus Cenarchaeum massiliensisK8823_1229	61	hypothetical protein			STG_0096	Unknown function	Cenarchaeum Nitrosoarchaeum Nitrosopumilus
Candidatus Cenarchaeum massiliensisK8823_1400	214	hypothetical membrane protein			nmar|YP_001582856	Unknown function	Cenarchaeum Nitrosoarchaeum Nitrosopumilus
Candidatus Cenarchaeum massiliensisK8823_1433	55	hypothetical protein			STG_0242	Unknown function	Cenarchaeum Nitrosoarchaeum Nitrosopumilus
Candidatus Cenarchaeum massiliensisK8823_1478	118	hypothetical membrane protein			nmar|YP_001582529	Unknown function	Cenarchaeum Nitrosoarchaeum Nitrosopumilus
Candidatus Cenarchaeum massiliensisK8823_141	52	hypothetical protein		IPR007159;IPR007159;IPR007159	nmar|YP_001583132	Transcription	Cenarchaeum Nitrosoarchaeum Nitrosopumilus
Candidatus Cenarchaeum massiliensisK8823_238	100	hypothetical protein			nmar|YP_001581356	Unknown function	Cenarchaeum Nitrosoarchaeum Nitrosopumilus
Candidatus Cenarchaeum massiliensisK8823_453	148	hypothetical protein			nmar|YP_001582829	Unknown function	Cenarchaeum Nitrosoarchaeum Nitrosopumilus
Candidatus Cenarchaeum massiliensisK8823_507	113	hypothetical protein			nmar|YP_001581847	Unknown function	Cenarchaeum Nitrosoarchaeum Nitrosopumilus
Candidatus Cenarchaeum massiliensisK8823_511	985	hypothetical protein			nmar|YP_001581964	Unknown function	Cenarchaeum Nitrosoarchaeum Nitrosopumilus
Candidatus Cenarchaeum massiliensisK8823_528	150	hypothetical protein			nmar|YP_001581808	Unknown function	Cenarchaeum Nitrosoarchaeum Nitrosopumilus
Candidatus Cenarchaeum massiliensisK8823_626	93	hypothetical membrane protein			nmar|YP_001582304	Unknown function	Cenarchaeum Nitrosoarchaeum Nitrosopumilus
Candidatus Cenarchaeum massiliensisK8823_661	114	hypothetical protein			nmar|YP_001582423	Unknown function	Cenarchaeum Nitrosoarchaeum Nitrosopumilus
Candidatus Cenarchaeum massiliensisK8823_943	381	aminotransferase class V	K04487	IPR000192;IPR020578	OG5_126959	Metabolism of cofactors and vitamins	Cenarchaeum Nitrosoarchaeum Nitrosopumilus Nitrososphaera
Candidatus Cenarchaeum massiliensisK8823_974	103	hypothetical protein			STG_0022	Unknown function	Cenarchaeum Nitrosoarchaeum Nitrosopumilus Nitrososphaera
Candidatus Cenarchaeum massiliensisK8823_92	217	Phosphate uptake regulator		IPR026022	OG5_131162	Transport	Cenarchaeum Nitrosoarchaeum Nitrosopumilus Nitrososphaera
Candidatus Cenarchaeum massiliensisK8823_93	272	ABC phosphate uptake transporter, ATP-binding protein	K02036	IPR003593;IPR003439;IPR005670;IPR017871;IPR003439;IPR015850	OG5_130152	Transport	Cenarchaeum Nitrosoarchaeum Nitrosopumilus Nitrososphaera
Candidatus Cenarchaeum massiliensisK8823_94	260	phosphate ABC transporter, permease protein PstA	K02038	IPR000515;IPR005672;IPR000515	OG5_131047	Transport	Cenarchaeum Nitrosoarchaeum Nitrosopumilus Nitrososphaera
Candidatus Cenarchaeum massiliensisK8823_95	325	phosphate ABC transporter permease subunit PstC	K02037	IPR011864;IPR000515;IPR000515	OG5_131165	Transport	Cenarchaeum Nitrosoarchaeum Nitrosopumilus Nitrososphaera
Candidatus Cenarchaeum massiliensisK8823_96	411	phosphate ABC transporter, periplasmic phosphate-binding protein	K02040	IPR024370;IPR005673	OG5_132742	Transport	Cenarchaeum Nitrosoarchaeum Nitrosopumilus Nitrososphaera
Candidatus Cenarchaeum massiliensisK8823_1047	821	methionine synthase	K00548	IPR003759;IPR000489;IPR006158;IPR004223;IPR003759;IPR006158;IPR003759;IPR004223;IPR000489	OG5_129374	Amino acid metabolism	Cenarchaeum Nitrosoarchaeum Nitrosopumilus Nitrososphaera
Candidatus Cenarchaeum massiliensisK8823_1048	323	homocysteine S-methyltransferase	K00548	IPR003726;IPR003726	OG5_129374	Amino acid metabolism	Cenarchaeum Nitrosoarchaeum Nitrosopumilus Nitrososphaera
Candidatus Cenarchaeum massiliensisK8823_99	519	hypothetical protein			OG5_241572	Unknown function	Cenarchaeum Nitrosoarchaeum Nitrosopumilus Nitrososphaera

Candidatus Cenarchaeum massiliensisK8823_1073	382	Redoxin domain-containing protein		IPR000866;IPR012336	OG5_208929	Misc	Cenarchaeum Nitrosoarchaeum Nitrosopumilus Nitrososphaera
Candidatus Cenarchaeum massiliensisK8823_1140	257	indole-3-glycerol-phosphate synthase	K01609	IPR013798	OG5_129980	Amino acid metabolism	Cenarchaeum Nitrosoarchaeum Nitrosopumilus Nitrososphaera
Candidatus Cenarchaeum massiliensisK8823_12	405	Cysteine sulfinate desulfinase/cysteine desulfurase	K04487	IPR000192;IPR020578	OG5_126959	Metabolism of cofactors and vitamins	Cenarchaeum Nitrosoarchaeum Nitrosopumilus Nitrososphaera
Candidatus Cenarchaeum massiliensisK8823_1460	335	pyridoxal-phosphate-dependent protein	K01738	IPR001926	OG5_126662	Amino acid metabolism	Cenarchaeum Nitrosoarchaeum Nitrosopumilus Nitrososphaera
Candidatus Cenarchaeum massiliensisK8823_1471	76	hypothetical protein			nmar|YP_001582526	Unknown function	Cenarchaeum Nitrosoarchaeum Nitrosopumilus Nitrososphaera
Candidatus Cenarchaeum massiliensisK8823_1475	151	hypothetical protein			nmar|YP_001582521	Unknown function	Cenarchaeum Nitrosoarchaeum Nitrosopumilus Nitrososphaera
Candidatus Cenarchaeum massiliensisK8823_1598	382	aminotransferase class V	K04487	IPR000192;IPR020578	OG5_126959	Metabolism of cofactors and vitamins	Cenarchaeum Nitrosoarchaeum Nitrosopumilus Nitrososphaera
Candidatus Cenarchaeum massiliensisK8823_164	565	hypothetical membrane protein	K07333		OG5_138561	Misc	Cenarchaeum Nitrosoarchaeum Nitrosopumilus Nitrososphaera
Candidatus Cenarchaeum massiliensisK8823_1658	272	6-pyruvoyltetrahydropterin synthase		IPR007115	OG5_141276	Misc	Cenarchaeum Nitrosoarchaeum Nitrosopumilus Nitrososphaera
Candidatus Cenarchaeum massiliensisK8823_1663	384	cysteine desulfurase	K04487	IPR000192;IPR020578	OG5_126959	Metabolism of cofactors and vitamins	Cenarchaeum Nitrosoarchaeum Nitrosopumilus Nitrososphaera
Candidatus Cenarchaeum massiliensisK8823_1670	384	cysteine desulfurase	K04487	IPR000192;IPR020578	OG5_126959	Metabolism of cofactors and vitamins	Cenarchaeum Nitrosoarchaeum Nitrosopumilus Nitrososphaera
Candidatus Cenarchaeum massiliensisK8823_242	131	hypothetical protein	K09736	IPR002739	OG5_145374	Unknown function	Cenarchaeum Nitrosoarchaeum Nitrosopumilus Nitrososphaera
Candidatus Cenarchaeum massiliensisK8823_267	695	regulatory protein ArsR	K00527	IPR001845;IPR005144	OG5_138803	Nucleotide metabolism	Cenarchaeum Nitrosoarchaeum Nitrosopumilus Nitrososphaera
Candidatus Cenarchaeum massiliensisK8823_349	79	hypothetical protein			nmar|YP_001582401	Unknown function	Cenarchaeum Nitrosoarchaeum Nitrosopumilus Nitrososphaera
Candidatus Cenarchaeum massiliensisK8823_380	1019	DEAD/DEAH box helicase	K01153	IPR014001;IPR021810;IPR006935;IPR004473;IPR007409;IPR001650;IPR014001	OG5_135105	Misc	Cenarchaeum Nitrosoarchaeum Nitrosopumilus Nitrososphaera
Candidatus Cenarchaeum massiliensisK8823_381	405	restriction endonuclease S subunit		IPR000055	OG5_132451	DNA modification	Cenarchaeum Nitrosoarchaeum Nitrosopumilus Nitrososphaera
Candidatus Cenarchaeum massiliensisK8823_382	529	N-6 DNA methylase	K03427	IPR022749;IPR003356	OG5_135747	DNA modification	Cenarchaeum Nitrosoarchaeum Nitrosopumilus Nitrososphaera
Candidatus Cenarchaeum massiliensisK8823_540	840	helicase	K06877	IPR001650;IPR014001;IPR001650;IPR011545;IPR018973;IPR014001;IPR001650	OG5_130740	DNA modification	Cenarchaeum Nitrosoarchaeum Nitrosopumilus Nitrososphaera
Candidatus Cenarchaeum massiliensisK8823_549	225	class I glutamine amidotransferase		IPR017926;IPR017926	OG5_136506	Amino acid metabolism	Cenarchaeum Nitrosoarchaeum Nitrosopumilus Nitrososphaera
Candidatus Cenarchaeum massiliensisK8823_559	418	hypothetical protein			OG5_241572	Unknown function	Cenarchaeum Nitrosoarchaeum Nitrosopumilus Nitrososphaera
Candidatus Cenarchaeum massiliensisK8823_576	330	hypothetical protein			OG5_182763	Carbohydrate metabolism	Cenarchaeum Nitrosoarchaeum Nitrosopumilus Nitrososphaera
Candidatus Cenarchaeum massiliensisK8823_581	606	potassium transporter Trk		IPR000644;IPR003445;IPR000644;IPR000644;IPR000644	OG5_133741	Transport	Cenarchaeum Nitrosoarchaeum Nitrosopumilus Nitrososphaera
Candidatus Cenarchaeum massiliensisK8823_641	253	Nucleotidyl transferase	K00966	IPR005835	OG5_131193	Carbohydrate metabolism	Cenarchaeum Nitrosoarchaeum Nitrosopumilus Nitrososphaera
Candidatus Cenarchaeum massiliensisK8823_666	371	carbamoyl-phosphate synthase small subunit	K01956	IPR002474;IPR002474;IPR006274;IPR017926;IPR006274;IPR017926	OG5_128943	Nucleotide metabolism	Cenarchaeum Nitrosoarchaeum Nitrosopumilus Nitrososphaera
Candidatus Cenarchaeum massiliensisK8823_61	327	Mg2 and Co2 transporter	K03284	IPR002523	OG5_155694	Transport	Cenarchaeum Nitrosoarchaeum Nitrosopumilus Nitrososphaera
Candidatus Cenarchaeum massiliensisK8823_745	74	hypothetical protein			nmar|YP_001581547	Unknown function	Cenarchaeum Nitrosoarchaeum Nitrosopumilus Nitrososphaera
Candidatus Cenarchaeum massiliensisK8823_773	389	aminotransferase class V	K04487	IPR000192;IPR020578	OG5_126959	Metabolism of cofactors and vitamins	Cenarchaeum Nitrosoarchaeum Nitrosopumilus Nitrososphaera
Candidatus Cenarchaeum massiliensisK8823_70	334	tRNA (5-methyl aminomethyl-2-thiouridylate)-methyltransferase	K00566	IPR020536;IPR004506	OG5_158947	Translation	Cenarchaeum Nitrosoarchaeum Nitrosopumilus Nitrososphaera
Candidatus Cenarchaeum massiliensisK8823_828	246	uroporphyrin-III C-methyltransferase	K02303	IPR006366;IPR000878;IPR003043	OG5_128100	Metabolism of cofactors and vitamins	Cenarchaeum Nitrosoarchaeum Nitrosopumilus Nitrososphaera
Candidatus Cenarchaeum massiliensisK8823_1452	91	hypothetical protein			STG_0060	Unknown function	Cenarchaeum Nitrosoarchaeum Nitrososphaera
Candidatus Cenarchaeum massiliensisK8823_273	159	Integrase protein family		IPR001584	STG_0023	Unknown function	Cenarchaeum Nitrosoarchaeum Nitrososphaera
Candidatus Cenarchaeum massiliensisK8823_337	502	MBL fold metallo-hydrolase	K12574	IPR001279;IPR011108;IPR004613;IPR004613	OG5_131049	Misc	Cenarchaeum Nitrosoarchaeum Nitrososphaera
Candidatus Cenarchaeum massiliensisK8823_1333	327	hypothetical protein			STG_0268	Unknown function	Cenarchaeum Nitrosopelagicus
Candidatus Cenarchaeum massiliensisK8823_1644	84	hypothetical protein		IPR007087;IPR007087	STG_0819	Unknown function	Cenarchaeum Nitrosopelagicus
Candidatus Cenarchaeum massiliensisK8823_682	436	hypothetical protein			OG5_152595	Unknown function	Cenarchaeum Nitrosopelagicus
Candidatus Cenarchaeum massiliensisK8823_205	102	hypothetical protein			OG5_146378	Unknown function	Cenarchaeum Nitrosopelagicus Nitrosopumilus
Candidatus Cenarchaeum massiliensisK8823_422	317	aerotolerance regulator BatA	K07114	IPR002035;IPR011933;IPR024163;IPR002035	OG5_143376	Stress & defense	Cenarchaeum Nitrosopelagicus Nitrosopumilus
Candidatus Cenarchaeum massiliensisK8823_423	295	hypothetical protein		IPR002881	OG5_149731	Unknown function	Cenarchaeum Nitrosopelagicus Nitrosopumilus
Candidatus Cenarchaeum massiliensisK8823_424	330	ATPase	K03924	IPR011703	OG5_130615	Misc	Cenarchaeum Nitrosopelagicus Nitrosopumilus
Candidatus Cenarchaeum massiliensisK8823_565	414	iron transporter		IPR001046	OG5_177650	Transport	Cenarchaeum Nitrosopelagicus Nitrosopumilus
Candidatus Cenarchaeum massiliensisK8823_598	43	hypothetical protein			nmar|YP_001582507	Unknown function	Cenarchaeum Nitrosopelagicus Nitrosopumilus
Candidatus Cenarchaeum massiliensisK8823_1179	353	DNA (cytosine-5-)-methyltransferase	K00558	IPR001525;IPR018117;IPR001525	OG5_129430	Amino acid metabolism	Cenarchaeum Nitrosopelagicus Nitrosopumilus Nitrososphaera
Candidatus Cenarchaeum massiliensisK8823_1398	165	hypothetical membrane protein			OG5_234685	Unknown function	Cenarchaeum Nitrosopelagicus Nitrosopumilus Nitrososphaera
Candidatus Cenarchaeum massiliensisK8823_1619	462	DNA cytosine methyltransferase	K00558	IPR001525;IPR018117;IPR001525	OG5_129430	Amino acid metabolism	Cenarchaeum Nitrosopelagicus Nitrosopumilus Nitrososphaera
Candidatus Cenarchaeum massiliensisK8823_322	48	Ribosomal protein L37E		IPR001569;IPR001569;IPR001569	OG5_126918	Translation	Cenarchaeum Nitrosopelagicus Nitrosopumilus Nitrososphaera
Candidatus Cenarchaeum massiliensisK8823_518	324	DNA-cytosine methyltransferase	K00558	IPR001525;IPR018117;IPR001525	OG5_129430	Amino acid metabolism	Cenarchaeum Nitrosopelagicus Nitrosopumilus Nitrososphaera
Candidatus Cenarchaeum massiliensisK8823_655	332	cytosine-specific methyltransferase	K00558	IPR001525;IPR018117;IPR001525	OG5_129430	Amino acid metabolism	Cenarchaeum Nitrosopelagicus Nitrosopumilus Nitrososphaera
Candidatus Cenarchaeum massiliensisK8823_763	353	DNA (cytosine-5-)-methyltransferase	K00558	IPR001525;IPR018117;IPR001525	OG5_129430	Amino acid metabolism	Cenarchaeum Nitrosopelagicus Nitrosopumilus Nitrososphaera
Candidatus Cenarchaeum massiliensisK8823_1536	156	methyltransferase		IPR007757;IPR007757	OG5_129618	Transcription	Cenarchaeum Nitrosopumilus
Candidatus Cenarchaeum massiliensisK8823_1573	124	hypothetical protein			nmar|YP_001582496	Unknown function	Cenarchaeum Nitrosopumilus
Candidatus Cenarchaeum massiliensisK8823_1603	320	hypothetical protein			OG5_229125	Unknown function	Cenarchaeum Nitrosopumilus
Candidatus Cenarchaeum massiliensisK8823_1650	356	Phosphonate ABC transporter, periplasmic phosphonate-binding protein	K02044	IPR005770	OG5_143155	Transport	Cenarchaeum Nitrosopumilus
Candidatus Cenarchaeum massiliensisK8823_1651	250	phosphate ABC transporter ATPase	K02071	IPR003593;IPR003439;IPR017871;IPR003439	OG5_152077	Nucleotide metabolism	Cenarchaeum Nitrosopumilus
Candidatus Cenarchaeum massiliensisK8823_1652	260	phosphonate ABC transporter inner membrane subunit (phnE)	K02042	IPR000515;IPR005769;IPR000515	OG5_136945	Transport	Cenarchaeum Nitrosopumilus
Candidatus Cenarchaeum massiliensisK8823_421	624	hypothetical protein			STG_0822	Unknown function	Cenarchaeum Nitrosopumilus
Candidatus Cenarchaeum massiliensisK8823_521	77	zinc finger CDGSH-type domain protein		IPR006622;IPR018967	OG5_131688	Unknown function	Cenarchaeum Nitrosopumilus
Candidatus Cenarchaeum massiliensisK8823_602	416	ABC-type branched-chain amino acid transport system, periplasmic component	K01999	IPR028081	OG5_130525	Transport	Cenarchaeum Nitrosopumilus
Candidatus Cenarchaeum massiliensisK8823_604	550	ABC-type branched-chain amino acid transport system, periplasmic component	K01999	IPR028081	OG5_130525	Transport	Cenarchaeum Nitrosopumilus
Candidatus Cenarchaeum massiliensisK8823_605	243	ABC-type branched-chain amino acid transport system, periplasmic component	K01999		OG5_130525	Transport	Cenarchaeum Nitrosopumilus
Candidatus Cenarchaeum massiliensisK8823_627	90	zinc finger UBP-type protein		IPR001607;IPR001607;IPR001607	OG5_132969	Unknown function	Cenarchaeum Nitrosopumilus
Candidatus Cenarchaeum massiliensisK8823_783	320	hypothetical protein			OG5_229125	Unknown function	Cenarchaeum Nitrosopumilus
Candidatus Cenarchaeum massiliensisK8823_901	331	Cobyrinic acid a,c-diamide synthase	K03496	IPR002586	OG5_130366	Metabolism of cofactors and vitamins	Cenarchaeum Nitrosopumilus Nitrososphaera
Candidatus Cenarchaeum massiliensisK8823_1322	271	parB-like partition protein		IPR003115;IPR003115;IPR004437	OG5_132436	Transcription	Cenarchaeum Nitrosopumilus Nitrososphaera
Candidatus Cenarchaeum massiliensisK8823_1566	275	Gp37Gp68 family protein		IPR011101	OG5_165182	Misc	Cenarchaeum Nitrosopumilus Nitrososphaera
Candidatus Cenarchaeum massiliensisK8823_398	346	CRISPR-associated protein Cas1	K15342	IPR002729;IPR019856;IPR002729;IPR002729	OG5_136066	Stress & defense	Cenarchaeum Nitrosopumilus Nitrososphaera
Candidatus Cenarchaeum massiliensisK8823_889	85	hypothetical protein			STG_0071	Unknown function	Cenarchaeum Nitrososphaera
Candidatus Cenarchaeum massiliensisK8823_899	244	Transposase		IPR002559	OG5_136743	DNA modification	Cenarchaeum Nitrososphaera
Candidatus Cenarchaeum massiliensisK8823_902	99	Winged helix-turn-helix containing protein			OG5_127031	Unknown function	Cenarchaeum Nitrososphaera
Candidatus Cenarchaeum massiliensisK8823_903	244	Transposase		IPR002559	OG5_136743	DNA modification	Cenarchaeum Nitrososphaera
Candidatus Cenarchaeum massiliensisK8823_904	79	Transposase		IPR002559	OG5_136743	DNA modification	Cenarchaeum Nitrososphaera
Candidatus Cenarchaeum massiliensisK8823_933	334	transposase			OG5_127031	Unknown function	Cenarchaeum Nitrososphaera
Candidatus Cenarchaeum massiliensisK8823_937	100	Transposase		IPR004291	STG_0047	Unknown function	Cenarchaeum Nitrososphaera
Candidatus Cenarchaeum massiliensisK8823_939	82	Transposase			STG_0101	Unknown function	Cenarchaeum Nitrososphaera
Candidatus Cenarchaeum massiliensisK8823_940	203	Transposase			OG5_127031	Unknown function	Cenarchaeum Nitrososphaera
Candidatus Cenarchaeum massiliensisK8823_949	101	Transposase			OG5_127031	Unknown function	Cenarchaeum Nitrososphaera
Candidatus Cenarchaeum massiliensisK8823_8	116	Transposase		IPR002559	STG_0032	Unknown function	Cenarchaeum Nitrososphaera
Candidatus Cenarchaeum massiliensisK8823_88	58	hypothetical protein			STG_0110	Unknown function	Cenarchaeum Nitrososphaera
Candidatus Cenarchaeum massiliensisK8823_992	92	hypothetical protein			OG5_185721	DNA modification	Cenarchaeum Nitrososphaera
Candidatus Cenarchaeum massiliensisK8823_993	314	Transposase		IPR002559	OG5_136743	DNA modification	Cenarchaeum Nitrososphaera
Candidatus Cenarchaeum massiliensisK8823_1004	347	RelA/SpoT protein		IPR007685;IPR007685	OG5_149795	Signaling	Cenarchaeum Nitrososphaera
Candidatus Cenarchaeum massiliensisK8823_1034	72	hypothetical membrane protein			STG_0103	Unknown function	Cenarchaeum Nitrososphaera
Candidatus Cenarchaeum massiliensisK8823_1036	148	Transposase			STG_0047	Unknown function	Cenarchaeum Nitrososphaera
Candidatus Cenarchaeum massiliensisK8823_1079	233	Transposase		IPR002559	OG5_136743	DNA modification	Cenarchaeum Nitrososphaera
Candidatus Cenarchaeum massiliensisK8823_1080	79	Transposase		IPR002559	OG5_136743	DNA modification	Cenarchaeum Nitrososphaera
Candidatus Cenarchaeum massiliensisK8823_1088	1137	restriction endonuclease	K07317	IPR025931;IPR003356;IPR011639;IPR002052	OG5_145232	DNA modification	Cenarchaeum Nitrososphaera
Candidatus Cenarchaeum massiliensisK8823_1089	1088	DEAD-like helicase domain-containing protein		IPR014001;IPR001650;IPR000330;IPR025202;IPR001650;IPR001650;IPR014001	OG5_163337	DNA modification	Cenarchaeum Nitrososphaera
Candidatus Cenarchaeum massiliensisK8823_1174	371	hypothetical protein			OG5_185721	DNA modification	Cenarchaeum Nitrososphaera
Candidatus Cenarchaeum massiliensisK8823_1187	44	hypothetical protein			OG5_185721	DNA modification	Cenarchaeum Nitrososphaera
Candidatus Cenarchaeum massiliensisK8823_1188	119	hypothetical protein			OG5_185721	DNA modification	Cenarchaeum Nitrososphaera
Candidatus Cenarchaeum massiliensisK8823_1193	187	Transposase			OG5_127031	Unknown function	Cenarchaeum Nitrososphaera
Candidatus Cenarchaeum massiliensisK8823_1194	145	Transposase			OG5_127031	Unknown function	Cenarchaeum Nitrososphaera
Candidatus Cenarchaeum massiliensisK8823_1235	323	Transposase		IPR002559	OG5_136743	DNA modification	Cenarchaeum Nitrososphaera
Candidatus Cenarchaeum massiliensisK8823_1255	506	abortive infection protein		IPR030987	STG_0814	Unknown function	Cenarchaeum Nitrososphaera
Candidatus Cenarchaeum massiliensisK8823_118	196	DNA-3-methyladenine glycosylase II	K01247	IPR003265;IPR003265	OG5_130684	DNA modification	Cenarchaeum Nitrososphaera
Candidatus Cenarchaeum massiliensisK8823_1275	92	Transposase		IPR002559	STG_0032	Unknown function	Cenarchaeum Nitrososphaera
Candidatus Cenarchaeum massiliensisK8823_1276	179	Transposase			OG5_127031	Unknown function	Cenarchaeum Nitrososphaera
Candidatus Cenarchaeum massiliensisK8823_1306	144	death-on-curing family protein		IPR003812;IPR006440;IPR003812	OG5_144670	Signaling	Cenarchaeum Nitrososphaera
Candidatus Cenarchaeum massiliensisK8823_1382	274	Transposase		IPR002559	OG5_136743	DNA modification	Cenarchaeum Nitrososphaera

Candidatus Cenarchaeum massiliensisK8823_1383	79	Transposase		IPR002559	OG5_136743	DNA modification	Cenarchaeum Nitrososphaera
Candidatus Cenarchaeum massiliensisK8823_1392	109	hypothetical protein			OG5_127031	Unknown function	Cenarchaeum Nitrososphaera
Candidatus Cenarchaeum massiliensisK8823_1393	212	Transposase			OG5_127031	Unknown function	Cenarchaeum Nitrososphaera
Candidatus Cenarchaeum massiliensisK8823_1411	96	hypothetical protein			STG_0103	Unknown function	Cenarchaeum Nitrososphaera
Candidatus Cenarchaeum massiliensisK8823_1567	118	Transposase		IPR002559	OG5_136743	DNA modification	Cenarchaeum Nitrososphaera
Candidatus Cenarchaeum massiliensisK8823_1571	323	Transposase		IPR002559	OG5_136743	DNA modification	Cenarchaeum Nitrososphaera
Candidatus Cenarchaeum massiliensisK8823_1574	304	Transposase		IPR002559	OG5_136743	DNA modification	Cenarchaeum Nitrososphaera
Candidatus Cenarchaeum massiliensisK8823_1575	106	Transposase			STG_0047	Unknown function	Cenarchaeum Nitrososphaera
Candidatus Cenarchaeum massiliensisK8823_1577	304	Transposase		IPR002559	OG5_136743	DNA modification	Cenarchaeum Nitrososphaera
Candidatus Cenarchaeum massiliensisK8823_1579	332	Transposase			OG5_127031	Unknown function	Cenarchaeum Nitrososphaera
Candidatus Cenarchaeum massiliensisK8823_1600	77	Transposase		IPR002559	STG_0032	Unknown function	Cenarchaeum Nitrososphaera
Candidatus Cenarchaeum massiliensisK8823_1608	183	Transposase		IPR002559	STG_0032	Unknown function	Cenarchaeum Nitrososphaera
Candidatus Cenarchaeum massiliensisK8823_1609	179	Transposase			OG5_127031	Unknown function	Cenarchaeum Nitrososphaera
Candidatus Cenarchaeum massiliensisK8823_1610	134	Homeodomain-like domain-containing protein			OG5_127031	Unknown function	Cenarchaeum Nitrososphaera
Candidatus Cenarchaeum massiliensisK8823_1611	112	Transposase		IPR003346	OG5_127931	DNA modification	Cenarchaeum Nitrososphaera
Candidatus Cenarchaeum massiliensisK8823_1615	248	Transposase			OG5_136743	DNA modification	Cenarchaeum Nitrososphaera
Candidatus Cenarchaeum massiliensisK8823_1616	79	Transposase		IPR002559	OG5_136743	DNA modification	Cenarchaeum Nitrososphaera
Candidatus Cenarchaeum massiliensisK8823_1628	125	Homeodomain-like domain-containing protein			OG5_127031	Unknown function	Cenarchaeum Nitrososphaera
Candidatus Cenarchaeum massiliensisK8823_1629	179	Transposase			OG5_127031	Unknown function	Cenarchaeum Nitrososphaera
Candidatus Cenarchaeum massiliensisK8823_1633	150	hypothetical protein		IPR007527	STG_0269	Unknown function	Cenarchaeum Nitrososphaera
Candidatus Cenarchaeum massiliensisK8823_163	430	hypothetical membrane protein			STG_0143	Unknown function	Cenarchaeum Nitrososphaera
Candidatus Cenarchaeum massiliensisK8823_1661	79	Transposase		IPR002559	OG5_136743	DNA modification	Cenarchaeum Nitrososphaera
Candidatus Cenarchaeum massiliensisK8823_175	604	AAA-like domain-containing protein			OG5_179911	Unknown function	Cenarchaeum Nitrososphaera
Candidatus Cenarchaeum massiliensisK8823_176	480	Type II/IV secretion system protein	K07332	IPR001482	OG5_175628	Transport	Cenarchaeum Nitrososphaera
Candidatus Cenarchaeum massiliensisK8823_18	369	Transposase		IPR003346;IPR002525	OG5_127931	DNA modification	Cenarchaeum Nitrososphaera
Candidatus Cenarchaeum massiliensisK8823_22	135	hypothetical protein			STG_0203	Unknown function	Cenarchaeum Nitrososphaera
Candidatus Cenarchaeum massiliensisK8823_234	75	hypothetical protein			STG_0101	Unknown function	Cenarchaeum Nitrososphaera
Candidatus Cenarchaeum massiliensisK8823_1	431	hypothetical protein			OG5_185721	DNA modification	Cenarchaeum Nitrososphaera
Candidatus Cenarchaeum massiliensisK8823_25	230	SMF family protein	K04096	IPR003488	OG5_133325	DNA modification	Cenarchaeum Nitrososphaera
Candidatus Cenarchaeum massiliensisK8823_274	185	Transposase		IPR007527	STG_0269	Unknown function	Cenarchaeum Nitrososphaera
Candidatus Cenarchaeum massiliensisK8823_27	191	Transposase		IPR003346	OG5_127931	DNA modification	Cenarchaeum Nitrososphaera
Candidatus Cenarchaeum massiliensisK8823_31	115	hypothetical protein			STG_0047	Unknown function	Cenarchaeum Nitrososphaera
Candidatus Cenarchaeum massiliensisK8823_360	311	Transposase		IPR002559	OG5_136743	DNA modification	Cenarchaeum Nitrososphaera
Candidatus Cenarchaeum massiliensisK8823_368	427	DNA methylase domain-containing protein		IPR002941	OG5_181907	Unknown function	Cenarchaeum Nitrososphaera
Candidatus Cenarchaeum massiliensisK8823_370	369	Transposase		IPR003346;IPR002525	OG5_127931	DNA modification	Cenarchaeum Nitrososphaera
Candidatus Cenarchaeum massiliensisK8823_374	179	Transposase			OG5_127031	Unknown function	Cenarchaeum Nitrososphaera
Candidatus Cenarchaeum massiliensisK8823_384	240	hypothetical protein			OG5_185721	DNA modification	Cenarchaeum Nitrososphaera
Candidatus Cenarchaeum massiliensisK8823_394	761	CRISPR-associated helicase Cas3 family protein	K07012	IPR014001;IPR011545;IPR001650;IPR006674;IPR006483;IPR006474;IPR014001;IPR006483;IPR001650	OG5_138526	Stress & defense	Cenarchaeum Nitrososphaera
Candidatus Cenarchaeum massiliensisK8823_396	297	type I-C CRISPR-associated protein Cas7/Csd2		IPR006482;IPR006482;IPR013418	OG5_228703	Stress & defense	Cenarchaeum Nitrososphaera
Candidatus Cenarchaeum massiliensisK8823_397	211	CRISPR-associated protein Cas4	K07464	IPR013343;IPR022765	OG5_139298	Stress & defense	Cenarchaeum Nitrososphaera
Candidatus Cenarchaeum massiliensisK8823_399	107	CRISPR-associated endonuclease Cas2		IPR021127;IPR019199;IPR021127	OG5_159065	Stress & defense	Cenarchaeum Nitrososphaera
Candidatus Cenarchaeum massiliensisK8823_414	72	hypothetical protein			OG5_185721	DNA modification	Cenarchaeum Nitrososphaera
Candidatus Cenarchaeum massiliensisK8823_416	73	hypothetical protein			OG5_185721	DNA modification	Cenarchaeum Nitrososphaera
Candidatus Cenarchaeum massiliensisK8823_418	427	DNA methylase		IPR002941	OG5_181907	Unknown function	Cenarchaeum Nitrososphaera
Candidatus Cenarchaeum massiliensisK8823_420	286	Transposase		IPR025959	OG5_127031	Unknown function	Cenarchaeum Nitrososphaera
Candidatus Cenarchaeum massiliensisK8823_447	99	Transposase			STG_0101	Unknown function	Cenarchaeum Nitrososphaera
Candidatus Cenarchaeum massiliensisK8823_472	1353	N6 adenine-specific DNA methyltransferase		IPR002052	OG5_145232	DNA modification	Cenarchaeum Nitrososphaera
Candidatus Cenarchaeum massiliensisK8823_473	198	helicase		IPR025202	OG5_163337	DNA modification	Cenarchaeum Nitrososphaera
Candidatus Cenarchaeum massiliensisK8823_474	893	helicase		IPR001650;IPR014001;IPR000330;IPR001650;IPR014001;IPR001650	OG5_163337	DNA modification	Cenarchaeum Nitrososphaera
Candidatus Cenarchaeum massiliensisK8823_40	148	hypothetical protein		IPR007438	OG5_141713	Amino acid metabolism	Cenarchaeum Nitrososphaera
Candidatus Cenarchaeum massiliensisK8823_41	230	SMF family protein	K04096	IPR003488	OG5_133325	DNA modification	Cenarchaeum Nitrososphaera
Candidatus Cenarchaeum massiliensisK8823_3	320	Transposase			OG5_127031	Unknown function	Cenarchaeum Nitrososphaera
Candidatus Cenarchaeum massiliensisK8823_651	315	Transposase		IPR002559	OG5_136743	DNA modification	Cenarchaeum Nitrososphaera
Candidatus Cenarchaeum massiliensisK8823_657	331	transposase		IPR002559	OG5_136743	DNA modification	Cenarchaeum Nitrososphaera
Candidatus Cenarchaeum massiliensisK8823_660	331	Transposase DDE domain protein		IPR002559	OG5_136743	DNA modification	Cenarchaeum Nitrososphaera
Candidatus Cenarchaeum massiliensisK8823_662	76	Transposase DDE domain protein		IPR002559	OG5_136743	DNA modification	Cenarchaeum Nitrososphaera
Candidatus Cenarchaeum massiliensisK8823_663	179	Transposase			OG5_127031	Unknown function	Cenarchaeum Nitrososphaera
Candidatus Cenarchaeum massiliensisK8823_693	94	hypothetical protein			STG_0071	Unknown function	Cenarchaeum Nitrososphaera
Candidatus Cenarchaeum massiliensisK8823_697	148	Transposase			STG_0047	Unknown function	Cenarchaeum Nitrososphaera
Candidatus Cenarchaeum massiliensisK8823_719	38	Transposase		IPR004291	STG_0047	Unknown function	Cenarchaeum Nitrososphaera
Candidatus Cenarchaeum massiliensisK8823_755	103	hypothetical protein			OG5_185721	DNA modification	Cenarchaeum Nitrososphaera
Candidatus Cenarchaeum massiliensisK8823_756	180	hypothetical protein			OG5_185721	DNA modification	Cenarchaeum Nitrososphaera
Candidatus Cenarchaeum massiliensisK8823_69	136	Transposase-like protein			OG5_127031	Unknown function	Cenarchaeum Nitrososphaera
Candidatus Cenarchaeum massiliensisK8823_790	207	Transposase			OG5_127031	Unknown function	Cenarchaeum Nitrososphaera
Candidatus Cenarchaeum massiliensisK8823_794	1029	ATPase		IPR003593	OG5_234716	Unknown function	Cenarchaeum Nitrososphaera
Candidatus Cenarchaeum massiliensisK8823_872	286	Transposase		IPR025959	OG5_127031	Unknown function	Cenarchaeum Nitrososphaera
Candidatus Cenarchaeum massiliensisK8823_77	162	hypothetical protein		IPR025272		Unknown function	Species specific
Candidatus Cenarchaeum massiliensisK8823_888	163	hypothetical protein		IPR025272		Unknown function	Species specific
Candidatus Cenarchaeum massiliensisK8823_910	334	hypothetical protein				Unknown function	Species specific
Candidatus Cenarchaeum massiliensisK8823_934	68	hypothetical protein				Unknown function	Species specific
Candidatus Cenarchaeum massiliensisK8823_942	251	hypothetical protein		IPR007421		Unknown function	Species specific
Candidatus Cenarchaeum massiliensisK8823_944	123	DNA sulfur modification protein DndE		IPR014969		DNA modification	Species specific
Candidatus Cenarchaeum massiliensisK8823_945	664	DNA sulfur modification protein DndD		IPR017599		Transport	Species specific
Candidatus Cenarchaeum massiliensisK8823_950	123	Transposase		IPR004291		Unknown function	Species specific
Candidatus Cenarchaeum massiliensisK8823_954	158	hypothetical protein				Unknown function	Species specific
Candidatus Cenarchaeum massiliensisK8823_955	79	hypothetical protein				Unknown function	Species specific
Candidatus Cenarchaeum massiliensisK8823_956	84	hypothetical protein				Unknown function	Species specific
Candidatus Cenarchaeum massiliensisK8823_958	100	Transposase		IPR025959		Unknown function	Species specific
Candidatus Cenarchaeum massiliensisK8823_959	151	Transposase				Unknown function	Species specific
Candidatus Cenarchaeum massiliensisK8823_966	148	hypothetical protein				Unknown function	Species specific
Candidatus Cenarchaeum massiliensisK8823_968	130	hypothetical protein				Unknown function	Species specific
Candidatus Cenarchaeum massiliensisK8823_970	113	hypothetical protein				Unknown function	Species specific
Candidatus Cenarchaeum massiliensisK8823_971	65	hypothetical protein				Unknown function	Species specific
Candidatus Cenarchaeum massiliensisK8823_972	84	hypothetical protein				Unknown function	Species specific
Candidatus Cenarchaeum massiliensisK8823_984	284	HNH endonuclease		IPR003615		Misc	Species specific
Candidatus Cenarchaeum massiliensisK8823_987	510	hypothetical protein				Unknown function	Species specific
Candidatus Cenarchaeum massiliensisK8823_988	198	hypothetical protein				Unknown function	Species specific
Candidatus Cenarchaeum massiliensisK8823_991	117	hypothetical protein				Unknown function	Species specific
Candidatus Cenarchaeum massiliensisK8823_995	94	Transposase		IPR002525		Unknown function	Species specific
Candidatus Cenarchaeum massiliensisK8823_997	62	hypothetical protein				Unknown function	Species specific
Candidatus Cenarchaeum massiliensisK8823_999	123	hypothetical protein				Unknown function	Species specific
Candidatus Cenarchaeum massiliensisK8823_1000	80	Transposase		IPR004291		Unknown function	Species specific
Candidatus Cenarchaeum massiliensisK8823_1005	390	hypothetical protein		IPR014942		Unknown function	Species specific
Candidatus Cenarchaeum massiliensisK8823_1006	627	hypothetical protein				Unknown function	Species specific
Candidatus Cenarchaeum massiliensisK8823_1018	60	hypothetical protein				Unknown function	Species specific
Candidatus Cenarchaeum massiliensisK8823_1020	81	Transposase		IPR002525		Unknown function	Species specific
Candidatus Cenarchaeum massiliensisK8823_1022	126	hypothetical membrane protein				Unknown function	Species specific
Candidatus Cenarchaeum massiliensisK8823_1024	95	hypothetical protein		IPR004291		Unknown function	Species specific

Candidatus Cenarchaeum massiliensis	K8823_1044	184	hypothetical protein		Unknown function	Species specific
Candidatus Cenarchaeum massiliensis	K8823_9	167	hypothetical protein		Unknown function	Species specific
Candidatus Cenarchaeum massiliensis	K8823_1082	47	Transposase	IPR003346	Unknown function	Species specific
Candidatus Cenarchaeum massiliensis	K8823_1083	191	hypothetical protein		Unknown function	Species specific
Candidatus Cenarchaeum massiliensis	K8823_1084	284	hypothetical membrane protein		Unknown function	Species specific
Candidatus Cenarchaeum massiliensis	K8823_1085	95	hypothetical protein		Unknown function	Species specific
Candidatus Cenarchaeum massiliensis	K8823_1086	66	hypothetical protein		Unknown function	Species specific
Candidatus Cenarchaeum massiliensis	K8823_1087	126	hypothetical protein		Unknown function	Species specific
Candidatus Cenarchaeum massiliensis	K8823_1090	70	Transposase	IPR004291	Unknown function	Species specific
Candidatus Cenarchaeum massiliensis	K8823_1101	52	hypothetical protein		Unknown function	Species specific
Candidatus Cenarchaeum massiliensis	K8823_106	54	hypothetical protein	IPR014044	Unknown function	Species specific
Candidatus Cenarchaeum massiliensis	K8823_1172	110	hypothetical protein		Unknown function	Species specific
Candidatus Cenarchaeum massiliensis	K8823_1173	93	hypothetical protein		Unknown function	Species specific
Candidatus Cenarchaeum massiliensis	K8823_1175	103	hypothetical protein		Unknown function	Species specific
Candidatus Cenarchaeum massiliensis	K8823_1178	239	hypothetical protein		Misc	Species specific
Candidatus Cenarchaeum massiliensis	K8823_1180	39	hypothetical protein		Unknown function	Species specific
Candidatus Cenarchaeum massiliensis	K8823_1183	103	hypothetical protein		Unknown function	Species specific
Candidatus Cenarchaeum massiliensis	K8823_1184	126	hypothetical protein		Unknown function	Species specific
Candidatus Cenarchaeum massiliensis	K8823_1185	75	hypothetical protein		Unknown function	Species specific
Candidatus Cenarchaeum massiliensis	K8823_1186	95	hypothetical protein		Unknown function	Species specific
Candidatus Cenarchaeum massiliensis	K8823_1189	165	hypothetical protein		Unknown function	Species specific
Candidatus Cenarchaeum massiliensis	K8823_1190	151	hypothetical protein		Unknown function	Species specific
Candidatus Cenarchaeum massiliensis	K8823_1195	110	hypothetical protein		Unknown function	Species specific
Candidatus Cenarchaeum massiliensis	K8823_1207	119	hypothetical membrane protein		Unknown function	Species specific
Candidatus Cenarchaeum massiliensis	K8823_113	126	hypothetical protein		Unknown function	Species specific
Candidatus Cenarchaeum massiliensis	K8823_1223	131	hypothetical protein		Unknown function	Species specific
Candidatus Cenarchaeum massiliensis	K8823_114	148	hypothetical protein		Unknown function	Species specific
Candidatus Cenarchaeum massiliensis	K8823_1234	97	hypothetical protein	IPR027395	Unknown function	Species specific
Candidatus Cenarchaeum massiliensis	K8823_1237	138	hypothetical protein		Unknown function	Species specific
Candidatus Cenarchaeum massiliensis	K8823_1251	155	AN1-like zinc finger protein	IPR000058;IPR000058	Unknown function	Species specific
Candidatus Cenarchaeum massiliensis	K8823_1252	135	hypothetical protein		Unknown function	Species specific
Candidatus Cenarchaeum massiliensis	K8823_1253	536	HNH endonuclease	IPR002711	Unknown function	Species specific
Candidatus Cenarchaeum massiliensis	K8823_1254	52	Transposase	IPR002525	Unknown function	Species specific
Candidatus Cenarchaeum massiliensis	K8823_1262	103	hypothetical protein		Unknown function	Species specific
Candidatus Cenarchaeum massiliensis	K8823_1277	49	hypothetical protein	IPR025959	Unknown function	Species specific
Candidatus Cenarchaeum massiliensis	K8823_1278	60	hypothetical protein		Unknown function	Species specific
Candidatus Cenarchaeum massiliensis	K8823_1279	185	hypothetical protein		Unknown function	Species specific
Candidatus Cenarchaeum massiliensis	K8823_1280	129	hypothetical membrane protein		Unknown function	Species specific
Candidatus Cenarchaeum massiliensis	K8823_1324	78	HicA toxin domain-containing protein	IPR012933	Unknown function	Species specific
Candidatus Cenarchaeum massiliensis	K8823_1325	70	hypothetical protein		Unknown function	Species specific
Candidatus Cenarchaeum massiliensis	K8823_1331	130	Transposase	IPR002525	Unknown function	Species specific
Candidatus Cenarchaeum massiliensis	K8823_1332	85	Transposase	IPR003346	Unknown function	Species specific
Candidatus Cenarchaeum massiliensis	K8823_1344	75	hypothetical membrane protein		Unknown function	Species specific
Candidatus Cenarchaeum massiliensis	K8823_1385	595	AlwI restriction endonuclease	IPR018573	DNA modification	Species specific
Candidatus Cenarchaeum massiliensis	K8823_1387	124	hypothetical protein		Unknown function	Species specific
Candidatus Cenarchaeum massiliensis	K8823_1388	138	hypothetical membrane protein		Unknown function	Species specific
Candidatus Cenarchaeum massiliensis	K8823_1394	95	hypothetical protein		Unknown function	Species specific
Candidatus Cenarchaeum massiliensis	K8823_1395	46	hypothetical protein		Unknown function	Species specific
Candidatus Cenarchaeum massiliensis	K8823_1396	58	Transposase	IPR003346	Unknown function	Species specific
Candidatus Cenarchaeum massiliensis	K8823_1397	62	hypothetical protein		Unknown function	Species specific
Candidatus Cenarchaeum massiliensis	K8823_1417	137	Transposase	IPR004291	Unknown function	Species specific
Candidatus Cenarchaeum massiliensis	K8823_1418	107	hypothetical protein		Unknown function	Species specific
Candidatus Cenarchaeum massiliensis	K8823_1451	112	hypothetical protein		Unknown function	Species specific
Candidatus Cenarchaeum massiliensis	K8823_1470	119	hypothetical protein		Unknown function	Species specific
Candidatus Cenarchaeum massiliensis	K8823_1490	285	methyltransferase	IPR007213	Unknown function	Species specific
Candidatus Cenarchaeum massiliensis	K8823_14	1138	hypothetical protein		Unknown function	Species specific
Candidatus Cenarchaeum massiliensis	K8823_1516	116	hypothetical protein		Unknown function	Species specific
Candidatus Cenarchaeum massiliensis	K8823_1517	162	hypothetical protein		Unknown function	Species specific
Candidatus Cenarchaeum massiliensis	K8823_1518	146	hypothetical protein		Unknown function	Species specific
Candidatus Cenarchaeum massiliensis	K8823_1519	80	hypothetical protein		Unknown function	Species specific
Candidatus Cenarchaeum massiliensis	K8823_1520	322	hypothetical protein		Unknown function	Species specific
Candidatus Cenarchaeum massiliensis	K8823_1521	946	hypothetical protein		Unknown function	Species specific
Candidatus Cenarchaeum massiliensis	K8823_1522	188	hypothetical protein		Unknown function	Species specific
Candidatus Cenarchaeum massiliensis	K8823_1523	103	hypothetical protein		Unknown function	Species specific
Candidatus Cenarchaeum massiliensis	K8823_1524	494	hypothetical protein		Unknown function	Species specific
Candidatus Cenarchaeum massiliensis	K8823_1525	124	hypothetical protein		Unknown function	Species specific
Candidatus Cenarchaeum massiliensis	K8823_1526	108	hypothetical protein		Unknown function	Species specific
Candidatus Cenarchaeum massiliensis	K8823_1528	147	hypothetical protein		Unknown function	Species specific
Candidatus Cenarchaeum massiliensis	K8823_1529	65	hypothetical protein		Unknown function	Species specific
Candidatus Cenarchaeum massiliensis	K8823_1530	148	hypothetical protein		Unknown function	Species specific
Candidatus Cenarchaeum massiliensis	K8823_1531	210	HNH nuclease domain-containing protein	IPR003615;IPR002711	Unknown function	Species specific
Candidatus Cenarchaeum massiliensis	K8823_1532	150	hypothetical protein		Unknown function	Species specific
Candidatus Cenarchaeum massiliensis	K8823_1533	107	hypothetical protein		Unknown function	Species specific
Candidatus Cenarchaeum massiliensis	K8823_1534	97	hypothetical protein		Unknown function	Species specific
Candidatus Cenarchaeum massiliensis	K8823_1535	33	hypothetical protein		Unknown function	Species specific
Candidatus Cenarchaeum massiliensis	K8823_1537	105	hypothetical protein		Unknown function	Species specific
Candidatus Cenarchaeum massiliensis	K8823_1540	152	hypothetical protein		Misc	Species specific
Candidatus Cenarchaeum massiliensis	K8823_1541	169	hypothetical protein		Unknown function	Species specific
Candidatus Cenarchaeum massiliensis	K8823_1542	115	hypothetical protein		Unknown function	Species specific
Candidatus Cenarchaeum massiliensis	K8823_1543	105	hypothetical protein		Unknown function	Species specific
Candidatus Cenarchaeum massiliensis	K8823_1544	224	hypothetical protein		Unknown function	Species specific
Candidatus Cenarchaeum massiliensis	K8823_1546	132	hypothetical protein		Unknown function	Species specific
Candidatus Cenarchaeum massiliensis	K8823_1547	131	hypothetical membrane protein		Unknown function	Species specific
Candidatus Cenarchaeum massiliensis	K8823_1548	287	hypothetical protein		Unknown function	Species specific
Candidatus Cenarchaeum massiliensis	K8823_1549	174	hypothetical protein		Unknown function	Species specific
Candidatus Cenarchaeum massiliensis	K8823_1550	154	hypothetical protein		Unknown function	Species specific
Candidatus Cenarchaeum massiliensis	K8823_1551	163	hypothetical protein		Unknown function	Species specific
Candidatus Cenarchaeum massiliensis	K8823_1552	323	hypothetical protein		Unknown function	Species specific
Candidatus Cenarchaeum massiliensis	K8823_1553	181	hypothetical protein		Unknown function	Species specific
Candidatus Cenarchaeum massiliensis	K8823_1554	283	hypothetical protein		Unknown function	Species specific
Candidatus Cenarchaeum massiliensis	K8823_1555	1155	hypothetical protein		Unknown function	Species specific
Candidatus Cenarchaeum massiliensis	K8823_1556	201	hypothetical protein		Unknown function	Species specific
Candidatus Cenarchaeum massiliensis	K8823_1557	140	hypothetical protein		Unknown function	Species specific
Candidatus Cenarchaeum massiliensis	K8823_1558	125	hypothetical protein		Unknown function	Species specific
Candidatus Cenarchaeum massiliensis	K8823_1559	424	hypothetical protein		Unknown function	Species specific
Candidatus Cenarchaeum massiliensis	K8823_1560	156	hypothetical protein		Unknown function	Species specific
Candidatus Cenarchaeum massiliensis	K8823_1561	188	hypothetical protein		Unknown function	Species specific
Candidatus Cenarchaeum massiliensis	K8823_1563	577	hypothetical protein		Unknown function	Species specific

Candidatus Cenarchaeum massiliensis	K8823_1564	258	hypothetical protein			Unknown function	Species specific
Candidatus Cenarchaeum massiliensis	K8823_1565	318	hypothetical protein			Unknown function	Species specific
Candidatus Cenarchaeum massiliensis	K8823_1569	65	hypothetical protein			Unknown function	Species specific
Candidatus Cenarchaeum massiliensis	K8823_1570	95	hypothetical protein			Unknown function	Species specific
Candidatus Cenarchaeum massiliensis	K8823_1576	108	hypothetical protein			Unknown function	Species specific
Candidatus Cenarchaeum massiliensis	K8823_1578	95	Winged helix-turn helix domain-containing protein		IPR025959	Unknown function	Species specific
Candidatus Cenarchaeum massiliensis	K8823_1592	137	hypothetical protein			Unknown function	Species specific
Candidatus Cenarchaeum massiliensis	K8823_1597	103	DNA sulfur modification protein DndE		IPR014969	DNA modification	Species specific
Candidatus Cenarchaeum massiliensis	K8823_1601	98	Transposase		IPR002525	Unknown function	Species specific
Candidatus Cenarchaeum massiliensis	K8823_1602	102	hypothetical protein			Unknown function	Species specific
Candidatus Cenarchaeum massiliensis	K8823_1613	136	hypothetical protein			Unknown function	Species specific
Candidatus Cenarchaeum massiliensis	K8823_1614	226	hypothetical membrane protein			Unknown function	Species specific
Candidatus Cenarchaeum massiliensis	K8823_160	885	hypothetical protein			Unknown function	Species specific
Candidatus Cenarchaeum massiliensis	K8823_1617	359	hypothetical protein			Unknown function	Species specific
Candidatus Cenarchaeum massiliensis	K8823_1618	49	transcriptional regulator AbrB family		IPR007159;IPR007159	Transcription	Species specific
Candidatus Cenarchaeum massiliensis	K8823_161	245	hypothetical protein			Unknown function	Species specific
Candidatus Cenarchaeum massiliensis	K8823_1620	127	hypothetical protein			Unknown function	Species specific
Candidatus Cenarchaeum massiliensis	K8823_1623	164	hypothetical protein			Unknown function	Species specific
Candidatus Cenarchaeum massiliensis	K8823_1626	91	Transposase		IPR003346	Unknown function	Species specific
Candidatus Cenarchaeum massiliensis	K8823_1627	73	hypothetical protein			Unknown function	Species specific
Candidatus Cenarchaeum massiliensis	K8823_162	121	hypothetical protein			Unknown function	Species specific
Candidatus Cenarchaeum massiliensis	K8823_1631	229	hypothetical protein			Unknown function	Species specific
Candidatus Cenarchaeum massiliensis	K8823_165	152	hypothetical protein			Unknown function	Species specific
Candidatus Cenarchaeum massiliensis	K8823_1654	100	hypothetical protein		IPR025959	Unknown function	Species specific
Candidatus Cenarchaeum massiliensis	K8823_166	219	hypothetical membrane protein			Unknown function	Species specific
Candidatus Cenarchaeum massiliensis	K8823_1668	125	hypothetical protein			Unknown function	Species specific
Candidatus Cenarchaeum massiliensis	K8823_1680	77	Transposase		IPR002559	Unknown function	Species specific
Candidatus Cenarchaeum massiliensis	K8823_1681	128	Transposase		IPR003346	Unknown function	Species specific
Candidatus Cenarchaeum massiliensis	K8823_1682	327	hypothetical protein			Unknown function	Species specific
Candidatus Cenarchaeum massiliensis	K8823_1683	77	Transposase		IPR002559	Unknown function	Species specific
Candidatus Cenarchaeum massiliensis	K8823_1695	162	Isocitrate/isopropylmalate dehydrogenase	K00052	IPR024084;IPR001804	Amino acid metabolism	Species specific
Candidatus Cenarchaeum massiliensis	K8823_1696	186	2-isopropylmalate synthase	K01649	IPR013709;IPR013709	Misc	Species specific
Candidatus Cenarchaeum massiliensis	K8823_173	143	hypothetical protein			Unknown function	Species specific
Candidatus Cenarchaeum massiliensis	K8823_174	137	hypothetical protein			Unknown function	Species specific
Candidatus Cenarchaeum massiliensis	K8823_182	44	cation-binding protein			Unknown function	Species specific
Candidatus Cenarchaeum massiliensis	K8823_190	101	hypothetical membrane protein			Unknown function	Species specific
Candidatus Cenarchaeum massiliensis	K8823_191	250	hypothetical protein			Unknown function	Species specific
Candidatus Cenarchaeum massiliensis	K8823_21	259	hypothetical protein			Unknown function	Species specific
Candidatus Cenarchaeum massiliensis	K8823_233	114	hypothetical protein			Unknown function	Species specific
Candidatus Cenarchaeum massiliensis	K8823_23	92	hypothetical protein			Unknown function	Species specific
Candidatus Cenarchaeum massiliensis	K8823_243	105	hypothetical protein			Unknown function	Species specific
Candidatus Cenarchaeum massiliensis	K8823_24	62	Transposase		IPR002525	Unknown function	Species specific
Candidatus Cenarchaeum massiliensis	K8823_26	113	Transposase		IPR002525	Unknown function	Species specific
Candidatus Cenarchaeum massiliensis	K8823_305	129	hypothetical membrane protein			Unknown function	Species specific
Candidatus Cenarchaeum massiliensis	K8823_29	109	hypothetical protein			Unknown function	Species specific
Candidatus Cenarchaeum massiliensis	K8823_30	590	hypothetical protein			Unknown function	Species specific
Candidatus Cenarchaeum massiliensis	K8823_346	295	hydrolase		IPR003615;IPR002711	Unknown function	Species specific
Candidatus Cenarchaeum massiliensis	K8823_366	141	nitrous oxide reductase		IPR028096	Oxidation-reduction	Species specific
Candidatus Cenarchaeum massiliensis	K8823_369	163	restriction endonuclease		IPR019042	Misc	Species specific
Candidatus Cenarchaeum massiliensis	K8823_371	145	hypothetical protein			Unknown function	Species specific
Candidatus Cenarchaeum massiliensis	K8823_372	63	hypothetical protein			Unknown function	Species specific
Candidatus Cenarchaeum massiliensis	K8823_373	49	hypothetical protein		IPR025959	Unknown function	Species specific
Candidatus Cenarchaeum massiliensis	K8823_375	95	hypothetical protein			Unknown function	Species specific
Candidatus Cenarchaeum massiliensis	K8823_376	107	hypothetical protein			Unknown function	Species specific
Candidatus Cenarchaeum massiliensis	K8823_377	152	hypothetical protein			Unknown function	Species specific
Candidatus Cenarchaeum massiliensis	K8823_383	310	hypothetical protein		IPR002793	Unknown function	Species specific
Candidatus Cenarchaeum massiliensis	K8823_32	100	hypothetical protein			Unknown function	Species specific
Candidatus Cenarchaeum massiliensis	K8823_385	86	hypothetical protein			Unknown function	Species specific
Candidatus Cenarchaeum massiliensis	K8823_415	77	hypothetical protein			Unknown function	Species specific
Candidatus Cenarchaeum massiliensis	K8823_419	128	hypothetical protein			Unknown function	Species specific
Candidatus Cenarchaeum massiliensis	K8823_440	62	hypothetical membrane protein			Unknown function	Species specific
Candidatus Cenarchaeum massiliensis	K8823_442	94	Transposase		IPR002525	Unknown function	Species specific
Candidatus Cenarchaeum massiliensis	K8823_443	78	hypothetical protein			Unknown function	Species specific
Candidatus Cenarchaeum massiliensis	K8823_2	127	hypothetical protein			Unknown function	Species specific
Candidatus Cenarchaeum massiliensis	K8823_464	109	hypothetical protein			Unknown function	Species specific
Candidatus Cenarchaeum massiliensis	K8823_488	463	hypothetical protein			Unknown function	Species specific
Candidatus Cenarchaeum massiliensis	K8823_42	191	hypothetical membrane protein			Unknown function	Species specific
Candidatus Cenarchaeum massiliensis	K8823_43	217	hypothetical membrane protein			Unknown function	Species specific
Candidatus Cenarchaeum massiliensis	K8823_558	191	hypothetical protein			Unknown function	Species specific
Candidatus Cenarchaeum massiliensis	K8823_44	192	hypothetical protein			Unknown function	Species specific
Candidatus Cenarchaeum massiliensis	K8823_568	105	hypothetical protein			Unknown function	Species specific
Candidatus Cenarchaeum massiliensis	K8823_45	355	hypothetical protein			Unknown function	Species specific
Candidatus Cenarchaeum massiliensis	K8823_572	124	transposase		IPR002525	Unknown function	Species specific
Candidatus Cenarchaeum massiliensis	K8823_573	62	hypothetical protein			Unknown function	Species specific
Candidatus Cenarchaeum massiliensis	K8823_46	288	hypothetical protein			Unknown function	Species specific
Candidatus Cenarchaeum massiliensis	K8823_582	34	hypothetical protein			Unknown function	Species specific
Candidatus Cenarchaeum massiliensis	K8823_4	142	hypothetical protein			Unknown function	Species specific
Candidatus Cenarchaeum massiliensis	K8823_631	177	hypothetical protein			Unknown function	Species specific
Candidatus Cenarchaeum massiliensis	K8823_632	103	hypothetical protein			Unknown function	Species specific
Candidatus Cenarchaeum massiliensis	K8823_652	971	LVIVD repeat-containing protein		IPR013211	Unknown function	Species specific
Candidatus Cenarchaeum massiliensis	K8823_654	225	Eco47II family restriction endonuclease		IPR019057	DNA modification	Species specific
Candidatus Cenarchaeum massiliensis	K8823_656	2904	LVIVD repeat-containing protein		IPR013211	Unknown function	Species specific
Candidatus Cenarchaeum massiliensis	K8823_659	147	Transposase DDE domain protein			Unknown function	Species specific
Candidatus Cenarchaeum massiliensis	K8823_664	95	hypothetical protein		IPR025959	Unknown function	Species specific
Candidatus Cenarchaeum massiliensis	K8823_691	98	hypothetical protein		IPR004291	Unknown function	Species specific
Candidatus Cenarchaeum massiliensis	K8823_692	110	hypothetical membrane protein			Unknown function	Species specific
Candidatus Cenarchaeum massiliensis	K8823_695	110	hypothetical protein			Unknown function	Species specific
Candidatus Cenarchaeum massiliensis	K8823_698	131	hypothetical protein			Unknown function	Species specific
Candidatus Cenarchaeum massiliensis	K8823_700	122	Transposase		IPR004291	Unknown function	Species specific
Candidatus Cenarchaeum massiliensis	K8823_712	80	hypothetical protein			Unknown function	Species specific
Candidatus Cenarchaeum massiliensis	K8823_713	177	hypothetical protein			Unknown function	Species specific
Candidatus Cenarchaeum massiliensis	K8823_716	263	hypothetical protein		IPR021398	Unknown function	Species specific
Candidatus Cenarchaeum massiliensis	K8823_717	100	hypothetical protein		IPR004291	Unknown function	Species specific
Candidatus Cenarchaeum massiliensis	K8823_720	75	hypothetical protein			Unknown function	Species specific
Candidatus Cenarchaeum massiliensis	K8823_749	31	hypothetical protein			Unknown function	Species specific
Candidatus Cenarchaeum massiliensis	K8823_752	184	hypothetical protein		IPR025959	Unknown function	Species specific
Candidatus Cenarchaeum massiliensis	K8823_758	72	Transposase		IPR003346	Unknown function	Species specific
Candidatus Cenarchaeum massiliensis	K8823_759	105	Transposase		IPR003346	Unknown function	Species specific

Candidatus Cenarchaeum massiliensis	K8823_762	39 hypothetical protein		Unknown function	Species specific
Candidatus Cenarchaeum massiliensis	K8823_764	239 restriction endonuclease		Misc	Species specific
Candidatus Cenarchaeum massiliensis	K8823_765	126 hypothetical protein		Unknown function	Species specific
Candidatus Cenarchaeum massiliensis	K8823_766	75 hypothetical protein		Unknown function	Species specific
Candidatus Cenarchaeum massiliensis	K8823_767	95 hypothetical protein		Unknown function	Species specific
Candidatus Cenarchaeum massiliensis	K8823_772	114 hypothetical protein	IPR014969	DNA modification	Species specific
Candidatus Cenarchaeum massiliensis	K8823_775	65 hypothetical protein		Unknown function	Species specific
Candidatus Cenarchaeum massiliensis	K8823_782	785 SIR2 family protein		Unknown function	Species specific
Candidatus Cenarchaeum massiliensis	K8823_785	158 hypothetical protein		Unknown function	Species specific
Candidatus Cenarchaeum massiliensis	K8823_787	147 Transposase	IPR002525	Unknown function	Species specific
Candidatus Cenarchaeum massiliensis	K8823_791	62 hypothetical protein	IPR025959	Unknown function	Species specific
Candidatus Cenarchaeum massiliensis	K8823_796	105 hypothetical protein		Unknown function	Species specific
Candidatus Cenarchaeum massiliensis	K8823_797	170 hypothetical protein		Unknown function	Species specific
Candidatus Cenarchaeum massiliensis	K8823_808	103 hypothetical protein	IPR002559	Unknown function	Species specific
Candidatus Cenarchaeum massiliensis	K8823_809	118 hypothetical protein		Unknown function	Species specific
Candidatus Cenarchaeum massiliensis	K8823_810	282 hypothetical protein		Unknown function	Species specific
Candidatus Cenarchaeum massiliensis	K8823_861	106 hypothetical protein		Unknown function	Species specific
Candidatus Cenarchaeum massiliensis	K8823_886	137 hypothetical protein		Unknown function	Species specific

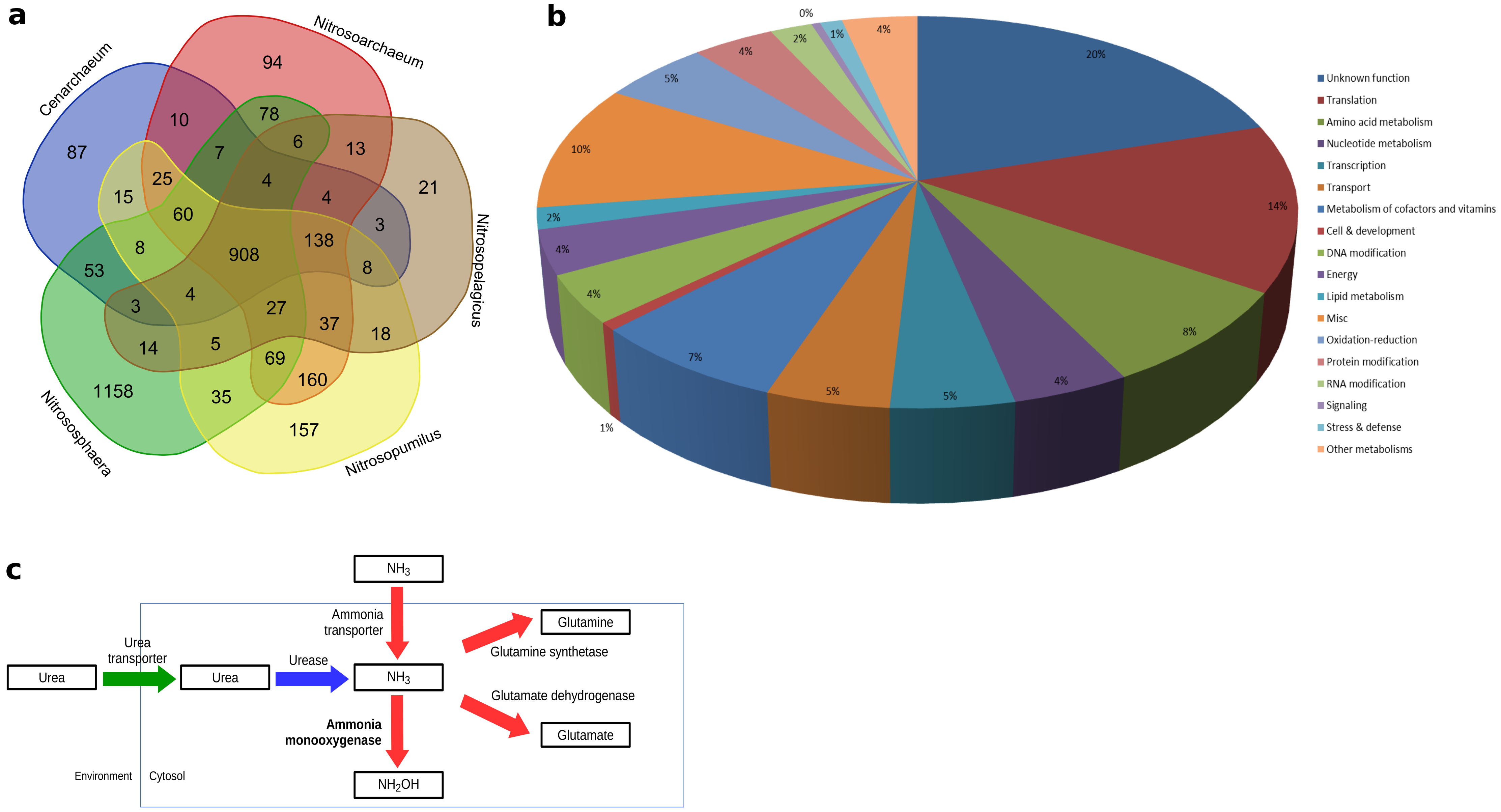

Figure S2: Characterization of the genome of *Candidatus Cenarchaeum massiliensis*, the main symbiont of *O. minuta*, compared to other Thaumarchaeota species. **a** :Five-way-Venn-diagram illustrating orthologous genes clusters generated using OrthoMCL analysis on predicted proteins of *Cenarchaeum symbiosum* and *Cenarchaeum massiliensis* (blue circle), *Nitrosoarchaeum koreensis* and *Nitrosoarchaeum limnia*(red circle), *Nitrosopelagicus brevis* (brown circle), *Nitrosopumilus maritimus* and N*itrosopumilus koreensis* (yellow circle) and three soil Thaumarchaeota (*Nitrososphaera viennensis, Nitrososphaera gargensis* and *Nitrososphaera evergladensis*; green circle). Venn-diagram is generated using a webtool (http://bioinformatics.psb.ugent.be/webtools/Venn/). **b**: Functional classes of the 908 Thaumarchaeota conserved gene families. **c**: The nitrogen metabolism in Thaumarchaeota. Conserved steps in all Thaumarchaeota (red arrows), conserved step only in soil Thaumarchaeota and *Cenarchaeum symbiosum* (blue arrow) and conserved step only in soil Thaumarchaeota (green arrow) are shown.

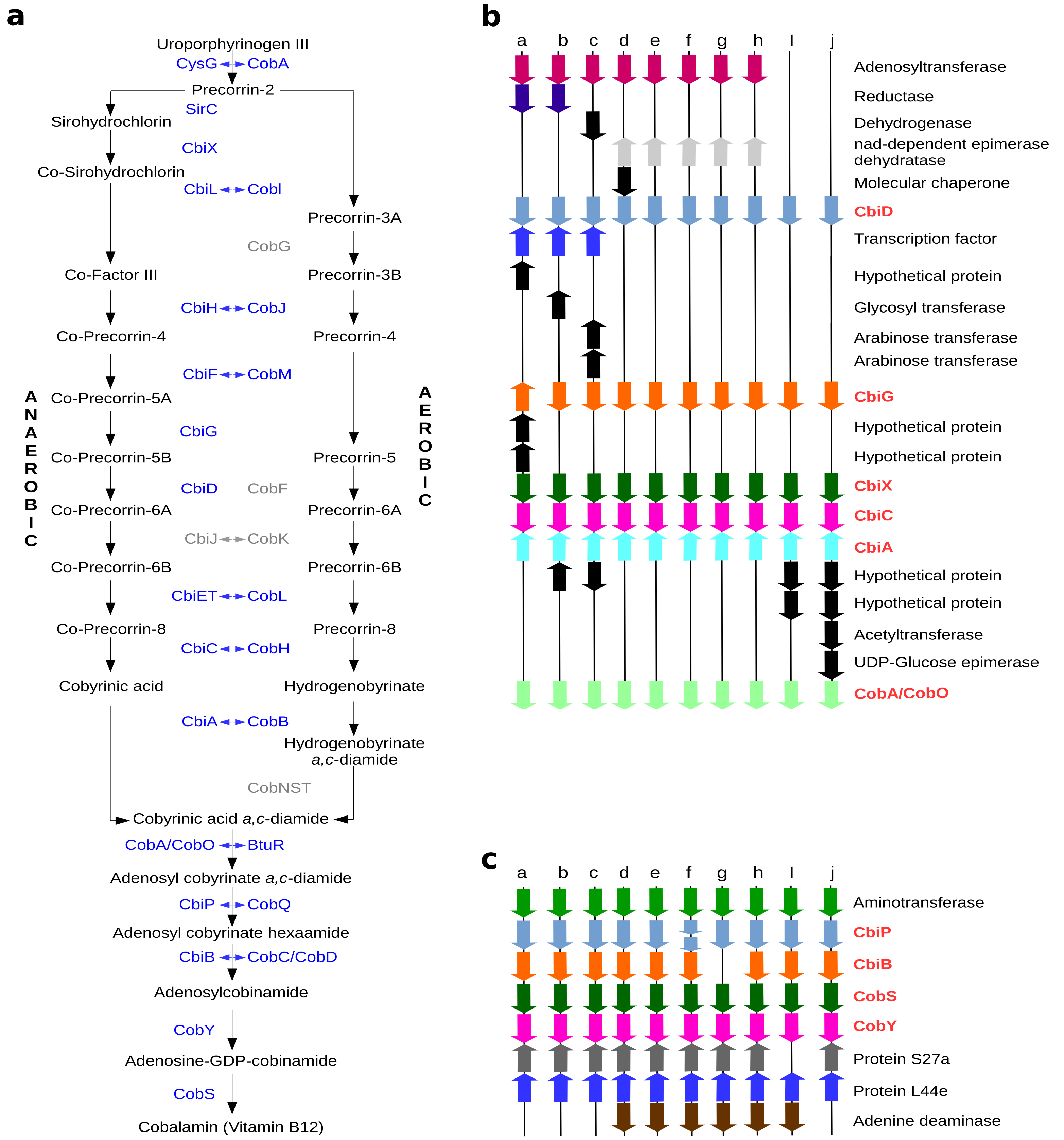

Figure S3: The cobalamin synthesis genes in Thaumarchaeota.
a:Pathway of the cobalamin synthesis adapted from Doxey et al., 2015. Blue arrows indicate shared enzymatic steps between aerobic and anaerobic pathways. All enzymes of the anaerobic pathway are retrieved (indicated in blue) except CbiJ (indicated in grey). **b**:Microsynteny analysis of Thaumarchaeota genomic segments bearing six genes coding for early enzymatic steps of cobalamin synthesis. **c**:Microsynteny analysis of Thaumarchaeota genomic segments bearing four genes coding for late enzymatic steps of cobalamin synthesis. Segments “a” to “j” correspond to genomic segments from *Nitrososphaera gargensis, Nitrososphaera viennensis, Nitrososphaera evergladensis,Nitrosopelagicus brevis, Nitrosoarchaeum koreensis, Nitrosoarchaeum limnia, Nitrosopumilus koreensis, Nitrosopumilus maritimus, Cenarchaeum symbiosum* and *Candidatus Cenarchaeum massiliensis*, respectively. Arrows of the same color indicate orthologous genes and black arrows represent genes without orthology relationship in these regions from OrthoMCL analysis.

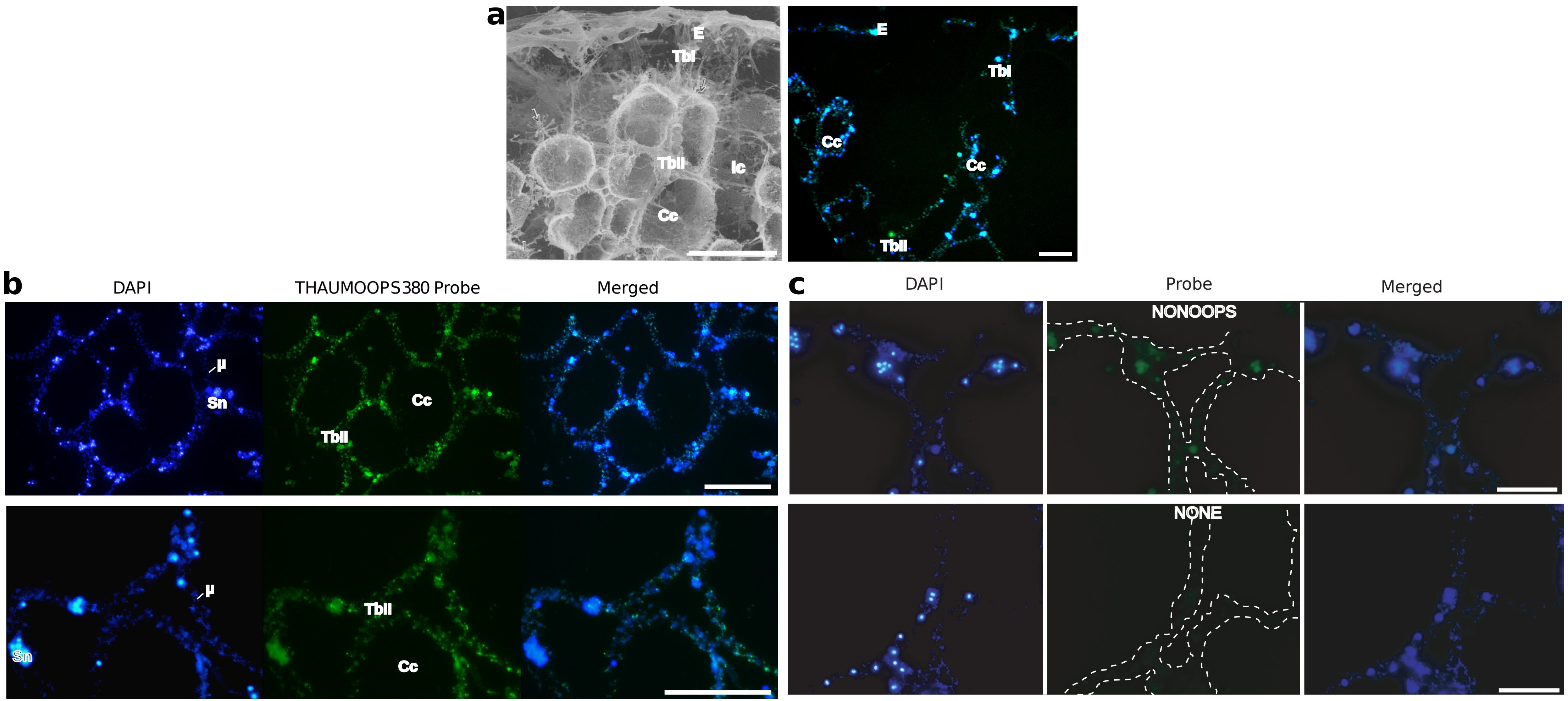

Figure S4: Localization by Card-FISH of the thaumarcheota *Candidatus Cenarchaeum massiliens* in the tissues of *Oopsacas minuta*. a: Confocal picture (on the right) showing its localization in the different parts of the trabecular syncitium (Tb) : the Ecto -some (E) or dermal layer, the Choanochambers (CC) or flagellated chambers, the inhalant canals (Ic), recognized by comparison with SEM pictu -res (on the left) (Boury-Esnault and Vacelet, 1994) scale bars = 150 µm. **b:** Localization in the trabecular syncitium shown at higher magnification in two different individuals with the THAUMOOPS380-HRP probe (green) and conterstained with DAPI (blue), µ: microorganisms DNA, Sn: Sponge nuclei, scale bars = 70µm. **c:** Card-FISH negative controls: with a random probe with no 100% match in the metagenome (NONOOPS-HRP), or no probe, evidencing that sponge cells have an endogenous peroxidase activity uncompletly abolished by 0.3% H_2O_2.

1. Altschul SF, Gish W, Miller W, Myers EW, Lipman DJ. Basic local alignment search tool. J Mol Biol. 1990;215(3):403–10.

2. Zhu W, Lomsadze A, Borodovsky M. Ab initio gene identification in metagenomic sequences. Nucleic Acids Res. 2010;38(12):e132.

3. Chan PP, Lowe TM. tRNAscan-SE: Searching for tRNA Genes in Genomic Sequences. Methods Mol Biol. 2019;1962:1–14.

4. Stieglmeier M, Klingl A, Alves RJE, Rittmann SKMR, Melcher M, Leisch N, et al. Nitrososphaera viennensis gen. nov., sp. nov., an aerobic and mesophilic, ammonia-oxidizing archaeon from soil and a member of the archaeal phylum Thaumarchaeota. Int J Syst Evol Microbiol. 2014;64(Pt 8):2738–52.

5. Spang A, Poehlein A, Offre P, Zumbrägel S, Haider S, Rychlik N, et al. The genome of the ammonia-oxidizing *Candidatus Nitrososphaera gargensis*: insights into metabolic versatility and environmental adaptations. Environ Microbiol. 2012;14(12):3122–45.

6. Zhalnina KV, Dias R, Leonard MT, Dorr de Quadros P, Camargo FAO, Drew JC, et al. Genome sequence of *Candidatus Nitrososphaera evergladensis* from group I.1b enriched from Everglades soil reveals novel genomic features of the ammonia-oxidizing archaea. PLoS One. 2014;9(7):e101648.

7. Moriya Y, Itoh M, Okuda S, Yoshizawa AC, Kanehisa M. KAAS: an automatic genome annotation and pathway reconstruction server. Nucleic Acids Res. 2007;35:W182-185.

8. Fischer S, Brunk BP, Chen F, Gao X, Harb OS, Iodice JB, et al. Using OrthoMCL to assign proteins to OrthoMCL-DB groups or to cluster proteomes into new ortholog groups. Curr Protoc Bioinformatics. 2011;Chapter 6:6.12.1-6.12.19.

9. Conesa A, Götz S, García-Gómez JM, Terol J, Talón M, Robles M. Blast2GO: a universal tool for annotation, visualization and analysis in functional genomics research. Bioinformatics. 2005;21(18):3674–6.

10. Santoro AE, Dupont CL, Richter RA, Craig MT, Carini P, McIlvin MR, et al. Genomic and proteomic characterization of "*Candidatus Nitrosopelagicus brevis*": an ammonia-oxidizing archaeon from the open ocean. Proc Natl Acad Sci U S A. 2015;112(4):1173–8.

11. Hallam SJ, Konstantinidis KT, Putnam N, Schleper C, Watanabe Y ichi, Sugahara J, et al. Genomic analysis of the uncultivated marine crenarchaeote *Cenarchaeum symbiosum*. Proc Natl Acad Sci U S A. 2006;103(48):18296–301.

12. Walker CB, de la Torre JR, Klotz MG, Urakawa H, Pinel N, Arp DJ, et al. *Nitrosopumilus maritimus* genome reveals unique mechanisms for nitrification and autotrophy in globally distributed marine crenarchaea. Proc Natl Acad Sci U S A. 2010;107(19):8818–23.

13. Park SJ, Kim JG, Jung MY, Kim SJ, Cha IT, Kwon K, et al. Draft genome sequence of an ammonia-oxidizing archaeon, "*Candidatus Nitrosopumilus koreensis*" AR1, from marine sediment. J Bacteriol. 2012;194(24):6940–1.

14. Blainey PC, Mosier AC, Potanina A, Francis CA, Quake SR. Genome of a low-salinity ammonia-oxidizing archaeon determined by single-cell and metagenomic analysis. PLoS One. 2011;6(2):e16626.

15. Kim BK, Jung MY, Yu DS, Park SJ, Oh TK, Rhee SK, et al. Genome sequence of an ammonia-oxidizing soil archaeon, "*Candidatus Nitrosoarchaeum koreensis*" MY1. J Bacteriol. 2011;193(19):5539–40.

16. Doxey AC, Kurtz DA, Lynch MDJ, Sauder LA, Neufeld JD. Aquatic metagenomes implicate Thaumarchaeota in global cobalamin production. ISME J. 2015;9(2):461–71.
