# Supplementary material for: The compact genome of the sponge Oopsacas minuta (Hexactinellida) is lacking key metazoan core genes
Source: BMC Biol. 2023 Jun 19;21:139. doi: 10.1186/s12915-023-01619-w (PMC10280926; doi:10.1186/s12915-023-01619-w)
Supplement: Supplementary file 2 — Additional file 2: Genomic characteristics of Oopascas minuta. Table S5. Genome features of the glass sponge O. minuta. Table S6. Respective number of predicted and annotated sequences in the genome of O. minuta. Figure S5. Gene ontology at the first two levels for the 7,737 genes that received GO annotations. Figure S6. a) Orthology clustering on predicted proteomes of six sponges. b) Gene Ontology at level3 for orthogroups absent in O. minuta and present in all other sponge lineages. Table S7. ORFING report, Comparison of protein sequence lengths and BUSCO scores between the different versions of the genome masked or unmasked. Table S8. Types and relative abundances of repeats in the genome of O. minuta. [file 12915_2023_1619_MOESM2_ESM.pdf]

<u>Additional File 2 Index</u>

Tables S5 : *Genome features of the glass sponge O. minuta, compared to other sponges from the three other classes*

Table S6 : *Respective number of predicted and annotated sequences in the genome of O. minuta*

Figure S5: *Gene ontology at the first two levels for the 7,737 genes that received GO annotations*

Figures S6 : *a) Orthology clustering on predicted proteomes of six sponges. b) Gene Ontology at level 3 for orthogroups absent in O. minuta and present in all other sponge lineages*

Table S7: *ORFING report, Comparison of protein sequence lengths and BUSCO scores between the different versions of the genome masked or unmasked*

Table S8: *Types and relative abundances of repeats in the genome of* O. minuta

<u>Table S5a:</u> Genome features of the glass sponge *O. minuta*, compared to other sponges from the three other classes. Table modified from Kenny et al., 2020 by adding Busco (Simao et al 2015) results (BUSCO v2/v3) for *O. minuta* using the Eukaryota OrthoDB v9 core genes set and computed on the gVolante server.

	Hexactinellida		Demospongiae				Homoscleromorpha	Calcarea
species	*O. minuta* (genome mode)	*O. minuta* (Augustus aa set)	*E. muelleri* (genome mode)	*E. muelleri* (Augustus aa set)	*A. queenslandica*	*T. wilhelma*	*O. pearsei*	*S. ciliatum*
**Total # of core genes queried**	**303**	**303**	303	303	303	303	303	303
**# of core genes detected**	**282 (93.07%)**	**278 (91.75%)**	243 (80.20%)	254 (83.83%)	274 (90.43%)	258 (85.15%)	207 (68.32%)	240 (79.21%)
**Complete + Partial**	**285 (94.06%)**	**284 (93.73%)**	254 (83.83%)	273 (90.10%)	281 (92.74%)	275 (90.76%)	237 (78.22%)	259 (85.48%)
**# of missing core genes**	**18 (5.94%)**	**19 (6.27%)**	49 (16.17%)	30 (9.90%)	22 (7.26%)	28 (9.24%)	66 (21.78%)	44 (14.52%)
**Average # of orthologues per core genes**	**1.01**	**1.01**	1.12	1.22	1.04	1.05	1	1.07
**% of detected core genes that have more than 1 ortholog**	**1.06**	**0.72**	10.7	19.6	4.38	4.26	0	6.67

<u>Table S5b:</u> Mean size of coding and non-coding sequences in *O. minuta* compared to two demosponges
Statistics are from the current *O. minuta* genome and two Demospongiae genomes used for Busco computation (genome soruce can be found in the main text).

	Hexactinellida	Demospongiae	
	Oopsacas minuta	*Ephydatia muelleri*	*Amphimedon queenslandica (V2.1)*
**Number of predicted proteins**	**16413**	39245	43615
**Mean protein length (aa residues)**	**425.2**	497.66	337.84
**Maximum protein length (aa residues)**	**13808**	37921	18893
**Minimum protein length (aa residues)**	**43**	10	5
**Mean number of introns per gene**	**1.59**	3.009	2.01
**Mean intron size (bp)**	**341.2**	361.49	327.62
**Median intron size (bp)**	**139.5**	138	70
**Mean gene size (bp)**	**1806**	4507.53	2425.68
**Mean inter-gene distance (bp)**	**2001**	3524.79	2145.72

<u>Table S6:</u> Respective number of predicted and annotated sequences in the genome of *O. minuta*

See methods for any details on the gene prediction.

Categories		Number
**predicted genes**		16413
**Annotated Genes**		7737
**GO Terms**	Biological	4092
	Cellular	2042
	Function	6704
	**Total**	12838

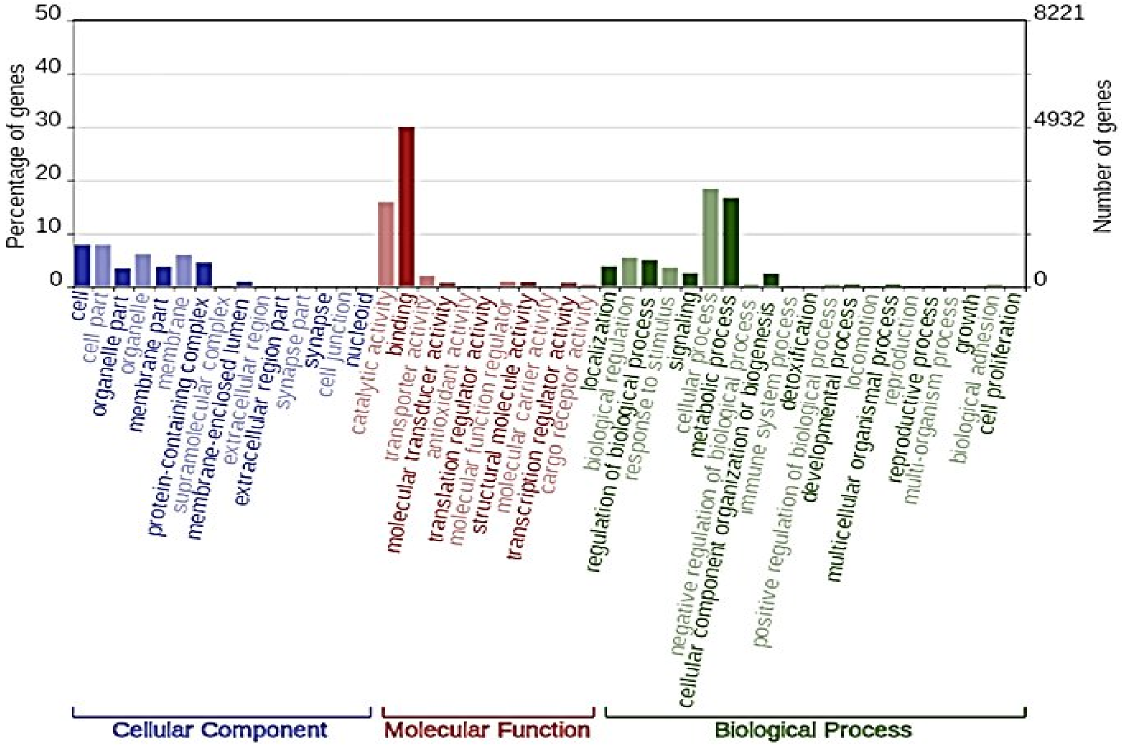

<u>Figure S5:</u> Gene ontology at the first two levels for the 7,737 genes that received GO annotations.

GO infered via a domain search using InterProScan (Quevillon et al 2005) against the conserved domain database at NCBI.

GO identifiers were used on WEGO (http://wego.genomics.org.cn/).

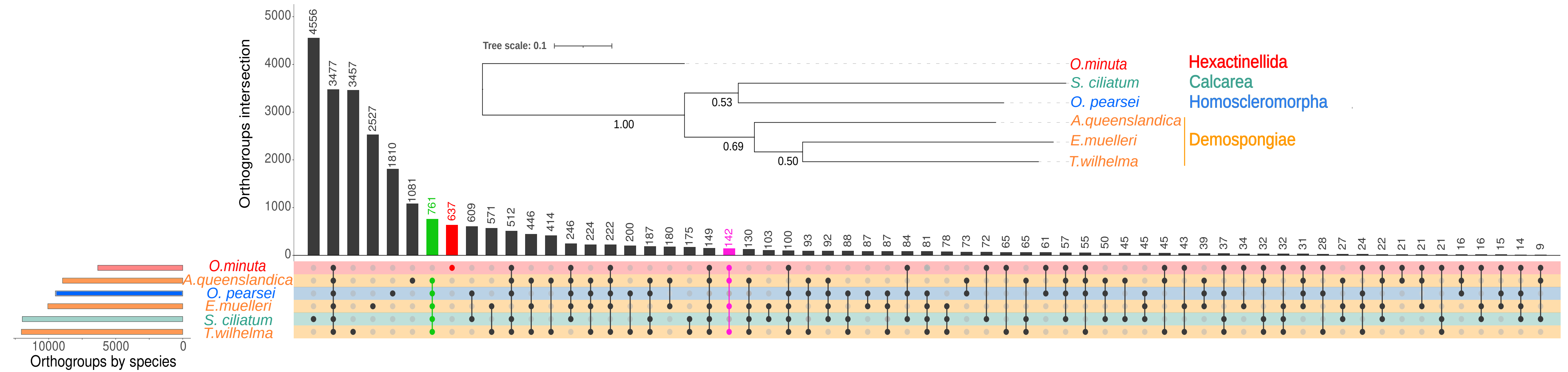

<u>Figure S6a</u>: Orthology clustering on predicted proteomes of six sponges pertaining to the four sponge classes.

Orthogroups were computed using Orthofinder (Emms & Kelly 2019). The figure was generated with the R module UpSetR (https://CRAN.R-project.org/package=UpSetR) on the basis of the Orthogroups.GeneCount.tsv simplified to take into account only the presence/absence of a species in an orthogroup and iTOL (Letunic & Bork 2021) based on the SpeciesTree_rooted.txt output files from Orthofinder.

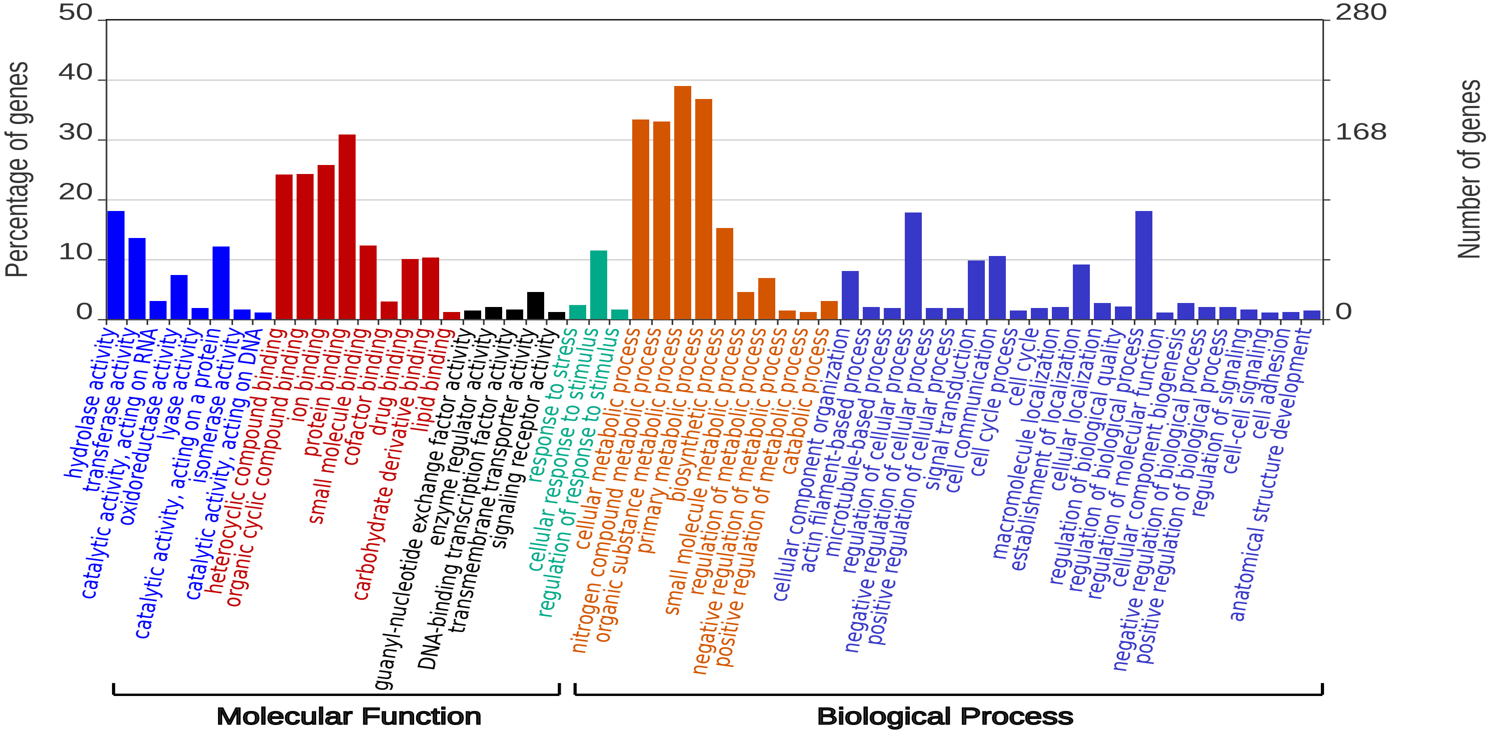

<u>Figure S6b</u>: Gene Ontology at level 3 for orthogroups absent in *O. minuta* and present in all other sponge lineages (if >1%)

GO infered via a domain search using InterProScan (Quevillon et al 2005) against the conserved domain database at NCBI. GO identifiers were used on WEGO (http://wego.genomics.org.cn/) Main functional categories are represented by different colors to make visualization easier. .

Table S7: ORFING report, Comparison of protein sequence lengths and BUSCO scores between the different versions of the genome masked or unmasked.

dataset	#sequences	Longest	Shortest	Mean	Median	Complete Busco	Partial Busco	Busco Score
**GCA_024704765.1_ASM2470476v1***	**16413**	**13808**	**43**	**425**	**301**	**278**	**284**	**C:91.8%[S:90.8%,D:1.0%],F:2.0%,M:6.2%,n:303**
uncleaned unmasked	17059	13808	38	429	303	278	284	C:91.7%[S:89.4%,D:2.3%],F:2.0%,M:6.3%,n:303
uncleaned hardmasked no low	11932	15060	38	502	360	277	282	C:91.4%[S:89.1%,D:2.3%],F:1.7%,M:6.9%,n:303
uncleaned hardmasked	11600	12346	39	510	365	273	281	C:90.1%[S:87.5%,D:2.6%],F:2.6%,M:7.3%,n:303
uncleaned softmasked no low	16668	15597	38	436	311	278	285	C:91.7%[S:88.1%,D:3.6%],F:2.3%,M:6.0%,n:303
uncleaned softmasked	16371	15257	38	442	316	278	284	C:91.8%[S:88.8%,D:3.0%],F:2.0%,M:6.2%,n:303

C: complete
S: complete and single copy
D: complete and duplicated
F: fragmented
M: missing
n: total search

*** version analysed in this work**

<u>Table S8 :</u> Types and relative abundances of repeats predicted in the genome of *O. minuta*. Repeats were predicted and analysed with RepeatModeler 2.0.4 and RepeatMasker 4.1.4 (default mode) (http://www.repeatmasker.org) from the Dfam TEtool container https://github.com/Dfam-consortium/TETools
.

	number of elements*	length occupied	percentage of sequence
Retroelements	967	402918 bp	0.66 %
SINEs:	0	0 bp	0.00 %
Penelope	0	0 bp	0.00 %
LINEs:	0	0 bp	0.00 %
CRE/SLACS	0	0 bp	0.00 %
L2/CR1/Rex	0	0 bp	0.00 %
R1/LOA/Jockey	0	0 bp	0.00 %
R2/R4/NeSL	0	0 bp	0.00 %
RTE/Bov-B	0	0 bp	0.00 %
L1/CIN4	0	0 bp	0.00 %
LTR elements:	967	402918 bp	0.66 %
BEL/Pao	0	0 bp	0.00 %
Ty1/Copia	576	85338 bp	0.14 %
Gypsy/DIRS1	391	317580 bp	0.52 %
Retroviral	0	0 bp	0.00 %
DNA transposons	16375	9486879 bp	15.44 %
hobo-Activator	3729	1655397 bp	2.69 %
Tc1-IS630-Pogo	5265	3313939 bp	5.39 %
En-Spm	0	0 bp	0.00 %
MULE-MuDR	1296	572590 bp	0.93 %
PiggyBac	0	0 bp	0.00 %
Tourist/Harbinger	171	170952 bp	0.28 %
Other (Mirage, P-element, Transib)	1552	748427 bp	1.22 %
Rolling-circles	148	83011 bp	0.14 %
Unclassified:	64870	11067513 bp	18.01 %
Total interspersed repeats:		20957310 bp	34.10 %
Small RNA:	0	0 bp	0.00 %
Satellites:	0	0 bp	0.00 %
Simple repeats:	7816	364942 bp	0.59 %
Low complexity:	910	45186 bp	0.07 %

* most repeats fragmented by insertions or deletions have been counted as one element

1. Kenny NJ, Francis WR, Rivera-Vicéns RE, Juravel K, de Mendoza A, Díez-Vives C, et al. Tracing animal genomic evolution with the chromosomal-level assembly of the freshwater sponge *Ephydatia muelleri*. Nat Commun. 2020;11(1):3676.

2. Simão FA, Waterhouse RM, Ioannidis P, Kriventseva EV, Zdobnov EM. BUSCO: assessing genome assembly and annotation completeness with single-copy orthologs. Bioinformatics. 2015;31(19):3210–2.

3. Quevillon E, Silventoinen V, Pillai S, Harte N, Mulder N, Apweiler R, et al. InterProScan: protein domains identifier. Nucleic Acids Res. 2005;33:W116-120.

4. Emms DM, Kelly S. OrthoFinder: phylogenetic orthology inference for comparative genomics. Genome Biology. 2019;20(1):238.

5. Letunic I, Bork P. Interactive Tree Of Life (iTOL) v5: an online tool for phylogenetic tree display and annotation. Nucleic Acids Research. 2021;49(W1):W293–6.
