# Supplementary material for: The compact genome of the sponge Oopsacas minuta (Hexactinellida) is lacking key metazoan core genes
Source: BMC Biol. 2023 Jun 19;21:139. doi: 10.1186/s12915-023-01619-w (PMC10280926; doi:10.1186/s12915-023-01619-w)
Supplement: Supplementary file 3 — Additional file 3: Survey of candidate genes for epithelia, multicilliogenesis, signaling pathways, transcription factors, neuro-sensory functions, biosilicification. Table S9. Blastp search of proteins involved in bilaterian epithelial functions. Figure S7. Domain prediction of integrins and of laminins. Table S10. Blastp search concerning proteins involved in bilaterian multiciliogenesis. Table S11. Blast Search of core genes involved in three main ancestral signalling pathways. Table S12. Blastp search results concerning G-proteins. Table S13. Blastp search and protein domain analyses concerning basic Helix Loop Helixtranscription factors. Figure S8. Phylogenetic relationships among bHLH Transcription factors. Figure S9. Phylogenetic position of Oopsacas bHLH-PAS Transcription factors. Table S14. Blastp search, domain and phylogenetic analyses performed on various transcription factor types. Figure S10. Domain Prediction of HD transcription factors. Figure S11. Phylogenetic positions of Oopsacas Homeobox Transcription factors of the ANTP class. Table S15. Survey of candidate genes for photoreception. Table S16. Blastp search for proteins involved in chemical signaling. Table S17. HHMER searches in O. minuta for voltage gated ion channels. Figure S12. Diversity and phylogenetic positions of Oopsacas calcium channels. Figure S13. Phylogenetic positions of Oopsacas potassium channels. Table S18. Blastp search of proteins involved in bilaterian synapses. Table S19. Results of HMMER searches for SNARES proteins. Table S20. Blast P search of proteins involved in silica biogenesis. Table S21. List of sequences used for phylogenetic analyses of silicatein and cathepsin. [file 12915_2023_1619_MOESM3_ESM.pdf]

Additional File 3 Index

Table S9 *: Blastp search of proteins involved in bilaterian epithelial functions.*

Figures S7 *: Domain prediction of integrins and of laminins*

Table S10 : *Blastp search concerning proteins involved in bilaterian multiciliogenesis.*

Table S11 **:** *Blast Search of core genes involved in three main ancestral signalling pathways.*

Table S12 **:** *Blastp search results concerning G-proteins.*

Table S13 *: Blastp search and protein domain analyses concerning basic Helix Loop Helix (bHLH) transcription factors*

Figure S8 *: Phylogenetic position of* Oopsacas *bHLH TF*

Figure S9 *: Phylogenetic position of* Oopsacas *bHLH-PAS TF*

Table S14 *: Summary of blast search results concerning homeobox transcription factors*

Figure S10 *: Prediction of protein domains of HD transcription factors*

Figure S11 *: Phylogenetic position of* Oopsacas *HD TF of the ANTP class*

Table S15 : *Survey of candidate genes for photoreception*

Table S16 : *Blast searches for proteins involved in chemical signaling*

Table S17 : *HHMER search results for voltage gated ion channels*

Figure S12 *: Phylogenetic positions of* Oopsacas *calcium channels*

Figure S13 : *Phylogenetic positions of* Oopsacas *potassium channels*

Table S18 *: Blastp search of genes involved in bilaterian synapses*

Table S19 : *HMMER search of SNARES*

Table S20 *: Blastp search of proteins involved in silica biogenesis*

Table S21 : *List of sequences used for phylogenetic analyses of silicatein and cathepsin*

Table S9: Blast P search of proteins involved in bilaterian epithelial functions (only genes with highly significant hits are indicated)

Query		Blast P vs. oopsacas proteome	reverse blast-p result against NR, best hit providing an annotated protein					
searched protein	query sequence (species/accession number)	*O.minuta* locus tag	description	score	cover	e-value	identity	accession number
Contactin	*Contactin (Aphrocalistes vastus) / Ava_Contig_16553-1*	**LOD99_12497**	*Contactin-2 [Mugil cephalus]*	186	95%	2.00E-44	24.62%	XP_047440236.1 (NCBI)
		**LOD99_4945**	*Protein sidekick-1 isoform X3 [Danio rerio]*	234	92%	3.00E-58	24.56%	XP_017211455.1 (NCBI)
		**LOD99_2903**	*Neural cell adhesion molecule L1-like [Lytechinus variegatus]*	286	62%	4.00E-74	27.87%	XP_041470985.1 (NCBI)
		**LOD99_13407**	*Dscam2 isoform X1 [Bombyx mori]*	234	61%	3.00E-58	25.77%	XP_012543882.3 (NCBI)
Integrin	*Integrin alpha (Aphrocalistes vastus) / Ava_Contig_11100-1_and 11100-2*	**LOD99_15309**	*Integrin alpha-9-like [Amphimedon queenslandica]*	347	99%	3.00E-98	29.16%	XP_019864024.1 (NCBI)
		**LOD99_15308**	*Integrin alpha-9-like [Amphimedon queenslandica]*	344	98%	4.00E-97	28.32%	XP_019864024.1 (NCBI)
		**LOD99_13917**	*Integrin alpha-V [Xiphophorus couchianus]*	249	99%	2.00E-64	26.38%	XP_027878087.1 (NCBI)
		**LOD99_9146**	*Integrin alpha-PS2-like [Limulus polyphemus]*	283	96%	8.00E-76	27.46%	XP_022250303.1(NCBI)
		**LOD99_6533**	*Integrin alpha-8-like [Scyliorhinus canicula]*	178	95%	3.00E-41	23.96%	XP_038635355.1 (NCBI)
	Integrin beta (Aphrocalistes vastus) / Ava_Contig_13475-2_integrin-beta-1	**LOD99_8099**	*Integrin beta-7 [Microcaecilia unicolor]*	447	95%	1.00E-141	34.29%	XP_030054041.1 (NCBI)
		**LOD99_2393**	*integrin beta-C subunit precursor [Strongylocentrotus purpuratus]*	322	98%	7.00E-94	29.26%	NP_999730.1 (NCBI)
		**LOD99_12882**	*ITB2 protein [Penelope pileata]*	230	91%	3.00E-63	32.11%	NXC50956.1 (NCBI)
Nidogen	*Nidogen (A. vastus) / Ava_Contig_19310_nidogen*	**LOD99_13205**	*Protogenin-like protein [Leptotrombidium deliense]*	80.5	54%	2.00E-11	27.09%	RWS24579.1 (NCBI)
		**LOD99_13214**	*receptor-type tyrosine-protein phosphatase epsilon-like [Amphimedon queenslandicc*	275	13%	3.00E-74	47.94%	XP_011404097.2
Laminin	*Laminin beta (A. vastus) / Ava_Contig_18402_Laminin-beta*	**LOD99_7300**	*laminin subunit gamma-1-like [Amphimedon queenslandica]*	779	98%	0.0	30.45%	XP_019849782.1 (NCBI)
		**LOD99_12851**	*Laminin subunit gamma-1-like [Acropora millepora]*	807	98%	0.0	29.12%	XP_044175181.1(NCBI)
		**LOD99_7636**	*Laminin-like protein epi-1 [Acropora millepora]*	892	77%	0.0	28.06%	XP_044182513.1 (NCBI)
		**LOD99_1235**	*Laminin subunit beta-1-like [Dermatophagoides farinae]*	281	47%	4.00E-72	33.89%	XP_046909188.1 (NCBI)
		**LOD99_8477**	*Usherin-like [Amphimedon queenslandica]*	1246	98%	0.0	35.50%	XP_011403870.2 (NCBI)

present

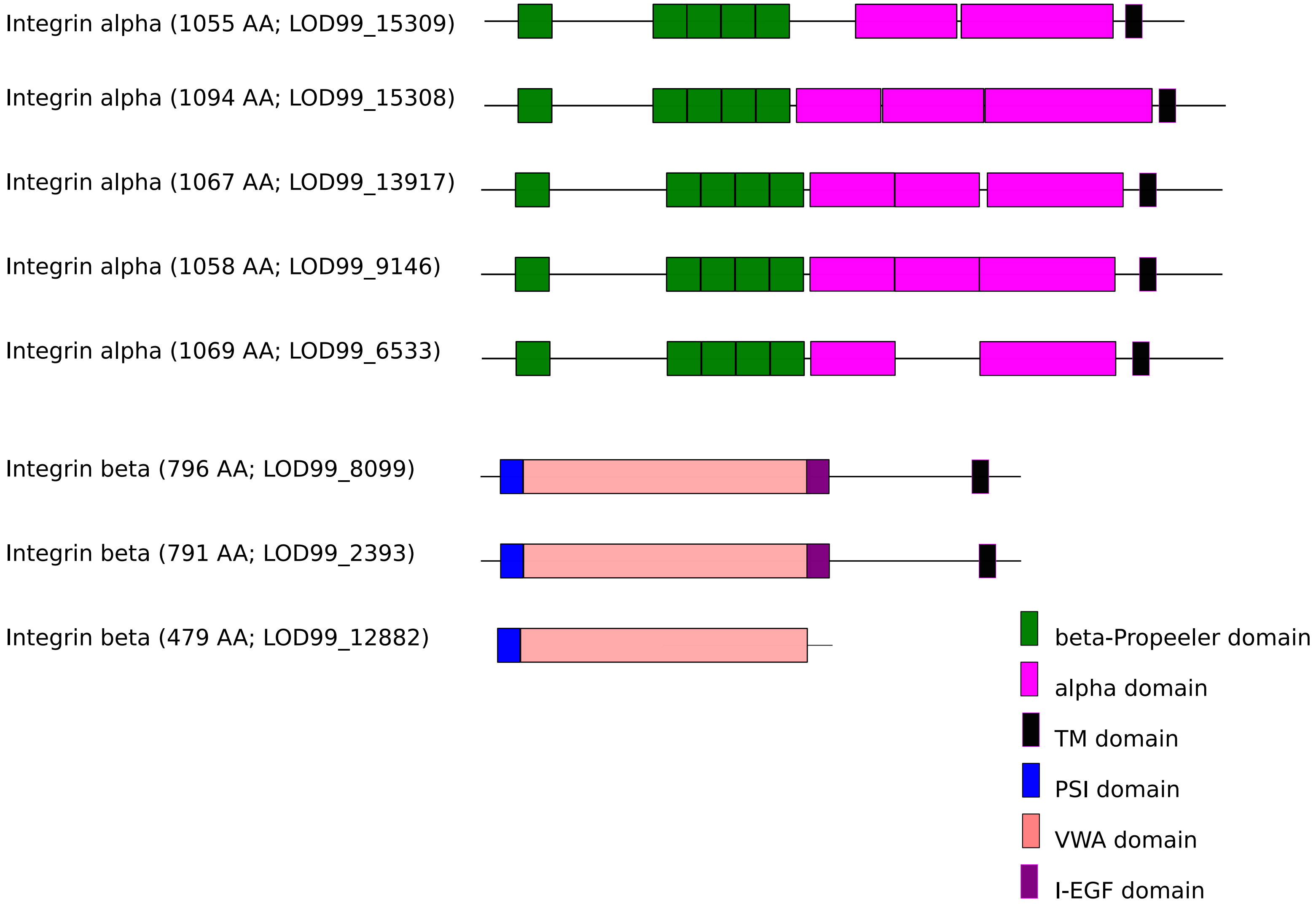

<u>Figure S7a</u>: Domain prediction of *Oopsacas* integrins using Pfam.
5 predicted alpha-integrins of *O. minuta* have a domain structure similar to those observed in other metazoans: a globular head region in the extracellular domain consisting in five or six repeats of about 60 amino acids that fold into a five/six-bladed β propeller followed by a large integrin alpha-2 domain. The 3 proteins predicted as beta-integrins share an integrin plexin domain (PSI) in the Nt globular part, an integrin beta chain VWA domain, an integrin beta epidermal growth factor like domain 1 as reported in other metazoans.

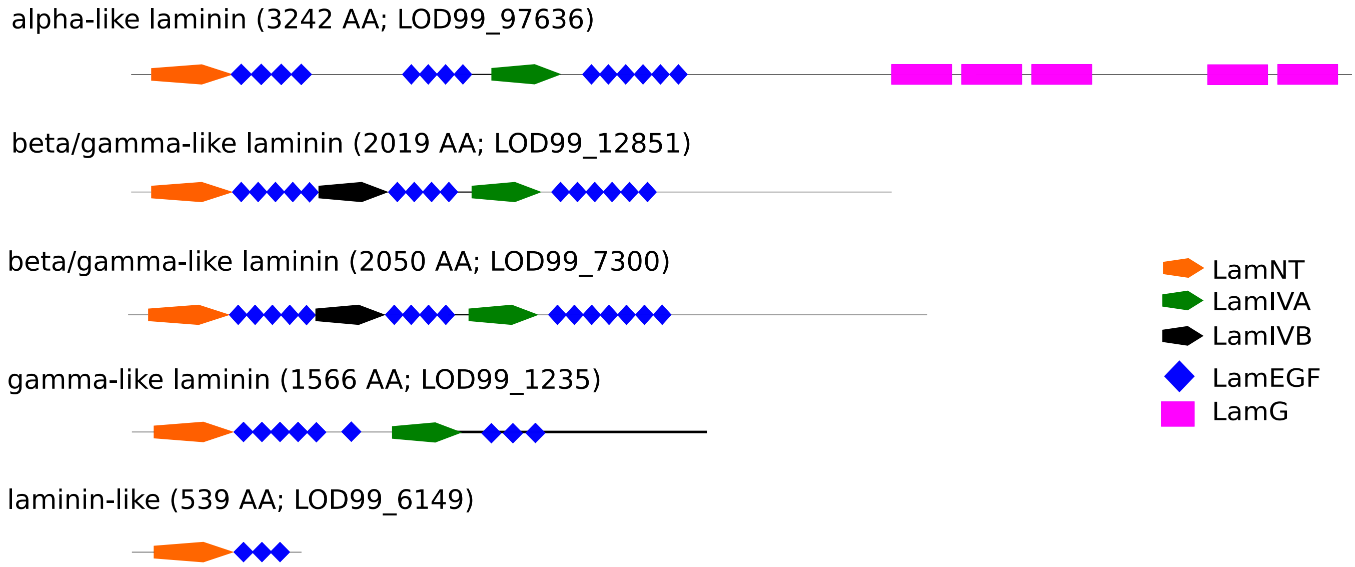

<u>Figure S7</u>b: Protein Domain prediction of *Oopsacas* laminins using Pfam

<u>Table S10:</u> Blastp search concerning proteins involved in bilaterian multiciliogenesis (only locus tags of sequences with e-value<10-2 are provided; reverse best blast hits were performed against NCBI NR database)

searched protein	query sequence (species/accession number)	*O.minuta* locus tag	reverse blast-p result against NR, best hit providing an annotated protein					
			description	score	cover	e-value	identity	accession number
DEUP 1	*Xenopus tropicalis*/XP_002940187.1	-						
CEP63	*Xenopus laevis*/JAG34037.1	-						
	Amphimedon queenslandica/XP_019856539.1	-						
multicilin	*Xenopus laevis*/ NP_001121276.1	-						
pericentrin	*Xenopus tropicalis*/ XP_017952870.2	-						
E2F4	*Xenopus laevis*/NP_001086706.1	LOD99_7024	transcription factor E2F3 [Cyclopterus lumpus]	87.8	45%	3.00E-15	32.46%	XP_034401885.1
		LOD99_13394	E2F_TDP-domain-containing protein [Fragilariopsis cylindrus CCMP1102]	76.6	58%	5.00E-12	24.63%	OEU15276.1
FOXJ1	*Xenopus laevis*/NP_001083644.1	LOD99_9296	Fork head domain [Trinorchestia longiramus]	166	36%	2.00E-48	69.23%	KAF2348946.1
	Amphimedon queenslandica/Larroux et al.2008	LOD99_4349	forkhead foxL2 [Suberites domuncula]	199	34%	6.00E-58	74.80%	CAE51212.1
		LOD99_3851	forkhead box protein J3-like isoform X2 [Limulus polyphemus]	125	87%	4.00E-29	29.43%	XP_013784418.2
		LOD99_5974	PREDICTED: forkhead box protein L2-like [Amphimedon queenslandica]	180	46%	7.00E-50	58.06%	XP_019858441.1
		LOD99_2423	Forkhead box protein P1 [Habropoda laboriosa]	187	46%	4.00E-46	37.60%	KOC66408.1
		LOD99_6831	Forkhead box protein O [Exaiptasia diaphana]	154	22%	5.00E-38	51.28%	KXJ17066.1
RX2/3	*Xenopus laevis*/NP_001090132.1	LOD99_9386	RFX2 protein [Urocynchramus pylzowi]	306	81%	5.00E-96	40.84%	NWT93764.1
		LOD99_6086	transcription factor RFX4 [Petromyzon marinus]	402	52%	8.00E-124	45.65%	XP_032805092.1
c-Myb	*Xenopus laevis*/AAH70808.1	-						
	Hydra vulgaris/QFU95890.1	-						
	Trichoplax sp. H2/RDD46708.1	-						
CCNO	*Xenopus tropicalis*/NP_998837.1	LOD99_13910B	PREDICTED: G2/mitotic-specific cyclin-B3-like [Amphimedon queenslandica]	244	49%	3.00E-71	45.25%	XP_019850667.1
		LOD99_13910A	PREDICTED: G2/mitotic-specific cyclin-B3-like [Amphimedon queenslandica]	244	52%	8.00E-72	45.25%	XP_019850667.1
		LOD99_11433	G2/mitotic-specific cyclin-B-like [Anneissia japonica]	296	90%	5.00E-94	49.12%	XP_033121875.1
		LOD99_11524	G2/mitotic-specific cyclin-A-like [Actinia tenebrosa]	280	40%	5.00E-84	55.25%	XP_031561724.1
		LOD99_2502	G2/mitotic-specific cyclin-B3 isoform X1 [Vombatus ursinus]	185	80%	1.00E-52	38.58%	XP_027693373.1
		LOD99_12806	G2/mitotic-specific cyclin-B-like isoform X1 [Actinia tenebrosa]	144	74%	8.00E-36	34.62%	XP_031565307.1
		LOD99_12212	CYCB1-1 protein [Petunia x hybrida]	244	80%	5.00E-28	30.87%	CAB58998.1
		LOD99_2433	PREDICTED: cyclin-J-like [Branchiostoma belcheri]	190	66%	2.00E-54	39.67%	XP_019625841.1
CEP152	*Xenopus laevis*/NP_001087086.1	-						
CEP 192	*Xenopus laevis*/NP_001186000.1	LOD99_3211	centrosomal protein of 192 kDa-like isoform X2 [Actinia tenebrosa]	235	65%	4.00E-58	23.05%	XP_031572133.1
CEP 57	*Xenopus tropicalis*/XP_002935622.1	LOD99_3713A	PREDICTED: centrosomal protein of 57 kDa-like isoform X16 [Branchiostoma belcheri]	147	83%	2.00E-34	27.54%	XP_019629318.1
		LOD99_3713B	PREDICTED: centrosomal protein of 57 kDa-like isoform X16 [Branchiostoma belcheri]	137	83%	9.00E-31	26.65%	XP_019629318.1
CEP 57/1	*Xenopus laevis*/NP_001089236.1	-						
PLK4	*Xenopus laevis*/NP_001083146.1	LOD99_2334	serine/threonine protein kinase [Culex quinquefasciatus]	308	100%	3.00E-92	36.55%	XP_001845832.1
			PREDICTED: serine/threonine-protein kinase PLK4 [Neodiprion lecontei]	306	99%	2.00E-91	35.44%	XP_015521281.1
			serine/threonine-protein kinase PLK4 isoform X1 [Nematostella vectensis]	308	63%	9.00E-91	44.76%	XP_001626807.1
SAS6	*Xenopus tropicalis*/XP_012819859.2	LOD99_1925	Chain A, Serine/threonine-protein kinase PLK4 [Homo sapiens]	286	50%	1.00E-89	50.37%	3COK_A
STIL	*Xenopus laevis*/NP_001084821.1	LOD99_9458	SCL-interrupting locus protein-like [Orbicella faveolata]	179	42%	2.00E-43	32.00%	XP_020624688.1
CENPJ	*Xenopus tropicalis*/XP_031753135.1	LOD99_7234	centromere protein J-like isoform X2 [Lingula anatina]	206	55%	2.00E-51	29.77%	XP_013399795.1

- (grey)	no reliable hit found (e-value>1.10-2)
(green)	retrieved in *O. minuta* genome with good support
(orange)	presence of members of the same family

Table S11: Blast Search of core genes involved in three main ancestral signalling pathways. A blastP search against the predicted proteome of Oopsacas was performed followed by a reciprocal best hit approach against NCBI NR database for blast hits with e-values <10^{-2}. Genes absent in the transcriptome of Oopsacas (Schenkelaars et al., 2017) are also absent in the genome. (Domain predictions supporting the annotation are available in Schenkelaars et al., 2017)

	Query		Blast P against oopsacas proteome	reverse blast-p result against NR, best hit providing an annotated protein					
	searched protein	query sequence (species/accession number)	*O.minuta* locus tag	description	score	cover	e-value	identity	accession number
Wnt pathway	APC	*Oopsacas minuta* / APZ80421.1 (NCBI)	**LOD99_7009**	*Oopsacas minuta APC*	1707	100%	0.0	100.00%	APZ80421.1 (NCBI)
	Axin	*Oopsacas minuta* / KX905244.1 (NCBI)	**LOD99_7340**	*Oopsacas minuta Axin*	1311	100%	0.0	99.53%	APZ81092.1 (NCBI)
	β-catenin	*Oopsacas minuta* / KU316199.1 *(NCBI)*	**LOD99_2816**	*Oopsacas minuta beta-catenin*	1787	100%	0.0	100.00%	APZ80422.1 (NCBI)
	CK1	*Mus musculus* / Q8BK63 (Uniprot)	**LOD99_3585**	*Oncorhynchus kisutch casein kinase I*	624	86%	0.0	90.18%	XM_031789862.1
	Dvl	*Mus musculus* / P51141 (Uniprot)	-						
		Amphimedon queenslandica / ADO16582.1	-						
		Oscarella lobularis / KT898949.1 (NCBI)	-						
	FzdB	*Oopsacas minuta* / KM365035.1 (NCBI)	**LOD99_13467**	*Oopsacas minuta Frizzled B*	1120	95%	0.0	100.00%	AJE25510.1 (NCBI)
	GSK3	*Oopsacas minuta* / KU316201.1 (NCBI)	**LOD99_2576**	*Oopsacas minuta Gsk3*	890	100%	0.0	100.00%	APZ80424.1 (NCBI)
	Gro	*Oopsacas minuta* / KU316200.1 (NCBI)	**LOD99_13544**	*Oopsacas minuta Gro*	1486	100%	0.0	100.00%	APZ80423.1 (NCBI)
	LRP	*Oopsacas minuta* / KU316202.1 (NCBI)	**LOD99_15634**	*Oopsacas minuta LRP*	3394	100%	0.0	100.00%	APZ80425.1 (NCBI)
	Porc	*Oopsacas minuta* / KU316203.1 (NCBI)	**LOD99_12796**	*Oopsacas minuta Porc*	880	100%	0.0	100.00%	APZ80426.1 (NCBI)
	TCF	*Oopsacas minuta* / KU316204.1 (NCBI)	**LOD99_8961**	*Oopsacas minuta TCF*	1015	100%	0.0	100.00%	APZ80427.1 (NCBI)
	Wls	*Mus musculus* / Q6DID7 (Uniprot)	-						
		Oscarella lobularis / (personal data)	-						
	Wnt	*Mus musculus* / P04426 (Uniprot)	-						
		Amphimedon queenslandica / ABX90060.1 (NCBI)	-						
		Oscarella lobularis / GQ144646.1 (NCBI)	-						
Notch pathway	ADAM10	*Oopsacas minuta* / MF589734 (NCBI)	**LOD99_118**	*Oopsacas minuta Adam 10*	1355	96%	0.0	100.00%	ATY70071.1 (NCBI)
	ADAM17	*Oopsacas minuta* / MF589735 (NCBI)	**LOD99_13145**	*Oopsacas minuta Adam17*	1686	100%	0.0	99.51%	ATY70072.1 (NCBI)
	APH1	*Oopsacas minuta* / MF589739 (NCBI)	**LOD99_3257**	*Oopsacas minuta APH1*	488	100%	1.00E-173	100.00%	ATY70076.1 (NCBI)
	Delta	*Oopsacas minuta* / KX932092 (NCBI)	**LOD99_19**	*Oopsacas minuta Delta*	2072	100%	0.0	100.00%	APZ81095.1 (NCBI)
	FurinL	*Oopsacas minuta* / MF589732 (NCBI)	**LOD99_4168**	*Oopsacas minuta FurinL*	1969	100%	0.0	100.00%	ATY70069.1 (NCBI)
	MamL	*Mus musculus* / Q92585 (Uniprot)	-						
	NCoRL	*Oopsacas minuta* / MF589741 (NCBI)	**LOD99_6013**	*Oopsacas minuta NCoRL*	1217	94%	0.0	97.24%	ATY70078.1 (NCBI)
	NCSTN	*Oopsacas minuta* / MF589736 (NCBI)	**LOD99_14978**	*Oopsacas minuta NCSTN*	1348	100%	0.0	99.39%	ATY70073.1 (NCBI)
	Notch	*Oopsacas minuta* / MF589733 (NCBI)	**LOD99_2688**	*Oopsacas minuta Notch*	3626	97%	0.0	92.32%	APZ81096.1 (NCBI)
	PEN2	*Oopsacas minuta* / MF589738 (NCBI)	**LOD99_7221**	*Oopsacas minuta Pen2*	206	100%	7.00E-67	100.00%	ATY70075.1 (NCBI)
	PSEN2	*Oopsacas minuta* / MF589737 (NCBI)	**LOD99_11660**	*Oopsacas minuta Psen*	885	100%	0.0	100.00%	ATY70074.1 (NCBI)
	SuH	*Oopsacas minuta* / MF589740 (NCBI)	**LOD99_6806**	*Oopsacas minuta SuH*	984	73%	0.0	95.07%	ATY70077.1 (NCBI)
TGF pathway	ACVRI	*Oopsacas minuta* / MF589727 (NCBI)	**LOD99_12520**	*Oopsacas minuta ACVRI*	793	73%	0.0	99.74%	ATY70064.1 (NCBI)
	ACVRII	*Oopsacas minuta* / MF589725 (NCBI)	**LOD99_13135**	*Oopsacas minuta ACVRII*	1264	95%	0.0	96.26%	ATY70062.1 (NCBI)
	BMPLa	*Oopsacas minuta* / MF589722 (NCBI)	**LOD99_11279**	*Oopsacas minuta BMPLa*	702	100%	0.0	99.71%	ATY70059.1 (NCBI)
	BMPLb	*Oopsacas minuta* / MF589723 (NCBI)	**LOD99_4658**	*Oopsacas minuta BMPLb*	893	100%	0.0	99.77%	ATY70060.1 (NCBI)
	BMPLc	*Oopsacas minuta* / MF589724 (NCBI)	**LOD99_13456**	*Oopsacas minuta BMPLc*	694	86%	0.0	100.00%	ATY70061.1 (NCBI)
	SARA	*Mus musculus* / A8Y5G5 (Uniprot)	-						
	Ski/Sn	*Mus musculus* / A7M7C7 (Uniprot)	-						
	Smad1/5	*Oopsacas minuta* / MF589729 (NCBI)	**LOD99_4111**	*Oopsacas minuta Smad1/5*	788	81%	0.0	100.00%	ATY70066.1 (NCBI)
	Smad2/3	*Oopsacas minuta* / MF589730 (NCBI)	**LOD99_13626**	*Oopsacas minuta Smad2/3*	969	100%	0.0	98.11%	ATY70067.1 (NCBI)
	Smad4	*Oopsacas minuta* / MF589731 (NCBI)	**LOD99_15194**	*Oopsacas minuta Smad 4*	1189	98%	0.0	99.83%	ATY70068.1 (NCBI)
	Smad6/7	*Mus musculus* / O35182 (Uniprot)	-						
	SMURF	*Mus musculus* / Q9CUN6 (Uniprot)	-						
	TGFBLa	*Oopsacas minuta* / MF589721 (NCBI)	**LOD99_185**	*Oopsacas minuta TGFbetaLa*	791	100%	0.0	99.48%	ATY70058.1 (NCBI)
	TGFBRIa	*Oopsacas minuta* / KX932095.1 (NCBI)	**LOD99_14512**	*Oopsacas minuta TGFbetaRIa*	1071	99%	0.0	100.00%	APZ81098.1 (NCBI)
	TGFBRIb	*Oopsacas minuta* / MF589728 (NCBI)	**LOD99_731**	*Oopsacas minuta TGFbetaRIb*	1006	91%	0.0	100.00%	ATY70065.1 (NCBI)
	TGFBRII	*Oopsacas minuta* / MF589726 (NCBI)	**LOD99_636**	*Oopsacas minuta TGFbetaTII*	1267	100%	0.0	97.33%	ATY70063.1 (NCBI)

-	no reliable hit found
	retrieved in *O. minuta* genome with good support
	presence of members of the same family

<u>Table S12:</u> Blastp search results concerning G-proteins (against the predicted proteome of Oopsacas minuta; locus tags provided only if e-value<10-2) and reverse best blast hits (against NCBI NR database).

	P Blast against Oopsacas predicted proteome			reverse blast-p result against NR, best hit providing an annotated protein					
	searched protein	query sequence (species/accession number)	*O.minuta* genome reference	description	score	cover	e-value	identity	accession number
G-proteins	GNA12	*Mus musculus* / P27600 (Uniprot)	**LOD99_15437 (GNA12/13)**	guanine nucleotide-binding protein G(13) subunit alpha [Amphimedon queenslandica]	411	97%	1.00E-137	55.24%	XM_011404685.2
	GNAI	*Mus musculus* / B2RSH2 (Uniprot)	**LOD99_15292 (GNAI-a)**	guanine nucleotide-binding protein G(o) subunit alpha [Acanthopleura granulata]	426	97%	2.00E-145	56.62%	MN587143.1
			**LOD99_2939 (GNAI-b)**	guanine nucleotide-binding protein G(i) subunit alpha [Acanthopleura granulata]	414	99%	6.00E-141	56.34%	MN587142.1
	GNAQ	*Mus musculus* / P50148 (Uniprot)	**LOD99_360 / LOD99_361 / LOD99_85 (GNAQ-a)**	guanine nucleotide-binding protein G(q) subunit alpha [Amphimedon queenslandica]	311	98%	1.00E-99	43.38%	XM_003382741.3
			**LOD99_9371 / LOD99_9369 (GNAQ-b)**	guanine nucleotide-binding protein G(q) subunit alpha [Doryteuthis pealeii]	311	96%	3.00E-100	46.07%	KR107042.1
			**LOD99_1676 (GNAQ-c)**	guanine nucleotide-binding protein G(q) subunit alpha [Oryctolagus cuniculus]	271	98%	2.00E-84	38.71%	NM_001082715.1
	GNAS	*Mus musculus* / P63094 (Uniprot)	**LOD99_716 (GNAS)**	guanine nucleotide-binding protein G subunit alpha [Chaetura pelagica]	360	98%	3.00E-118	48.11%	XM_010006874.1
	GNAV	*Mus musculus* / B0V3V7 (Uniprot)	**LOD99_15202 / LOD99_15197 (GNAV)**	guanine nucleotide-binding protein G(o) subunit alpha [Geodia cydonium]	435	96%	4.00E-147	59.55%	Y14247.1
	GNB	*Mus musculus* / P62873 (Uniprot)	**LOD99_9728 (GNB1/4-a)**	guanine nucleotide-binding protein G subunit beta [Loa loa]	484	98%	2.00E-166	63.87%	XM_020450860.1
			**LOD99_4665 (GNB1/4-b)**	guanine nucleotide-binding protein G subunit beta [Tsuchiyaea wingfieldii]	315	93%	4.00E-102	46.76%	XM_019174048.1
			**LOD99_2821 (GNB5)**	guanine nucleotide-binding protein G(5) subunit beta [Saccoglossus kowalevskii]	293	76%	2.00E-92	47.57%	XM_002740478.2
	GNG	*Mus musculus* / Q61012 (Uniprot)	**LOD99_13691b (GBG-a)**	guanine nucleotide-binding protein G(T2) gamma [Pteropus vampyrus]	50.1	90%	5.00E-06	42.37%	XM_023524652.1
			**LOD99_14385 (GBG-b)**	guanine nucleotide-binding protein G(14) gamma [Terrapene carolina]	48.1	93%	3.00E-05	49.18%	XM_024219802.1

-	no reliable hit found
	retrieved in *O. minuta* genome with good support
	presence of members of the same family

<u>Table S13</u>: Blastp search and protein domain analyses concerning basic Helix Loop Helix (bHLH) transcription factors

BlastP searches against the predicted proteome of O.minuta were performed using various cnidarian and bilaterian sequences and reciprocal best hit against Human and *Drosophila Refseq protein databases as described in Fortunato et al., 2016. The domain analysis was performed using NCBI CD-Search (Yang et al,2020).*

The assignment to groups or families was refined by phylogenetic analysis (Fig. S8 and S9)

Identification	Family	Superfamily	High-order group	Domain(s)
LOD99_13791	-	Atonal-related	**A**	54 - 106: bHLH
LOD99_2605	E12/E47	-	**B**	379 - 431: bHLH
LOD99_14017	E12/E47	-	**B**	236 - 288: bHLH
LOD99_4504	E12/E47	-	**B**	452 - 504: bHLH
LOD99_6968	MITF	-	**B**	304 - 355: bHLH
LOD99_4176	SREBP	-	**B**	246 - 295: bHLH
LOD99_1394	AP4	-	**B**	315 - 366: bHLH
LOD99_13764	-	ARNT + BMAL	**C**	59 - 114: bHLH<br>300 - 349: PAS
LOD99_4226	-	ARNT + BMAL	**C**	99 - 152: bHLH
LOD99_4302	Clock		**C**	21 - 77: bHLH<br>237 - 291: PAS

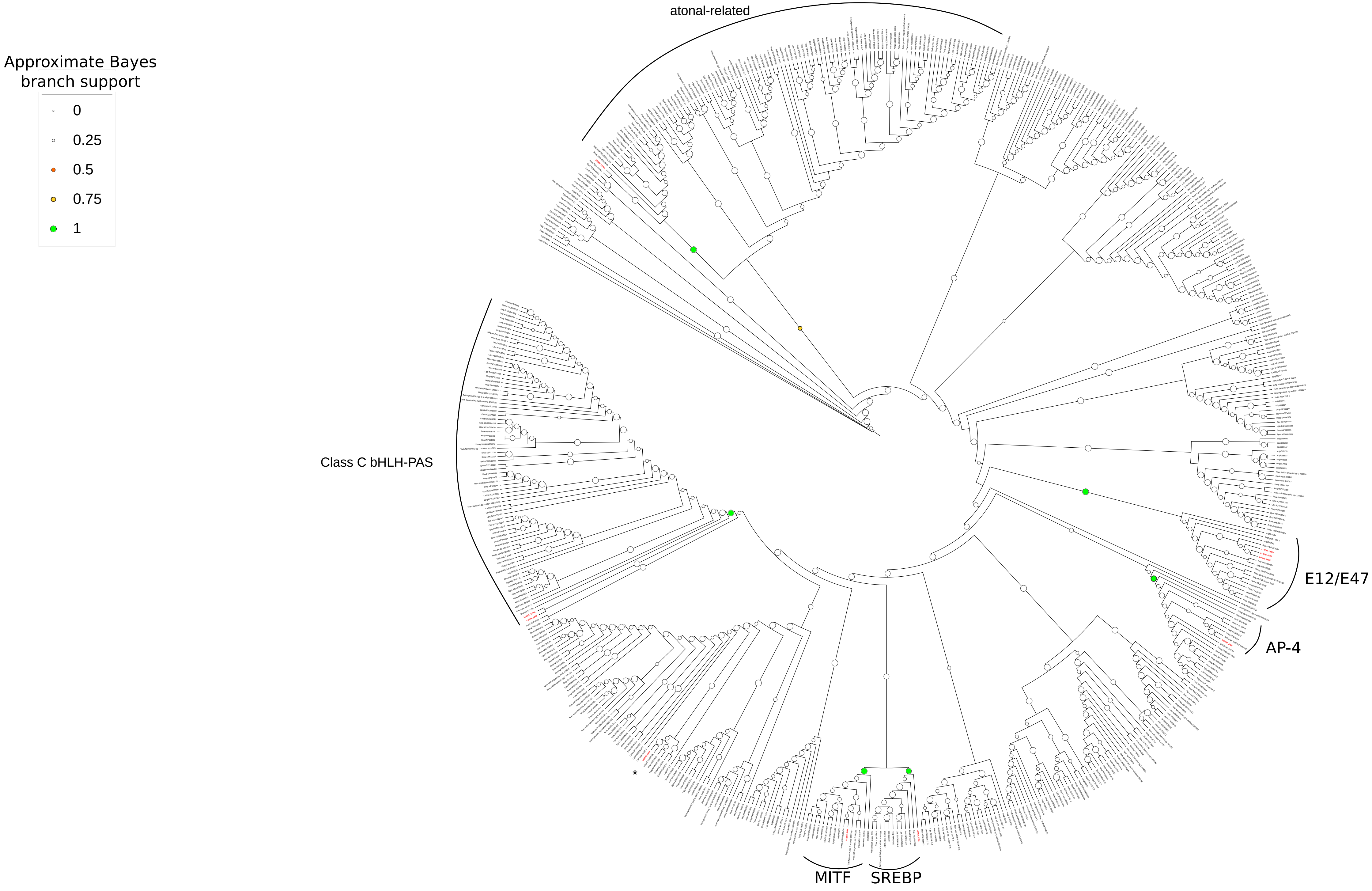

<u>Figure S8</u>: Phylogenetic relationships among bHLH transcription factors. Sequences from *O. minuta* are in bold red. The analysis was performed using PhyML (Guidon et al., 2010) with LG model as described in Fortunato et al. 2016, and the branch support estimated by an approximate bayes test (using the custom workflow provided at https://ngphylogeny.fr (Lemoine et al., 2019)). The alignment of bHLH domain is available at https://zenodo.org/communities/oops_13.The obvious misplacement of LOD99_4302* in the Hes/Hey family was resolved thanks to a second phylogenetic analysis (Additional file 3: Fig. S9).

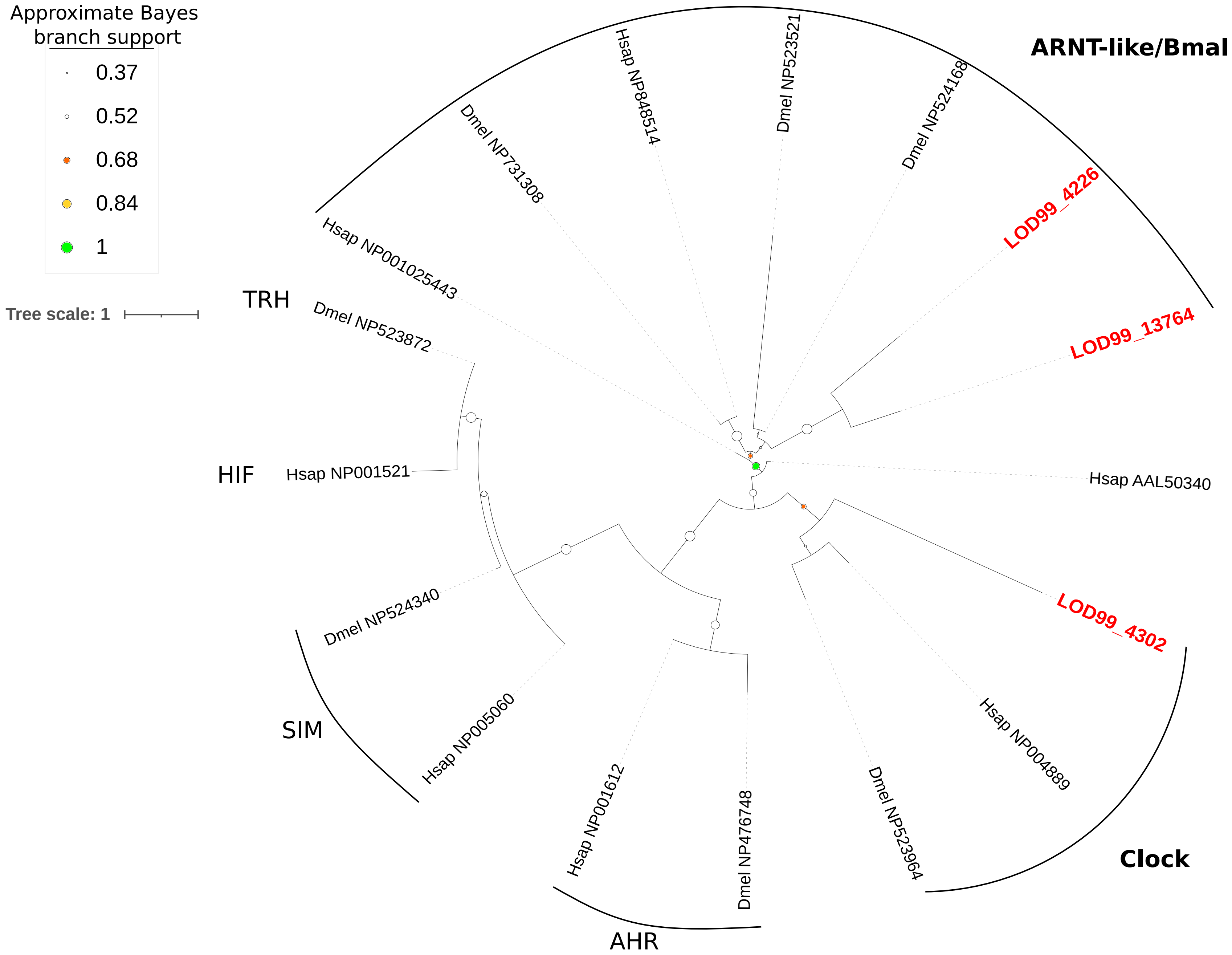

Figure S9: Phylogenetic position of *O. minuta* bHLH-PAS transcription factors.
The same method as described in Fig. S8 was used, using a set of sequences limited to bHLH-PAS sequences from *Drosophila melanogaster* (Dmel) and *Homo sapiens* (Hsap).

<u>Table S14:</u> Blastp search, domain and phylogenetic analyses performed on various transcription factor types with a particular focus on HD transcription factors

The BlastP search against the predicted proteome of *O. minut*a was performed using several queries from different non-bilaterian species: the cnidarian Nematostella vectensis (AAG37787.1; AAD39349.1; AFJ11248.1; AAO27886.2), from the placozoan *Trichoplax adhaerens* (AAC05783.1) and from the sponges *Sycon raphanus* and *S. ciliatum* (AAG28516.1; AAG28515.1; AAG28514.1; AAG28509.1; AAG28513.1; AAG28512.1; AAG28511.1; AAG28510.1; CEF71798.1; CEF71797.1; CEF71796.1; CEF71794.1; CEF71792.1; CEF71799.1; CEF71795.1; CEF71793.1) and *Amphimedon queenslandica* (XP_019851214.1; XP_011408876.1; XP_011409937.1; XP_011408017.1; XP_003387700.1; XP_003386306.1; XP_003383252.1; XP_019864339.1; XP_003387395.1; XP_019849423.1; XP_019849421.1; XP_011410538.1; XP_003384683.1 XP_019851578.1; XP_019849051.1; XP_019864471.1; XP_019863496.1; XP_011404713.1; XP_011410028.1; XP_003386365.1; NP_001292180.1; NP_001295556.1; NP_001292184.1; XP_011410308.1; XP_019858268.1; ACC76763.1; XP_011403814.2; XP_011410489.1; XP_011409566.1; XP_003389232.1; XP_003387530.1). Only the locus tags of *Oopsacas* sequences that gave reliable results (local Blast P e-values<10-2 + congruent reciprocal best hit against NR + domain prediction) are indicated here after. The protein domain prediction and phylogenetic analyses are available in Fig. S10 and S11.

DBD type	Locus tag in *O.minuta*	reciprocal best hit result						domain analysis	phylogeny	chosen annotation
		description	score	cover	e-value	identity	accesssion number	(Fig.S10)	(Fig.S11)	
HD	LOD99_6815	homeobox-containing protein [Ephydatia fluviatilis]	110	30 %	4.00E-27	69.44%	AAA20149.1	HD	probable divergent NkX (long branch)	HD containing transcription factor ANTP class
	LOD99_3608	homeobox-containing protein [Ephydatia fluviatilis]	141	22 %	2.00E-37	83.54%	AAA20151.1	HD	Msx	Msx
	LOD99_1044	BarX/Bsh [Halichondria bowerbanki]	98.2	24 %	1.00E-20	70.15%	AAQ24371.1	HD	Barx	BarH_Like
	LOD99_1602	homeobox protein DLX-3-like [Crassostrea virginica]	77.0	19 %	2.00E-12	47.95%	XP_022296381.1	HD	probable divergent NkX	HD containing transcription factor
	LOD99_7225	brain-specific homeobox Bsh [Mnemiopsis leidyi]	84.3	37 %	1.00E-16	60.00%	ACD85817.1	HD	Barx	BarH_Like
	LOD99_11824	paired-like homeodomain protein [Clytia hemisphaerica]	123	32 %	2.00E-29	59.41%	ALY05312.1	HD		Arx 1
	LOD99_4409	aristaless-related homeobox protein-like [Stylophora pistillata]	134	60 %	2.00E-34	54.11%	XP_022796171.1	HD		Arx 2
	LOD99_13863	LIM/homeobox protein Lhx3-like isoform X3 [Mizuhopecten yessoensis]	270	70 %	2.00E-84	47.93%	XP_021340799.1	HD + 2 LIM		Lhx
	LOD99_1585	PREDICTED: insulin gene enhancer protein ISL-2 isoform X2 [Callorhinchus milii]	201	82 %	2.00E-58	41.43%	XP_007906411.1	HD+ 2 LIM		Isl
	LOD99_2513	PREDICTED: POU domain protein 2-like [Octopus bimaculoides]	72.8	9 %	1.00E-09	52.54%	XP_014784998.1	HD + POU		Pou1
	LOD99_6803	ventral veins lacking protein [Monochamus alternatus]	202	48 %	2.00E-57	52.06%	AQY56479.1	HD + POU		Pou2
	LOD99_16229	REDICTED: pituitary-specific positive transcription factor 1-like [Branchiostoma belcheri]	247	58 %	4.00E-77	69.64%	XP_019639300.1	HD + POU		Pou3
	LOD99_13601	PREDICTED: hepatocyte nuclear factor 1-alpha-A-like isoform X2 [Amphimedon queenslandica]	171	35 %	1.00E-43	49.43%	XP_019859393.1	HD + HNF-1		Hnf-1
	LOD99_15015	homeobox protein six1a-like isoform X3 [Pomacea canaliculata]	340	57 %	4.00E-112	64.80%	XP_025105983.1	HD+ SIX		Six
	LOD99_14985	prep homeobox protein [Phallusia mammillata]	147	54%	4.00E-35	33.13%	CAB3265147.1	HD+KN+MeisPKnox		Meis
	LOD99_14669	PREDICTED: pre-B-cell leukemia transcription factor 1-like isoform X3 [Sinocyclocheilus anshuiensis]	153	68 %	2.00E-40	37.79%	XP_016344714.1	HD +PBC		Pbx 1
	LOD99_1385	Pbx [Oikopleura dioica]	162	64 %	1.00E-44	39.61%	AAW23083.1	HD +PBC		Pbx 2
	LOD99_13406	PREDICTED: homeobox protein TGIF2-like [Hyalella azteca]	67.4	29 %	6.00E-11	48.21%	XP_018015131.1	HD + KN		Tgif
	LOD99_15245	PREDICTED: homeobox protein TGIF2-like [Hyalella azteca]	77.0	23%	8.00E-14	51.61%	XP_018010193.1	HD + KN		HD containing transcription factor TALE class
	LOD99_15710	PREDICTED: flocculation protein FLO11-like [Amphimedon queenslandica]	104	23 %	3.00E-22	74.58%	XP_011403814.2	HD + KN		Irx
PRD	LOD99_13136A	Paired domain-containing protein [Caenorhabditis elegans]	182	24%	4.00E-48	51.38%	NP_501836.1	PRD		PRD containing protein
	LOD99_13136B	Paired domain-containing protein [Caenorhabditis elegans]	182	23%	3.00E-48	51.38%	NP_501836.1	PRD		PRD containing protein
SOX/HMG	LOD99_5639	transcription factor Sox14b [Takifugu rubripes]	146	47%	4.00E-39	54.40%	AAQ18499.1	HMG box		Sox
	LOD99_6974	soxB [Capitella teleta]	148	30%	4.00E-39	71.84%	AST23030.1	HMG box		Sox
	LOD99_2577	transcription factor Sox-14-like [Mizuhopecten yessoensis]	124	27%	2.00E-29	58.33%	XP_021356125.1	HMG box		Sox
	LOD99_3528	PREDICTED: high mobility group protein DSP1-like [Amphimedon queenslandica]	281	85%	6.00E-51	52.54%	XP_019862547.1	HMG box		HMG box containing transcription factor
	LOD99_9953	high mobility group-T protein-like [Acanthochromis polyacanthus]	151	90%	1.00E-42	43.60%	XP_022052747.1	HMG box		HMG box containing transcription factor
	LOD99_642	hypothetical protein A1O3_02036 [Capronia epimyces CBS 606.96]	80.9	25%	5.00E-13	45.33%	XP_007730369.1	Hmg box + bHLH		HMG box containing transcription factor
	LOD99_1071	hypothetical protein ILUMI_23413 [Ignelater luminosus]	98.2	22%	1.00E-17	40.74%	KAF2882749.1	HMG box		HMG box containing transcription factor
	LOD99_8961	Transcription factor [Oopsacas minuta]	1015	100%	0.0	100.00%	APZ80427.1	HMG box		TCF/LEF transcription factor family
T BOX	LOD99_3517	T-box-containing protein TBX6L-like [Octopus vulgaris]	208	27%	1.00E-57	49.24%	XP_029654306.1	T-Box		T-Box transcription factor
	LOD99_7913	hypothetical protein CAPTEDRAFT_110717 [Capitella teleta]	206	45%	5.00E-60	46.33%	ELT90467.1	T-Box		T-Box transcription factor
	LOD99_5104	Tbx4/5 protein [Podocoryna carnea]	254	55%	2.00E-77	61.03%	CAE45765.1	T-Box		T-Box transcription factor
	LOD99_3574	brachyury [Oopsacas minuta]	301	32%	6.00E-99	98.60%	AAU95753.1	T-Box		Brachyury

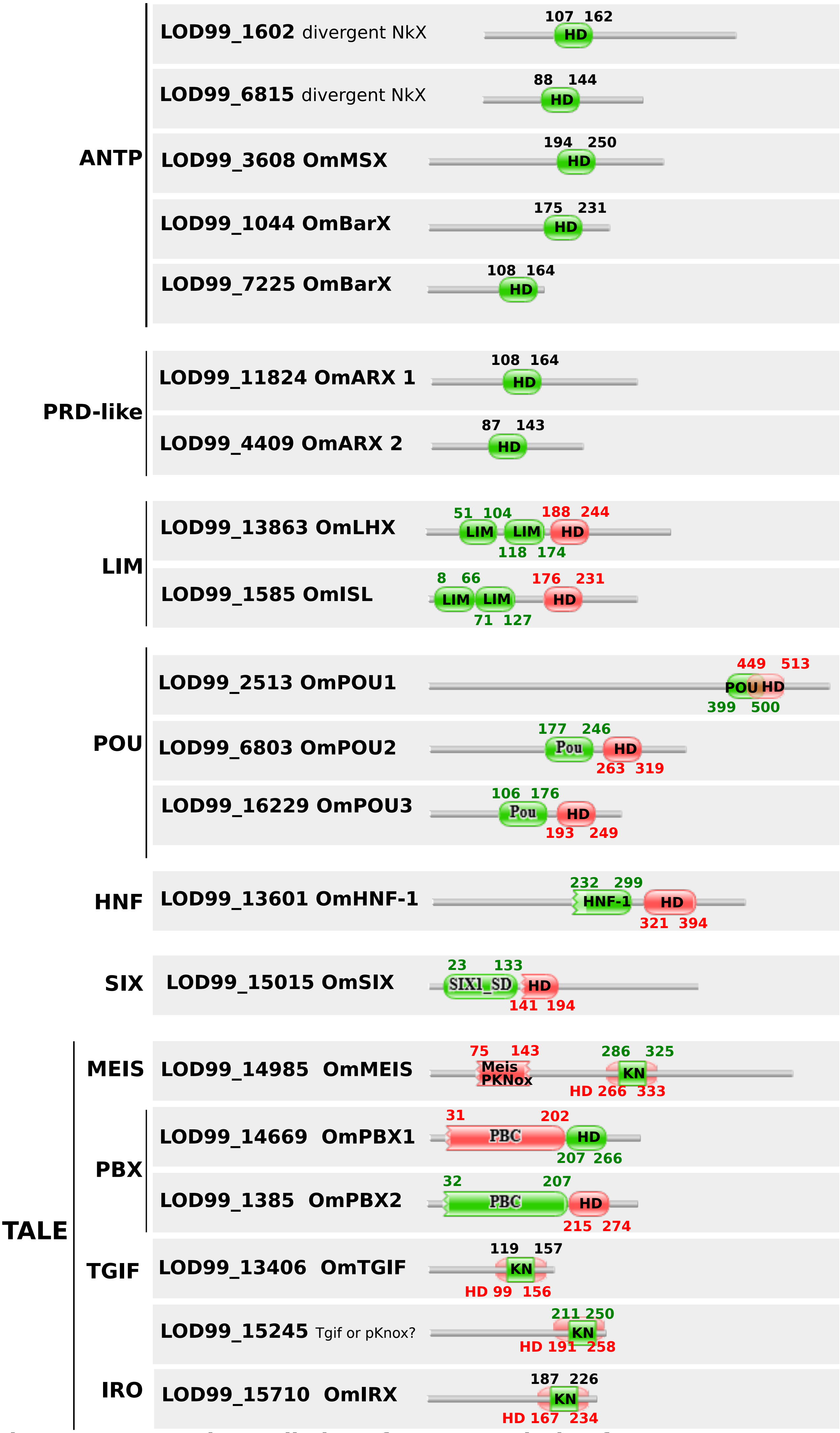

Figure S10: Domain prediction of HD transcription factors by Pfam (checked with InterProScan (Quevillon et al. 2005) for OmPOU1). Annotation according to Blastp hits and phylogenetic analysis: additional file 3: Fig.S11.

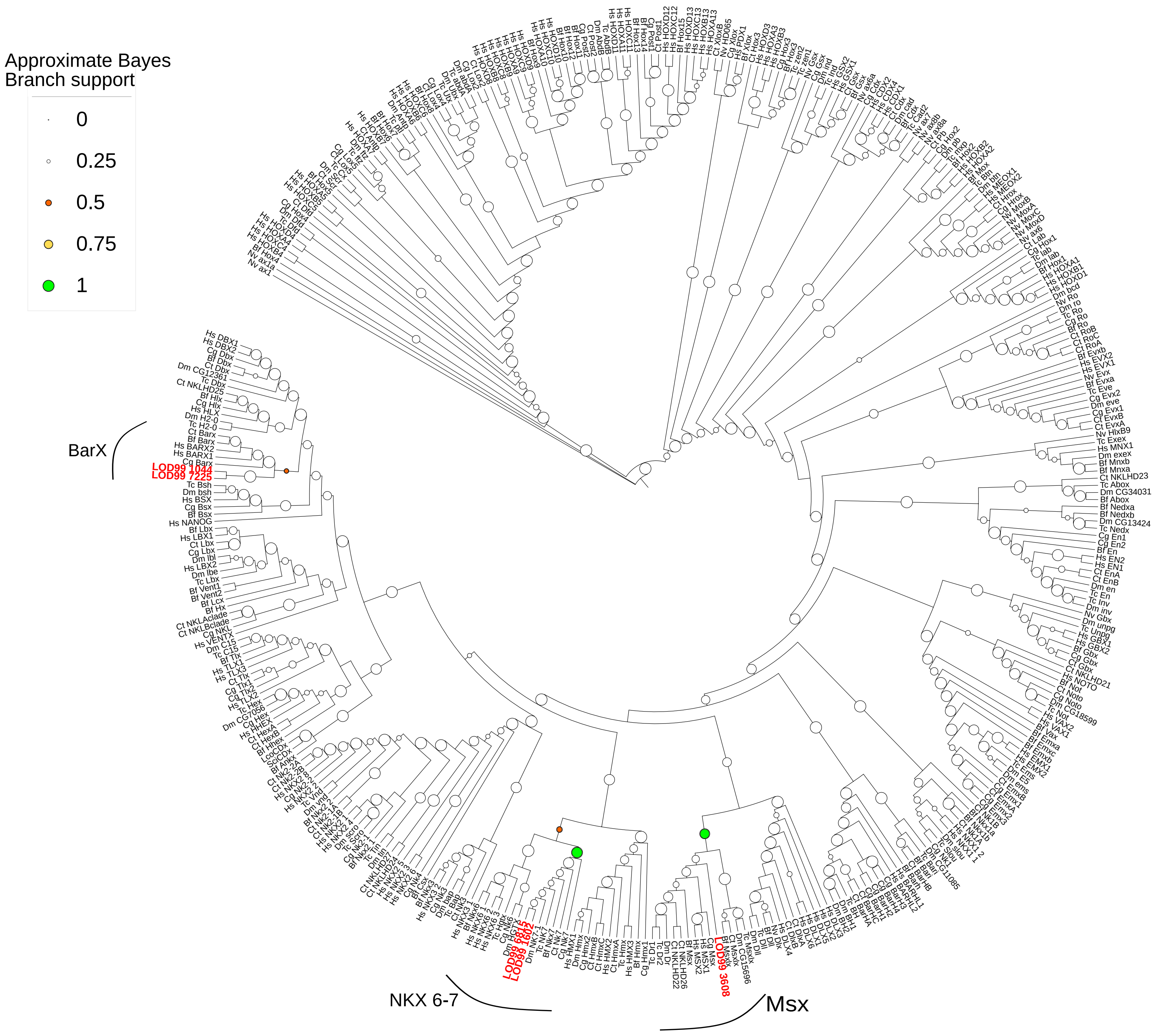

Figure S11: Phylogenetic positions of homeobox transcription factors of *O. minuta* assigned to the Antennapedia class (in red).
The *Oopsacas* HD sequences were integrated and aligned using MUSCLE (Edgar, 2004) in the dataset used by Pastrana *et al*., 2019, gathering sequences from *Homo sapiens* (Hs) *Capitella teleta* (Ct), *Crassostrea gigas* (Cg), *Nematostella vectensis* (Nv). The alignment is available at https://zenodo.org/communities/oops_13. The phylogenetic analysis was performed using PhyML (LG model, estimated gamma shape with 4 categories of substitution, approximate bayes branch support) at https://ngphylogeny.fr.

<u>Table S15:</u> Survey of candidate genes for photoreception. Blast P against the *O. minuta* predicted proteome, followed by a reciprocal best hit approach against NCBI NR database.

Query			Blast P against Oopsacas minuta predicted proteome		Reverse Blast againts NR				
**searched protein**	**description**	**ID**	**ID**	**e-value**	**best hit description**	**score**	**e-value**	**identity**	**accession**
**Cryptochrome/ photolyase**	cryptochrome-2-like [Amphimedon queenslandica]	XP_003386569.1	LOD99_7291	5.36×10^{-69}	photolyase related protein (Aphrocallistes vastus)	704	0	63%	CAD24679
			LOD99_6814	7.84×10^{-58}	cryptochrome (Crateromorpha meyeri)	606	0	54.49%	CAZ66368.1
Opsin	Opsin (Hydra vulgaris)	QHF16611.1	no hit found	with e.value<1.10^{-2}	_				
	long-wave-sensitive opsin 1-like [Hydra vulgaris]	XP_002163209.2	no hit found	with e.value<1.10^{-2}	_				
	medium-wave-sensitive opsin 1-like [Hydra vulgaris]	XP_002163358.2	LOD99_3226	$3,11.10^{-2}$	baculoviral IAP repeat-containing protein 6 isoform X2 [Choloepus didactylus]	704	0	30.31%	XP037663092.1
	opsin [Drosophila melanogaster]	AAA28733.1	no hit found	with e.value<1.10^{-2}	_				

present
absent

<u>Table S16:</u> Blastp search for proteins involved in chemical signaling against *Oopsacas* predicted proteome and reverse blast best hits againts the human NR database

	Blast P against *O. minuta* proteome					Reciprocal blast hit			Domain Prediction
**Searched proteins**	**Query sequence description and NCBI ID**		***Oopsacas minuta* locus tags**	**e value**	**#hits (e<-5)**	**Human hit rev blast**	**e value**	**Hum hit name**	(Pfam)
Monamine neurotransmitter molecules									
5HT receptor	5-hydroxytryptamine receptor 5B	P35365	LOD99_183	1.10-6	1	CAG46720.1	1.10-10	Dopamine receptor D1	7TM_GPCR_Srx
TpH	tryptophan 5-hydroxylase 1 [Homo sapiens]	NP_004170.1	LOD99_15798	1.10-159	1	same as PaH	1.10-178	phenylalanine-4-hydroxylase [Homo sapiens]	
AADC	aromatic-L-amino-acid decarboxylase isoform 4 [Homo sapiens]	NP_001229817.1	LOD99_3607	1.10-21	1	NP_000809.1	1.10-141	glutamate decarboxylase 2 [Homo sapiens]	
Th	tyrosine hydroxylase [Mus musculus]	AAA40434.1	LOD99_15798	1.10-142	1	same as PaH	same		
Qdpr	Quinoid dihydropteridine reductase [Mus musculus]	AAH02107.1	LOD99_14177	1.10-71	1	NP_000311.2	1.10-78	dihydropteridine reductase isoform 1 [Homo sapiens]	
slcl8a2	solute carrier organic anion transporter family member 1A2 [Homo sapiens]	NP_602307.1	LOD99_7596	7.12E-79	5	NP_057438.3	1.10-104	solute carrier organic anion transporter family member 4A1 [Homo sapiens]	
PaH	phenylalanine hydroxylase [Homo sapiens]	AAC51772.1	LOD99_15798	-179	1	NP_000268.1	1.10-178	phenylalanine-4-hydroxylase [Homo sapiens]	BiopterinH domain
Dbh	dopamine-beta-hydroxylase [Mus musculus]	AAB69054.1	LOD99_7269	-67	1	NP_056344.2	1.1074	DBH-like monooxygenase protein 1 precursor [Homo sapiens]	
Pnmt	phenylethanolamine-N-methyltransferase [Mus musculus]	EDL16137.1	LOD99_8458	1	0	AAH54342.1	1.10-83	EIF3A protein [Homo sapiens]	PINT (proteosome intiation factor) domain
Dopamine receptor	beta-2 adrenergic receptor [Mus musculus]	NP_031446.2	LOD99_183	1	1	CAG46720.1	1.10-10	DRD1 [Homo sapiens]	Pfam:7TM_GPCR_Srx
Glutamate									
Gl5	glutaminase [Homo sapiens]	AAF21933.1	LOD99_13155	3.31E-157	7	NP_055720.3	1.10-155	glutaminase kidney isoform, mitochondrial isoform 1 precursor [Homo sapiens]	
Glud1	glutamate dehydrogenase [Homo sapiens]	AAA20969.1	LOD99_15625	0	3	NP_005262.1	0	glutamate dehydrogenase 1, mitochondrial isoform a precursor [Homo sapiens]	ELFV_dehydrog_N domain
mGluR	metabotropic glutamate receptor 1 isoform alpha precursor [Homo sapiens]	NP_001264993.1	LOD99_14769	5.22E-81	6	NP_870989.1	1.10-109	metabotropic glutamate receptor 7 isoform b precursor [Homo sapiens]	
GAD	Glutamate decarboxylase GAD1 protein [Homo sapiens]	AAH36552.1	LOD99_3607	1.10-68	2	NP_000809.1	1.10-141	glutamate decarboxylase 2 [Homo sapiens]	
EAAT	glutamate transporter [Mus musculus]	AAK01708.1	LOD99_3448	1.10-116	1	NP_005062.1	1.10-120	excitatory amino acid transporter 4 isoform 2 [Homo sapiens]	
VGlut1	Brain-specific Na(+)-dependent inorganic phosphate cotransporter [Mouse]	Q3TXX4.2	LOD99_6988	9.48E-33	4	NP_065079.1	1.10-34	vesicular glutamate transporter 2 [Homo sapiens]	
**GABA**									
GABA	Gamma-aminobutyric acid type B receptor subunit 1	Q9Z0U4.1	LOD99_2448	1.56E-47	24	XP_011512755.1	1.10-49	gamma-aminobutyric acid type B receptor subunit 1 isoform X3 [Homo sapiens]	
			LOD99_3013	2.37E-47		BAD92027.1	1.10-47	gamma-aminobutyric acid (GABA) B receptor 1 isoform c precursor variant [Homo sapiens]	
			LOD99_3007	2.05E-44		NP_001305982.1	1.10-43	gamma-aminobutyric acid type B receptor subunit 1 isoform k [Homo sapiens]	
			LOD99_13509	2.30E-35		NP_005449.5	1.10-39	gamma-aminobutyric acid type B receptor subunit 2 precursor [Homo sapiens]	
			plus 19 more**						
ABAT	4-aminobutyrate aminotransferase, mitochondrial isoform 2 precursor [Mus mu	NP_001164449.1	LOD99_2634	2.79E-08	2	XP_005248394.1	1.10-89	alanine--glyoxylate aminotransferase 2, mitochondrial isoform X1 [Homo sapiens]	
TAT	tyrosine aminotransferase [Homo sapiens]	NP_000344.1	LOD99_10114	1.22E-124	7	NP_000344.1	1.10-123	tyrosine aminotransferase [Homo sapiens]	
Nitric Oxide									
NOS	nitric oxide synthase [Mus musculus domesticus]	BAA03415.1	LOD99_11608	2.07E-69	4	NP_000932.3	0	NADPH--cytochrome P450 reductase [Homo sapiens]	
sGC	guanylate cyclase [Mus musculus]	CAC41350.1	LOD99_7276	2.15E-143	91	NP_001513.2	1.10-120	retinal guanylyl cyclase 2 [Homo sapiens]	
			plus 87 more sequences**						
PKG-1	cGMP-dependent protein kinase 1 isoform 1 [Homo sapiens]	NP_001091982.1	LOD99_8398	0	289	NP_006249.1	0	cGMP-dependent protein kinase 1 isoform 2 [Homo sapiens]	
			plus 283 more sequences**						
**Acetylcholinesterase**									
Ach	acetylcholinesterase precursor [Bos taurus]	NP_001069688.1	LOD99_15812	4.78E-56	5	NP_000656.1	-57	acetylcholinesterase isoform E4-E6 precursor [Homo sapiens]	

** note some of the 'plus more sequences' appear to be different regions of the same gene

considered absent

present

Table S17: HMMER searches in *O. minuta* for voltage gated ion channels

HMMER (Johnson et al. 2010) searches were carried out with hmmer builds generated from alignments of ion channels (provided in Liebeskind et al. 2015).
HMMER profiles for Kv channels from Warren Gallin's Voltage Gated Potassium Channel database were also used (http://vkcdb.biology.ualberta.ca/).
For same sequence hits with different searches the highest e-value is given. This analysis is completed by the phylogenetic analysis provided in Fig. S12 and S13.

		full sequence			best domain			# domains		
	O. minuta Sequence	E-value	score	bias	E-value	score	best bias	exp	N	Description
CaV<br>voltage gated calcium channels	LOD99_1634	1.90E-41	141.5	43	6.10E-21	73.6	25.4	3	2	Two-pore channel 1
	LOD99_538	4.70E-41	140.2	18	5.50E-33	113.5	9.3	1.8	2	Cation channel sperm-associated protein 2
	LOD99_1635	5.20E-40	136.8	46.4	9.20E-20	69.7	28.7	3	2	Two-pore channel 1
	LOD99_3412	1.70E-37	128.4	19	4.20E-37	127.1	19.1	1.3	1	Hypothetical protein
	LOD99_2879	6.40E-37	126.5	53.5	9.80E-16	56.3	18	3.5	4	Two pore calcium channel protein 1 isoform X3
	LOD99_10799	8.90E-36	122.7	20	1.20E-35	122.3	20	1.1	1	Ion transporter
	LOD99_13596	1.80E-33	115.1	93.4	4.70E-14	50.7	34.6	3.5	4	MGC115225 protein
	LOD99_8690	full sequence	94.3	30.7	2.20E-21	75.1	15.6	2	2	Cation channel sperm-associated protein 3
	LOD99_16007	2.50E-12	45	5.3	4.40E-12	44.2	5.3	1.2	1	Sodium/hydrogen exchanger 10-like
	LOD99_5612	5.80E-06	23.9	17.7	4.90E-05	20.9	11.6	2	2	Hypothetical protein
NaV<br>voltage gated sodium channels	LOD99_1634	1.60E-35	121.5	40	1.60E-29	101.6	6.6	3.3	3	Two-pore channel 1
	LOD99_1635	5.90E-35	119.6	41.2	3.60E-29	100.4	7.2	3.3	3	Two-pore channel 1
	LOD99_3412	1.80E-34	118	27.8	6.90E-26	89.6	18.2	2	2	Hypothetical protein
	LOD99_538	9.70E-30	102.3	15.7	1.20E-17	62.2	7.4	2.1	2	Cation sperm-associated protein 2
	LOD99_8690	1.20E-28	98.8	30.8	2.10E-25	87.9	12.4	2	2	Cation sperm-associated protein 2
	LOD99_10799	5.00E-27	93.4	30.3	2.50E-26	91	29.8	1.9	1	Ion transporter
	LOD99_2879	1.90E-23	81.5	59.5	2.10E-14	51.5	20.4	4.2	4	Two pore calcium channel protein 1
	LOD99_16007	3.00E-21	74.2	10	5.40E-21	73.4	10	1.3	1	Sodium/hydrogen exchanger 10-like
	LOD99_13596	1.30E-19	68.8	57.1	1.30E-13	48.9	28.4	3.7	3	MGC115225 protein
	LOD99_5612	1.50E-09	35.4	17	1.90E-07	28.4	12.7	2	2	Hypothetical protein
	LOD99_13410	4.80E-07	27.1	7.4	4.80E-07	27.1	7.4	2.3	2	Cilia-and flagella-associated protein 43
	LOD99_10098B	0.0026	14.8	0	0.0029	14.6	0	1.1	1	Hypothetical protein
	LOD99_5102	0.0044	14	0.4	0.0056	13.6	0.4	1.1	1	Calmodulin and related proteins
	LOD99_10098	0.0046	13.9	0	0.0053	13.7	0	1.1	1	Hypothetical protein
Kv<br>voltage gated potassium channels	LOD99_1024	1.60E-17	63.7	106.2	8.70E-08	31.7	1.8	8.5	5	Hypothetical protein
	LOD99_8292	1.90E-16	60.2	0.3	3.60E-16	59.3	0.3	1.4	1	Potassium channel subfamily member 1
	LOD99_3527	5.30E-16	58.7	11.8	5.30E-16	58.7	11.8	2.2	2	Potassium channel subfamily member 1 isoform X21
	LOD99_12281	1.30E-14	54.2	1.1	2.60E-14	53.2	1.1	1.4	1	Potassium channel subfamily member 2 isoform X1
	LOD99_10799	3.40E-13	49.5	14.2	3.40E-13	49.5	14.2	2.8	4	Ion transporter
	LOD99_171	9.50E-13	48	0	1.40E-12	47.5	0	1.2	1	Putative potassium channel protein
	LOD99_3254	1.80E-12	47.1	0	2.10E-12	46.9	0	1.1	1	BTB/POZ domain-containing protein isoform X2
	LOD99_4149	4.60E-12	45.8	17.1	2.90E-10	39.8	0.2	2.9	3	Potassium channel subfamily member 17-like
	LOD99_15024	2.10E-11	43.6	0	2.60E-11	43.3	0	1.1	1	K+ channel tetramerisation protein
	LOD99_8690	2.70E-10	39.9	17.8	3.00E-10	39.8	17.2	1.4	1	Cation channel sperm-associated 3
	LOD99_3412	2.40E-09	36.8	21.1	6.70E-09	35.3	21.1	1.7	1	Hypothetical protein
	LOD99_4141	3.40E-09	36.3	0	4.80E-09	35.8	0	1.2	1	SH3KBP1-binding protein 1
	LOD99_4255	9.40E-08	31.6	10.5	1.60E-07	30.8	10.5	1.3	1	Hypothetical protein
	LOD99_538	3.00E-07	29.9	13.1	3.00E-07	29.9	13.1	2.1	3	Cation channel sperm-associated 2
	LOD99_2415	3.20E-07	29.8	0	4.80E-07	29.3	0	1.2	1	BTB/POZ domain-containing protein
	LOD99_2879	1.20E-06	27.9	9.3	1.20E-06	27.9	9.3	2.9	3	Two pore calcium protein 1 isoform X3
	LOD99_14396	8.20E-06	25.2	0	1.10E-05	24.8	0	1.1	1	Hypothetical protein
	LOD99_12269	1.10E-05	24.7	0	1.40E-05	24.4	0	1.3	1	BTB/POZ domain-containing adapter CUL3-mediate
	LOD99_16007	2.70E-05	23.5	6.6	2.70E-05	23.5	6.6	1.9	2	Sodium/hydrogen exchanger 10-like
	LOD99_7230	2.90E-05	23.4	0	3.90E-05	23	0	1.2	1	BTB/POZ domain-containing protein
EnAC	LOD99_3778	0.0015	18.3	0.7	0.0022	17.7	0.7	1.3	1	Transmembrane protein 206 partial
Leak	LOD99_1634	5.70E-34	116.8	47.1	1.30E-18	65.9	8.4	3	3	Two-pore channel 1
	LOD99_1635	1.40E-33	115.5	47.4	3.00E-18	64.6	8.6	3	3	Two-pore channel 1
	LOD99_2879	7.80E-27	93.1	56.7	8.40E-13	46.6	17	3.1	3	Two pore calcium protein 1 isoform X3

	LOD99_3412	1.60E-25	88.7	27.4	1.30E-14	52.7	17	2	2	Hypothetical protein
	LOD99_538	2.70E-25	88	15.8	6.20E-25	86.8	15.8	1.4	1	Cation channel sperm-associated 2
	LOD99_8690	2.30E-22	78.3	30.4	8.80E-18	63.1	13.1	2.3	2	Cation channel sperm-associated 3
	LOD99_10799	5.20E-18	63.9	30.1	6.70E-11	40.3	11.1	2.6	2	Ion transporter
	LOD99_13596	7.80E-18	63.3	61.7	7.90E-06	23.5	3.1	4.4	4	MGC115225 protein
	LOD99_11552	4.80E-07	27.5	0.1	0.00026	18.5	0	2	2	Myosin regulatory light
	LOD99_5612	1.40E-06	26	7.4	1.40E-06	26	7.4	1.8	2	Hypothetical protein
	LOD99_11553	3.70E-05	21.3	0	0.022	12.1	0	2	2	Myosin regulatory light
	LOD99_11551	9.60E-05	19.9	0.1	0.0056	14	0.1	2	2	Myosin regulatory light
	LOD99_5102	0.00066	17.1	0	0.009	13.4	0	2	2	Calmodulin and related
	LOD99_8750	0.0025	15.2	0	0.0043	14.4	0	1.5	2	Calmodulin
	LOD99_15670	0.003	15	0.1	0.011	13.1	0	1.7	2	Myosin regulatory light
	LOD99_2361	0.0051	14.2	0	0.02	12.2	0	1.9	2	Calmodulin
**Ano** anostamin	LOD99_15752A	5.70E-194	646	19.9	1.30E-185	618.4	18.1	3.9	2	Anoctamin-7-like
	LOD99_15752B	5.80E-194	646	19.8	1.20E-185	618.5	18	3.9	2	Anoctamin-7-like
	LOD99_170A	2.60E-180	600.9	22.6	2.40E-163	544.9	16.5	4.8	4	Anoctamin-7-like
	LOD99_170B	2.80E-180	600.8	22.6	2.40E-163	544.8	16.5	4.8	4	Anoctamin-7-like
	LOD99_170C	3.00E-180	600.7	22.6	2.50E-163	544.8	16.5	4.8	4	Anoctamin-7-like
	LOD99_1335	1.80E-173	578.3	20.7	5.10E-155	517.3	6.5	5.1	4	Anoctamin-7-like
	LOD99_15763	6.30E-101	338.7	9.7	1.10E-89	301.5	7	2	2	Anoctamin-7-like
	LOD99_15768	4.60E-35	121.1	1	2.80E-33	115.2	1	2	1	LOW QUALITY PROTEIN: anoctamin-2
	LOD99_15771	5.70E-27	94.3	0	4.00E-20	71.7	0	2.2	2	Hypothetical protein
**GIC (glutamate-gated ion channel family)**	LOD99_8292	0.44	9.7	4.3	0.81	8.8	4.3	1.3	1	Potassium channel subfamily member 1
**ClC** chloride channel	LOD99_4646	1.3E-211	704.3	24.1	2.3E-199	663.8	18.8	3	2	H(+)/Cl(-) exchange transporter isoform X4
	LOD99_2395	1.6E-182	608.3	17.6	8.3E-90	302	0	3	2	H(+)/Cl(-) exchange transporter
	LOD99_4967	6.1E-181	603	25.7	1.5E-94	317.7	16.9	3	3	H(+)/Cl(-) exchange transporter isoform X2
	LOD99_1206	4.6E-171	570.4	17.1	1.1E-170	569.1	17.1	1.5	1	Hypothetical protein
	LOD99_13962	1.9E-86	290.9	1.4	5.4E-68	229.9	1.6	2	2	Chloride transport protein isoform X4
	LOD99_13960	1.2E-83	281.7	14.1	2.2E-81	274.2	14.1	2.7	1	Chloride transport protein
**CNG-HCN** cyclic nucleotide-gated (CNG) channels hyperpolarization-activated cyclic nucleotide-modulated (HCN) channels	LOD99_1024	0	1294.5	75.6	3.7E-123	411.5	10.9	5.4	4	Hypothetical protein
	LOD99_239	4E-114	381.7	10.9	8.9E-73	245.5	0	2	2	Cyclic nucleotide-gated channel photoreceptor
	LOD99_238	1.6E-113	379.8	22.2	4.4E-112	375	22.2	2	1	Cyclic nucleotide-gated channel photoreceptor
	LOD99_3764	1.2E-57	195.6	21.1	4.1E-57	193.8	21.1	1.7	1	Cyclic nucleotide-gated cation beta-1 isof
	LOD99_1250	1.2E-54	185.7	22.6	5.3E-54	183.6	22.6	1.7	1	Cyclic-nucleotide-gated cation channel
	LOD99_238	4.1E-49	167.5	26.8	1.6E-47	162.2	26.8	2	1	Cyclic nucleotide-gated channel photoreceptor
	LOD99_8398	6E-46	157	1.1	6.1E-23	81.2	0.1	2.2	2	CGMP-dependent protein kinase
	LOD99_3573	1E-43	149.7	2.2	1E-26	93.6	0.1	2.9	3	CGMP-dependent protein kinase, 1
	LOD99_9579	7.9E-36	123.7	0	2.4E-17	62.7	0	2.1	2	CAMP-dependent protein kinase I-alpha regulat
	LOD99_7853	3.2E-34	118.4	0.1	4E-34	118	0.1	1.1	1	Cyclic nucleotide-gated olfactory partial
	LOD99_12665	2E-26	92.7	0.3	2.3E-13	49.6	0.1	2	2	CAMP-dependent protein kinase II-alpha regula
	LOD99_6963	3.5E-12	45.7	0.1	5.5E-12	45.1	0.1	1.3	1	Hypothetical protein
	LOD99_6099	3.1E-10	39.3	0.4	0.00000007	31.6	0.1	2	2	Cyclic nucleotide-binding domain-containing
	LOD99_3426	8.4E-09	34.6	0	0.063	11.9	0	3.2	3	Neuropathy target esterase,
	LOD99_349	0.00000014	30.5	0.3	0.00000085	28	0.2	1.9	2	Hypothetical protein
	LOD99_4149	0.0000019	26.8	7.3	0.000032	22.8	0.3	2.7	2	Potassium channel subfamily member 17-like
	LOD99_16007	0.00033	19.5	6.1	0.52	8.9	0	3.2	3	Sodium/hydrogen exchanger 10-like
	LOD99_8292	0.0019	17	0	0.0035	16.1	0	1.3	1	Potassium channel subfamily member 1
	LOD99_12281	0.0031	16.2	2.4	0.006	15.3	2.4	1.5	1	Potassium channel subfamily member 2 isoform X1
	LOD99_6960	0.0072	15	0	0.011	14.4	0	1.3	1	Hypothetical protein
**P2x** purinoreceptor	LOD99_4230	1.5E-100	336.8	2.7	2E-100	336.4	2.7	1	1	P2X purinoceptor 4-like X2
	LOD99_15028	5.4E-40	137.5	1.2	8.1E-22	77.6	0.2	2.7	2	Hypothetical protein
	LOD99_5071	0.0036	16.2	0	0.0049	15.8	0	1.2	1	Hypothetical protein
**PCC (polycystin cation channel)**	LOD99_3375	7.50E-42	143.4	32.2	6.10E-39	133.8	25	2.1	2	Mucolipin-3
	LOD99_4522	2.50E-21	75.7	16.9	2.50E-21	75.7	16.9	2.2	2	Transient receptor potential pyrexia-like
	LOD99_8690	2.40E-17	62.6	17.9	3.40E-17	62.1	17.9	1.2	1	Cation channel sperm-associated 3
	LOD99_3412	7.60E-16	57.6	18.9	2.40E-15	56	18.8	1.8	1	Hypothetical protein

	LOD99_1219	4.10E-13	48.6	22.1	8.20E-10	37.7	16	2.3	2	Hypothetical protein
	LOD99_10799	8.60E-11	41	24.9	8.60E-11	41	24.9	1.6	1	Ion transporter
	LOD99_538	2.50E-09	36.1	20.6	5.50E-09	35	20.6	1.5	1	Cation channel sperm-associated 2
	LOD99_5717	5.90E-09	34.9	17.8	5.90E-09	34.9	17.8	2	2	Transient receptor potential channel subfam
	LOD99_5720	1.10E-08	33.9	28.3	2.40E-08	32.9	28	1.5	1	Transient receptor potential channel subfam
	LOD99_1635	1.70E-08	33.3	55.1	2.50E-07	29.5	13.3	4.6	4	Two-pore channel 1
	LOD99_1634	3.00E-08	32.6	55.5	2.50E-07	29.5	13.3	4.6	4	Two-pore channel 1
	LOD99_6819	5.00E-06	25.2	22.8	5.00E-06	25.2	22.8	2.7	2	Transient receptor potential channel subfam
	LOD99_13596	5.60E-06	25.1	64.3	1.50E-05	23.7	21.4	5.2	2	MGC115225 protein
	LOD99_3407	1.20E-05	24	19.5	1.20E-05	24	19.5	2.1	2	Hypothetical protein
	LOD99_15407	1.70E-05	23.5	18.6	1.70E-05	23.5	18.6	3.2	3	Transient receptor potential channel subfam
	LOD99_7638	3.20E-05	22.6	0.3	3.20E-05	22.6	0.3	3.2	2	Ankyrin
	LOD99_7638	4.20E-05	22.2	0.3	4.20E-05	22.2	0.3	3.2	2	Ankyrin
	LOD99_14651	0.00024	19.7	1.8	0.00024	19.7	1.8	3.3	2	Hypothetical protein
	LOD99_5693	0.00028	19.5	1.1	0.00028	19.5	1.1	3	3	Vanilloid receptor
	LOD99_746	0.00033	19.3	0.7	0.00033	19.3	0.7	3.3	3	Ankyrin repeat protein
	LOD99_12646	0.00037	19.1	4.2	0.00037	19.1	4.2	3.6	4	Ankyrin repeat containing
	LOD99_12619	0.00085	17.9	4	0.00085	17.9	4	3.3	3	Hypothetical protein
	LOD99_2879	0.001	17.6	72.2	0.017	13.6	8.3	4.2	3	Two pore calcium protein 1 isoform X3
	LOD99_2914	0.0036	15.8	0.6	0.0036	15.8	0.6	3.4	4	Ankyrin repeat domain-containing 29-like
**RyR** **Ryanodine receptor**	LOD99_3002	0	1180.5	112.5	5.5E-178	594.1	65	6	4	Inositol 1,4,5-trisphosphate receptor 1 isofo
	LOD99_4139	0	1110.2	63.9	1.5E-188	629.1	15	5.1	4	Hypothetical protein
	LOD99_631	1.2E-208	695.8	79.1	6.1E-69	232.5	10.6	10.4	8	Ryanodine receptor 3
	LOD99_255	2.3E-189	631.8	87.8	2.9E-95	319.8	23.8	9.9	8	Ryanodine receptor 44F-like X1
	LOD99_4139	1.3E-170	569.7	11.7	3.2E-170	568.3	11.7	1.5	1	Hypothetical protein
	LOD99_3298	0.0014	15.8	0.3	0.052	10.6	0.00E+00	2	2	Hypothetical protein
**Kchannel Slo** High-conductance K+ channels encoded by the *Slo* gene family	LOD99_3527	1.2E-122	410.6	5.7	6.7E-90	302.3	4.2	2.6	2	Potassium channel subfamily member 1 isoform X21
	LOD99_4255	7.9E-112	374.8	2.7	6.3E-84	282.5	0.7	2.5	3	Hypothetical protein
	LOD99_8292	7.3E-105	351.8	4.2	1.3E-82	278.2	4.4	2.6	2	Potassium channel subfamily member 1
	LOD99_12281	1.5E-92	311.1	9	2.8E-91	306.9	3.2	3.3	3	Potassium channel subfamily member 2 isoform X1
	LOD99_1024	1.5E-14	53	60	0.0000016	26.4	1.8	4.9	4	Hypothetical protein
	LOD99_4149	5.7E-10	37.8	10.2	0.000000015	33.1	0.6	2.4	2	Potassium channel subfamily member 17-like
**Two Pore Channel**	LOD99_13596	3.6E-137	458.5	44.8	5.9E-94	315.6	28.5	2.1	2	MGC115225 protein
	LOD99_1634	3.1E-126	422.3	43.9	9E-102	341.5	35.4	2	2	Two-pore channel 1
	LOD99_1635	1.3E-125	420.2	43.2	1.2E-100	337.8	35.9	2	2	Two-pore channel 1
	LOD99_2879	5.3E-117	391.8	53.6	8.2E-97	325.1	42.5	2	2	Two pore calcium protein 1 isoform X3
	LOD99_538	1.7E-32	112.5	9.1	2.2E-32	112.1	9.1	1.1	1	Cation channel sperm-associated 2
	LOD99_3412	2.2E-32	112.2	20.3	2.7E-32	111.8	20.3	1.1	1	Hypothetical protein
	LOD99_10799	1.7E-30	105.9	21.9	2.1E-30	105.6	21.9	1	1	Ion transporter
	LOD99_8690	3.6E-22	78.4	22.1	3.6E-22	78.4	22.1	1.5	1	Cation channel sperm-associated 3
	LOD99_222	8.8E-20	70.5	1	5.2E-09	34.9	0	2	2	Centrin-3
	LOD99_3273	1.2E-19	70	2	2.1E-09	36.2	0.2	2	2	Caltractin isoform X1
	LOD99_6789	3.6E-17	61.9	0.4	9.3E-09	34.1	0.1	2	2	Calmodulin
	LOD99_2361	5.4E-17	61.3	0	0.000000005	35	0	2	2	Calmodulin
	LOD99_8750	1.4E-16	59.9	0	0.000000061	31.4	0	2	2	Calmodulin
	LOD99_16024	2.5E-16	59.1	8.2	3.3E-09	35.6	0.3	3.5	4	LOW QUALITY PROTEIN: protein 12
	LOD99_16025	6.7E-15	54.4	1.8	0.00000087	27.6	0.9	2	2	EF-hand_1 domain-containing protein,
	LOD99_13633	8.4E-14	50.7	0.3	0.0000015	26.8	0	2	2	Calmodulin-like
	LOD99_5102	1E-12	47.1	0.1	0.000015	23.5	0	2	2	Calmodulin and related
	LOD99_5612	9.2E-12	44	15.3	3E-11	42.3	8.9	2.1	2	Hypothetical protein
	LOD99_16023	1E-11	43.9	3	0.00000062	28	0.6	2	2	Calmodulin-A-like isoform X1
	LOD99_4496	5.7E-11	41.4	3.8	0.00000092	27.5	0.4	2	2	EF-hand calcium-binding domain-containing
	LOD99_11552	1.4E-10	40.1	0.2	0.000028	22.6	0	2.2	2	Myosin regulatory light
	LOD99_11553	1.7E-10	39.8	0.3	0.000021	23	0	2	2	Myosin regulatory light
	LOD99_11743	1.9E-10	39.7	0.4	0.000000012	33.7	0	2	2	Calmodulin-like protein 11
	LOD99_13997	2.3E-10	39.4	1	0.0000056	24.9	0.8	2	2	Hypothetical protein

	LOD99_8285	1.9E-09	36.4	0.7	0.000067	21.3	0.3	2.2	2	Calcium uptake protein mitochondrial isoform X2
	LOD99_655	0.000000011	33.8	3.2	0.00017	20	0.2	2.1	2	Calmodulin-like
	LOD99_13410	0.000000022	32.8	7.7	0.000000022	32.8	7.7	2.5	2	Cilia- and flagella-associated 43
	LOD99_13585	0.000000045	31.8	0.2	0.00000025	29.4	0.1	1.8	2	Calmodulin
	LOD99_4634	0.000000071	31.2	0	0.00018	19.9	0	2	2	Calcyphosin-like protein
	LOD99_1978	0.000000082	31	0.3	0.0004	18.8	0.1	2.1	2	Protein phosphatase 3 subunit
	LOD99_15670	0.000000088	30.9	0.1	0.0029	15.9	0	2	2	Myosin regulatory light
	LOD99_13996	0.00000011	30.5	1.7	0.0000011	27.3	1.5	1.9	2	Hypothetical protein
	LOD99_5752	0.00000012	30.4	0	0.0068	14.7	0	2	2	Myosin-2 essential light isoform X1
	LOD99_12384	0.00000055	28.2	1	0.00000072	27.8	1	1.1	1	Hypothetical protein
	LOD99_9717	0.00000056	28.2	0	0.00078	17.8	0	2.4	2	Calcium uptake protein mitochondrial-like isofo
	LOD99_14810	0.00000089	27.5	0.1	0.00000099	27.4	0.1	1.1	1	Calmodulin
	LOD99_15745	0.00000091	27.5	2.4	0.0000011	27.2	1	1.6	2	Hypothetical protein
	LOD99_13206	0.000001	27.3	0.1	0.0000014	26.8	0.1	1.1	1	Caltractin
	LOD99_11551	0.0000012	27.1	0.1	0.014	13.6	0.1	2	2	Myosin regulatory light
	LOD99_5921	0.0000016	26.7	0.1	0.000002	26.4	0.1	1.1	1	Troponin C, isoform
	LOD99_254	0.0000023	26.2	0	0.0043	15.4	1.00E-01	2.20E+00	2	Hypothetical protein
	LOD99_8752	0.0000028	25.9	1.4	0.013	13.8	0.5	2	2	Troponin isoform 2
	LOD99_4668	0.000011	23.9	0.7	0.0076	14.5	0	2	2	Hypothetical protein
	LOD99_15016	0.00003	22.5	0.2	0.0055	15	0.1	2.3	2	Neurocalcin homolog
	LOD99_12296	0.000047	21.8	1.6	0.0072	14.6	0.2	2	2	Sorcin isoform X1
	LOD99_631	0.00007	21.3	17.3	0.13	10.5	0	3.5	3	Ryanodine receptor 3
	LOD99_4139	0.000085	21	6.8	0.00018	19.9	6.8	1.4	1	Hypothetical protein
	LOD99_12355	0.000092	20.9	0	0.00014	20.3	0	1.1	1	Fimbrin
	LOD99_2539	0.00019	19.8	0	0.044	12	0	2	2	Hypothetical protein
	LOD99_13581	0.00033	19.1	0.1	0.00046	18.6	0.1	1.1	1	EF-hand domain-containing family B-like
	LOD99_13643	0.00037	18.9	0.7	0.00054	18.3	0.7	1.1	1	Glycerol-3-phosphate dehydrogenase, mitochondrial-
	LOD99_11717	0.00047	18.5	0.2	0.00058	18.2	0.2	1.2	1	Hypothetical protein
	LOD99_2930	0.00092	17.6	0.1	0.0015	16.9	0.1	1.2	1	WD repeat-containing protein Y chromosome-like
	LOD99_13512	0.0011	17.3	0.1	0.0065	14.8	0	1.8	2	Hypothetical protein, partial
	LOD99_4650	0.0015	16.9	0	0.002	16.4	0	1.2	1	EF-hand domain-containing protein
	LOD99_3177	0.0038	15.5	0.1	0.005	15.1	0.1	1.3	1	Apoptosis-linked gene 2
	LOD99_15804	0.006	14.9	0.4	0.0068	14.7	0.4	1.1	1	Allograft inflammatory factor
	LOD99_7638	0.0066	14.7	15.4	0.16	10.2	0.3	2.4	2	Ankyrin
**TRP channels**	LOD99_5717	8.2E-59	199.5	36.3	1.9E-44	152.3	31.6	9.2	3	Transient receptor potential channel subfam
**Transient receptor potential channel**	LOD99_5720	5.9E-57	193.4	40.4	4.2E-31	108.4	2.35E+01	9.70E+00	4	Transient receptor potential channel subfam
	LOD99_12740	1.6E-56	192	21.5	6.9E-56	189.9	2.15E+01	1.90E+00	1	Transient receptor potential channel subfam
	LOD99_13716	5.3E-54	183.7	53.7	5.8E-33	114.6	2.81E+01	9.30E+00	4	Transient receptor potential channel subfam
	LOD99_3407	3.4E-47	161.3	33.4	2.3E-35	122.4	2.48E+01	3.3	3	Hypothetical protein
	LOD99_1219	4.1E-47	161.1	15.2	9.6E-47	159.9	15.2	1.5	1	Hypothetical protein
	LOD99_4631	1.5E-43	149.3	19.1	8.2E-34	117.3	16.4	6.6	3	Transient receptor potential channel subfam
	LOD99_4522	3.2E-41	141.7	32.6	3.5E-38	131.7	32.6	3	1	Transient receptor potential pyrexia-like
	LOD99_6819	2.1E-38	132.4	25.8	6.3E-38	130.9	25.8	1.7	1	Transient receptor potential channel subfam
	LOD99_746	5.4E-35	121.2	21.6	6.1E-24	84.9	13.8	4.1	3	Ankyrin repeat protein
	LOD99_13591	5.6E-35	121.2	25.9	5.8E-20	71.8	19.4	6.6	3	AAEL014742-PA, partial
	LOD99_15407	1.8E-33	116.2	35	1.3E-32	113.4	35	2.3	1	Transient receptor potential channel subfam
	LOD99_9808	7.5E-33	114.2	28.4	2.6E-23	82.8	17.5	3.6	2	Ankyrin repeat protein
	LOD99_9808	2.3E-32	112.6	33.8	3.3E-23	82.5	17	5.5	2	LOW QUALITY PROTEIN: protein
	LOD99_12388	4.2E-32	111.7	26.4	1.1E-31	110.3	26.4	1.7	1	Transient receptor potential channel subfam
	LOD99_11812	1.1E-31	110.3	20	1.8E-17	63.6	18.9	5.5	2	Ankyrin-3
	LOD99_11859	3E-31	108.9	24.3	1E-16	61.1	21.7	5.2	3	Ankyrin-3-like isoform X3
	LOD99_2913	2.4E-30	105.9	22.9	4.1E-22	78.9	13.1	4.5	2	Hypothetical protein
	LOD99_2914	6.6E-30	104.5	18.8	2.3E-19	69.9	13.5	3.3	2	Ankyrin repeat domain-containing 29-like
	LOD99_12619	7.2E-30	104.4	23.4	6.6E-21	74.9	19.3	4.3	3	Hypothetical protein

LOD99_10303	4.1E-29	101.9	32.1	1.4E-19	70.5	17.9	6	3	Serine/threonine-protein phosphatase 6
LOD99_11817	5.8E-29	101.4	30.7	6.6E-18	65	21.5	5.4	3	Ankyrin repeat protein
LOD99_7638	1.7E-28	99.9	19	8E-21	74.6	13.5	3	2	Ankyrin
LOD99_7638	3.8E-28	98.7	21.4	1.2E-20	74.1	13.5	3.7	3	Ankyrin
LOD99_13881	2.8E-27	95.8	31.7	4.4E-21	75.5	23.1	2.1	2	Hypothetical protein
LOD99_712	5.7E-26	91.6	30.9	1.2E-22	80.6	30.9	3.2	1	Long-chain-fatty-acid--CoA ligase
LOD99_2912	7.2E-26	91.2	20.4	1E-16	61.1	14.2	3.7	3	Ankyrin repeat domain-containing 29-like
LOD99_2818	1.6E-25	90.1	22	1.5E-22	80.3	22	3.8	1	Hypothetical protein
LOD99_2880	2.8E-25	89.3	28.1	1.4E-17	63.9	21.7	4.3	2	Ankyrin repeat domain-containing 29-like
LOD99_5693	3.2E-25	89.1	21.2	2.5E-22	79.6	21.2	3.3	1	Vanilloid receptor
LOD99_12340	6.9E-25	88	29.4	1.3E-11	44.3	15.1	5.6	2	Ankyrin repeat protein
LOD99_12646	2E-24	86.5	35.3	2.1E-15	56.8	28.6	4.4	3	Ankyrin repeat containing
LOD99_15265	2.8E-24	86	52.6	9.5E-14	51.4	12.9	11.6	10	Ankyrin repeat protein
LOD99_15032	1.9E-23	83.2	44	5.3E-15	55.5	15.3	10.2	7	Hypothetical protein
LOD99_3470	2E-23	83.2	29.2	4.5E-14	52.4	14.7	5.7	3	Serine/threonine-protein phosphatase 6
LOD99_14651	6.8E-23	81.4	24.1	2.2E-20	73.2	23.9	3.8	1	Hypothetical protein
LOD99_5	5.6E-22	78.4	31.6	1.1E-13	51.1	21.7	5	2	AAEL017480-PA, partial
LOD99_5818	5.8E-22	78.4	34.3	1.4E-16	60.7	20.6	5.6	3	Hypothetical protein
LOD99_8688	2.7E-21	76.2	30.6	2.4E-13	50	17.2	5.4	2	Ankyrin-1-like
LOD99_3522	6.2E-20	71.7	22.9	1.4E-12	47.5	18.9	3.5	3	Hypothetical protein
LOD99_3669	1.6E-18	67.1	26.5	6.7E-14	51.9	26.5	5.1	1	Hypothetical protein
LOD99_954	4.7E-18	65.5	51.9	1.1E-12	47.8	33.3	6.1	4	Ankyrin-3-like
LOD99_5020	1.4E-17	64	27.4	0.000000027	33.4	21.1	5.8	4	Hypothetical protein
LOD99_13129	5E-17	62.2	23.3	1.8E-16	60.3	23.1	2	1	Hypothetical protein
LOD99_15022	1.8E-15	57	18.4	0.12	11.5	0.1	12.3	3	Serine/threonine-protein phosphatase 6
LOD99_4536	5.9E-14	52.1	35.2	9.5E-11	41.5	29.4	5	2	Ankyrin repeat protein
LOD99_3412	1.9E-13	50.3	19.3	3E-13	49.7	19.3	1.2	1	Hypothetical protein
LOD99_14015	2.1E-13	50.2	1	0.29	10.3	1	9.4	1	Ankyrin repeat and domain-containing protein ma
LOD99_3727	5.7E-13	48.8	1.3	0.17	11.1	0	7.7	4	Tankyrase-1-like
LOD99_2619	1.2E-11	44.4	1.7	0.043	13.1	1.8	7.1	1	Hypothetical protein, partial
LOD99_12876	4.1E-11	42.7	22.8	8.6E-10	38.4	23.8	2.6	1	Nacht and ankyrin protein
LOD99_7581	9.6E-11	41.5	0	0.016	14.5	0	5	1	Serine/threonine-protein kinase TNNI3K
LOD99_15322	1.3E-10	41.1	0	0.043	13.1	0.1	5.5	3	Ankyrin repeat domain-containing 27-like
LOD99_8983	3.2E-10	39.8	35.1	0.000022	23.8	22.2	4.5	2	Serine/threonine-protein phosphatase 6
LOD99_2912	3.6E-10	39.6	5.5	0.000007	25.5	0.2	3.7	3	Ankyrin repeat domain-containing 29-like
LOD99_2533	8.2E-10	38.4	34.5	0.000026	23.6	21.5	5.5	2	WD40 repeat-containing protein
LOD99_8690	1.9E-09	37.2	18	2.5E-09	36.8	18	1.3	1	Cation channel sperm-associated 3
LOD99_2491	2.1E-09	37.1	31.2	0.0000094	25.1	31.2	4.1	1	Ankyrin repeat domain-containing 50-like
LOD99_13879	0.000000003	36.6	2.9	0.15	11.3	0	6.6	4	Transient receptor potential channel subfam
LOD99_2619	4.8E-09	35.9	1.7	0.16	11.2	1.7	6.2	1	Hypothetical protein, partial
LOD99_2917	0.000000035	33.1	0.1	0.000000049	32.6	0.1	1.2	1	Ankyrin repeat domain-containing 29-like
LOD99_14107	0.00000015	31	1.2	0.23	10.6	0	4.1	4	Ankyrin repeat domain-containing 55-like
LOD99_5813	0.00000021	30.5	0.6	1.4	8.1	0	7.4	6	Ankyrin repeat protein
LOD99_9100	0.00000023	30.4	1.6	0.033	13.4	0.8	4.3	2	Ankyrin repeat domain-containing 16-like
LOD99_2916	0.00000028	30.1	1	0.0000003	30	1	1.1	1	Hypothetical protein
LOD99_11576	0.00000031	29.9	0	0.1	11.8	0	3.2	2	E3 ubiquitin-protein ligase isoform X1
LOD99_12215	0.00000049	29.3	1.1	0.18	11	0.4	3.7	3	AAEL017480-PA, partial
LOD99_4626	0.0000028	26.8	0.4	0.0079	15.5	0.1	3	3	Protein phosphatase 1 subunit 12A-like
LOD99_3521	0.0000036	26.5	2.6	3.7	6.7	0	6.5	5	Rabankyrin-5-like
LOD99_5743	0.000004	26.3	0.6	0.058	12.6	6.00E-01	3.90E+00	1	26S proteasome non-ATPase subunit 10-li
LOD99_538	0.0000059	25.8	25.6	0.000014	24.5	2.56E+01	1.40E+00	1	Cation channel sperm-associated 2

LOD99_2087	0.0000072	25.5	0.1	0.079	12.2	0.1	4.5	1	Death-associated protein kinase isoform X2
LOD99_4626	0.0000082	25.3	0.5	0.0034	16.7	0.3	3	2	MYPT-75D, partial
LOD99_14496	0.00001	25	2.5	0.068	12.4	0	4.9	4	Serine/threonine-protein phosphatase 6
LOD99_3375	0.000011	24.8	18.6	0.000024	23.8	18.6	1.4	1	Mucolipin-3
LOD99_14495	0.000014	24.5	4	0.5	9.6	0	5.7	3	Ankyrin repeat protein
LOD99_2298	0.000015	24.4	0.8	0.005	16.1	0.8	2.9	1	Ankyrin repeat domain-containing 42, parti
LOD99_46	0.000085	21.9	0.3	1.3	8.2	0.3	5.1	1	Inversin 2, partial
LOD99_7189	0.00009	21.9	0	2	7.6	0	5.2	1	Ankyrin repeat protein
LOD99_3598	0.00011	21.5	0.1	0.52	9.5	0.1	2.9	2	Ankyrin repeat domain-containing 39-like
LOD99_12649	0.00012	21.5	0	0.014	14.6	0	2.8	1	Tankyrase-2-like, partial
LOD99_660	0.00013	21.3	1	0.29	10.3	0.1	3.1	2	Oxysterol-binding protein-related protein
LOD99_15033	0.00015	21.1	5.6	1.5	8	0.6	6	3	Ankyrin repeat protein
LOD99_14405	0.00017	21	0.7	0.16	11.2	0.6	3.7	1	B-cell lymphoma 3
LOD99_13869	0.00023	20.5	0	0.11	11.7	0	3.5	2	Fibronectin type 3 ankyrin repeat domains prot
LOD99_12878	0.00024	20.5	0.4	5.8	6	0.1	5.2	3	Leucine-rich repeat serine/threonine-protein
LOD99_12278	0.00027	20.3	0	0.031	13.5	0	2.6	2	Cortactin-binding protein 2-like
LOD99_4487	0.00029	20.2	0.2	1.2	8.3	0	4.5	2	Ankyrin repeat and domain-containing prote
LOD99_8148	0.00078	18.8	0	0.0024	17.2	0	1.9	1	Ankyrin repeat and alpha motif domain-cont
LOD99_4375	0.0011	18.3	3.6	0.071	12.3	1.4	3.8	2	Ankyrin-3
LOD99_15420	0.0012	18.2	0.3	0.44	9.7	0.2	3.3	1	Caskin-2
LOD99_4987	0.0012	18.2	0	1.7	7.8	0	4	2	FEM-1
LOD99_10116	0.0012	18.1	0.5	1.3	8.1	0.1	4.4	2	Espin-like
LOD99_6090	0.0015	17.9	0.1	0.26	10.5	0.2	2.4	1	A Designed Ankyrin Protein With Four Identi
LOD99_1317	0.0016	17.7	0	0.2	10.9	0	2.2	1	Integrin-linked protein kinase-like X2
LOD99_15047	0.0017	17.7	0	0.22	10.7	0	2.4	1	Ankyrin repeat protein
LOD99_6550	0.0019	17.5	1	0.37	10	0.2	3	1	S-adenosyl-L-methionine-dependent tRNA 4-demethylw
LOD99_11432	0.002	17.4	0	0.023	14	0	2.3	1	Espin-like
LOD99_2666	0.0035	16.6	0.1	5.4	6.1	0.1	4.1	2	Espin-like
LOD99_7008	0.0047	16.2	0.1	0.57	9.4	0.1	2.4	1	NF-kappa-B inhibitor delta
LOD99_6688	0.0049	16.2	0	0.55	9.4	0	2.8	2	Espin-like
LOD99_12564	0.0053	16	0.1	0.21	10.8	0.1	2.5	1	Ankyrin repeat domain-containing 49-like
LOD99_11885	0.0073	15.6	0.1	0.27	10.4	0	2.5	2	Feminization-1b
LOD99_12649	0.0076	15.5	0	0.27	10.4	0	3.1	1	Leucine-rich repeat serine/threonine-protein
LOD99_4518	0.0095	15.2	0.4	0.7	9.1	0.4	2.9	1	Protein phosphatase 1 subunit 12A-like

present

considered absent

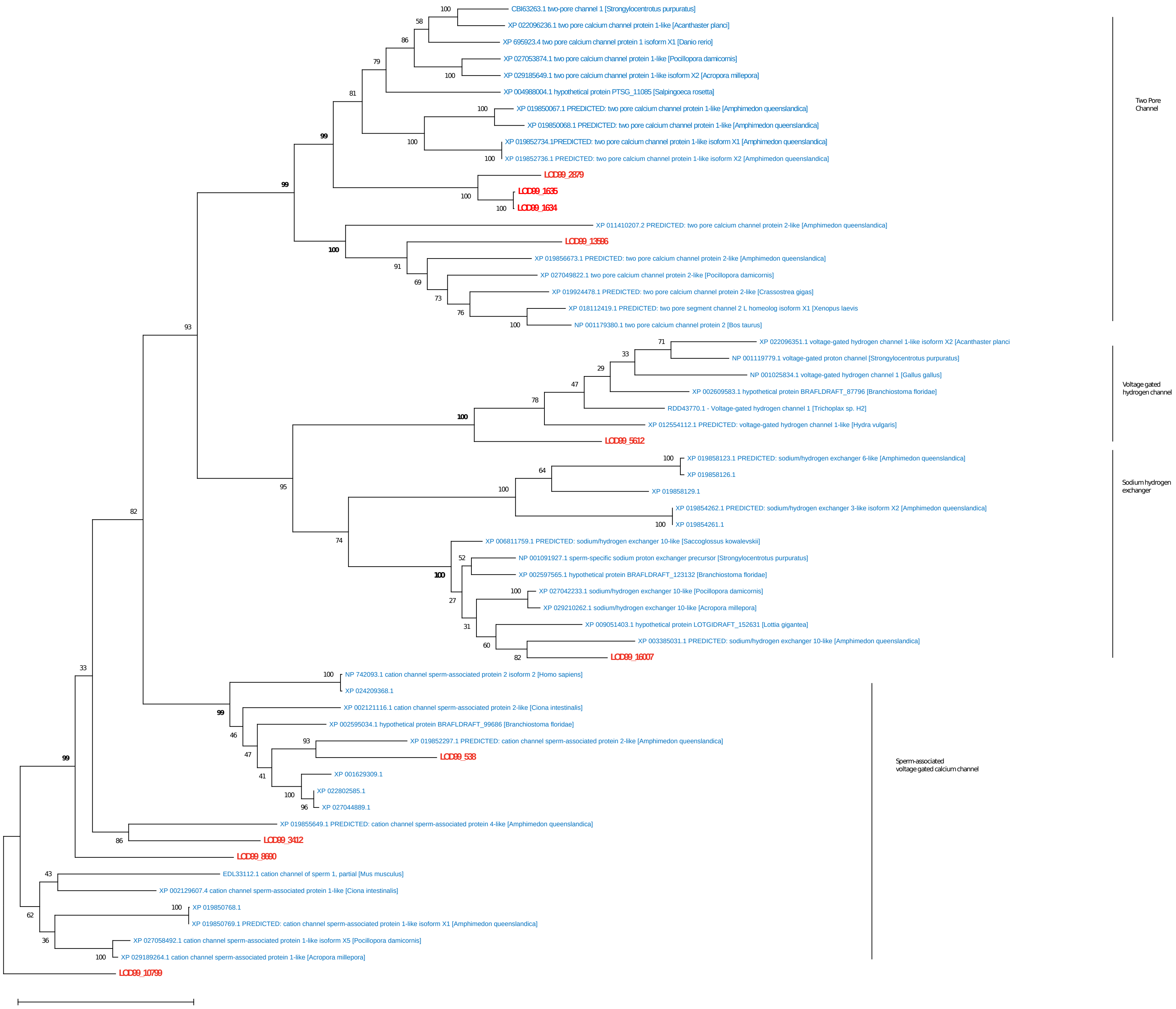

Figure S12: Diversity and phylogenetic positions of *Oopsacas* voltage gated ion channels.

The alignment was constructed using MAFFT (Katoh et al, 2002), trimmed With trimAL (Capella-Gutierrez et al. 2009). Phylogenetic trees were constructed using PhyML (Guindon et al. 2010) with the Smart Model Selection (Lefort *et al.*, 2017), then viewed with MEGA (Kumar et al 2018). The multiple alignment is available at https://zenodo.org/communities/oops 13.

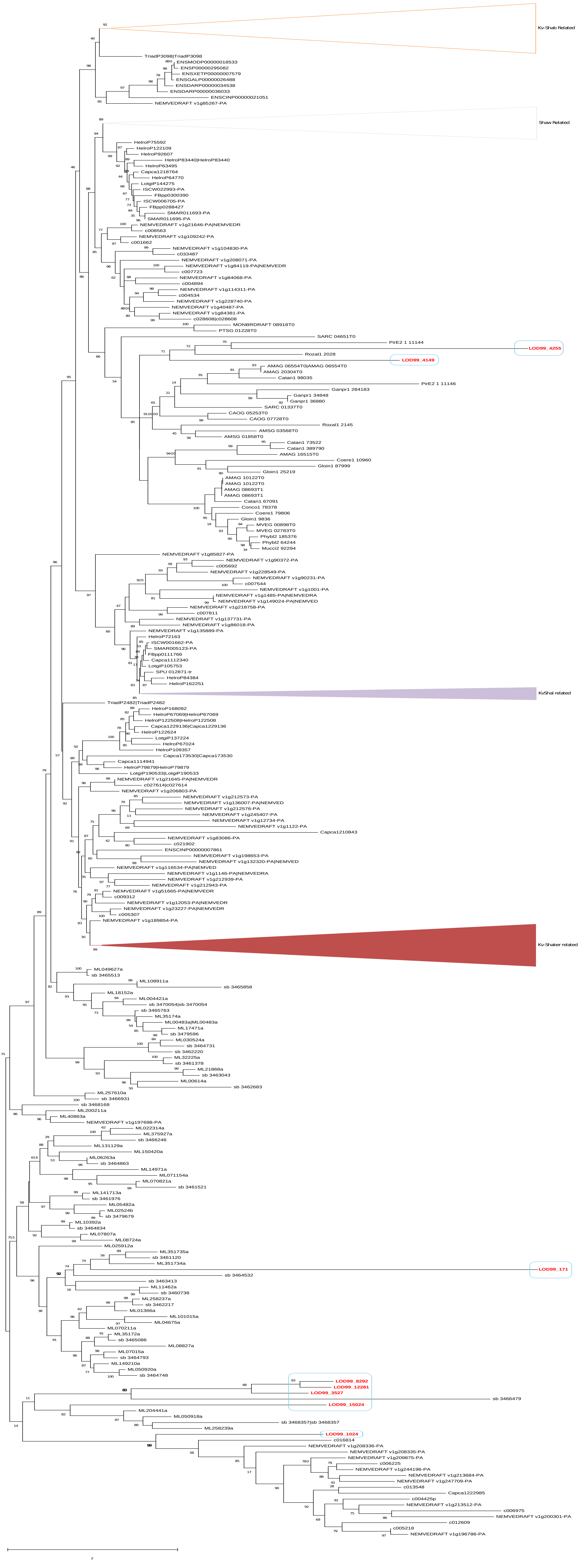

Figure S13: Phylogenetic positions of *Oopsacas* potassium channels (red), confirming that they are not related to voltage gated channels. The alignment was performed using MAFFT and trimmed using trimAL, it is available publicly at https://zenodo.org/communities/oops_13. The phylogenetic analysis was performed with PhyML (Smart Model Selection). Bootstrap support (1000 iterations) values are written at each node.

Table S18: Blast P search of proteins involved in bilaterian synapses against the predicted proteome of *O. minuta* and reverse blast best hits against NCBI human NR and general NR databases.

Searched proteins		Query		Blast P against O. minuta predicted proteome: hits	support		Reciprocal best hits against human NR			Reciprocal best hits against NR		
Category	protein name	NCBI ID	description	*Oopsacas minuta* locus tags	e value	#hits (e<-5)	Human hit rev blast	e value E-	Hum hit name	First rev blast hit (all species)	NCBI gene	e value E-
Pre-synaptic genes	DNA JC5	NP_001244572.1	dnaJ homolog subfamily C member 5 [Macaca mulatta]	LOD99_6857	9.43E-37	26	NP_001336361.1	-37	dnaJ homolog subfamily C member 5B [Homo sapiens]	dnaJ homolog subfamily C member 5-like isoform X1 [Pocillopora damicornis]	XP_027056181.1	-38
				LOD99_14682	5.50E-22							
				LOD99_2992	4.05E-18							
	Epsin1_Homo	NP_037465.2	epsin-1 isoform c [Homo sapiens]	LOD99_1145	9.20E-66	2	AAH93972.1	-72	Epsin 2 [Homo sapiens]	hypothetical protein BRAFLDRAFT_200079 [Branchiostoma floridae]	XP_002599355.1	-74
				LOD99_2284	7.31E-43							
	Neurexin	NP_004792.1	Neurexin isoform1	LOD99_2715	9.86E-19	5	NP_001136272.1	-74	protein eyes shut homolog isoform 1 precursor [Homo sapiens]	PREDICTED: neurogenic locus notch homolog protein 1-like [Saccoglossus kowalevskii]	XP_006823081.1	-125
	Pascin	NP_001186512.1	protein kinase C and casein kinase substrate in neurons protein 1 [Homo sapiens]	LOD99_9417	7.02E-46	4	NP_001171900.1	-45	protein kinase C and casein kinase substrate in neurons protein 2 isoform B [Homo sapiens]	protein kinase C and casein kinase substrate in neurons protein 2-like [Orbicella faveolata]	XP_020620621.1	-58
				LOD99_9047	2.73E-18							
				LOD99_210	6.37E-10							
				LOD99_9297	3.16E-07							
	Piccolo	NP_149015.2	protein piccolo isoform 1 [Homo sapiens]	LOD99_15363	1.07E-09	4	BAG65229.1	0	unnamed protein product [Homo sapiens]	PREDICTED: E3 ubiquitin-protein ligase NEDD4-like isoform X2 [Xenopus laevis]	XP_018110948.1	0
				LOD99_7374	2.61E-06					protein kinase C		
				LOD99_12634	3.48E-06					centrosomal protein		
				LOD99_19	3.49E-06					Delta		
	Profilin	EAW78854.1	profilin 2, isoform CRA_c [Homo sapiens]	Not present		0						
	SCAM	NP_036433.2	membrane-associated guanylate kinase, WW and PDZ domain-containing protein 2 isoform 1 [Homo sapiens]	LOD99_10136	2.39E-50	1	AUX13844.1	-55	MAGI-1b [Homo sapiens]	PREDICTED: cysteine proteinase 1-like isoform X1 [Amphimedon queenslandica]	XP_003386471.1	-115
	SNAP29	NP_004773.1	synaptosomal-associated protein 29 [Homo sapiens]	LOD99_1179	2.73E-10	1	BAD97337.1	-15	synaptosomal-associated protein 25 isoform SNAP25B variant [Homo sapiens]	PREDICTED: synaptosomal-associated protein 29-like [Hydra vulgaris]	XP_002155969.1	-17
	Synaphin	NP_006642.1	complexin-1 [Homo sapiens]	Not found	0.34	0						
	Synaptobrevin	NP_055046.1	vesicle-associated membrane protein 1 isoform 1 [Homo sapiens]	LOD99_4446	4.40E-19	2	NP_055046.1	-19	vesicle-associated membrane protein 1 isoform 1 [Homo sapiens]	vesicle-associated membrane protein 2-like [Melanaphis sacchari]	XP_025199128.1	-24
				LOD99_3822	2.28E-11							
	Synaptogyrin	CAA05322.1	synaptogyrin 1a [Homo sapiens]	LOD99_1009	1.88E-29	3	no hits			Synaptogyrin-3-like isoform 1		
				LOD99_1011	1.66E-16					Synaptogyrin-2-like		
				LOD99_5796	2.00E-10					Transmembrane channel-like protein 7		
	Synaptojanin	NP_003886.3	synaptojanin-1 isoform a [Homo sapiens]	LOD99_2854	0	8	XP_016883988.1	0	synaptojanin-1 isoform X6 [Homo sapiens]	PREDICTED: synaptojanin-1 isoform X3 [Anolis carolinensis]	XP_008105797.1	0
				LOD99_13134	3.76E-58					phosphatidylinositol-3-phosphatase SAC1		
				LOD99_11666	2.08E-47							
				LOD99_4796	6.15E-47							
				LOD99_4425	2.11E-44							
				LOD99_288	5.97E-39							
				LOD99_15421	1.02E-24							
				LOD99_2270	1.60E-19							
	Synaptoporin	NP_653243.1	synaptoporin isoform 2 precursor [Homo sapiens]	LOD99_2700	2.12E-07	1	no hits			PREDICTED: synaptophysin-like protein 1 [Amphimedon queenslandica]	XP_003384953.1	-32
	Synaptogamin	NP_001129277.1	synaptotagmin-1 isoform 1 [Homo sapiens]	LOD99_12634	2.24E-11	13	XP_005271958.1	-89	centrosomal protein of 120 kDa isoform X3 [Homo sapiens]	centrosomal protein	XP_001629704.1	-96
				LOD99_15776	5.78E-11					Copine-8		
				LOD99_10127	6.07E-11					rasGAP-activating-like protein 1		
				LOD99_4460	1.27E-10					PKC-alpha-beta-gamma		
				LOD99_7374	1.46E-10					protein kinase C		
				LOD99_5855	1.33E-08					Copine-9		
				LOD99_6604	1.97E-08					BAI1-associated protein 3-like		
	Syntaxin	NP_004594.1	syntaxin-1A isoform 1 [Homo sapiens]	LOD99_15683	7.42E-47	6	AAA20940.1	-52	syntaxin [Homo sapiens]	PREDICTED: syntaxin-like isoform X8 [Branchiostoma belcheri]	XP_019641353.1	-54
				LOD99_3843	5.99E-41							
				LOD99_12684	3.29E-09							
				LOD99_13883	2.32E-08							
				LOD99_14031	1.89E-07							
				LOD99_7767	2.64E-06							
Postsynaptic genes	Classical cadherin	NP_001783.2	cadherin-2 isoform 1 preproprotein [Homo sapiens]	LOD99_13082	2.16E-32	many	NP_001278214.1	0	protocadherin Fat 4 isoform 2 precursor [Homo sapiens]	PREDICTED: protocadherin Fat 4-like [Sinocyclocheilus rhinocerous]	XP_016379492.1	0
				LOD99_8123	2.32E-31							
				LOD99_15964	5.69E-31							
				LOD99_7816	4.43E-27							
				LOD99_14941	5.14E-27							
				LOD99_10096	2.08E-26							
	CASK	NP_003679.2	peripheral plasma membrane protein CASK isoform 1 [Homo sapiens]	LOD99_2802	6.06E-76	many	XP_006714393.1	-149	calcium/calmodulin-dependent protein kinase type II subunit delta isoform X12 [Homo sapien:	calcium/calmodulin-dependent protein kinase type II delta chain isoform X16 [Astatotilapia calliptera]	XP_026015572.1	-148
				LOD99_6818	2.10E-75							
				LOD99_4632	3.80E-61							
				LOD99_15011	3.58E-54							
				LOD99_6486	1.26E-48							
	ERBB-R	XP_005246434.1	receptor tyrosine-protein kinase erbB-4 isoform X8 [Homo sapiens]	LOD99_12748	1.07E-72	many	XP_016859071.1	-79	receptor tyrosine-protein kinase erbB-4 isoform X9 [Homo sapiens]	epidermal growth factor receptor isoform X1 [Diachasma alloeum]	XP_015119610.1	-89
				LOD99_5423	3.43E-58							
				LOD99_7259	1.82E-55							
				LOD99_428	2.01E-53							
				LOD99_13894	3.55E-53							
				LOD99_9475	1.26E-52							
				LOD99_14066	1.27E-52							
				LOD99_3486	2.46E-52							
				LOD99_428	4.40E-51							
	GKAP	NP_004737.2	disks large-associated protein 1 isoform 1 [Homo sapiens]	LOD99_4955	1.56E-06	2	BAH14105.1	-6	unnamed protein product [Homo sapiens]	disks large-associated protein 1 isoform X1 [Chelonia mydas]	XP_007063949.1	-4
				LOD99_13058	5.42E-06							
	GRIP	AAI15394.1	Glutamate receptor interacting protein 1 [Homo sapiens]	LOD99_10157	4.42E-32	many	XP_011532444.1	-34	glutamate receptor-interacting protein 2 isoform X2 [Homo sapiens]	Glutamate receptor-interacting protein 1 [Stegodyphus mimosarum]	KFM63452.1	-40
				LOD99_10153	8.40E-26							
				LOD99_10155	3.94E-19							
				LOD99_10136	1.19E-16							
				LOD99_8955	9.41E-16					DLG		
				LOD99_3535	9.41E-16					MPDZ		
	GABA-R	NP_001461.1	gamma-aminobutyric acid type B receptor subunit 1 isoform a precursor [Homo sapiens]	LOD99_2448	6.16E-51	23	XP_011512755.1	-49	gamma-aminobutyric acid type B receptor subunit 1 isoform X3 [Homo sapiens]	gamma-aminobutyric acid type B receptor subunit 1 [Drosophila willistoni]	XP_023032190.1	-61
				LOD99_3013	2.61E-48							
				LOD99_3007	3.62E-47							
				LOD99_13509	2.83E-40							
				LOD99_13510	4.98E-39							
				LOD99_2980	2.16E-33							
				LOD99_15816	2.57E-33							
	Homer	NP_004263.1	homer protein homolog 1 isoform 1 [Homo sapiens]	LOD99_173	1.56E-52	2	AAH47438.1	-53	Homer homolog 1 (Drosophila) [Homo sapiens]	putative homer protein-like 2 isoform X2 [Apostichopus japonicus]	PIK37620.1	-69
				LOD99_14732	6.67E-08							
	Neuroligin	NP_001352858.1	neuroligin-1 isoform 3 [Homo sapiens]	LOD99_9823	1.09E-41	5	6EMI_A	-56	Chain A, Cholinesterase [Homo sapiens]	putative cholinesterase [Heterostelium album PN500]	XP_020429363.1	-92
				LOD99_15812	1.96E-40							
				LOD99_15043	6.51E-35							
				LOD99_6636	1.26E-33							
				LOD99_9822	3.90E-33							
	Alpha catenin	NP_001310911.1	catenin alpha-1 isoform 1 [Homo sapiens]	LOD99_9843	9.22E-53	3	NP_001269527.1	-63	catenin alpha-2 isoform 4 [Homo sapiens]	alpha-catenin [Oopsacas minuta]	AVM85906.1	0
				LOD99_722	1.21E-24							
				LOD99_6852	4.23E-20					Vinculin		
	Beta catenin	NP_001091679.1	catenin beta-1 isoform 1 [Homo sapiens]	LOD99_2816	3.66E-115		NP_001317658.1	-115	catenin beta-1 isoform 2 [Homo sapiens]	Beta-catenin [Oopsacas minuta]	APZ80422.1	0

Colour	Meaning
green	present
red	considered absent
orange	unclear

Table S19: Results of HMMER searches for SNARES proteins present in the predicted proteome of *O. minuta* followed by a reciprocal blast best hit approach evidencing several syntaxins and SNAP proteins.

To perform this search 4 distinct HMMER profiles were built and used: one on human sequences only, and three others, each focused on a subfamily (Qa , Qb and Qc) gathering sequences of species representative of the main metazoan taxa: Vertebrata (*Homo sapiens, Neophocaena asiaeorientalis, Carassius auratus, Python bivittatus, Microcebus murinus, Cynoglossus semilaevis, Xenopus laevis, Astyanax mexicanus*), Cephalorchordata (*Branchiostoma belcheri*), Arthopoda (*Centruroides sculpturatus, Caligus rogercresseyi*), Nematoda (*Loa loa*), Mollusca (*Octopus bimaculoides*), Cnidaria (*Hydra vulgaris, Acropora millepora, Stylophora pistillata*), Placozoa (*Trichoplax sp.*), Porifera (*Amphimedon queenslandica*); and the Alveolata *Trypanosoma brucei*.The four sequence datasets used to build each HHMER profile are available at https://zenodo.org/communities/oops_13.

RESULTS OF HMMER SEARCH				RECIPROCAL BLAST P ON NCBI NR DATABASE: Best hits		
HMMER profile Query	E-value (Full Sequence)	*Oopsacas* locus tag	*Oopsacas* gene Descritption	E-value	description	accession number ID
human SNAREs	2.00E-10	LOD99_12684	Hypothetical protein	3.00E-58	syntaxin-16 isoform d [Homo sapiens]	NP_001128245.1
	8.30E-06	LOD99_3843	Syntaxin-3	4.00E-47	syntaxin-1B isoform X1 [Homo sapiens]	XP_016878382.1
	0.001	LOD99_15683	Syntaxin-1A isoform X3	1.00E-52	syntaxin [Homo sapiens]	AAA20940.1
	0.006	LOD99_2734	Hypothetical protein	2.00E-08	Chain B, BACULOVIRAL IAP REPEAT-CONTAINING PROTEIN 7 [Homo sapiens]	4AUQ_B
	0.0094	LOD99_8299	Hypothetical protein	_	*no hit*	_
Qa-SNARES	1.10E-57	LOD99_12684	Hypothetical protein	3.00E-58	syntaxin-16 isoform d [Homo sapiens]	NP_001128245.1
	6.50E-54	LOD99_632	Hypothetical protein	4.00E-77	Syntaxin-5 [Trichoplax sp. H2]	RDD42967.1
	1.10E-45	LOD99_3843	Syntaxin-3	6.00E-50	syntaxin-1A-like isoform X1 [Acropora millepora]	XP_029194329.1
	1.30E-40	LOD99_15683	Syntaxin-1A isoform X3	5.00E-54	PREDICTED: syntaxin-like isoform X8 [Branchiostoma belcheri]	XP_019641353.1
	2.70E-36	LOD99_13883	Syntaxin-7	3.00E-41	syntaxin-7 isoform X2 [Neophocaena asiaeorientalis]	XP_024614921.1
	8.70E-25	LOD99_14031	Syntaxin-12	2.00E-12	syntaxin-7-like [Carassius auratus]	XP_026146358.1
	6.80E-23	LOD99_1731	Syntaxin-18 isoform X2	2.00E-23	syntaxin-18 [Python bivittatus]	XP_007437315.2
	3.70E-08	LOD99_11618	Hypothetical protein	6.00E-19	syntaxin-17 [Microcebus murinus]	XP_012624451.1
	2.60E-05	LOD99_12196	Syntaxin-6-like isoform X2	5.00E-26	PREDICTED: syntaxin-6-like isoform X2 [Octopus bimaculoides]	XP_014784937.1
	0.0005	LOD99_7767	Hypothetical protein	_	*no hit*	_
	0.0016	LOD99_9730	Hypothetical protein	2.00E-12	vesicular membrane trafficking protein p18 [Loa loa]	XP_020304007.1
	0.0096	LOD99_6844	BET1-like protein	2.00E-15	BET1-like protein [Cynoglossus semilaevis]	XP_024911915.1
Qb-SNARES	1.10E-38	LOD99_4126	Hypothetical protein	1.00E-49	Golgi SNAP receptor complex member 1 [Trichoplax sp. H2]	RDD46535.1
	1.10E-33	LOD99_4578	Vesicle transport through interaction with t-SNARE	7.00E-38	vesicle transport through interaction with t-SNAREs homolog 1A-like [Stylophora pistillata]	XP_022809715.1
	8.20E-18	LOD99_2115	Vesicle transport protein SEC20	5.00E-05	vesicle transport protein SEC20-like [Centruroides sculpturatus]	XP_023213355.1
	7.20E-13	LOD99_2350	Golgi SNAP receptor complex member	6.00E-11	Golgi SNAP receptor complex member 2 [Caligus rogercresseyi]	ACO10315.1
	6.50E-09	LOD99_1179	Synaptosomal-associated protein 29-like	2.00E-17	PREDICTED: synaptosomal-associated protein 29-like [Hydra vulgaris]	XP_002155969.1
	2.30E-08	LOD99_9724	Synaptosomal-associated protein 25-A-like	9.00E-46	PREDICTED: synaptosomal-associated protein 25-A-like [Amphimedon queenslandica]	XP_003388483.1
	1.00E-05	LOD99_12196	Syntaxin-6-like isoform X2	5.00E-26	PREDICTED: syntaxin-6-like isoform X2 [Octopus bimaculoides]	XP_014784937.1
	2.00E-05	LOD99_9730	Hypothetical protein	2.00E-12	vesicular membrane trafficking protein p18 [Loa loa]	XP_020304007.1
	0.0022	LOD99_12863	Biogenesis of lysosome-related organelles complex	5.00E-20	biogenesis of lysosome-related organelles complex 1 subunit 6 [Astyanax mexicanus]	XP_022542735.1
	0.0036	LOD99_632	Hypothetical protein	4.00E-77	Syntaxin-5 [Trichoplax sp. H2]	RDD42967.1
Qc-SNARES	1.10E-34	LOD99_12196	Syntaxin-6-like isoform X2	5.00E-26	PREDICTED: syntaxin-6-like isoform X2 [Octopus bimaculoides]	XP_014784937.1
	1.60E-20	LOD99_12198	MGC78971 protein	2.00E-26	MGC78971 protein [Xenopus laevis]	AAI08777.1
	5.90E-19	LOD99_9730	Hypothetical protein	2.00E-12	vesicular membrane trafficking protein p18 [Loa loa]	XP_020304007.1
	1.00E-10	LOD99_13883	Syntaxin-7	3.00E-41	syntaxin-7 isoform X2 [Neophocaena asiaeorientalis]	XP_024614921.1
	1.30E-10	LOD99_16136	Poly(rC)-binding protein 2	7.00E-23	heterogeneous nuclear ribonucleoprotein K-like [Acropora millepora]	XP_029186313.1
	4.90E-08	LOD99_6844	BET1-like protein	2.00E-15	BET1-like protein [Cynoglossus semilaevis]	XP_024911915.1
	3.00E-07	LOD99_14031	Syntaxin-12	2.00E-12	syntaxin-7-like [Carassius auratus]	XP_026146358.1
	1.50E-06	LOD99_1179	Synaptosomal-associated protein 29-like	2.00E-17	PREDICTED: synaptosomal-associated protein 29-like [Hydra vulgaris]	XP_002155969.1
	1.50E-06	LOD99_632	Hypothetical protein	4.00E-77	Syntaxin-5 [Trichoplax sp. H2]	RDD42967.1
	2.70E-05	LOD99_12684	Hypothetical protein	3.00E-58	syntaxin-16 isoform d [Homo sapiens]	NP_001128245.1
	0.00013	LOD99_9724	Synaptosomal-associated protein 25-A-like	9.00E-46	PREDICTED: synaptosomal-associated protein 25-A-like [Amphimedon queenslandica]	XP_003388483.1
	0.00014	LOD99_3843	Syntaxin-3	6.00E-50	syntaxin-1A-like isoform X1 [Acropora millepora]	XP_029194329.1
	0.00075	LOD99_8951	Hypothetical protein	_	*no hit*	_
	0.0055	LOD99_4578	Vesicle transport through interaction with t-SNARE	7.00E-38	vesicle transport through interaction with t-SNAREs homolog 1A-like [Stylophora pistillata]	XP_022809715.1

<u>Table S20:</u> Blast P search of proteins involved in silica biogenesis against *Oopsacas* predicted proteome, and reverse blast (against NR) best hit identity.

BlastP against *O. minuta* predicted proteome					Reverse Blast against NR
Name of protein searched	Query	Accession number/ID	Hit ID Oopsacas	evalue	best hit description/chosen annotation
Silicatein	silicatein-like protein [Aulosaccus sp. GV-2009]	ACU86972.1	NONE		
Silintaphin	silintaphin-1 [Suberites domuncula]	CAP16640.1	NONE		
galectin	galectin [Suberites domuncula]	CAD37940.1	NONE		
Cathepsin L	cathepsin L [Sympagella nux]	Own contig	LOD99_12702	2.52 × 10-102	Cathepsin L
Cathepsin L	cathepsin L [Sympagella nux]	Own contig	LOD99_4353	2.18 × 10-100	Cathepsin L
Cathepsin L	cathepsin L [Sympagella nux]	Own contig	LOD99_10364	5.08 × 10-92	Cathepsin L
Cathepsin L	cathepsin L [Sympagella nux]	Own contig	LOD99_11258	8.03 × 10-85	Cathepsin L
Cathepsin L	cathepsin L [Sympagella nux]	Own contig	LOD99_6637	3.02 × 10-84	Cathepsin L
Cathepsin L	cathepsin L [Sympagella nux]	Own contig	LOD99_6632	1.86 × 10-81	Cathepsin L
Cathepsin L	cathepsin L [Sympagella nux]	Own contig	LOD99_5135	4.93 × 10-73	Cathepsin L
Cathepsin L	cathepsin L [Sympagella nux]	Own contig	LOD99_7783	2.69 × 10-72	Cathepsin L
Cathepsin L	cathepsin L [Sympagella nux]	Own contig	LOD99_9107	1.42 × 10-71	Cathepsin L
Cathepsin L	cathepsin L [Sympagella nux]	Own contig	LOD99_29	3.92 × 10-53	Cathepsin L
Cathepsin L	cathepsin L [Sympagella nux]	Own contig	LOD99_14333	7.93 × 10-51	Cathepsin L
Cathepsin L	cathepsin L [Sympagella nux]	Own contig	LOD99_6910	1.12 × 10-45	Cathepsin L
Cathepsin L	cathepsin L [Sympagella nux]	Own contig	LOD99_16078	3.21 × 10-54	Cathepsin L
Cathepsin B	cathepsin B [Sympagella nux]	Own contig	LOD99_2071	3.21 × 10-51	Cathepsin B
Cathepsin B	cathepsin B [Sympagella nux]	Own contig	LOD99_8394	4.15 × 10-100	Cathepsin B
Cathepsin B	cathepsin B [Sympagella nux]	Own contig	LOD99_6927	1.35 × 10-64	Cathepsin B
Cathepsin B	cathepsin B [Sympagella nux]	Own contig	LOD99_2671	1.34 × 10-84	Cathepsin B
Cathepsin B	cathepsin B [Sympagella nux]	Own contig	LOD99_3637	2.26 × 10-100	Cathepsin B
NBCSA cotransporter	natriumbicarbonate silicic acid cotransporter [Suberites domuncula]	CAF32326.1	LOD99_7310	2.52 × 10-144	anion exchange protein 2-like [Biomphalaria glabrata]
arsB transporter (LSi2)	PREDICTED: putative transporter arsB [Amphimedon queenslandica]	XP_003386169.1	LOD99_7531	2.76 × 10-99	arsB transporter
arsB transporter (LSi2)	PREDICTED: putative transporter arsB [Amphimedon queenslandica]	XP_003386169.1	LOD99_12653	1.84 × 10-94	arsB transporter
Glassin	glassin, partial [Euplectella curvistellata]	BAS21353.1	LOD99_3750	1.32 × 10-54	Glassin, partial
Silicase	CA_SubDo [Suberites domuncula]	SFW10617.1	LOD99_7560	2.57 × 10-21	Carbonic anhydrase 20
Silicase	CA_SubDo [Suberites domuncula]	SFW10617.1	LOD99_966	3.45 × 10-18	Carbonic anhydrase 1-like
Silicase	CA_SubDo [Suberites domuncula]	SFW10617.1	LOD99_7562	1.96 × 10-15	Carbonic anhydrase 2-like
Silicase	CA_SubDo [Suberites domuncula]	SFW10617.1	LOD99_7559	4.38 × 10-12	Alpha carbonic anhydrase 5
Ferretin	ferretin [Amphimedon queenlsandica]	XP_019854974.1	LOD99_6480	5.72 × 10-70	Ferretin
Chitin synthase	chitin synthase CHS5611, partial [Spongilla lacustris]	AEI55441.1	LOD99_13043	4.08 × 10-100	Chitin synthase 6

<u>Table S21:</u> List of sequences used for phylogenetic analyses of silicatein and cathepsin (Fig. 6)

The corresponding alignment (*) is available at : https://zenodo.org/communities/oops_13

Sequence label	Protein	Organism	Accession number
PfiSi1	Silicatein	*Petrosia ficiformis*	sequence provided in the alignment*
AquSil	Silicatein	*Amphimedon queenslandica*	XP_003383103.1
PfiSil2	Silicatein	*Petrosia ficiformis*	sequence provided in the alignment
GcydoSil	Silicatein	*Geodia cydonium*	CAM57981.1
TaurSilA	Silicatein	*Tethya aurantium*	AAC23951.1
DjapoSil	Silicatein	*Discodermia japonica*	CBY80151.1
HokaSil	Silicatein	*Halichondria okadai*	BAB86343.1
Cmey_Sil	Silicatein	*Crateromorpha meyeri*	CAP49202.2
Mchuni_Sil	Silicatein	*Monoraphis chuni*	CAZ04880.1
LbaikSil	Silicatein	*Lubomirskia baikalensis*	CAH10753.1
PfiSil4	Silicatein	*Petrosia ficiformis*	sequence provided in the alignment
PfiSil3	Silicatein	*Petrosia ficiformis*	sequence provided in the alignment
EupleSil	Silicatein	*Euplectella aspergillum*	CBY80150.1
Cnu_L1	Cathepsin L	*Chondrilla caribea*	sequence provided in the alignment
CnuL6	Cathepsin L	*Chondrilla caribea*	sequence provided in the alignment
CnuL5	Cathepsin L	*Chondrilla caribea*	sequence provided in the alignment
CnuL3	Cathepsin L	*Chondrilla caribea*	sequence provided in the alignment
CowCathL2	Cathepsin L	*Capsaspora owzcarzacki*	EFW46557.1
HvuCathL	Cathepsin L	*Hydra vulgaris*	AAO65603.1
Hyalonema_populiferum_CathL	Cathepsin L	*Hyalonema populiferum*	sequence provided in the alignment
Sympagella_nux_CatL1	Cathepsin L	*Sympagella nux*	sequence provided in the alignment
Ircinia_fasculata_L1	Cathepsin L	*Ircinia fasciculata*	sequence provided in the alignment
Ircinia_fasculata_L3	Cathepsin L	*Ircinia fasciculata*	sequence provided in the alignment
AphisCatL	Cathepsin L	*Aphis gossypii*	CAD33266.1
Apis_mellifera_CatL	Cathepsin L	*Apis melifera*	XP_006557802.1
PfiCatL3	Cathepsin L	*Petrosia ficiformis*	sequence provided in the alignment
CnuL8	Cathepsin L	*Chondrilla caribea*	sequence provided in the alignment
PfiCatL2	Cathepsin L	*Petrosia ficiformis*	sequence provided in the alignment
PfiCatL1	Cathepsin L	*Petrosia ficiformis*	sequence provided in the alignment
CcaCathL4	Cathepsin L	*Corticium candelabrum*	sequence provided in the alignment
CnuL4	Cathepsin L	*Chondrilla caribea*	sequence provided in the alignment
Ircinia_fasculata_L2	Cathepsin L	*Ircinia fasciculata*	sequence provided in the alignment
Amphimedon_queenslandica_CatL1	Cathepsin L	*Amphimedon queenslandica*	XP_003391202.1
Rossella_fibulata_CatL	Cathepsin L	*Rosella fibulata*	sequence provided in the alignment
Sympagella_nux_CatL3_	Cathepsin L	*Sympagella nux*	sequence provided in the alignment
lcl|g4353.t1	Cathepsin L	*Oopsacas minuta*	sequence provided in the alignment
lcl|g12702.t1	Cathepsin L	*Oopsacas minuta*	sequence provided in the alignment
Hyalonema_populiferum_CatL1	Cathepsin L	*Hyalonema populiferum*	sequence provided in the alignment
Pheronema_raphanus_CatL1	Cathepsin L	*Pheronema raphanus*	sequence provided in the alignment
lcl|g10364.t1	Cathepsin L	*Oopsacas minuta*	sequence provided in the alignment
Aphrocallistes_vastus_Cathepsi	Cathepsin L	*Aphrocallistes vastus*	sequence provided in the alignment
Aphrocallistes_vastus_CathL4	Cathepsin L	*Aphrocallistes vastus*	sequence provided in the alignment
lcl|g11258.t1	Cathepsin L	*Oopsacas minuta*	sequence provided in the alignment
lcl|g6637.t1	Cathepsin L	*Oopsacas minuta*	sequence provided in the alignment
Sycon_coactum_CathL1	Cathepsin L	*Sycon coactum*	sequence provided in the alignment
Sycon_coactum_CathL2	Cathepsin L	*Sycon coactum*	sequence provided in the alignment
Aulosac_CatL2_	Cathepsin L	*Aulosaccus sp.*	ACU86972.1
Aulosac_CatL1_ACU86972.1	Cathepsin L	*Aulosaccus sp.*	ACU86973.1
lcl|g7783.t1	Cathepsin L	*Oopsacas minuta*	sequence provided in the alignment
lcl|g9107.t1	Cathepsin L	*Oopsacas minuta*	sequence provided in the alignment
Pheronema_raphanus_CatL	Cathepsin L	*Pheronema raphanus*	sequence provided in the alignment
lcl|g6632.t1	Cathepsin L	*Oopsacas minuta*	sequence provided in the alignment
CnuL7	Cathepsin L	*Chondrilla caribea*	sequence provided in the alignment
PfiCatL4	Cathepsin L	*Petrosia ficiformis*	sequence provided in the alignment
Bathy_sp_CatL2	Cathepsin L	*Bathydorus sp.*	ACU86975.1
Bathy_sp_CatL3	Cathepsin L	*Bathydorus sp.*	ACU86973.1
lcl|g5135.t1	Cathepsin L	*Oopsacas minuta*	sequence provided in the alignment
lcl|g6910.t1	Cathepsin L	*Oopsacas minuta*	sequence provided in the alignment
lcl|g29.t1	Cathepsin L	*Oopsacas minuta*	sequence provided in the alignment
CcaCatLl	Cathepsin L	*Corticium candelabrum*	sequence provided in the alignment
lcl|g14333.t1	Cathepsin L	*Oopsacas minuta*	sequence provided in the alignment
lcl|g16078.t1	Cathepsin L	*Oopsacas minuta*	sequence provided in the alignment
CcandeB	Cathepsin B	*Corticium candelabrum*	sequence provided in the alignment
PRefB1	Cathepsin B	*Petrosia ficiformis*	sequence provided in the alignment
AqueCatB	Cathepsin B	*Amphimedon queenslandica*	XP_003388852.1
IfasCatB2	Cathepsin B	*Ircinia fasciculata*	sequence provided in the alignment
PseuCatB	Cathepsin B	*Pseudospongosorites suberitoides*	sequence provided in the alignment
SdoCatB	Cathepsin B	*Suberites domuncula*	CAH04630.1
NvecCatB	Cathepsin B	*Nematostella vectensis*	EDO38122.1
CplicaCatB	Cathepsin B	*Cristaria plicata*	AEF32260.1
PfuctCatB	Cathepsin B	*Pinctada fucata*	ADX32985.1
CteneCatB	Cathepsin B	*Capitella teleta*	ELT94358.1
MmusCatB	Cathepsin B	*Mus musculus*	AAA37375.1
TadhCatB	Cathepsin B	*Trichoplax adhaerens*	EDV23587.1
SalpCatB	Cathepsin B	*Salpingoeca rosetta*	EGD76061.1
MbreviCatB	Cathepsin B	*Monosiga brevicollis*	EDQ84610.1
SlacuCatB	Cathepsin B	*Spongilla lacustris*	sequence provided in the alignment
ScoCatB2	Cathepsin B	*Sycon coactum*	sequence provided in the alignment
ScoCatB3	Cathepsin B	*Sycon coactum*	sequence provided in the alignment
LpannCatB	Cathepsin B	*Lithopennaeus vannamei*	ADI80349.1
AvastCatB3	Cathepsin B	*Aphrocallistes vastus*	sequence provided in the alignment
AvastCatB4	Cathepsin B	*Aphrocallistes vastus*	sequence provided in the alignment
CmeyerCatB	Cathepsin B	*Crateromorpha meyeri*	CAP17587.1
lcl|g2671.t1	Cathepsin B	*Oopsacas minuta*	sequence provided in the alignment
lcl|g3637.t1	Cathepsin B	*Oopsacas minuta*	sequence provided in the alignment
CnuCatB	Cathepsin B	*Chondrilla caribea*	sequence provided in the alignment
AvastCatB2	Cathepsin B	*Aphrocallistes vastus*	sequence provided in the alignment
lcl|g6927.t1	Cathepsin B	*Oopsacas minuta*	sequence provided in the alignment
AvastCatB1	Cathepsin B	*Aphrocallistes vastus*	sequence provided in the alignment
UmarCatB	Cathepsin B	*Uronema marinum*	AAR19103.1
SpurCatB	Cathepsin B	*Strongylocentrotus purpuratus*	XP_003729132.1
HmagniCatB	Cathepsin B	*Hydra magnipapillata*	XP_002166984.1
PeRefB2	Cathepsin B	*Petrosia ficiformis*	sequence provided in the alignment
CowarCatB	Cathepsin B	*Capsaspora owzcarzacki*	XP_004364814.1
lcl|g8394.t1	Cathepsin B	*Oopsacas minuta*	sequence provided in the alignment
lcl|g2071.t1	Cathepsin B	*Oopsacas minuta*	sequence provided in the alignment
IfasCatB1	Cathepsin B	*Ircinia fasciculata*	sequence provided in the alignment

1. Larroux C, Luke GN, Koopman P, Rokhsar DS, Shimeld SM, Degnan BM. Genesis and expansion of metazoan transcription factor gene classes. Mol Biol Evol. 2008;25(5):980–96.

2. Schenkelaars Q, Pratlong M, Kodjabachian L, Fierro-Constain L, Vacelet J, Le Bivic A, et al. Animal multicellularity and polarity without Wnt signaling. Sci Rep. 2017;7(1):15383.

3. Yang M, Derbyshire MK, Yamashita RA, Marchler-Bauer A. NCBI's Conserved Domain Database and Tools for Protein Domain Analysis. Curr Protoc Bioinformatics. 2020;69(1):e90.

4. Johnson LS, Eddy SR, Portugaly E. Hidden Markov model speed heuristic and iterative HMM search procedure. BMC Bioinformatics. 2010;11:431.

5. Liebeskind BJ, Hillis DM, Zakon HH. Convergence of ion channel genome content in early animal evolution. Proceedings of the National Academy of Sciences. 2015;112(8):E846–51.

6. Duschene M, Leys S. Sequences used for building HMMER profiles to search for SNAREs proteins. Zenodo; 2023. https://zenodo.org/record/7534968.

7. Riesgo A. alignment of cathepsin and silicatein sequences. Zenodo; 2022. https://zenodo.org/record/7534980.

8. Guindon S, Dufayard JF, Lefort V, Anisimova M, Hordijk W, Gascuel O. New algorithms and methods to estimate maximum-likelihood phylogenies: assessing the performance of PhyML 3.0. Syst Biol. 2010;59(3):307–21.

9. Lemoine F, Correia D, Lefort V, Doppelt-Azeroual O, Mareuil F, Cohen-Boulakia S, et al. NGPhylogeny.fr: new generation phylogenetic services for non-specialists. Nucleic Acids Res. 2019;47(W1):W260–5.

10. Vervoort M, Renard E. Alignment bHLH domains. Zenodo; 2022. https://zenodo.org/record/7473983.

11. Quevillon E, Silventoinen V, Pillai S, Harte N, Mulder N, Apweiler R, et al. InterProScan: protein domains identifier. Nucleic Acids Res. 2005;33:W116-120.

12. Edgar RC. MUSCLE: multiple sequence alignment with high accuracy and high throughput. Nucleic Acids Res. 2004;32(5):1792–7.

13. Pastrana CC, DeBiasse MB, Ryan JF. Sponges lack ParaHox genes. Piontkivska H, editor. Genome Biology and Evolution. 2019. https://academic.oup.com/gbe/advance-article/doi/10.1093/gbe/evz052/5374758.

14. Renard E. alignment of homeodomains of transcription factors of the ANTP family. Zenodo; 2022. https://zenodo.org/record/7494952.

15. Katoh K, Misawa K, Kuma K ichi, Miyata T. MAFFT: a novel method for rapid multiple sequence alignment based on fast Fourier transform. Nucleic Acids Res. 2002;30(14):3059–66.

16. Capella-Gutiérrez S, Silla-Martínez JM, Gabaldón T. trimAl: a tool for automated alignment trimming in large-scale phylogenetic analyses. Bioinformatics. 2009;25(15):1972–3.

17. Lefort V, Longueville JE, Gascuel O. SMS: Smart Model Selection in PhyML. Mol Biol Evol. 2017;34(9):2422–4.

18. Kumar S, Stecher G, Li M, Knyaz C, Tamura K. MEGA X: Molecular Evolutionary Genetics Analysis across Computing Platforms. Mol Biol Evol. 2018;35(6):1547–9.

19. Leys S. Alignment used for voltage gated ion channels phylogenetic analysis. Zenodo; 2023. https://zenodo.org/record/7534934

20. Leys S. alignment of potassium channels sequences. Zenodo; 2023. https://zenodo.org/record/7534942
